# Reactions of a Zn–Zn bond with main group carbene analogues as a prototypical case of reductive addition

**DOI:** 10.1038/s44160-025-00790-y

**Published:** 2025-04-29

**Authors:** Wenbang Yang, Andrew J. P. White, Mark R. Crimmin

**Affiliations:** Molecular Sciences Research Hub, Imperial College London, London, UK

**Keywords:** Organometallic chemistry, Organometallic chemistry, Chemical bonding

## Abstract

Oxidative addition most commonly involves the addition of a substrate to a metal centre. This reaction is fundamental across synthetic chemistry and underpins numerous catalytic methods. In the textbook description of oxidative addition reactions, a net increase in the formal oxidation state of the metal occurs with simultaneous bond breaking at the substrate. The majority of known oxidative addition reactions, however, involve substrates bearing relatively electronegative elements (for example, hydrogen, carbon, nitrogen, oxygen and halogens) and there has been little discussion of how addition processes may fundamentally change if substrates were constructed from more electropositive elements. Here we show that the zinc–zinc bonded complex, Cp*ZnZnCp* (Cp* = pentamethylcyclopentadienyl), which is isoelectronic with dihydrogen, undergoes facile addition to the metal (or semi-metal) centres of a series of main group carbene analogues based on silicon, aluminium, gallium or indium. Reactions proceed with complete breaking of the zinc–zinc bond and an increase in the coordination number of the central metal from two to four. Our analysis suggests that these addition processes are not oxidative, but rather there is likely a continuum of redox outcomes spanning oxidative, redox neutral and reductive. The addition of Cp*ZnZnCp* to silicon(II) provides the most compelling case for a prototypical reductive addition process.

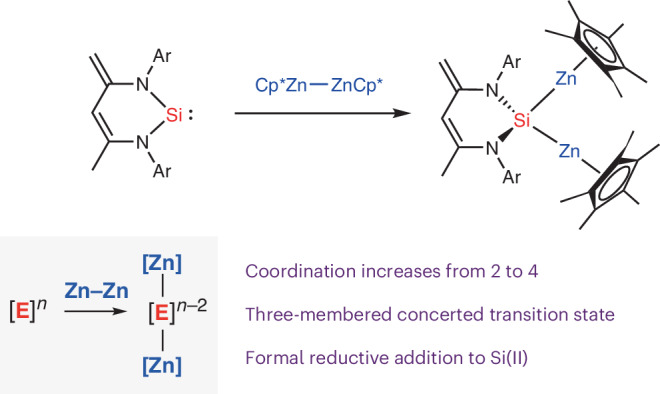

## Abstract (web summary)

The zinc–zinc bonded complex, Cp*ZnZnCp* (Cp* = pentamethylcyclopentadienyl), undergoes facile addition to the metal (or semi-metal) centres of a series of main group carbene analogues based on silicon, aluminium, gallium or indium. The addition of Cp*ZnZnCp* to silicon(II) provides a compelling case for a prototypical reductive addition process.

## Main

Oxidative addition^1^ and its microscopic reverse, reductive elimination, are fundamental steps in chemistry^2,3^. These steps are known to occur at discrete molecular complexes and at the surface of heterogeneous materials. Numerous value-added chemicals are created in catalytic processes that rely on oxidative addition and reductive elimination as key mechanistic steps. For example, catalysts that are used in hydrogenation typically operate by mechanisms in which metal sites react with dihydrogen (H_2_) by oxidative addition^4,5^. Oxidative addition of H_2_ to a metal site (M) involves donation of electron density from the *σ*-bond of H_2_ to an empty orbital of the metal, and back-donation from the metal site to the σ*-orbital of H_2_. Simultaneous depopulation of the *σ*-bond of dihydrogen with population of the σ*-orbital leads to breaking of the H–H *σ*-bond, which occurs with simultaneous making of two M–H *σ*-bonds and a change in the formal oxidation state at the metal from M to M^2+^. Similar processes can be found in textbook descriptions of mechanisms for hydrosilylation, hydroboration, isomerization, cross-coupling, hydroformylation and carbonylation^2^.

**Fig. 1:**
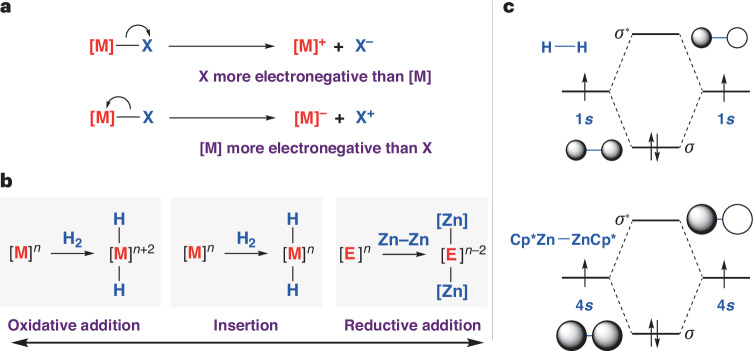
Oxidative addition reactions. **a**, IUPAC definition of formal oxidation states based on division of heteronuclear bonds and assignment of electrons based on Allen electronegativity. **b**, Continuum of redox outcomes for addition of H_2_ and Zn–Zn bonds to metal (and semi-metal) centres. **c**, Frontier molecular orbitals and the isoelectronic relationship between H_2_ and Cp*ZnZnCp*.

The description of this process as ‘oxidative’ is formalized in the International Union of Pure and Applied Chemistry (IUPAC) definition of oxidation state. Oxidation state is defined as the charge of the atom after its homonuclear bonds have been divided equally and the heteronuclear bonds assigned to the bonding partners according to the Allen electronegativity (Fig. 1a)^6,7^. In the case of dihydrogen, the underpinning assumption is that the hydrogen atoms are more electronegative than the metal site they are adding to, and as such the process is defined as oxidative from the perspective of the metal^8^. While a formalism, the description is intuitive and useful: it suggests a nucleophilic role of the hydrogen sites commonly involved in onwards steps (for example, migratory insertion). A philosophical question arises: if electron density in the M–H bond was to be shared evenly between the metal and hydrogen atoms, should the processes still be considered oxidative? Moreover, if the metal were more electronegative than hydrogen and the polarity of this bond reversed, M^δ−^–H^δ+^, would it be more suitable to describe this as a reductive addition? (Fig. 1b)^3^.

In this paper, we describe the addition of Cp*ZnZnCp* to a series of main group carbene analogues, including silicon(II), aluminium(I), gallium(I) and indium(I) compounds. The Zn–Zn bond of Cp*ZnZnCp* can be considered to be isolobal to the H–H bond, with *σ*- and σ*-molecular orbitals constructed from overlap of 4*s*–4s atomic orbitals rather than 1*s*–1*s* (Fig. 1c)^9–12^. Previous studies have shown that Cp*ZnZnCp* reacts with transition metal complexes to form addition products^13–16^. In the current case, the addition reactions involve complete breaking of the Zn–Zn bond and construction of two new Si–Zn, Al–Zn, Ga–Zn or In–Zn bonds, resulting in an increase in the coordination number of the main group centre from two to four. In the case of the gallium(I), the process is reversible, with both addition and elimination accessible under ambient conditions. In the case of indium(I), the initial product of addition is unstable and cannot be detected spectroscopically because it undergoes further reaction to form a tetrametallic array.

Based on the Allen electronegativities of the elements involved and the IUPAC definition of oxidation state, these reactions are all formally described as reductive addition processes. A deeper analysis of the mechanism of addition and electronic structure of the products through computational methods suggests the situation is nuanced. While these reactions are perhaps best described by a continuum of redox outcomes, the case of addition to silicon(II) provides the most convincing data for a prototypical reductive addition process.

## Results and discussion

**Fig. 2:**
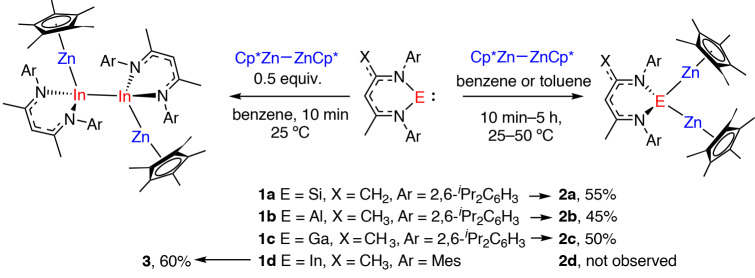
Synthesis of 2a–2c and 3. Addition of Cp*ZnZnCp* to heavier carbene analogues of silicon, aluminium, gallium and indium. Isolated yields given. THF, tetrahydrofuran; Mes, 2,4,6-trimethylphenyl.

Addition of Cp*ZnZnCp* to the heavier carbene analogues **1a**–**1c** (E = Si, Al, Ga) in benzene or toluene solution resulted in the formation of **2a**–**2c** (Fig. 2). These reactions were monitored by ^1^H NMR spectroscopy over a period of 10 min–5 h between 25 °C and 50 °C; in all cases product formation was indicated by a desymmetrization of the proton environments of the β-diketiminate ligand, along with an upfield shift of the methine resonance of the ligand backbone referenced against **1a**–**1c**. In the case of **2a**, the reaction was also characterized by a diagnostic ^29^Si NMR spectroscopy resonance observed at *δ* = +25.0 ppm, shifted upfield by Δ*δ* = 63.4 ppm compared with the parent silylene **1a**^17^. While **2a** and **2b** were generated cleanly in high yield, **2c** formed as a 9:1 equilibrium mixture with **1c** and Cp*ZnZnCp*. Variable-temperature ^1^H NMR spectroscopy studies were conducted on isolated samples of **2c** dissolved in toluene-*d*_8_. Data were recorded across a −80 °C to +80 °C temperature range and showed that the equilibrium position shifts entirely toward **2c** at lower temperatures^18^. A van’t Hoff analysis is consistent with the forward reaction having a Gibbs free energy of Δ*G*°_298K_ = –4.7 kcal mol^−1^. The process is reversible, as confirmed by a crossover experiment between **2c** and **1b** which resulted in complete conversion to **2b** and **1c**.

A similar reaction between the indium(I) complex **1d** (E = In)^19^ and Cp*ZnZnCp* did not led to isolation of the analogous compound **2d**, but instead resulted in the generation of a tetrametallic complex **3** derived from a 2:1 reaction stoichiometry. **2d** is proposed to be an intermediate is this reaction but was not stable enough to be observed spectroscopically or isolated. Related homometallic indium chains have been prepared through catenation of indium(I) complexes^20^. Additional experiments in which **1a**–**1d** (along with the germanium analogue of **1a**) were reacted with a series of complexes containing Zn–Zn and Mg–Mg bonds, under both thermal and photochemical conditions, failed to yield isolable products (Supplementary Tables 1 and 2).

**Fig. 3:**
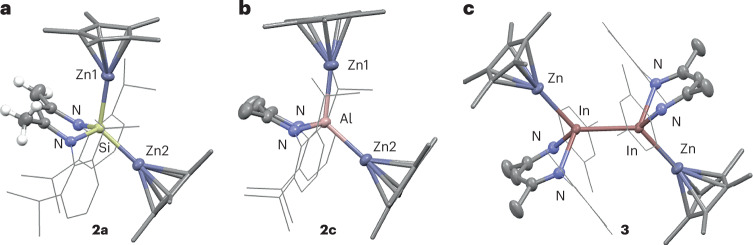
Crystal structures of 2a, 2c and 3. **a**–**c**, Structures of **2a** (**a**), **2c** (**b**) and **3** (**c**). Hydrogen atoms, with the exception of key methyl and the methylene group of **2c**, removed for clarity. Aryl groups shown as wireframe.

**2a**–**2c** could be crystallized and isolated in 45–55% yield. In the solid-state, **2a**–**2c** all demonstrated an approximate tetrahedral geometry at the silicon, aluminium or gallium centres (Fig. 3). The Zn–Zn distances are all in excess of 4 Å. These distances are beyond the sum of the covalent radii (Pyykkö, 2.36 Å (ref. ^21^); Pauling, 2.50 Å (ref. ^22^)) and are far greater than those experimentally determined in coordination complexes of Cp*ZnZnCp* with transition metal fragments which range from approximately 2.4 to 2.8 Å (refs. ^12–15,23–26^). The data suggest that complete breaking of the Zn–Zn bond occurs on reaction with the main group carbene analogue. The Si–Zn, Al–Zn and Ga–Zn distances in each compound are unsymmetrical and take values of 2.3268(6) and 2.3831(6) Å, 2.4102(9) and 2.4957(9) Å, and 2.3707(6) and 2.4576(6) Å, to the Zn2 and Zn1 atoms, respectively. All are within the sum of the covalent radii. Angles around the central main group element are distorted away from ideal tetrahedral geometry, the N–E–N angles are acute and range from 92.46(11)° to 101.12(9)°, while the Zn–E–Zn angles are more obtuse, taking values from 123.98(3)° to 133.84(2)°. **3** demonstrates an alternative structure comprised of an array of Zn–In–In–Zn metals with a centre of symmetry in the middle of the In–In bond. The In–Zn distance is 2.5360(4) Å, while the In–In distance is 2.7604(3) Å. The four metal atoms are coplanar, with the zinc sites adopting an anti-periplanar geometry with respect to the In–In bond.

**Fig. 4:**
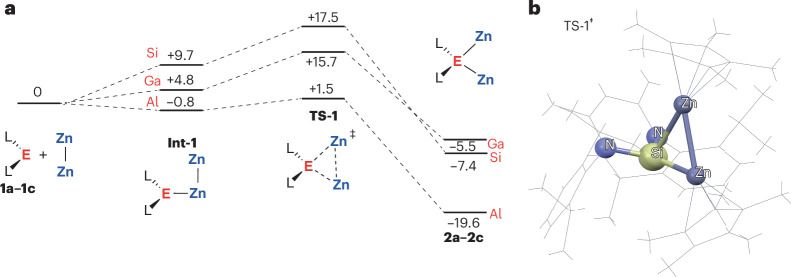
Calculated reaction pathway for the addition of Cp*ZnZnCp* to 1a–1c. **a**, Calculated potential energy surface. **b**, Transition state geometry for E = Si. Gibbs energies reported in kcal mol^−1^. Calculations G09: M06L/def2TZVPP/PCM (benzene)//M06L/6-31G**/6-311+G*/ SDDAll (Al, Zn). L_2_, β-diketiminate ligand.

Pathways for the addition of Cp*ZnZnCp* to **1a**–**1c** were calculated by density functional theory (Fig. 4a). **1a**–**1c** can all be considered low-valent complexes; each has an available lone-pair^27^. In all cases, the reaction is proposed to occur through formation of an intermediate **Int-1**, derived from coordination of the low-valent fragment to one of the zinc centres of Cp*ZnZnCp*. This coordination event is endergonic for both silicon and gallium (Δ*G*°_298K_ = +4.8 to +9.7 kcal mol^−1^) but slightly exergonic for aluminium (Δ*G*°_298K_ = −0.8 kcal mol^−1^). The Cp*ZnZnCp* moiety in **Int-1** is both desymmetrized and polarized and the system is set up to reach the addition transition state, **TS-1**. **TS-1** is a low-energy (Δ*G*^‡^_298K_ = 2.3 to 17.5 kcal mol^−1^) three-centred transition state, reminiscent of those found in oxidative addition mechanisms for both main group and transition metal complexes (Fig. 4b)^28–31^. **TS-1** appears late in the reaction pathway, with Zn–Zn bond breaking and Zn–E bond breaking almost complete as it is traversed. While formation of **2a**–**2c** is calculated to be downhill, the gallium analogue is exergonic by only Δ*G*°_298K_ = –5.5 kcal mol^−1^, consistent with the potential for this reaction to be reversible within the expected accuracy of the calculations. For **1d**, a low-energy pathway for reaction with Cp*ZnZnCp* to form **2d** followed by addition of a second equivalent of **1d** to form the thermodynamic product **3** was identified (Supplementary Fig. 18).

In terms of the Allen scale of electronegativity, zinc (*χ*_Zn_ = 1.59) is the least electronegative element in all these complexes. The main group centres that undergo the addition reaction are all more electronegative (*χ*_Si_ = 1.92, *χ*_Al_ = 1.61, *χ*_Ga_ = 1.76, *χ*_In_ = 1.66). Hence, based on the formal definition, these processes should be described as reductive additions. This definition, however, is fragile. The difference in electronegativity between aluminium and zinc is only 0.02 on the Allen scale, so small as to consider electron localization on either element insubstantial. The differences in electronegativities are also scale dependent (for example, Pauling, Mulliken–Jaffe, Alfred–Rochow). Hence, for aluminium, gallium and indium, the reactions could as well be described as oxidative or redox neutral (insertion) if the strict IUPAC definition was not followed. That said, silicon is more electronegative than zinc regardless of the electronegativity scale used, and hence this example may constitute a prototypical reductive elimination process.

**Fig. 5:**
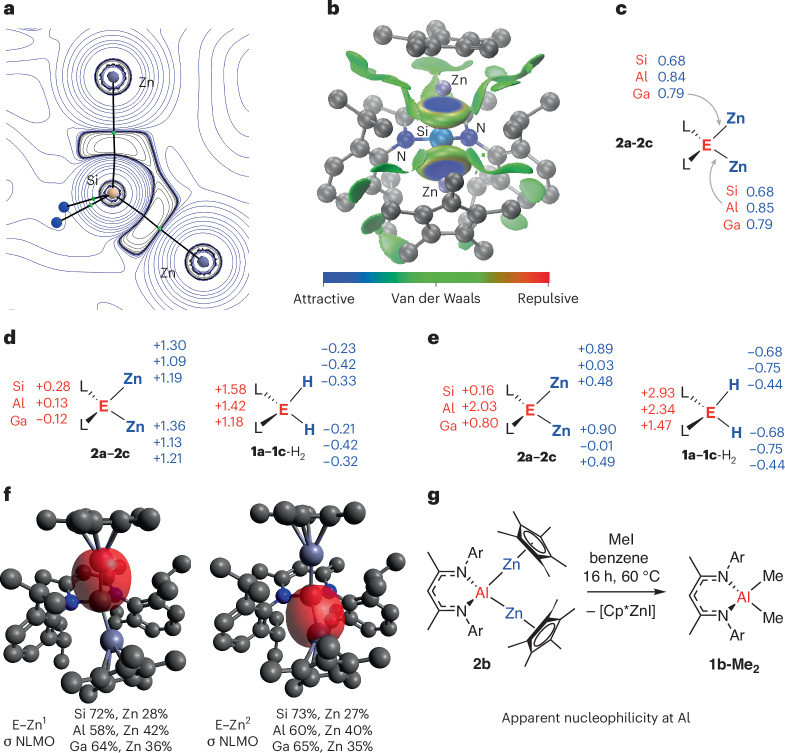
Bonding analysis and reactivity of 2a–2c. **a** AIM plot of **2a** showing Laplacian of electron density ∇^2^*ρ*(*r*) between silicon and zinc. **b**, Independent gradient model based on Hirshfield partition analysis of **2b**. **c**, Wiberg bond indices for the E–Zn bonds in **2a**–**2c**. **d**, Natural population analysis charges at key atomic sites on **2a**–**2c**. **e**, AIM charges at key atomic sites in **2a**–**2c**. **f**, NLMOs for the E–Zn bonds of **2a** along with calculated coefficients for partitioning of NLMOs between E and Zn. **g**, Reaction of **2b** with MeI. Ar, 2,6-di-iso-propylphenyl.

The bonding in **2a**–**2c** was interrogated through a suite of computational methods. There is no suggestion of any significant Zn–Zn interaction in these compounds. Atoms in molecules (AIM) treatment returned bond paths and associated bond critical points from the central main group element to each of the zinc atoms in **2a**–**2c**, with no bond paths between the two zinc atoms themselves (Fig. 5a). Metrics associated with the bond critical points are consistent with their description as weak covalent bonds^32^. Independent gradient model based on Hirshfield partition (IGMH) analysis also supports an attractive covalent interaction with no or little Zn–Zn bonding (Fig. 5b)^33^. Natural bonding orbital analysis suggests that the E–Zn bonds in **2a**–**2c** are best formulated as polar covalent interaction. Wiberg bond indices take average values of 0.68, 0.84 and 0.79 for the E–Zn bonds of **2a**, **2b** and **2c**, respectively (Fig. 5c). In combination, the calculations support the proposal that complete Zn–Zn bond breaking occurs on addition to **1a**–**1c** and these species are best formulated as tetrahedral four-coordinate complexes with zinc sites covalently bonding to the central atom.

Additional computational analysis was undertaken to understand the polarization of the newly formed covalent bonds in **2a**–**2c**. Although care should be taken to not convolute calculated atomic charges with formal oxidation states, in general the analysis suggests that the site of addition accumulates negative charge as the reaction proceeds. Natural population analysis charges show accumulation of negative charge on the central main group element of **2a**–**2c**, with the zinc centres being the most electropositive sites in these molecules. For comparison, the calculated products from reactions between **1a**–**1c** and H_2_ (refs. ^34,35^), a classical oxidative addition process, were also considered^36–38^. These species demonstrate greater charge separation than **2a**–**2c**, consistent with a larger ionic contribution and the expected charge depletion at the main group centre and charge accumulation at the hydride ligands (Fig. 5d). Similar conclusions are drawn from inspection of fragment charges which consider delocalization of the charge across the supporting ligands (Supplementary Information). The analysis based on calculated AIM charges for **2a**–**2c** is less definitive, with only the silicon analogue of the series demonstrating significant charge accumulation at the central main group metal (Fig. 5e). Natural localized molecular orbitals (NLMOs) were used to further interrogate the distribution of electron density between zinc and E. For **2a**–**2c**, NLMOs describing the *σ*-bonding orbitals between the central main group element and zinc could be identified. In each case, these orbitals are polarized toward the central main group element (for example, silicon, aluminium, gallium or indium), having the largest coefficients on this element and smallest coefficients on zinc in the series; again the silicon analogue has the largest coefficients on the central atom (Fig. 5f).

In combination, the calculations suggest that **2a**–**2c** contain polar covalent bonds that are polarized toward the central main group element (silicon, aluminium or gallium). As such, it could be expected that these species might be nucleophilic at this site. Addition of methyl iodide (MeI) to **2b** over 16 h at 60 °C resulted in the formation of the corresponding dimethyl aluminium compound **1b-Me**_**2**_ and Cp*ZnI as major reaction products. Although small amounts of Cp*ZnMe were also observed in this reaction, the implication is that the aluminium site of **2b** is nucleophilic (Fig. 5g).

## Conclusions

We report reactions of a Zn–Zn bonded complex, Cp*ZnZnCp*, with a series of main group carbene analogues bearing on low-valent silicon, aluminium, gallium and indium centres. In each case, the reactions occur with addition of the Zn–Zn bond to the low-valent centre, increasing the coordination number from 2 to 4. Although it is tempting to describe this as an oxidative addition process, the description is not strictly consistent with the IUPAC definition of oxidation state based on the Allen electronegativity scale. Formally these are reductive addition processes. Computational analysis of the reaction pathway and electronic structure of the products reveals a more nuanced picture, which is best considered by a continuum of redox outcomes ranging from oxidative through to reductive. The concept probably applies to transition metal as well as main group complexes^1,2^. Calculations on [Ni(PMe_3_)_3_(ZnCp*)(ZnMe)], originally reported by Fischer and co-workers^15^, suggest that this species shows increased charge build up at nickel compared with [Ni(PMe_3_)_3_(H)_2_] (Supplementary Figs. 68–71). Moreover, the three-fold addition of CpBeBeCp to [Ni(COD)_2_] to form [Ni(BeCp)_6_] was recently reported by Boronski and Aldridge^39^, and these authors describe the process as a ‘reductive addition’.

These results highlight the limitations of ‘normative scientific language’ in describing chemical reactivity. We suggest that addition reactions should not blindly be called oxidative. Rather it might be more constructive to consider a continuum of redox outcomes. In some special circumstances, such as the case of the addition of Cp*ZnZnCp* to a silylene complex reported herein, these may even be termed reductive addition processes.

## Methods

### Example procedure: preparation of 2a

In a glovebox, {DippNC(=CH_2_)CH(Me)NDipp}Si (20.0 mg, 0.045 mmol, 1 equiv.) and Cp*ZnZnCp* (18.1 mg, 0.045 mmol, 1 equiv.) were dissolved in C_6_D_6_ (0.6 ml) and transferred to a J. Young NMR tube. The reaction mixture was kept at 50 °C for 3 h. A ^1^H NMR spectrum was taken at this time point and showed the full conversion to **2a**. The J. Young NMR tube was returned to the glovebox, the solvent was removed under vacuum and the crude product dissolved in 1 ml diethyl ether/*n*-pentane (1:10 v/v) mixture. The solution was filtered into a 4-ml vial and then placed in a freezer in the glovebox (−35 °C) for 2 days. Colourless crystals (**2a**) were successfully obtained. The filtrated crystals were washed with cold *n*-pentane (3 × 1 ml) and then dried in vacuo. Yield: 21 mg, 0.025 mmol, 55%.

^1^H NMR (C_6_D_6_, 298 K, 400 MHz) *δ* (ppm): 7.22–7.18 (m, 1H, ArC*H*), 7.16–7.14 (m, 1H, ArC*H*), 7.14–7.12 (m, 2H, ArC*H*), 7.12–7.10 (m, 1H, ArC*H*), 7.10–7.07 (m, 1H, ArC*H*), 5.55 (s, 1H, CH_2_CC*H*C(CH_3_)), 4.07 (s, 1H, NCC*H*_*2*_), 3.64 (hept, ^3^*J*_H–H_ = 6.7 Hz, 1H, (C*H*(CH_3_)(CH_3_)), 3.48 (hept, ^3^*J*_H–H_ = 6.8 Hz, 1H, (C*H*(CH_3_)(CH_3_)), 3.29 (s, 1H, NCC*H*_*2*_), 2.85 (hept, ^3^*J*_H–H_ = 6.8 Hz, 1H, (C*H*(CH_3_)(CH_3_)), 2.84 (hept, ^3^*J*_H–H_ = 6.8 Hz, 1H, (C*H*(CH_3_)(CH_3_)), 2.18 (s, 15H, Cp*Me*), 1.55 (s, 15H, Cp*Me*), 1.49 (s, 3H, NCC*H*_*3*_), 1.45 (d, ^3^*J*_H–H_ = 6.7 Hz, 3H, (CH(CH_3_)(C*H*_*3*_)), 1.41 (d, ^3^*J*_H–H_ = 7.0 Hz, 3H, (CH(CH_3_)(C*H*_*3*_)), 1.40 (d, ^3^*J*_H–H_ = 6.8 Hz, 3H, (CH(CH_3_)(C*H*_*3*_)), 1.39 (d, ^3^*J*_H–H_ = 6.9 Hz, 3H, (CH(CH_3_)(C*H*_*3*_)), 1.35 (d, ^3^*J*_H–H_ = 7.0 Hz, 3H, (CH(CH_3_)(C*H*_*3*_)), 1.32 (d, ^3^*J*_H–H_ = 6.9 Hz, 3H, (CH(CH_3_)(C*H*_*3*_)), 1.30 (d, ^3^*J*_H–H_ = 6.7 Hz, 3H, (CH(CH_3_)(C*H*_*3*_)), 1.28 (d, ^3^*J*_H–H_ = 6.9 Hz, 3H, (CH(CH_3_)(C*H*_*3*_)). ^13^C NMR (C_6_D_6_, 298 K, 101 MHz) *δ* (ppm): 153.1 (N*C*CH_2_), 149.2 (N*C*(CH_3_)), 148.4 (Ar-*C*), 148.3 (Ar-*C*), 147.3 (Ar-*C*), 146.3 (Ar-*C*), 142.6 (Ar-*C*), 126.6 (Ar-*C*H), 126.2 (Ar-*C*H), 124.7 (Ar-*C*H), 124.6 (Ar-*C*H), 124.5 (Ar-*C*H), 124.4 (Ar-*C*H), 109.7 (*Cp*Me), 108.6 (*Cp*Me), 105.0 (CH_2_C*C*HC(CH_3_)), 86.2 (NC*C*H_2_), 30.4 (*C*H(CH_3_)(CH_3_)), 30.3 (*C*H(CH_3_)(CH_3_)), 29.1 (*C*H(CH_3_)(CH_3_)), 28.7 (*C*H(CH_3_)(CH_3_)), 27.5 (CH(CH_3_)(*C*H_3_)), 26.9 (CH(CH_3_)(*C*H_3_)), 26.8 (CH(CH_3_)(*C*H_3_)), 26.1 (CH(CH_3_)(*C*H_3_)), 25.9 (CH(CH_3_)(*C*H_3_)), 25.8 (CH(CH_3_)(*C*H_3_)), 25.6 (CH(CH_3_)(*C*H_3_)), 25.0 (CH(CH_3_)(*C*H_3_)), 22.4 (NC(*C*H_3_)), 11.4 (Cp*Me*), 10.1 (Cp*Me*). ^29^Si NMR (C_6_D_6_, 298 K, 99 MHz) *δ* (ppm): 25.0 (s, Zn–*Si*–Zn). Analysis: Calculated (C_49_H_70_N_2_SiZn_2_): C, 69.57; H, 8.34; N, 3.31. Found: C, 69.50; H, 8.64; N, 3.09.

## Supplementary information

Supplementary Information Experimental details, Supplementary Figs. 1–71 and Tables 1–144.

Supplementary Data 1 X-ray crystallographic data for **2a**, CCDC 2350790.

Supplementary Data 2 X-ray crystallographic data for **2b**, CCDC 2350788.

Supplementary Data 3 X-ray crystallographic data for **2c**, CCDC 2350789.

Supplementary Data 4 X-ray crystallographic data for **3**, CCDC 2410821.

Supplementary Data 5 Computational coordinates.

## Data Availability

Crystallographic data for the structures reported in this Article have been deposited at the Cambridge Crystallographic Data Centre, under deposition numbers CCDC 2350790 (**2a**), 2350788 (**2b**), 2350789 (**2c**) and 2410821 (**3**). Experimental procedures, details of calculations and spectroscopic data are available as Supplementary Information associated with this article, as are the cartesian coordinates for the density functional theory calculated stationary points (*xyz*).

## References

[1] Collman, J. P. & Roper, W. R. Preparation and oxidative addition reactions of a monomeric ruthenium(0) complex. *J. Am. Chem. Soc.* **87**, 4008–4009 (1965).

[2] Hartwig, J. F. (ed.). *Organotransition Metal Chemistry. From Bonding to Catalysis* (University Science Books, 2010).

[3] Labinger, J. A. Tutorial on oxidative addition. *Organometallics* **34**, 4784–4795 (2015).

[4] Vaska, L. & DiLuzio, J. W. Activation of hydrogen by a transition metal complex at normal conditions leading to a stable molecular dihydride. *J. Am. Chem. Soc.* **84**, 679–680 (1962).

[5] Halpern, J., Okamoto, T. & Zakhariev, A. Mechanism of the chlorotris(triphenylphosphine) rhodium(I)-catalyzed hydrogenation of alkenes. The reaction of chlorodihydridotris(triphenylphosphine)rhodium(III) with cyclohexene. *J. Mol. Catal.* **2**, 65–68 (1977).

[6] Karen, P., McArdle, P. & Takats, J. Comprehensive definition of oxidation state (IUPAC recommendations 2016). *Pure Appl. Chem.* **88**, 831–839 (2016).

[7] ‘Oxidation State’ in *IUPAC Compendium of Chemical Terminology*, 3rd ed. (International Union of Pure and Applied Chemistry, 2006), online version 3.0.1, 2019. 10.1351/goldbook.O04365

[8] Mann, J. B., Meek, T. L., Knight, E. T., Capitani, J. F. & Allen, L. C. Configuration energies of the *d*-block elements. *J. Am. Chem. Soc.* **122**, 5132–5137 (2000).

[9] Resa, I., Carmona, E., Gutierrez-Puebla, E. & Monge, A. Decamethyldizincocene, a stable compound of Zn(I) with a Zn–Zn bond. *Science* **305**, 1136–1138 (2004). 15326350 10.1126/science.1101356

[10] Río, D., del, Galindo, A., Resa, I. & Carmona, E. Theoretical and synthetic studies on [Zn_2_(η^5^‐C_5_Me_5_)_2_]: analysis of the Zn–Zn bonding interaction. *Angew. Chem. Int. Ed.* **44**, 1244–1247 (2005). 10.1002/anie.200462175 15662655

[11] Grirrane, A. et al. Zinc−zinc bonded zincocene structures. synthesis and characterization of Zn_2_(η^5^-C_5_Me_5_)_2_ and Zn_2_(η^5^-C_5_Me_4_Et)_2_. *J. Am. Chem. Soc.* **129**, 693–703 (2007). 17227033 10.1021/ja0668217

[12] Ayala, R., Carmona, E. & Galindo, A. The dizinc bond as a ligand: a computational study of elongated dizinc bonds. *Inorg. Chim. Acta* **470**, 197–205 (2018).

[13] Bollermann, T., Freitag, K., Gemel, C., Seidel, R. W. & Fischer, R. A. Reactivity of [Zn_2_Cp*_2_] toward transition metal complexes: synthesis and characterization of [Cp*M(ZnCp*)_3_] (M = Ni, Pd, Pt). *Organometallics* **30**, 4123–4127 (2011).

[14] Freitag, K. et al. The *σ*‐aromatic clusters [Zn_3_]^+^ and [Zn_2_Cu]: embryonic brass. *Angew. Chem. Int. Ed.* **54**, 4370–4374 (2015). 10.1002/anie.201410737 25676739

[15] Freitag, K. et al. Zn···Zn interactions at nickel and palladium centers. *Chem. Sci.* **7**, 6413–6421 (2016). 28451097 10.1039/c6sc02106a PMC5355958

[16] Hidalgo, N., Romero-Pérez, C., Maya, C., Fernández, I. & Campos, J. Reactivity of [Pt(P^t^Bu_3_)_2_] with zinc(I/II) compounds: bimetallic adducts, Zn–Zn bond cleavage, and cooperative reactivity. *Organometallics* **40**, 1113–1119 (2021). 34602699 10.1021/acs.organomet.1c00088 PMC8479860

[17] Driess, M., Yao, S., Brym, M., Wüllen, Cvan & Lentz, D. A new type of N-heterocyclic silylene with ambivalent reactivity. *J. Am. Chem. Soc.* **128**, 9628–9629 (2006). 16866506 10.1021/ja062928i

[18] Ganesamoorthy, C., Bläser, D., Wölper, C. & Schulz, S. Temperature‐dependent electron shuffle in molecular group 13/15 intermetallic complexes. *Angew. Chem. Int. Ed.* **53**, 11587–11591 (2014). 10.1002/anie.201406304 25196650

[19] Hill, M. S., Hitchcock, P. B. & Pongtavornpinyo, R. Solid- and solution-state structures of indium ‘alkene analogues’. *Dalton Trans*. 731–733 (2007). 10.1039/b616747k 17279243

[20] Hill, M. S., Hitchcock, P. B. & Pongtavornpinyo, R. A linear homocatenated compound containing six indium centers. *Science* **311**, 1904–1907 (2006). 16574862 10.1126/science.1123945

[21] Pyykkö, P. & Atsumi, M. Molecular single‐bond covalent radii for elements 1–118. *Chem. Eur. J.* **15**, 186–197 (2008). 10.1002/chem.200800987 19058281

[22] Pauling, L. Atomic radii and interatomic distances in metals. *J. Am. Chem. Soc.* **69**, 542–553 (1947).

[23] Cadenbach, T. et al. Twelve one‐electron ligands coordinating one metal center: structure and bonding of [Mo(ZnCH_3_)_9_(ZnCp*)_3_]. *Angew. Chem. Int. Ed.* **47**, 9150–9154 (2008). 10.1002/anie.200802811 18846517

[24] Cadenbach, T. et al. Molecular alloys, linking organometallics with intermetallic Hume–Rothery phases: the highly coordinated transition metal compounds [M(ZnR)_*n*_] (*n* ≥ 8) containing organo-zinc ligands. *J. Am. Chem. Soc.* **131**, 16063–16077 (2009). 19827777 10.1021/ja904061w

[25] Chen, M., Jiang, S., Maron, L. & Xu, X. Transition metal-induced dehydrogenative coupling of zinc hydrides. *Dalton Trans.* **48**, 1931–1935 (2018). 10.1039/c8dt04651d 30560981

[26] Jiang, S. et al. Synthesis and reactivity of triangular heterometallic complexes containing Zn–Zn bond. *Inorg. Chem.* **61**, 8083–8089 (2022). 35533341 10.1021/acs.inorgchem.2c00956

[27] Parkin, G. Valence, oxidation number, and formal charge: three related but fundamentally different concepts. *J. Chem. Ed.* **83**, 791 (2006).

[28] Villegas-Escobar, N., Gutiérrez-Oliva, S. & Toro-Labbé, A. Catalytic mechanism of H_2_ activation by a carbenoid aluminum complex. *J. Phys. Chem. C* **119**, 26598–26604 (2015).

[29] García‐Rodeja, Y., Bickelhaupt, F. M. & Fernández, I. Understanding the oxidative addition of *σ*‐bonds to group 13 compounds. *Chem. Eur. J.* **22**, 13669–13676 (2016). 27439790 10.1002/chem.201602505

[30] Schoeller, W. W. & Frey, G. D. Oxidative addition of *π*-bonds and *σ*-bonds to an Al(I) center: the second-order carbene property of the AlNacNac compound. *Inorg. Chem.* **55**, 10947–10954 (2016). 27739674 10.1021/acs.inorgchem.6b01488

[31] Zhang, X. & Cao, Z. Insight into the reaction mechanisms for oxidative addition of strong *σ*-bonds to an Al(I) center. *Dalton Trans.* **45**, 10355–10365 (2016). 27249667 10.1039/c6dt01154c

[32] Matta, C. F. & Boyd, R. *An Introduction to the Quantum Theory of Atoms in Molecules* (Wiley-VCH, 2007).

[33] Lu, T. & Chen, Q. Independent gradient model based on Hirshfeld partition: a new method for visual study of interactions in chemical systems. *J. Comput. Chem.* **43**, 539–555 (2022). 35108407 10.1002/jcc.26812

[34] Roy, M. M. D. et al. Molecular main group metal hydrides. *Chem. Rev.* **121**, 12784–12965 (2021). 34450005 10.1021/acs.chemrev.1c00278

[35] Stoelzel, M., Präsang, C., Inoue, S., Enthaler, S. & Driess, M. Hydrosilylation of alkynes by Ni(CO)_3_‐stabilized silicon(II) hydride. *Angew. Chem. Int. Ed.* **51**, 399–403 (2012). 10.1002/anie.201105722 22105836

[36] Chu, T., Korobkov, I. & Nikonov, G. I. Oxidative addition of *σ*-bonds to an Al(I) center. *J. Am. Chem. Soc.* **136**, 9195–9202 (2014). 24893309 10.1021/ja5038337

[37] Morris, L. J., Rajeshkumar, T., Okumura, A., Maron, L. & Okuda, J. Solvent‐dependent oxidative addition and reductive elimination of H_2_ across a gallium–zinc bond. *Angew. Chem. Int. Ed.* **61**, e202208855 (2022). 10.1002/anie.202208855 PMC9544028 35833688

[38] Seifert, A., Scheid, D., Linti, G. & Zessin, T. Oxidative addition reactions of element–hydrogen bonds with different polarities to a gallium(I) compound. *Chem. Eur. J.* **15**, 12114–12120 (2009). 19780109 10.1002/chem.200901403

[39] Boronski, J. T., Crumpton, A. E. & Aldridge, S. A crystalline NiX_6_ complex. *J. Am. Chem. Soc.* **146**, 35208–35215 (2024). 39668527 10.1021/jacs.4c12125 PMC11673578

