# Supplementary material for: Reactions of a Zn–Zn bond with main group carbene analogues as a prototypical case of reductive addition
Source: Nat Synth. 2025 Apr 29;4(8):995–1000. doi: 10.1038/s44160-025-00790-y (PMC12334357; doi:10.1038/s44160-025-00790-y)
Supplement: Supplementary file 1 — Experimental details, Supplementary Figs. 1–71 and Tables 1–144. [file 44160_2025_790_MOESM1_ESM.pdf]

nature synthesis

Supplementary information https://doi.org/10.1038/s44160-025-00790-y

Reactions of a Zn–Zn bond with main group carbene analogues as a prototypical case of reductive addition

In the format provided by the authors and unedited

Table of Contents

1) General Experimental **S2**

2) Experimental Methods **S3**

2.1) Preparation of **2a-2c** and **3** S3

3) Single Crystal X-ray Diffraction Data **S13**

4) Reactivity of 2a-c **S18**

4.1) Reaction of **2b** with MeI S18

4.2) Crossover experiment between **2c** and **1b** S23

5) Van't Hoff analysis **S24**

6) DFT Studies **S26**

6.1) Computational methods S26

6.2) Calculated Reaction Pathway S28

6.3) Optimized structures S32

6.4) NBO Analysis S47

6.5) QTAIM Analysis S71

6.6) ETS-NOCV S84

6.7) IBO Analysis S91

6.8) IGMH Analysis S99

6.9) NCIPLOT Analysis S113

6.10) Energies for the Homolytic Cleavage of E–Zn Bonds in **2a-c** and **3** S117

6.11) Calculated NPA and AIM Charges for Addition of Zn–Zn and H–H bonds to Ni and Ir centres S119

7) NMR Spectroscopy **S121**

8) XYZ Coordinates **S125**

9) References **S152**

1) General experimental

Standard schlenk line and glovebox techniques were used for all manipulations under an inert atmosphere of dinitrogen or argon unless otherwise stated. NMR scale reactions were performed in J. Young NMR tubes. A MBraun Labmaster glovebox was employed, operating at <0.1 ppm O_2 and <0.1 ppm H_2O.

Instruments: ^{1}H, ^{13}C NMR spectra were recorded on BRUKER 400 MHz or 500 MHz machines, and referenced against $SiMe_4$ (^{1}H, ^{13}C). All peaks are referenced against residual solvent and values are quoted in ppm. Data were processed using the MestReNova software. Where needed, chemical shifts were assigned with the assistance of 2D NMR (COSY, HSQC, HMBC, DEPTQ) spectra. The coupling constants (J) are reported in Hertz (Hz). The following abbreviations are used to define multiplicities: s (singlet), d (doublet), t (triplet), q (quadruplet), hept. (heptet), dd (doublet of doublets), ddd (doublet of doublets of doublets), dt (doublet of triplets), td (triplet of doublets), m (multiplet).

Single crystal X-ray data was obtained on Agilent Diffraction Xcalibur PX Ultra A and Xcalibur 3 E diffractometers, and the structures were refined using the SHELXTL, SHELX-97, and SHELX-2013 program systems.

Photolysis experiments were carried out using a Spectral Energy mercury arc lamp (400 W) or Kessil lamps (40 W, 390 nm) with samples contained in a J. Young's NMR tube.

UV-Vis spectra were collected in a Agilent Technologies Cary 60 UV-VIS. For the photochemical reactions, Kessil lamps (40 W, 428 nm and 390 nm) were used at 5 cm distance of the reaction vessels

Elemental analyses were performed by Elemental Labs (https://www.elementallab.co.uk/).

Chemicals: Solvents were dried over activated alumina from a solvent purification system (SPS) based upon the Grubbs design and de-gassed before use. Glassware was dried for >6 h prior to use at 120 °C. Benzene-d$_6$ and Toluene-d$_8$ were de-gassed and stored over 3 Å molecular sieves before use. All reagents were acquired from Sigma Aldrich (Merck), Fluorochem, or VWR and used without further purification unless specified. ^{Dipp}BDIAl (^{Dipp}BDI = {DippNC(Me)}$_2$CH, Dipp = 2,6-di-isopropylphenyl)[S1], ^{Dipp}BDIGa[S2], {DippNC(=CH$_2$)CH(Me)NDip}Si[S3] , {DippNC(=CH$_2$)CH(Me)NDip}Ge[S4], (^{Mes}BDIMg)$_2$ (^{Mes}BDI = {MesNC(Me)}$_2$CH)[S5-S6], (^{Dipp}BDIMg)$_2$[S5-S6], ^{Mes}BDIIn[S7], ^{Dipp}BDIIn[S8], ^{Xyl}BDIIn (^{Xyl}BDI = {XylNC(Me)}$_2$CH)[S9], (^{Mes}BDIZn)$_2$ and Cp*ZnZnCp* (Cp* = 1,2,3,4,5-pentamethylcyclopenta-1,3-diene) were prepared by literature procedures.[S10-S12]

2) Experimental Methods
2.1 Preparation of Compounds

Preparation of 2a

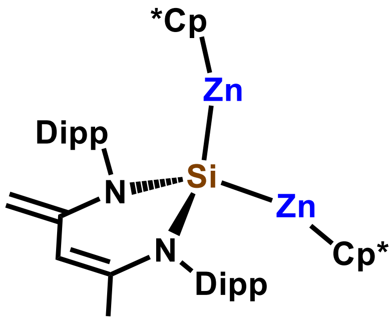

In a glovebox, {DippNC(=CH_2)CH(Me)NDipp}Si (20.0 mg, 0.045 mmol, 1 eq) and Cp*ZnZnCp* (18.1 mg, 0.045 mmol, 1 eq) were dissolved in C_6D_6 (0.6 mL) and transferred to a J. Young NMR tube. The reaction mixture was kept at 50 °C for 3h. A ^{1}H NMR spectrum was taken at this time point and showed the full conversion to **2a**. The J. Young NMR tube was returned to the glovebox, the solvent was removed under vacuum and the crude dissolved in 1 mL Et_2O/n-pentane (1:10 v: v) mixture. The solution was filtered into a 4 mL vial and then placed in the glovebox freezer (–35 °C) for 2 days. Colorless crystals (**2a**) were successfully obtained. The filtrated crystals were washed with cold n-pentane (3 x 1mL) and then dried in vacuo. **Yield: 21 mg,** 0.025 mmol, 55%.

^{1}H NMR (C_6D_6, 298 K, 400 MHz) δ: 7.22 – 7.18 (m, 1H, ArC***H***), 7.16 – 7.14 (m, 1H, ArC***H***), 7.14 – 7.12 (m, 2H, ArC***H***), 7.12 – 7.10 (m, 1H, ArC***H***), 7.10 – 7.07 (m, 1H, ArC***H***), 5.55 (s, 1H, CH_2CC***H***C(CH_3)), 4.07 (s, 1H, NCC***H₂***), 3.64 (hept, $^3J_{H-H}$ = 6.7 Hz, 1H, (C***H***(CH_3)(CH_3)), 3.48 (hept, $^3J_{H-H}$ = 6.8 Hz, 1H, (C***H***(CH_3)(CH_3)), 3.29 (s, 1H, NCC***H₂***), 2.85 (hept, $^3J_{H-H}$ = 6.8 Hz, 1H, (C***H***(CH_3)(CH_3)), 2.84 (hept, $^3J_{H-H}$ = 6.8 Hz, 1H, (C***H***(CH_3)(CH_3)), 2.18 (s, 15H, Cp***Me***), 1.55 (s, 15H, Cp***Me***), 1.49 (s, 3H, NCC***H₃***), 1.45 (d, $^3J_{H-H}$ = 6.7 Hz, 3H, (CH(CH_3)(C***H₃***)), 1.41 (d, $^3J_{H-H}$ = 7.0 Hz, 3H, (CH(CH_3)(C***H₃***)), 1.40 (d, $^3J_{H-H}$ = 6.8 Hz, 3H, (CH(CH_3)(C***H₃***)), 1.39 (d, $^3J_{H-H}$ = 6.9 Hz, 3H, (CH(CH_3)(C***H₃***)), 1.35 (d, $^3J_{H-H}$ = 7.0 Hz, 3H, (CH(CH_3)(C***H₃***)), 1.32 (d, $^3J_{H-H}$ = 6.9 Hz, 3H, (CH(CH_3)(C***H₃***)), 1.30 (d, $^3J_{H-H}$ = 6.7 Hz, 3H, (CH(CH_3)(C***H₃***)), 1.28 (d, $^3J_{H-H}$ = 6.9 Hz, 3H, (CH(CH_3)(C***H₃***)).

^{13}C NMR (C_6D_6, 298 K, 101 MHz) δ: 153.1 (N***C***CH_2), 149.2 (N***C***(CH_3)), 148.4 (Ar-***C***), 148.3 (Ar-***C***), 147.3 (Ar-***C***), 146.3 (Ar-***C***), 142.6 (Ar-***C***), 126.6 (Ar-***C***H), 126.2 (Ar-***C***H), 124.7 (Ar-***C***H), 124.6 (Ar-***C***H), 124.5 (Ar-***C***H), 124.4 (Ar-***C***H), 109.7 (***Cp***Me), 108.6 (***Cp***Me), 105.0 (CH_2C***C***HC(CH_3)), 86.2 (NC***C***H_2), 30.4 (***C***H(CH_3)(CH_3)), 30.3 (***C***H(CH_3)(CH_3)), 29.1 (***C***H(CH_3)(CH_3)), 28.7 (***C***H(CH_3)(CH_3)), 27.5 (CH(CH_3)(***C***H_3)), 26.9 (CH(CH_3)(***C***H_3)), 26.8 (CH(CH_3)(***C***H_3)), 26.1 (CH(CH_3)(***C***H_3)), 25.9 (CH(CH_3)(***C***H_3)), 25.8 (CH(CH_3)(***C***H_3)), 25.6 (CH(CH_3)(***C***H_3)), 25.0 (CH(CH_3)(***C***H_3)), 22.4 (NC(***C***H_3)), 11.4 (Cp***Me***), 10.1 (Cp***Me***).

^{29}Si NMR (C_6D_6, 298 K, 99 MHz) δ: 25.0 (s, Zn-***Si***-Zn).

Anal. Calc. ($C_{49}H_{70}N_2SiZn_2$): C, 69.57; H, 8.34; N, 3.31. Found: C, 69.50; H, 8.64; N, 3.09.

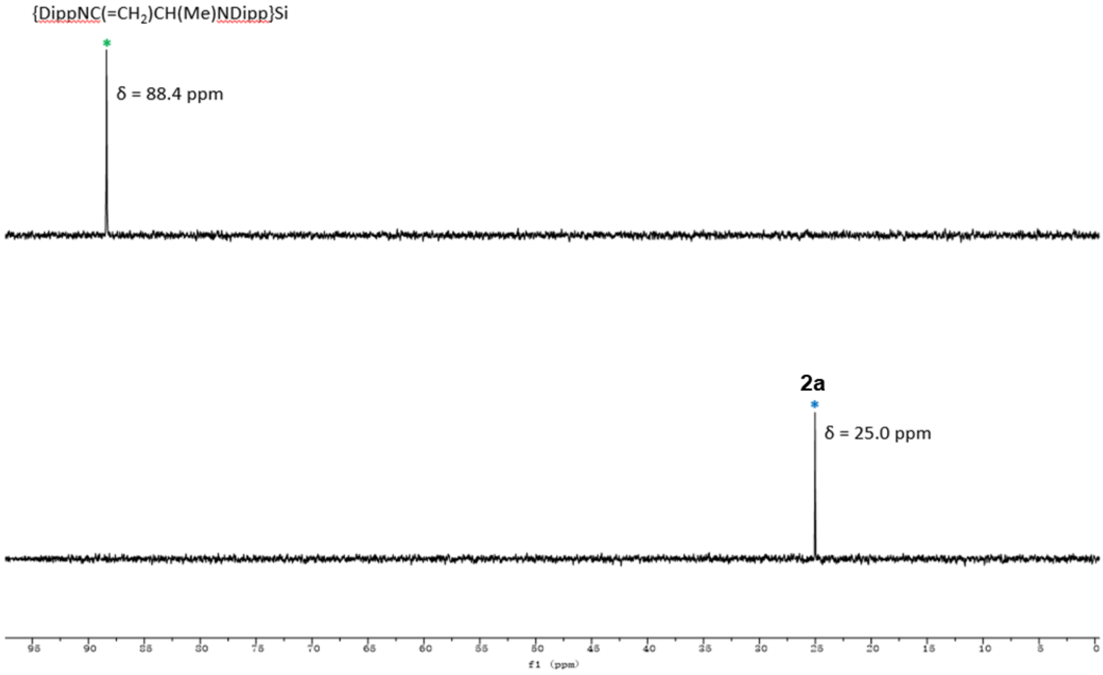

Figure S1. ^{29}Si NMR (C_6D_6, 298 K, 99.4 MHz) "*" is the peak of {DippNC(=CH_2)CH(Me)NDipp}Si, "*" is the peak of **2a**.

Preparation of 2b

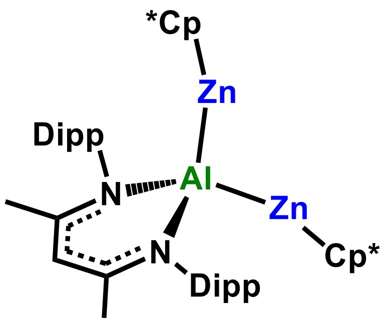

In a glovebox, ^{Dipp}BDIAl (20.0 mg, 0.045 mmol, 1 eq) and Cp*ZnZnCp* (18.0 mg, 0.045 mmol, 1 eq) were dissolved in C_6D_6 (0.6 mL) and transferred to a J. Young NMR tube. The reaction mixture was kept at 25 °C for 10 mins. A ^{1}H NMR spectrum was taken at this time point and showed the full conversion of ^{Dipp}BDIAl to **2b**. The J. Young NMR tube was returned to the glovebox, the solvent was removed under vacuum and the crude reaction mixture dissolved in 1 mL Et_2O/n-pentane (1:1 v: v) mixture. The solution was filtered into a 4 mL vial and then placed in the glovebox freezer (–35 °C) for 2 days. Yellow crystals (**2b**) were successfully obtained. The filtrated crystals were washed with cold n-pentane (3 x 1mL) and then dried in vacuo. **Yield: 17 mg,** 0.020 mmol, 45%.

^{1}H NMR (Tol-d_8, 253 K, 400 MHz) δ: 7.08 – 7.04 (m, 3H, ArC***H***), 7.03 – 6.98 (m, 2H, ArC***H***), 6.94 – 6.92 (m, 1H, ArC***H***), 5.21 (s, 1H, C***H***{C(CH$_3$)}$_2$), 2.97 (hept, $^3J_{H-H}$ = 6.7 Hz, 2H, C***H***(CH$_3$)$_2$), 2.92 (hept, $^3J_{H-H}$ = 6.7 Hz, 2H, C***H***(CH$_3$)$_2$), 2.17 (s, 15H, Cp***Me***), 1.73 (s, 15H, Cp***Me***), 1.48 (s, 6H, 2x NC(C***H$_3$***)), 1.34 (d, $^3J_{H-H}$ = 6.7 Hz, 6H, (CH(C***H$_3$***)$_2$), 1.28 (d, $^3J_{H-H}$ = 6.7 Hz, 6H, (CH(C***H$_3$***)$_2$), 1.27 (d, $^3J_{H-H}$ = 6.7 Hz, 6H, (CH(C***H$_3$***)$_2$), 0.99 (d, $^3J_{H-H}$ = 6.9 Hz, 6H, (CH(C***H$_3$***)$_2$).

^{13}C NMR (Tol-d_8, 253 K, 101 MHz) δ: 171.8 (CH{***C***(CH$_3$)}$_2$), 145.5 (Ar-***C***), 142.3 (Ar-***C***), 142.1 (Ar-***C***), 126.4 (Ar-***C***H), 124.7 (Ar-***C***H), 124.1 (Ar-***C***H), 108.8 (***Cp***Me), 107.8 (***Cp***Me), 98.9 (***C***H{C(CH$_3$)}$_2$), 29.2 (2x ***C***H(CH$_3$)$_2$), 28.8 (2x ***C***H(CH$_3$)$_2$), 26.6 (CH(***C***H$_3$)$_2$), 25.7 (CH(***C***H$_3$)$_2$), 25.2 (CH(***C***H$_3$)$_2$), 24.8 (CH(***C***H$_3$)$_2$), 23.4 (2x CH{C(***C***H$_3$)}$_2$), 11.6 (Cp***Me***), 10.3 (Cp***Me***).

Anal. Calc. ($C_{49}H_{71}N_2AlZn_2$): C, 69.58; H, 8.46; N, 3.31. Found: C, 68.83.; H, 8.04; N, 3.20. Due to the instability of this compound the experiment CHN analysis is an imperfect match for theoretical values.

Preparation of 2c

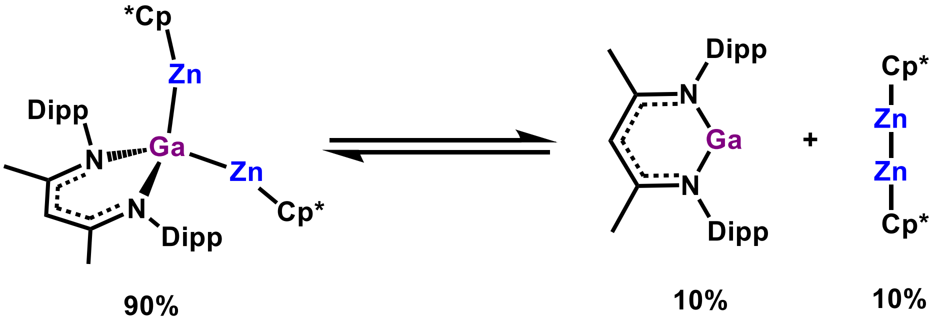

In a glovebox, ^{Dipp}BDIGa (20.0 mg, 0.041 mmol, 1 eq) and Cp*ZnZnCp* (16.5 mg, 0.041 mmol, 1 eq) were dissolved in C_6D_6 (0.6 mL) and transferred to a J. Young NMR tube. The reaction mixture was kept at 40 °C for 5h. A ^{1}H NMR spectrum was taken at this time point and showed 90% conversion of ^{Dipp}BDIGa to **2c**. The J. Young NMR tube was returned to the glovebox, the solvent was removed under vacuum and the crude dissolved in 1 mL Et_2O/n-pentane (1:10 v: v) mixture. The solution was filtered into a 4 mL vial and then placed in the glovebox freezer (–35 °C) for 2 days. Light yellow crystals (**2c**) were successfully obtained. The filtrated crystals were washed with cold n-pentane (3 x 1mL) and then dried in vacuo. Analysis of the pure crystalline material by ^{1}H NMR spectroscopy shows an equilibrium mixture of **2c** and DippBDIGa(I) + Cp*ZnZnCp* suggestive of a reversible process. The ratio is 9 (**2c**): 1 (Ga): 1 (Zn–Zn) at 25 °C. **Yield: 18 mg,** 0.021 mmol, 50%.

^{1}H NMR (C_6D_6, 298 K, 400 MHz) δ: 7.17 – 7.15 (m, 2H, ArC***H***), 7.13 – 7.11 (m, 4H, ArC***H***), 5.16 (s, 1H, C***H***{C(CH$_3$)}$_2$), 3.21 (hept, $^3J_{H\text{-}H}$ = 6.8 Hz, 2H, C***H***(CH$_3$)$_2$), 2.86 (hept, $^3J_{H\text{-}H}$ = 6.8 Hz, 2H, C***H***(CH$_3$)$_2$), 2.22 (s, 15H, Cp***Me***), 1.75 (s, 15H, Cp***Me***), 1.57 (s, 6H, 2x NC(C***H₃***)), 1.38 (d, $^3J_{H\text{-}H}$ = 6.9 Hz, 6H, (CH(C***H₃***)$_2$), 1.36 (d, $^3J_{H\text{-}H}$ = 6.9 Hz, 6H, (CH(C***H₃***)$_2$), 1.30 (d, $^3J_{H\text{-}H}$ = 6.7 Hz, 6H, (CH(C***H₃***)$_2$), 1.12 (d, $^3J_{H\text{-}H}$ = 6.9 Hz, 6H, (CH(C***H₃***)$_2$).

^{13}C NMR (C_6D_6, 298 K, 101 MHz) δ: 170.0 (CH{***C***(CH$_3$)}$_2$), 145.5 (Ar-***C***), 143.9 (Ar-***C***), 142.6 (Ar-***C***), 125.9 (Ar-***C***H), 124.6 (Ar-***C***H), 124.1 (Ar-***C***H), 109.3 (***Cp***Me), 108.1 (***Cp***Me), 97.3 (***C***H{C(CH$_3$)}$_2$), 29.3 (2x ***C***H(CH$_3$)$_2$), 28.7 (2x ***C***H(CH$_3$)$_2$), 26.9 (CH(***C***H$_3$)$_2$), 26.0 (CH(***C***H$_3$)$_2$), 25.3 (CH(***C***H$_3$)$_2$), 25.0 (CH(***C***H$_3$)$_2$), 23.4 (2x CH{C(***C***H$_3$)}$_2$), 11.5 (Cp***Me***), 10.2 (Cp***Me***).

Anal. Calc. ($C_{49}H_{71}N_2GaZn_2$): C, 66.23; H, 8.05; N, 3.15. Found: C, 65.22; H, 8.22; N, 3.01.

VT-NMR of 2c (193K – 333K)

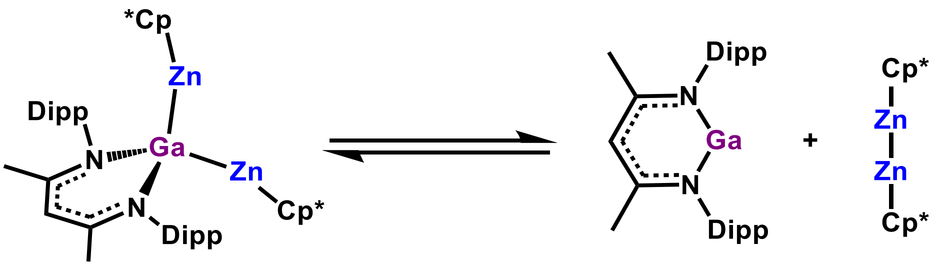

90% **10%** **10%**

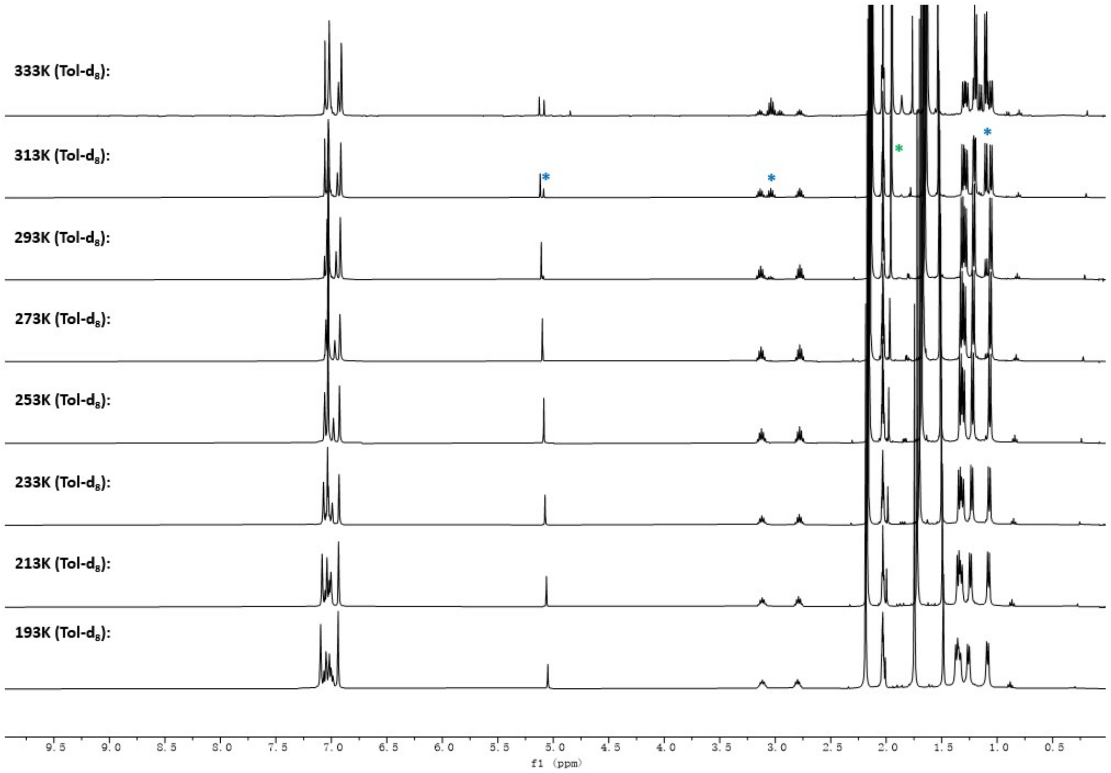

Figure S2. ^{1}H NMR (Tol-d_8, 400 MHz) of **2c**: ("*" is a selected resonance of**1c,** "*"is a selected resonance of **Cp*ZnZnCp***). The equilibrium shifts toward **2c** at lower temperatures.

Preparation of 3

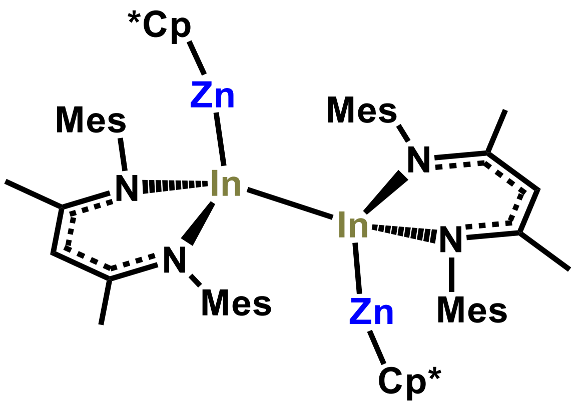

In a glove box, Cp*ZnZnCp* (9.0 mg, 0.025 mmol, 1 eq) and ^{Mes}BDIIn (20.0 mg, 0.045 mmol, 2 eq) were dissolved in 1 mL of n-pentane and 1 m THF respectively. The n-pentane solution of Cp*ZnZnCp* was then added dropwise to the THF solution of ^{Mes}BDIIn and the reaction mixture stirred for 3 mins. The formation of bright yellow precipitate could be clearly observed at thie time. The reaction mixture was filtered, the bright yellow precipitate was collected by filtration and washed with 2×1mL n-pentane, to give compound **3**. **Yield: 17 mg,** 0.013 mmol, 60%. Compound **3** is highly insoluble in common laboratory solvents (Such as: THF, Et_2O, cyclohexane, toluene) precluding the collection of multinuclear NMR spectroscopic data. In addition, compound **3** is very susceptible to decomposition at room temperature in aromatic solvents. Crystals were obtained from very dilute solutions of **3** in toluene at -35 °C in the glovebox freezer.

Anal. Calc. ($C_{66}H_{88}N_4In_2Zn_2$): C, 61.08; H, 6.83; N, 4.32. Found: C, 61.24; H, 6.75; N, 4.14.

Additional Reactions

A series of reactions were conducted between Mg–Mg and Zn–Zn bonded compounds bearing β-diketiminate ligands, including (^{Mes}BDIMg)$_2$ (^{Mes}BDI = {MesNC(Me)}$_2$CH), (^{Dipp}BDIMg)$_2$ and (^{Mes}BDIZn)$_2$, with **1a-c** under both thermal or photochemical conditions. Photolysis experiments were carried out using a Spectral Energy mercury arc lamp (400 W) or Kessil lamps (40 W, 390 nm) with samples contained in a J. Young's NMR tubes. In no cases did these reactions lead to the formation of single products, forming either complex mixtures or unreacted starting materials. All attempts to isolate pure crystalline compounds from product mixtures failed. The findings are summarised below.

	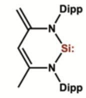	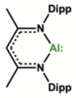	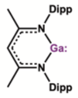	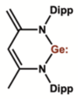
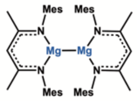 **S1a**	60 °C, 16 h, no reaction	60 °C, 16 h, no reaction	60 °C, 16 h, no reaction	N/A
	UV photolysis (Hg lamp), 6 h **S1a** decomposed	UV photolysis (Hg lamp), 6 h **S1a** decomposed	UV photolysis (Hg lamp), 6 h **S1a** decomposed	
	390nm LED, 6 h **S1a** decomposed	390nm LED, 6 h **1b** and **S1a** decomposed	390nm LED, 6 h **S1a** decomposed	
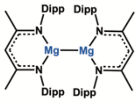 **S1b**	60 °C, 16 h, no reaction	60 °C, 16 h, no reaction	60 °C, 16 h, no reaction	N/A
	UV photolysis (Hg lamp), 6 h no reaction	UV photolysis (Hg lamp), 6 h no reaction	UV photolysis (Hg lamp), 6 h no reaction	
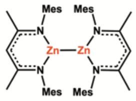 **S2a**	60 °C, 16 h, no reaction	60 °C, 16 h, no reaction	60 °C, 16 h, no reaction	60 °C, 16 h, no reaction
	UV photolysis (Hg lamp), 6 h formation of complex mixture	UV photolysis (Hg lamp), 6 h formation of complex mixture	UV photolysis (Hg lamp), 6 h formation of complex mixture	
	390nm LED, 6 h **1a** decomposed	390nm LED, 6 h **1b** decomposed	390nm LED, 6 h **1c** decomposed	
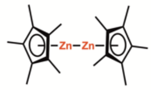	**2a**	**2b**	**2c**	60 °C, 16 h, no reaction

Table S1. Summary of outcomes of reactions of group 13 and 14 carbene analogues with Zn–Zn and Mg–Mg bonds. N/A are reactions not attempted.

Attempts to adjust the stoichiometry of the reaction of **1d** with Cp*ZnZnCp*, or change the sterics of the ligand on indium led to ligand exchange processes in which the ligand on In and Zn switched sites.

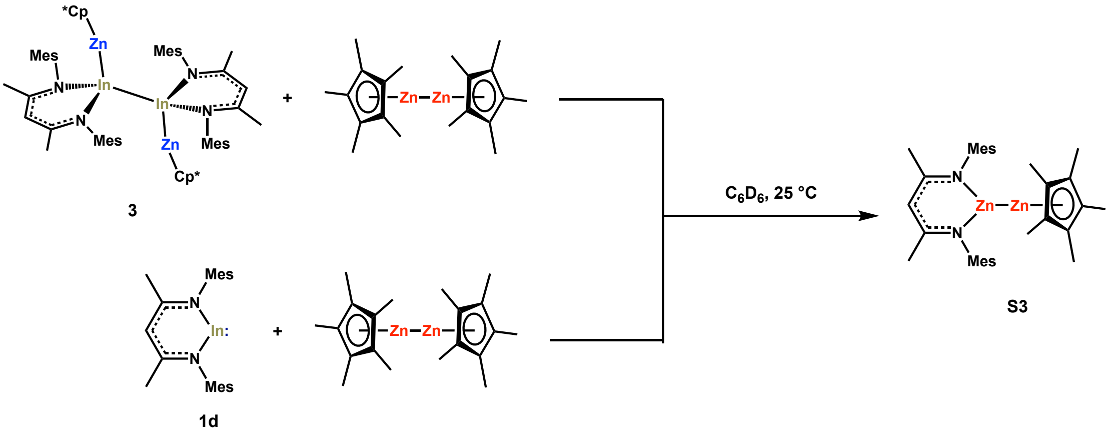

Method 1: In a glovebox, **3** (20.0 mg, 0.016 mmol, 1 eq) and Cp*ZnZnCp* (6.2 mg, 0.016 mmol, 1 eq) were dissolved in C_6D_6 (0.6 mL) and transferred to a J. Young NMR tube. The reaction mixture was kept at 25 °C for 10 mins. A ^{1}H NMR spectrum was taken at this time point and showed that all the **3** has been reacted and product **S3** was found. Product **S3** can be successfully extracted by 1ml pentane. The solution was filtered into a 4 mL vial and then placed in the glovebox freezer (–35 °C) for 2 days. Colourless crystals (**S3**) were successfully obtained. **Yield: 4.6 mg,** 0.008 mmol, 50%.

Method Two: In a glovebox, **1d** (10.0 mg, 0.022 mmol, 1 eq) and Cp*ZnZnCp* (9.0 mg, 0.022 mmol, 1 eq) were dissolved in C_6D_6 (0.6 mL) and transferred to a J. Young NMR tube. The reaction mixture was kept at 25 °C for 30 mins. A ^{1}H NMR spectrum was taken at this time point and showed that all the **1d** has been reacted and product **S3** was found.

^{1}H NMR (C_6D_6, 298 K, 400 MHz) δ: 6.83 (s, 1H, ArC***H***), 4.93 (s, 1H, C***H***{C(CH$_3$)}$_2$), 2.20 (s, 6H, 2x NC(C***H$_3$***)), 2.07 (s, 12H, *o*-Ar***H***), 1.91 (s, 15H, Cp***Me***), 1.60 (s, 6H, *p*-Ar***H***). **^{13}C NMR** (C_6D_6, 298 K, 101 MHz) δ: 166.1 (CH{***C***(CH$_3$)}$_2$), 145.7 (Ar-***C***), 133.5 (Ar-***C***), 129.2 (Ar-***C***H), 108.3 (***Cp***Me), 96.1 (***C***H{C(CH$_3$)}$_2$), 22.8 (NC(***C***H$_3$)), 20.9 (Ar***C***H$_3$), 19.1 (*o*-Ar***C***H$_3$), 10.2 (*p*-Ar***C***H$_3$).

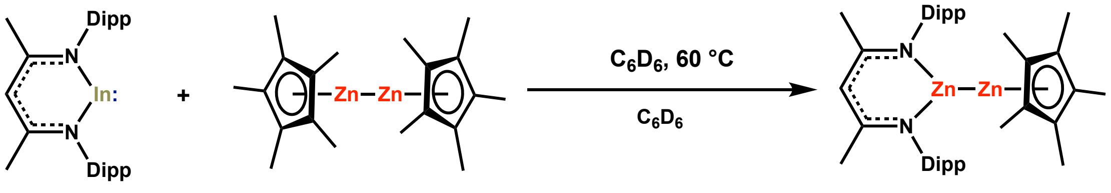

1d'

S4

In a glovebox, **1d’** (10.0 mg, 0.019 mmol, 1 eq) and Cp*ZnZnCp* (7.5 mg, 0.019 mmol, 1 eq) were dissolved in C_6D_6 (0.6 mL) and transferred to a J. Young NMR tube. The reaction mixture was kept at 25 °C for 1 h and no reaction occurred. The reaction mixture was then kept at 60 °C for 6 h. A ^{1}H NMR spectrum was taken at this time point and showed that all the **1d’** has been reacted and product **S4**[S13] was obtained

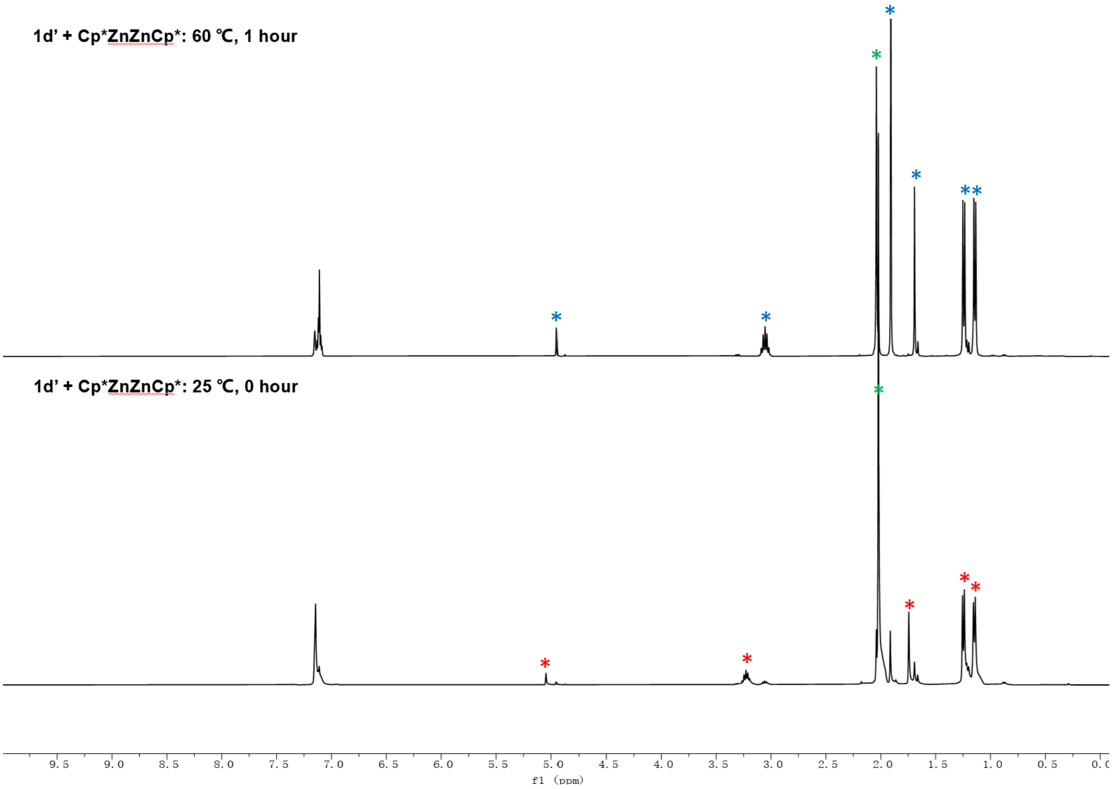

Figure S3. Crude ^{1}H NMR (C_6D_6, 298 K, 400 MHz) “*” is the peak of **S4**, “*” is the peak of Cp*ZnZnCp*, “*” is the peak of **1d’**.

We also investigated additional reactions with some transition metal precursors. We would note that aspects of this work have already been considered and published by us [S14-16] and Fischer and coworkers.[S17-18] Nevertheless, we reinvestigated these reactions, including under photolytic conditions, with no clear cut identification of products related to **2a-c** identified. The results are summarised below.

	**[Ni(COD)$_2$]**	**(Cy$_3$P)$_2$Ni-N$_2$-Ni(PCy$_3$)$_2$**	**[Pd(PCy$_3$)$_2$]**
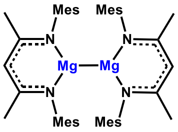 **S1a**	60 °C, 16 h, **S1a** decomposed	Reference S14	Reference S15
	UV photolysis (Hg lamp), 6 h **S1a** decomposed	UV photolysis (Hg lamp), 6 h **S1a** decomposed	UV photolysis (Hg lamp), 6 h **S1a** decomposed
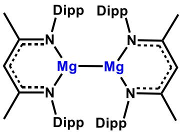 **S1b**	60 °C, 16 h, no reaction	60 °C, 16 h, no reaction	60 °C, 16 h, no reaction
	UV photolysis (Hg lamp), 6 h no reaction	UV photolysis (Hg lamp), 6 h no reaction	UV photolysis (Hg lamp), 6 h no reaction
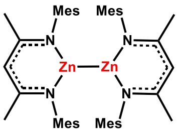 **S2a**	60 °C, 16 h, no reaction	60 °C, 16 h, no reaction	60 °C, 16 h, no reaction
	UV photolysis (Hg lamp), 6 h no reaction	UV photolysis (Hg lamp), 6 h no reaction	UV photolysis (Hg lamp), 6 h no reaction

Table S2. Summary of outcomes of reactions of selected Ni and Pd complexes with Zn–Zn and Mg–Mg bonds under both thermal and photochemical conditions.

3) Single Crystal X-ray Diffraction Data

Table S3. Crystal Data, Data Collection and Refinement Parameters for the structures of **2a**, **2b**, **2c** and **3**.

data	2a	2b	2c	3
**formula**	$C_{49}H_{70}N_2SiZn_2$	$C_{49}H_{71}AlN_2Zn_2$	$C_{49}H_{71}GaN_2Zn_2$	$C_{66}H_{88}In_2N_4Zn_2$
**solvent**	—	$C_4H_{10}O$	1.75($C_4H_{10}O$)	C_7H_8
**formula weight**	845.90	919.91	1018.24	1389.91
**colour, habit**	colourless	yellow blocks	yellow tablets	yellow blocks
**temperature / K**	173	173	173	173
**crystal system**	monoclinic	monoclinic	trigonal	monoclinic
**space group**	$P2_1/n$ (no. 14)	$P2_1/n$ (no. 14)	$R3m$ (no. 160)	$C2/c$ (no. 15)
***a* / Å**	11.83370(17)	11.07416(14)	37.6593(3)	22.8976(2)
***b* / Å**	31.5359(4)	12.96858(16)	37.6593(3)	19.6820(2)
***c* / Å**	12.51969(18)	36.2851(5)	10.88658(11)	15.26125(14)
**α / deg**	90	90	90	90
**β / deg**	90.6535(13)	96.4551(12)	90	92.6250(9)
**γ / deg**	90	90	120	90
**V / Å^3**	4671.87(11)	5178.09(11)	13371.1(2)	6870.59(12)
Z	4	4	9 [c]	4 [d]
**D_c / g cm^{-3}**	1.203	1.180	1.138	1.344
**radiation used**	Cu-Kα	Cu-Kα	Cu-Kα	Cu-Kα
**μ / mm^{-1}**	1.742	1.566	1.733	6.398
**no. of unique reflns**				
**measured (R_{int})**	9389 (0.0611)	13848	6149 (0.0235)	6800 (0.0311)
**obs, $\|F_o\| > 4\sigma(\|F_o\|)$**	7611	11659	5944	5941
**completeness (%)** [a]	100	100	99.9	99.9
**no. of variables**	528	606	327	380
**R_1(obs), wR_2(all)** [b]	0.0405, 0.1091	0.0507,	0.0229,	0.0279,
**CCDC code**	2350790	2350788	2350789	2410821

[a] Completeness to 0.84 Å resolution. [b] $R_1 = \Sigma||F_o| - |F_c||/\Sigma|F_o|$; $wR_2 = \{\Sigma[w(F_o^2 - F_c^2)^2] / \Sigma[w(F_o^2)^2]\}^{1/2}$; $w^{-1} = \sigma^2(F_o^2) + (aP)^2 + bP$. [c] The complex has crystallographic C_S symmetry. [d] The complex has crystallographic C_i symmetry.

Table S3 provides a summary of the crystallographic data for the structures of **2a**, **2b**, **2c** and **3**. Data were collected using an Agilent Xcalibur PX Ultra A diffractometer, and the structures were solved and refined using the OLEX2,[S19] SHELXTL[S20] and SHELX-2013[S21] program systems. The absolute structure of **2c** was determined by use of the Flack parameter [x = –0.023(11)]. CCDC 2350788 to 2350790 (**2a** to **2c**), and CCDC 2410821 (**3**).

The X-ray crystal structure of 2a

The C12- and C27-based isopropyl groups in the structure of **2a** were found to be disordered, and two orientations were identified of *ca*. 59:41 occupancy in each case. The geometries of each pair of orientations were optimised, the thermal parameters of adjacent atoms were restrained to be similar, and only the non-hydrogen atoms of the major occupancy orientations were refined anisotropically (those of the minor occupancy orientations were refined isotropically).

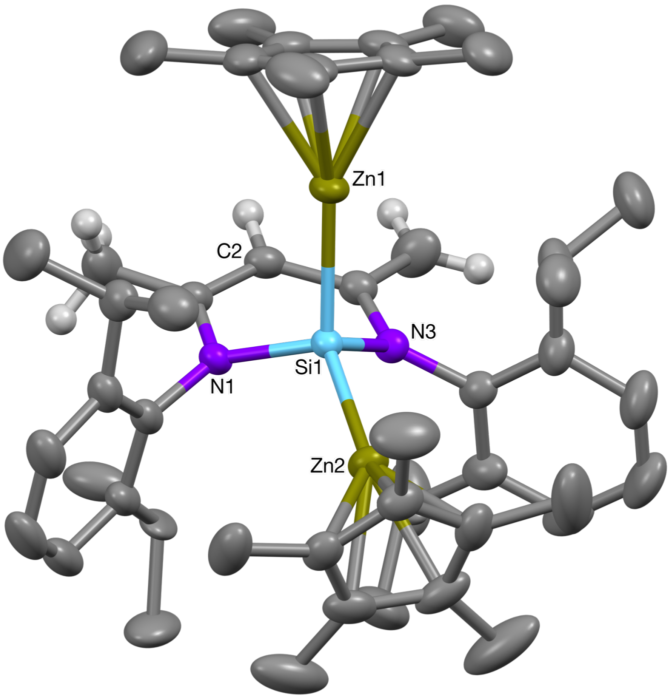

Figure S4. The crystal structure of **2a** (50% probability ellipsoids).

The X-ray crystal structure of 2b

The crystal of **2b** that was studied was found to be a two component twin in a *ca*. 63:37 ratio, with the two lattices related by the approximate twin law [1.00 0.00 0.00 0.00 –1.00 0.00 –0.74 0.00 –1.00]. The C6- and C18-based diisopropylphenyl rings, the C30-based Cp* ring, and the O50-based included diethylether solvent molecule were all found to be disordered, and two orientations were identified in each case of *ca*. 56:44, 60:40, 77:23 and 67:33 occupancy respectively. The geometries of each pair of orientations were optimised, the thermal parameters of adjacent atoms were restrained to be similar, and only the non-hydrogen atoms of the major occupancy orientations were refined anisotropically (those of the minor occupancy orientations were refined isotropically).

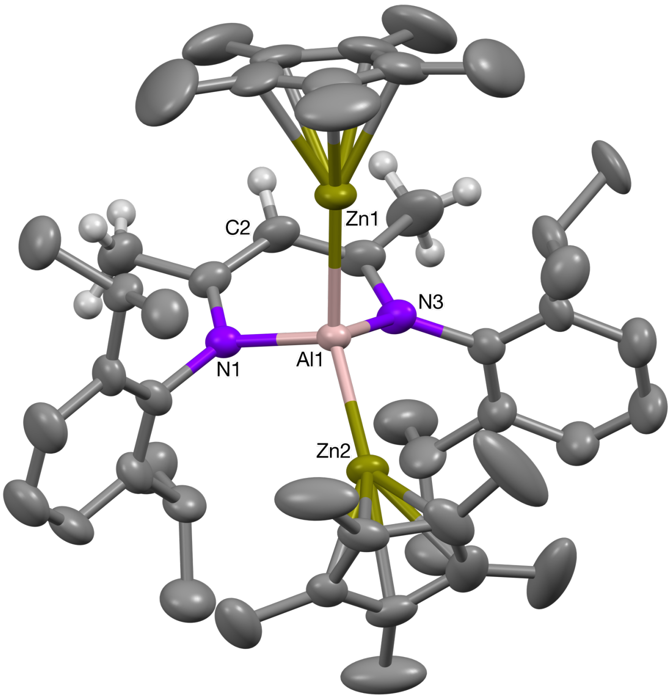

Figure S5. The crystal structure of **2b** (50% probability ellipsoids).

The X-ray crystal structure of 2c

The structure of **2c** was found to sit across a mirror plane that passes through Ga1, Zn1, Zn2 and C2. The C20- and C30-based Cp* rings were both found to be disordered across this mirror plane, and in each case this was modelled using one complete, 50% occupancy, orientation (with a second 50% occupancy orientation being generated by operation of the mirror plane). The geometry of each unique orientation was optimised and all of the non-hydrogen atoms were refined anisotropically. The included solvent was found to be highly disordered, and the best approach to handling this diffuse electron density was found to be the SQUEEZE routine of PLATON.[S22] This suggested a total of 641 electrons per unit cell, equivalent to 71.2 electrons per complex. Before the use of SQUEEZE the solvent equally resembled both of the possibilities (diethylether, $C_4H_{10}O$, 42 electrons, and pentane, C_5H_{12}, 42 electrons), and so the most recently used solvent was assumed, and as 1.75 diethylether molecules corresponds to 73.5 electrons this was used as the solvent present. As a result, the atom list for the asymmetric unit is low by 1.75/2($C_4H_{10}O$) = $C_{3.5}H_{8.75}O_{0.875}$ (and that for the unit cell low by $C_{63}H_{157.5}O_{15.75}$) compared to what is actually presumed to be present. The absolute structure of **2c** was determined by use of the Flack parameter [x = –0.023(11)].

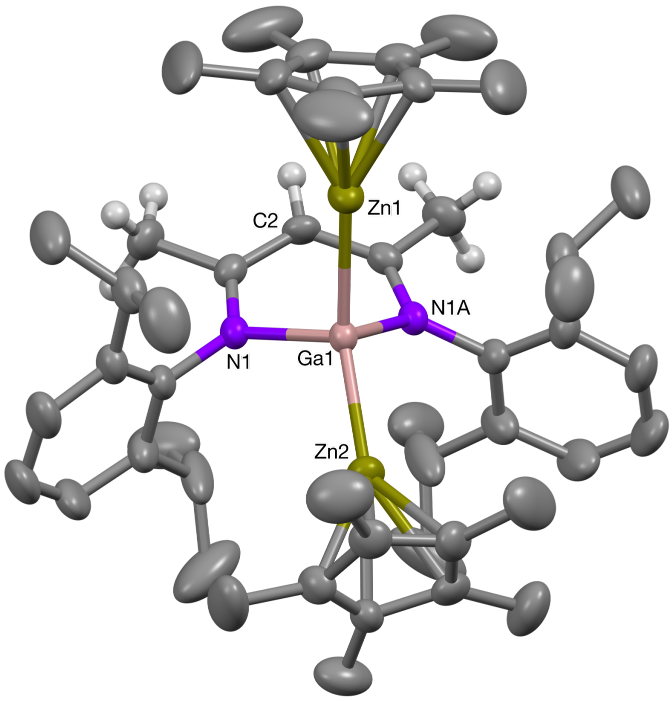

Figure S6. The crystal structure of **2c** (50% probability ellipsoids).

The X-ray crystal structure of 3

The structure of **3** was found to sit across a centre of symmetry at the middle of the In1–In1A bond. The C50-based included toluene solvent molecule was found to be disordered across a centre of symmetry, and two unique orientations were identified of *ca*. 26 and 24% occupancy (with two further orientations of the same occupancies being generated by operation of the inversion centre). The geometries of the two unique orientations were optimised, the thermal parameters of adjacent atoms were restrained to be similar, and all of the atoms were refined isotropically.

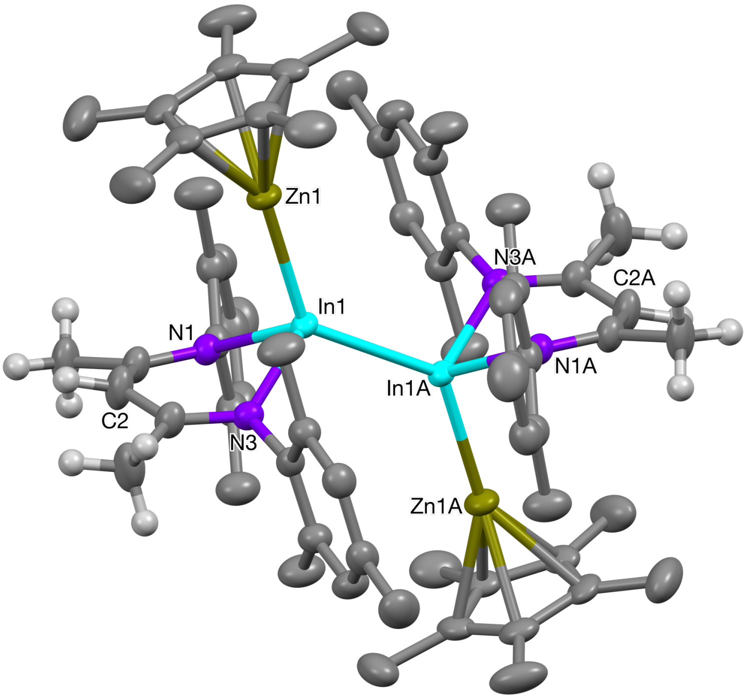

Figure S7. The crystal structure of the *Ci*-symmetric complex **3** (50% probability ellipsoids).

4) Reactivity of 2a-c

4.1 Reaction of 2b with MeI

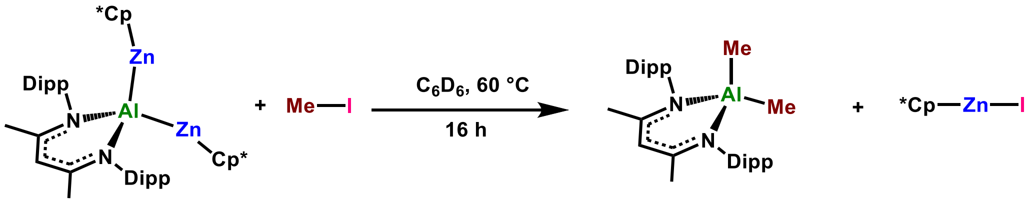

Procedure: In a glovebox, **2b** (10.0 mg, 0.012 mmol, 1 eq) and methyl iodide (5.0 mg, 0.035 mmol, 3 eq) were dissolved in C_6D_6 (0.6 mL) and transferred to a J. Young NMR tube. The reaction mixture was kept at 60 °C for 16 hours. A ^{1}H NMR spectrum was taken at this time point and showed all **2b** reacted. The NMR spectra were showed in Figure S8 and show the formation of ^{Dipp}BDIAlMe$_2$, Cp*ZnI, along with small amounts of Cp*ZnMe.

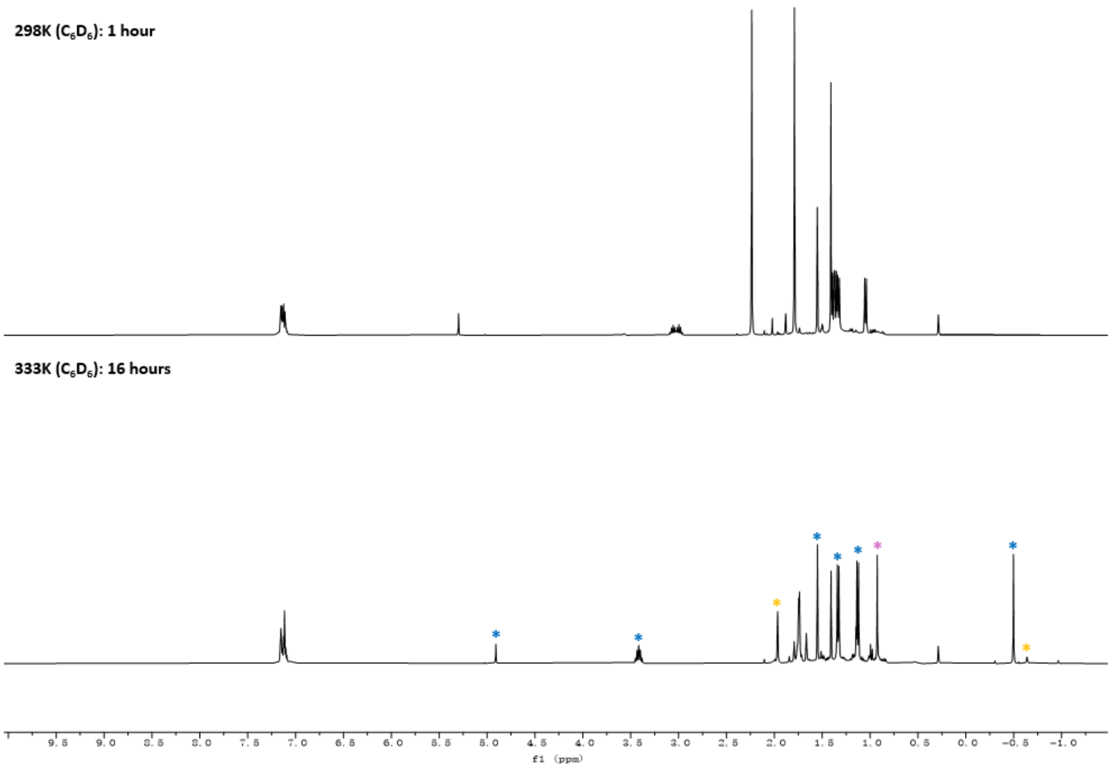

Figure S8. Crude ^{1}H NMR (C_6D_6, 298 K, 400 MHz) "*" is the peak of ^{Dipp}BDIAlMe$_2$, "*" is the peak of Cp*ZnI, "*" is the peak of Cp*ZnMe.

4.1.1 Control reaction between ^{Dipp}BDIAl and MeI

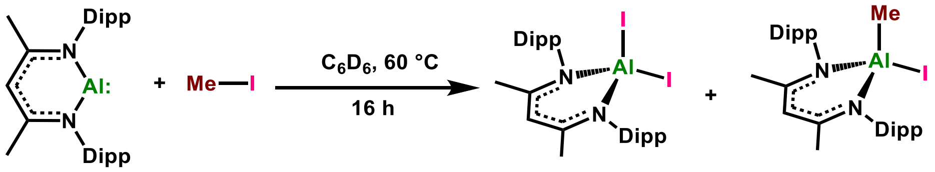

In a glovebox, ^{Dipp}BDIAl (5.0 mg, 0.011 mmol, 1 eq) and methyl iodide (4.8 mg, 0.034 mmol, 3 eq) were dissolved in C_6D_6 (0.6 mL) and transferred to a J. Young NMR tube. The reaction mixture was kept at 60 °C for 16 hours. A ^{1}H NMR spectrum was taken at this time point and showed complete consumption of ^{Dipp}BDIAl. The NMR spectra are presented in Figure S9 and show the formation of a mixture of ^{Dipp}BDIAlI$_2$ and ^{Dipp}BDIAl(Me)I.

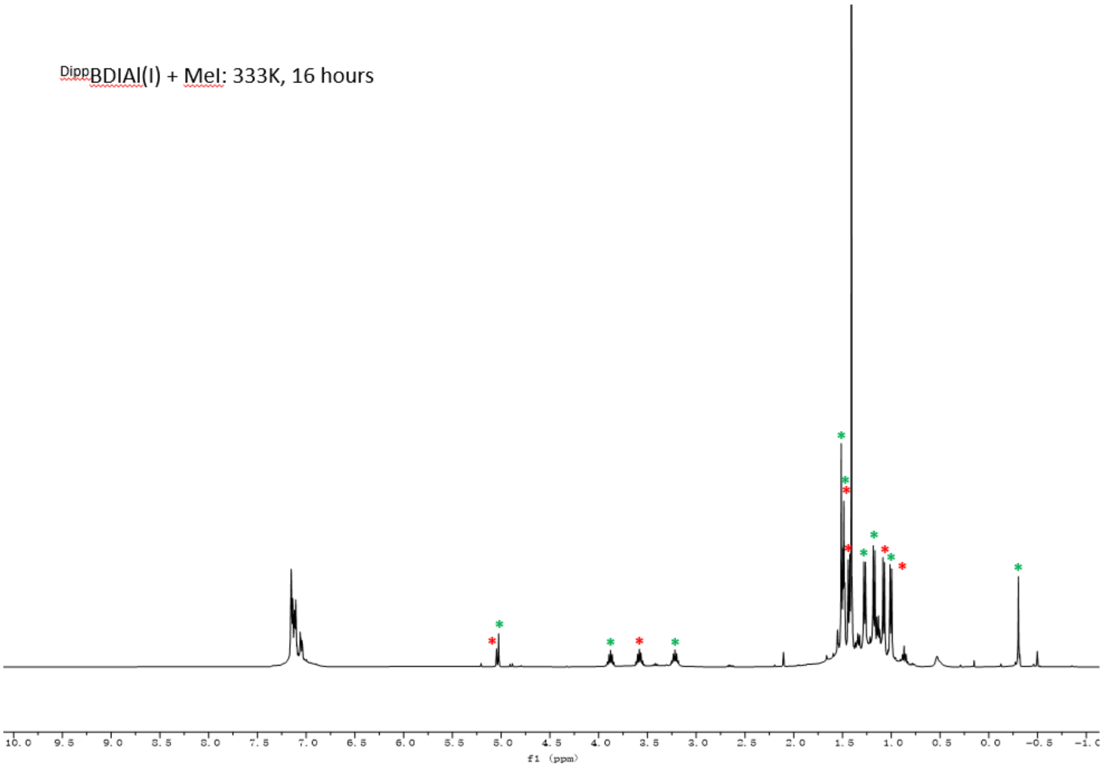

Figure S9. Crude ^{1}H NMR (C_6D_6, 298 K, 400 MHz) "*" is the peak of ^{Dipp}BDIAlMeI, "*" is the peak of ^{Dipp}BDIAlI$_2$.

4.1.2 Control reaction of Cp*ZnZnCp* with MeI and Cp*ZnMe with ^{Dipp}BDIAl

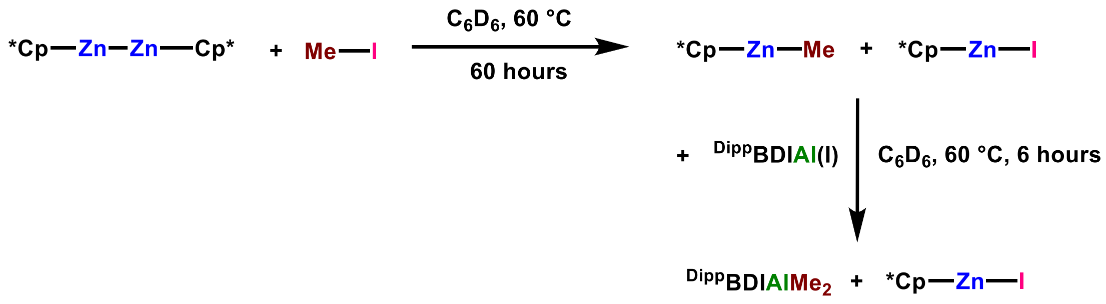

Step One: In a glovebox, Cp*ZnZnCp* (5.0 mg, 0.012 mmol, 1 eq) and methyl iodide (5.3 mg, 0.037 mmol, 3 eq) were dissolved in C_6D_6 (0.6 mL) and transferred to a J. Young NMR tube. The reaction mixture was kept at 60 °C for 60 hours. A ^{1}H NMR spectrum was taken at this time point and showed all Cp*ZnZnCp* reacted. The ^{1}H NMR spectra were showed in Figure S10 which record the whole process to form Cp*ZnMe and Cp*ZnI.

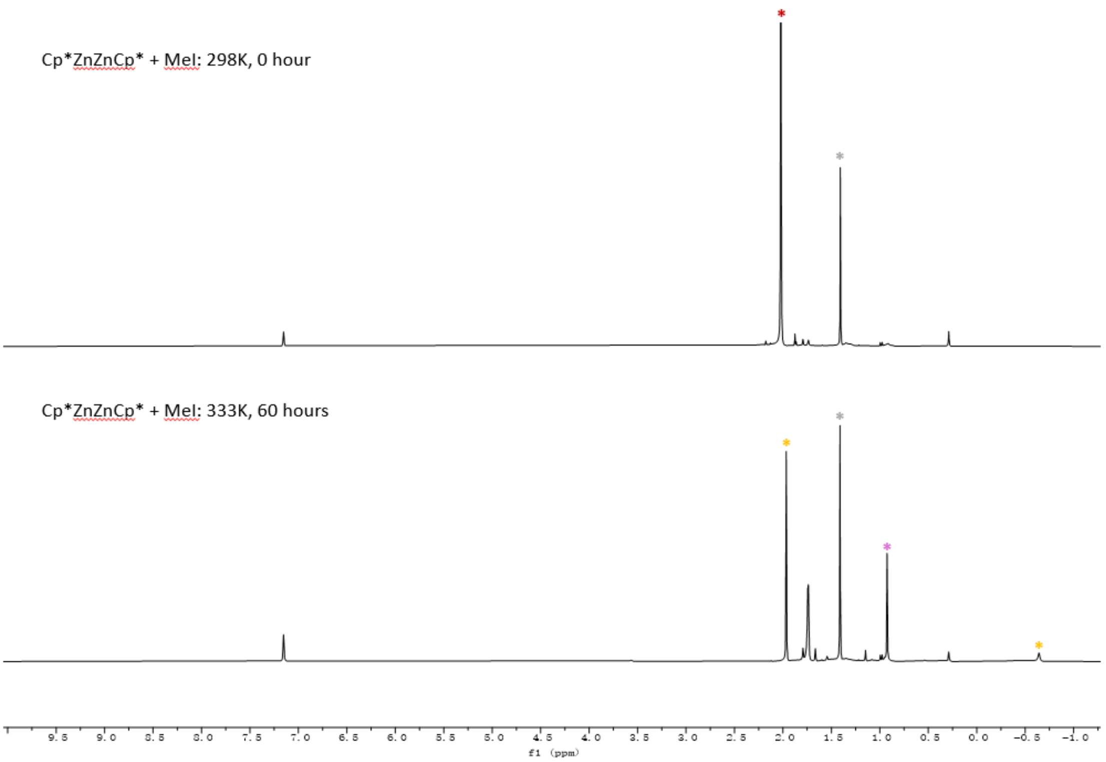

Figure S10. ^{1}H NMR (C_6D_6, 298 K, 400 MHz) "*" is the peak of Cp*ZnMe, "*" is the peak of Cp*ZnI, "*" is the peak of Cp*ZnZnCp*, "*" is the peak of MeI.

Step Two: In a glovebox, ^{Dipp}BDIAl(I) (2.8 mg, 0.007 mmol, 0.5 eq) was dissolved in C_6D_6 (0.2 mL) and transferred to the raw reaction of step one. The reaction mixture was kept at 60 °C for 6 hours. A ^{1}H NMR spectrum was taken at this time point and showed all ^{Dipp}BDIAl(I) reacted. The ^{1}H NMR spectra showed in Figure S11 which record the whole process to form ^{Dipp}BDIAlMe$_2$ and original Cp*ZnI which didn't join to the step two reaction.

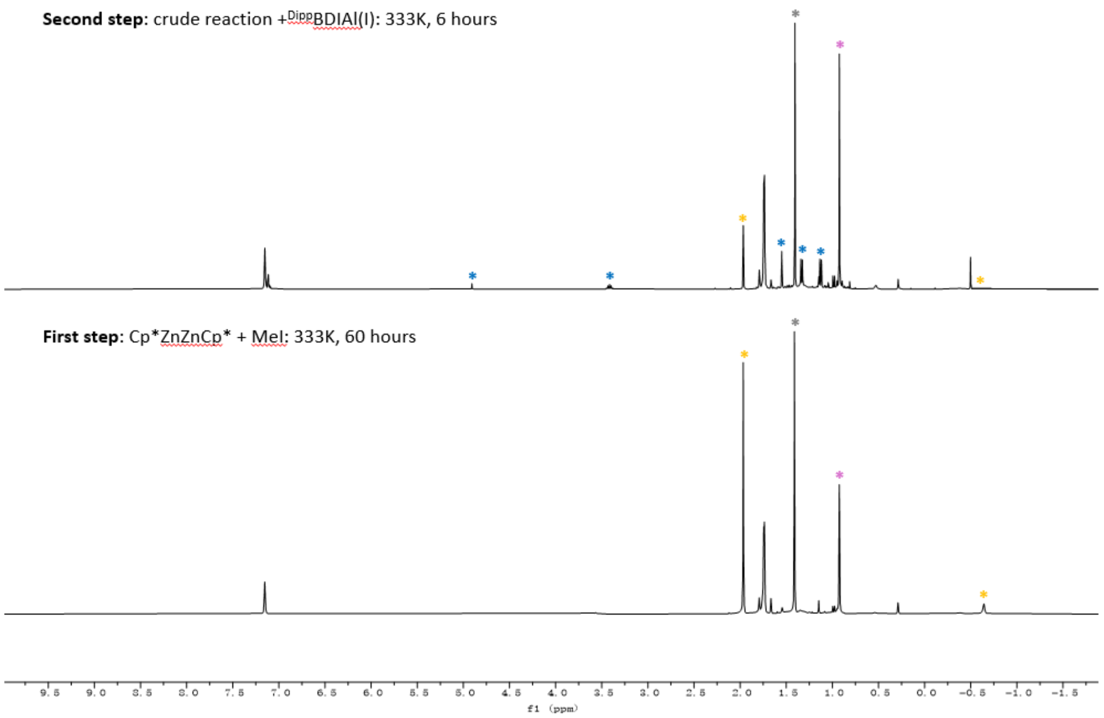

Figure S11. ^{1}H NMR (C_6D_6, 298 K, 400 MHz) "*" is the peak of ^{Dipp}BDIAlMe$_2$, "*" is the peak of Cp*ZnMe, "*" is the peak of Cp*ZnI, "*" is the peak of MeI.

4.1.3 Control reaction of Cp*ZnZnCp* with MeI and Cp*ZnMe with ^{Dipp}BDIAl

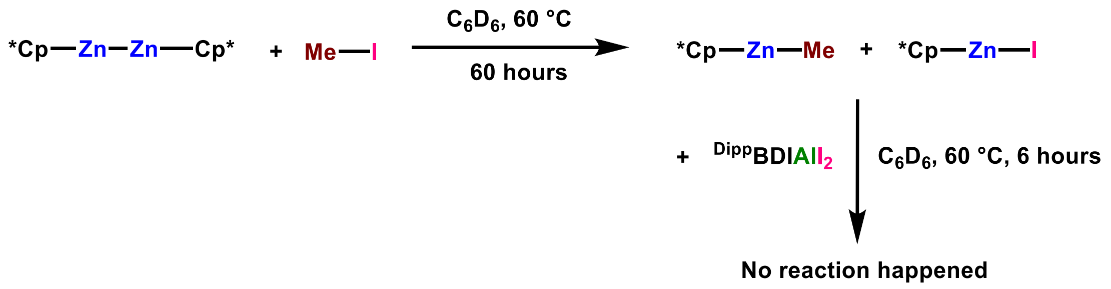

Step One: In a glovebox, Cp*ZnZnCp* (5.0 mg, 0.012 mmol, 1 eq) and Methyl iodide (5.3 mg, 0.037 mmol, 3 eq) were dissolved in C_6D_6 (0.6 mL) and transferred to a J. Young NMR tube. The reaction mixture was kept at 60 °C for 60 hours. A ^{1}H NMR spectrum was taken at this time point and showed all Cp*ZnZnCp* reacted. The ^{1}H NMR spectra were showed like Figure S10 which record the whole process to form Cp*ZnMe and Cp*ZnI.

Step Two: In a glovebox, ^{Dipp}BDIAlI$_2$ (4.4 mg, 0.006 mmol, 0.5 eq) was dissolved in C_6D_6 (0.2 mL) and transferred to the raw reaction of step one. The reaction mixture was kept at 60 °C for 6 hours. A ^{1}H NMR spectrum was taken at this time point and showed that no reaction happened.

4.1.4 Control reaction between ^{Dipp}BDIAlMe$_2$ and ^{Dipp}BDIAlI$_2$

In a glovebox, ^{Dipp}BDIAlMe$_2$ (5.0 mg, 0.011 mmol, 1 eq) and ^{Dipp}BDIAlI$_2$ (6.4 mg, 0.011 mmol, 1 eq) was dissolved in C_6D_6 (0.6 mL) and transferred to a J. Young NMR tube. The reaction mixture was kept at 60 °C for 60 hours and monitored by ^{1}H NMR spectroscopy. No reaction occurred.

4.2 Cross-over experiment

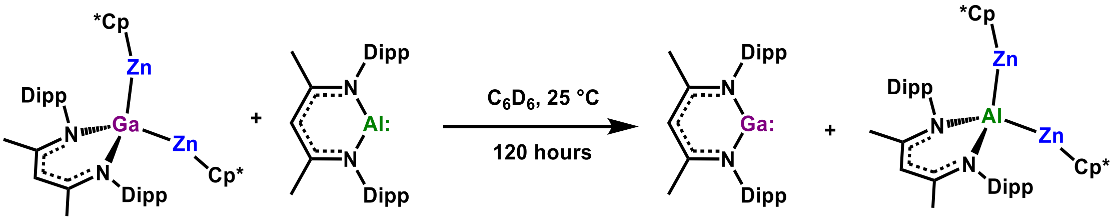

Procedure: In a glovebox, **2c** (10.0 mg, 0.011 mmol, 1 eq) and ^{Dipp}BDIAl (5.0 mg, 0.011 mmol, 1 eq) were dissolved in d_8-Toluene (0.6 mL) and transferred to a J. Young NMR tube. The reaction mixture was kept at 25 °C for 120 hours. A ^{1}H NMR spectrum was taken at this time point and showed the full conversion of **2c** to **2b**. The NMR spectra are presented in Figure S12.

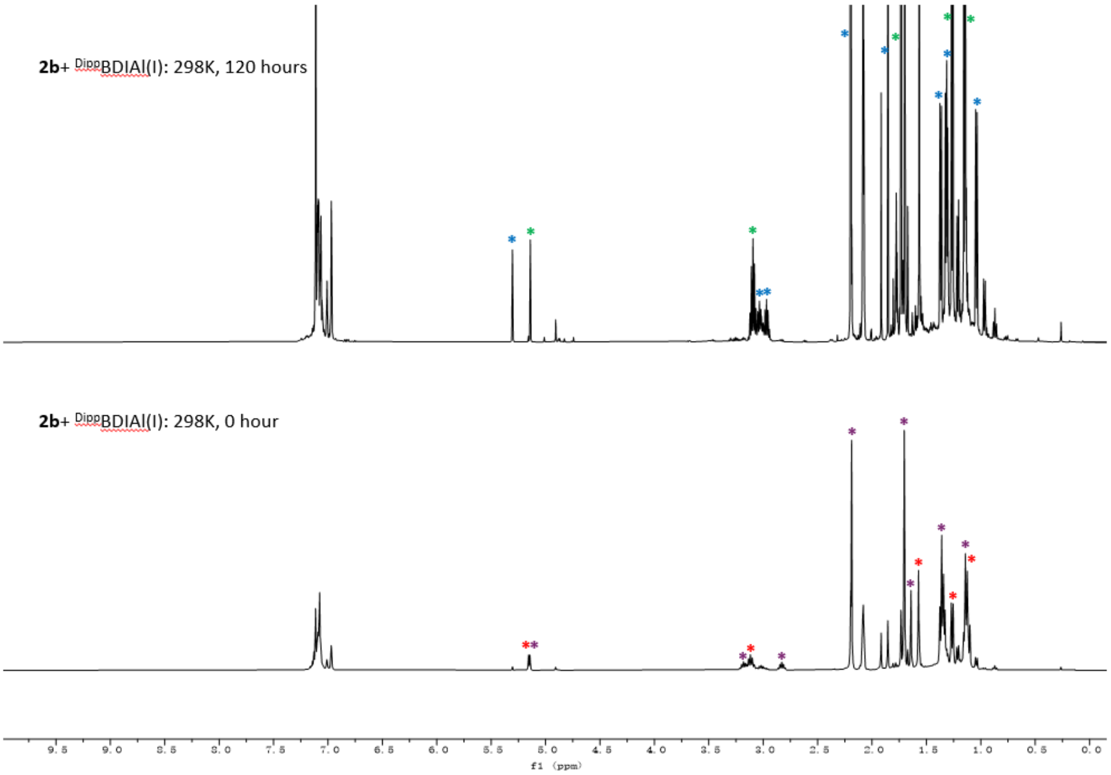

Figure S12. ^{1}H NMR (C_6D_6, 298 K, 400 MHz) for cross-over experiment. "*" is the peak of **2b**, "*" is the peak of ^{Dipp}BDIGa(I), "*" is the peak of ^{Dipp}BDIAl(I), "*" is the peak of **2c**.

5) Van't Hoff analysis

Van't Hoff analysis of **2c** (303 – 343K) was monitored in situ in a Bruker 400 MHz machine. The testing concentration of **2c** is 0.0205 M. (Capillary: ferrocene in C_6D_6, concentration: 0.007 M)

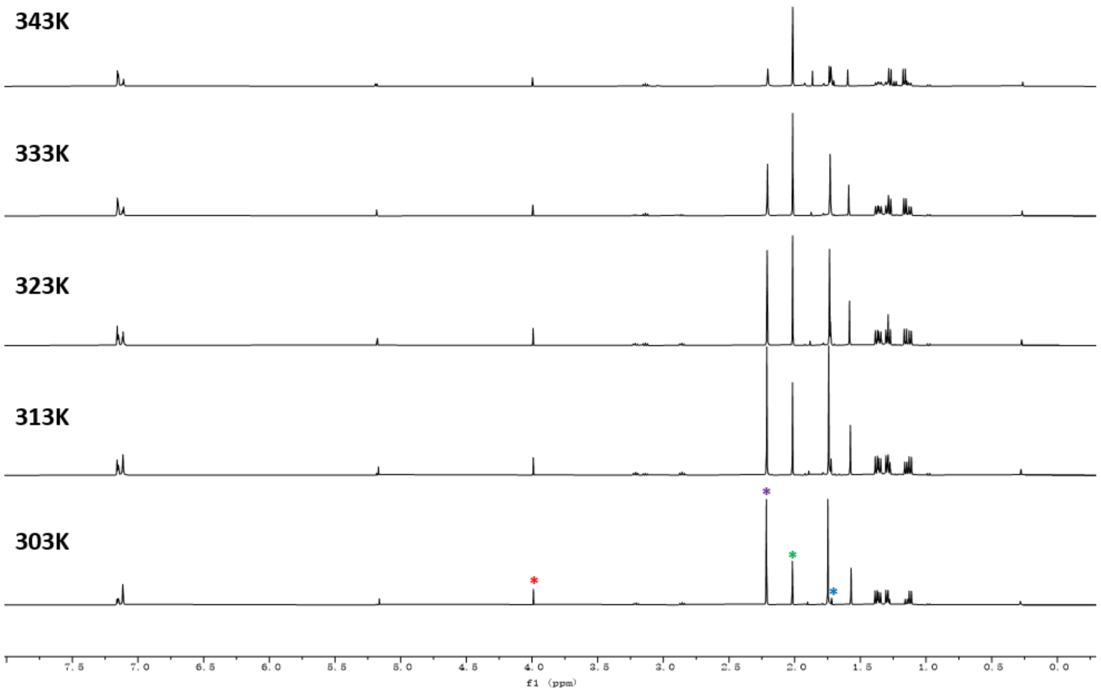

Figure S13. ^{1}H NMR spectroscopic data for the equilibrium between **1c** + **Cp*ZnZnCp*** and **2c** in C_6D_6. ("*" is a selected resonance of **1c,** "*"is a selected resonance of Cp*ZnZnCp*, "*"is a selected resonance of **2c**, "*" is the resonance of ferrocene).

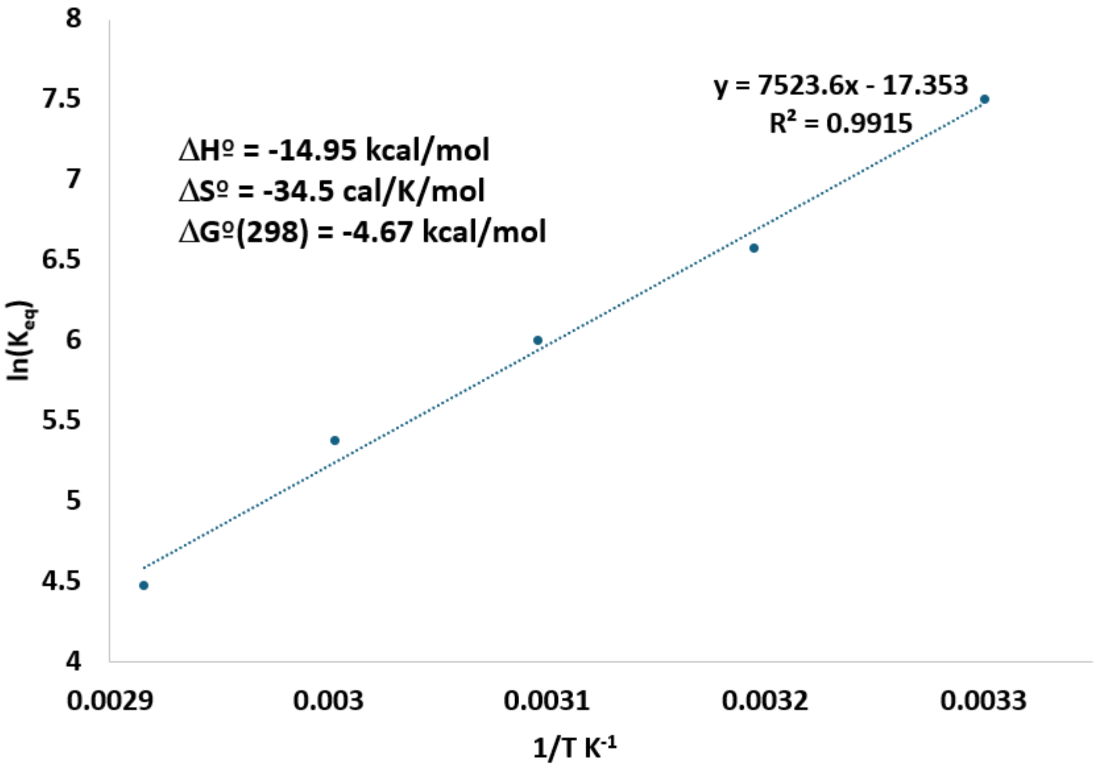

Figure S14. Van’t Hoff plot of data obtained by ^{1}H NMR for a temperature range of 303 – 343K.

6) DFT Studies

6.1. Computational methods

DFT calculations were performed using Gaussian 09 (Revision D.01) using an ultrafine integration grid (int=ultrafine).[S23] Geometry optimisations and frequency calculations were performed using the M06-L (for Si, Al, Ga) or BP86-D3 (for In) density functional including solvent corrections (PCM, benzene, ε = 2.2706) with SDDAll (Al, Ga, Zn), 6-31G** (C, H) and 6-311+G* (N, Si) basis set. Frequency analyses for all stationary points were performed using the enhanced criteria to confirm the nature of the structures as either minima (no imaginary frequency) or transition states (only one imaginary frequency). The electronic energies of the optimised geometries were calculated using the M06-L (for Si, Al, Ga) or BP86-D3 (for In) functional including solvent corrections with def2TZVPP[S24] basis sets for all atoms with solvent corrections (PCM, benzene or THF). The Gibbs free energy correction from the frequency calculation was added to this electronic energy to generate Gibbs free energy values for the calculated stationary points. For these systems it appears the same functional cannot be used to accurately model both the lighter (Al, Ga) and heavier (In) group 13 elements, hence we investigated a series of functionals ultimately using two different functional to model the formation of **2a-c** and **3** as separate processes, to provide the best match to experimental results.

Intrinsic reaction coordinate (IRC) calculations were used to connect transition states and minima located on the potential energy surface allowing a full energy profile (calculated at 298.15 K, 1 atm.) of the reaction to be constructed.

NBO analysis was performed and compared at the level of M06-L/def2tzvpp, M06-L-D3/def2tzvpp, B3PW91-D3/def2TZVPP, wB97xD/ def2tzvpp and BP86-D3/ def2TZVPP for all atoms and solvent corrections (PCM, benzene, ε = 2.2706) or (PCM, tetrahydrofuran, ε = 7.4257) by using NBO 6.0.[S25]

NLMO calculations were performed at the level of M06-L/def2tzvpp or BP86-D3/ def2tzvpp for all atoms and solvent corrections (PCM, benzene, ε = 2.2706) or (PCM, tetrahydrofuran, ε = 7.4257). [S25]

QTAIM analysis was performed at the B3PW91-D3/def2TZVPP and BP86-D3/ def2TZVPP level for all atoms and solvent corrections (PCM, benzene, ε = 2.2706) or (PCM, tetrahydrofuran, ε = 7.4257) and conducted using the AIMAll software. [S26-S27]

ETS-NOCV[S28] calculations were performed using DFT as implemented in Orca 4.2.1.[S29-S30] Optimised geometries of complexes from the Gaussian 09 calculations detailed above were used. Single-point calculations were performed using the B3PW91[S31] functional. The def2TZVPP basis set was used for all atoms. Graphical surface representations shown below were plotted using Avogadro 1.2.0.

IGMH analysis[S32] was performed using Multiwfn v3.8dev.[S33] NCIPLOT calculations were performed using NCIPLOT 4.2 using promolecular densities.[S34]

IBO calculation was performed using the ORCA 5.0.3[S35] software package using the B3PW91 and BP86-D3 functional with the def2TZVPP basis set for all atoms. Calculations were performed with the resolution of identity approximation for the Coulomb integrals, and chain of spheres approximation for the exchange integrals[S36] (RIJCOSX) with the def2/j auxiliary basis set.[S37] Atomic contributions to the IBOs were taken from the IBO calculation using the localised orbitals.

6.2. Calculated Reaction Pathway

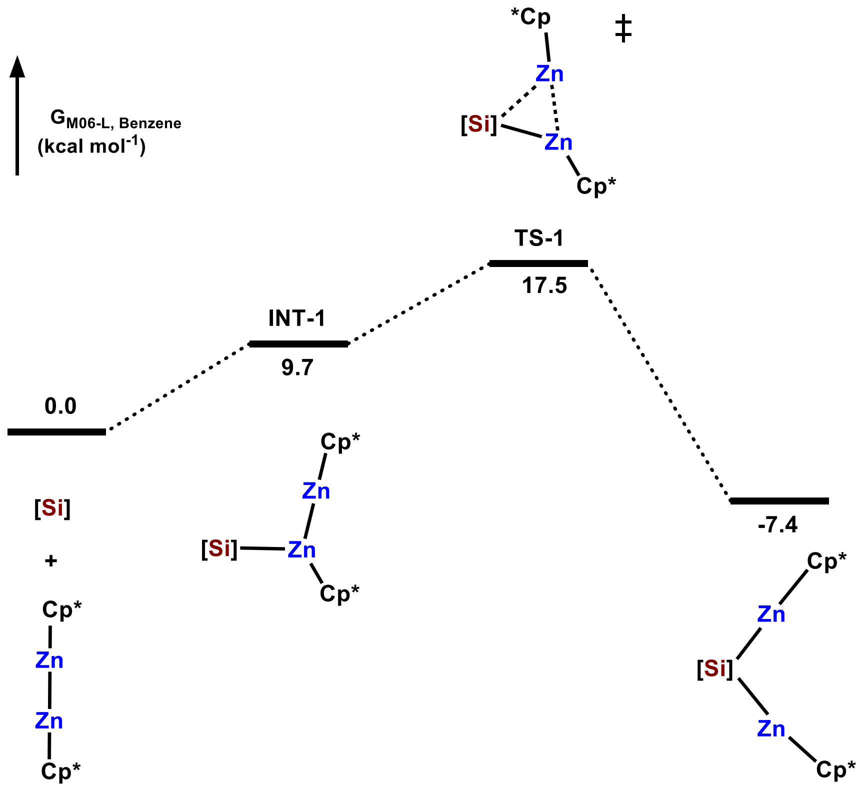

[Si] = {^{Dipp}NC(=CH2)CH(Me)N^{Dipp}}Si

Figure S15. Proposed pathway for the formation of **2a** based on DFT calculations G09: M06-L /def2TZVPP/ PCM (benzene) // M06-L / 6-31G** / 6-311+G* / SDDAll (Zn).

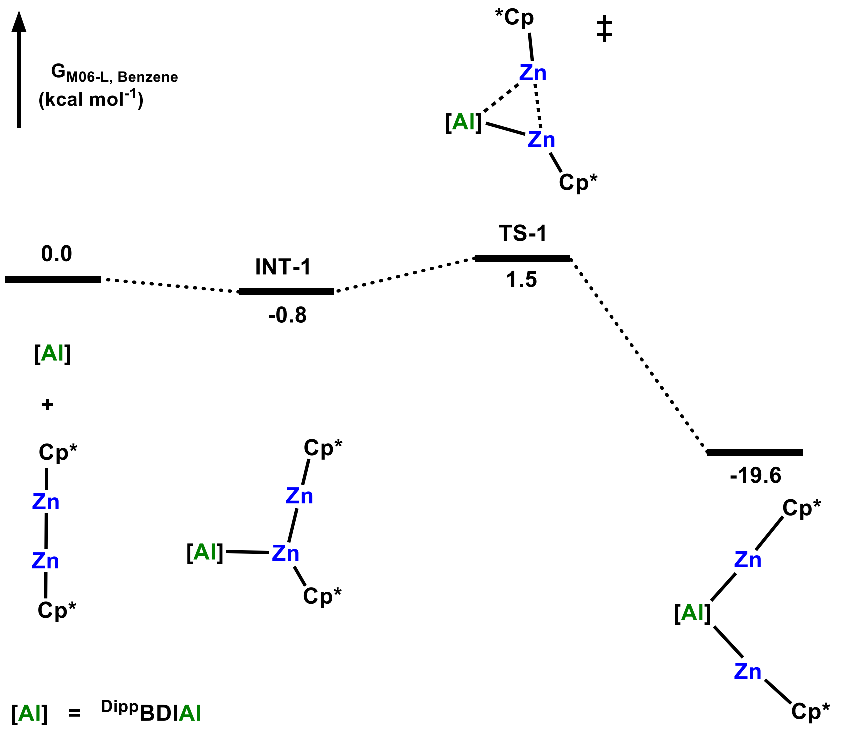

Figure S16. Proposed pathway for the formation of **2b** based on DFT calculations G09: M06-L / def2TZVPP/ PCM (benzene) // M06-L / 6-31G** / 6-311+G* / SDDAll (Al, Zn).

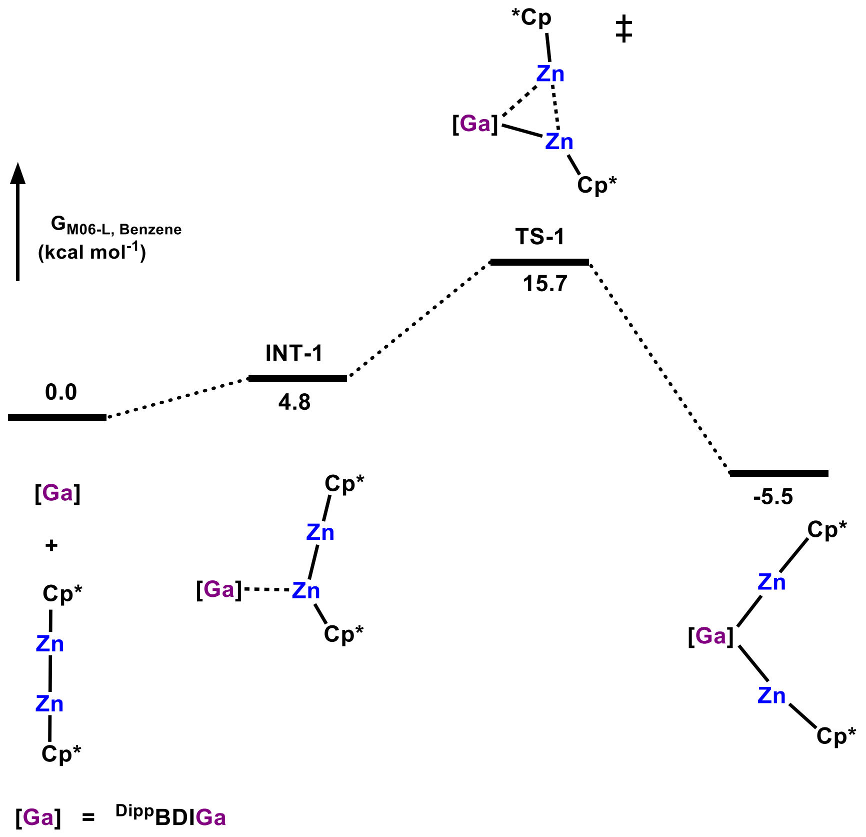

Figure S17. Proposed pathway for the formation of **2c** based on DFT calculations G09: M06-L / def2TZVPP/ PCM (benzene) // M06-L / 6-31G** / 6-311+G* / SDDAll (Ga, Zn).

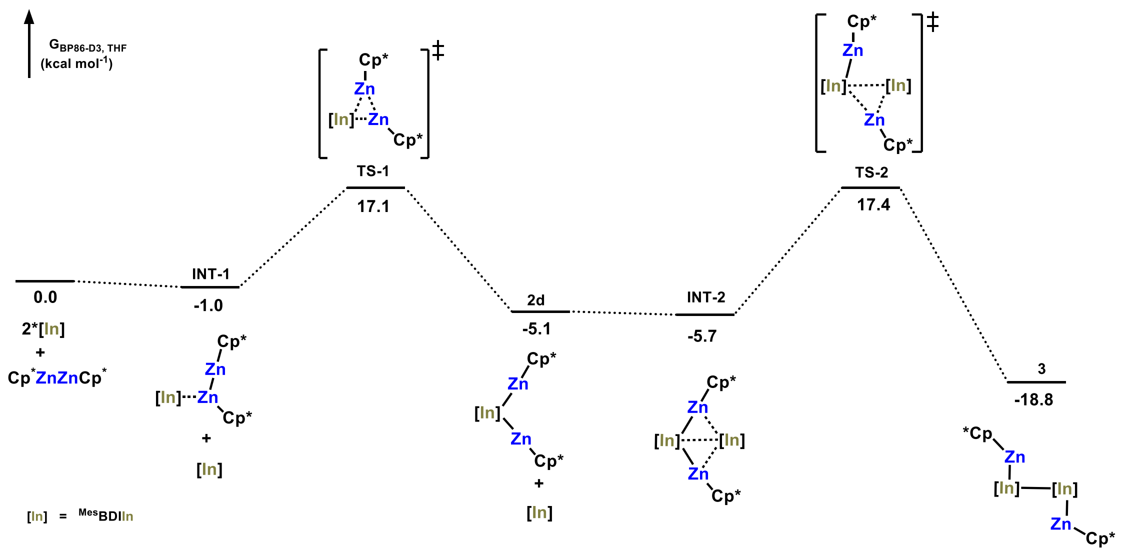

Figure S18. Proposed pathway for the formation of **3** via putative intermediate **2d** based on DFT calculations G16: BP86-D3/ def2TZVPP/ PCM (Tetrahydrofuran) // BP86-D3 / 6-31G** / 6-311+G* / SDDAll (Zn, In).

6.3. Optimised stationary points

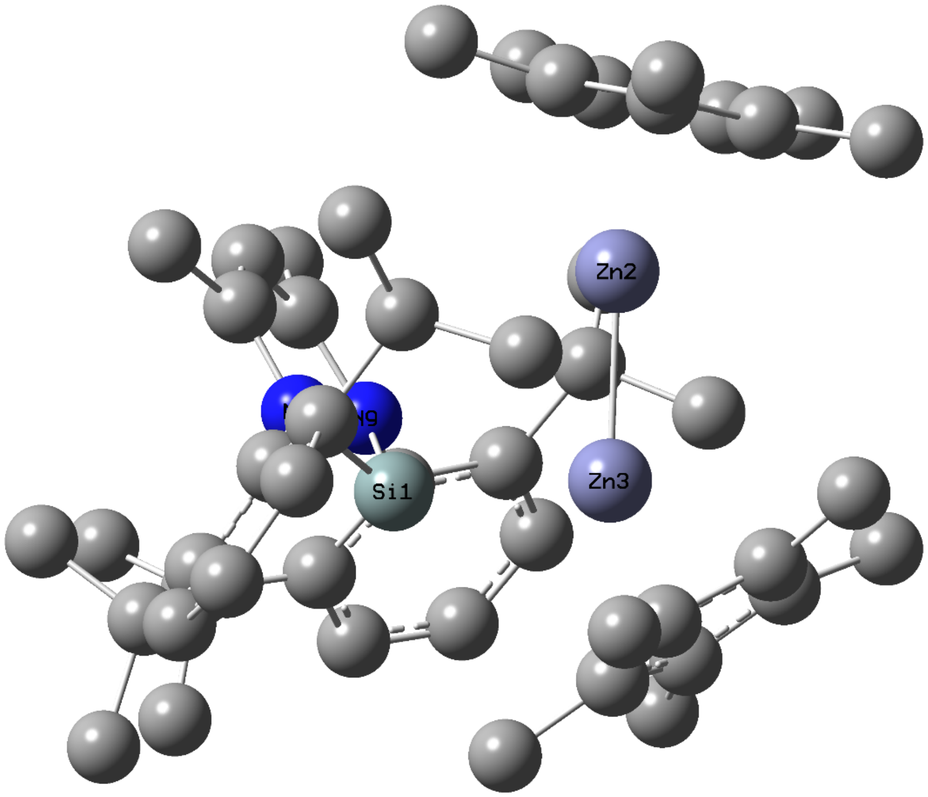

Si-INT-1

Calculated Bond Lengths and Angles	
**Si1–Zn2**	3.632
**Si1–Zn3**	2.614
**Zn2–Zn3**	2.371
**∠Zn2–Si1–Zn3**	40.68
**∠Si1–Zn2–Zn3**	45.83
**∠Si1–Zn3–Zn2**	93.39

Table S4. Calculated Bond Lengths (Å) and Angles (°) of **Si-INT-1**

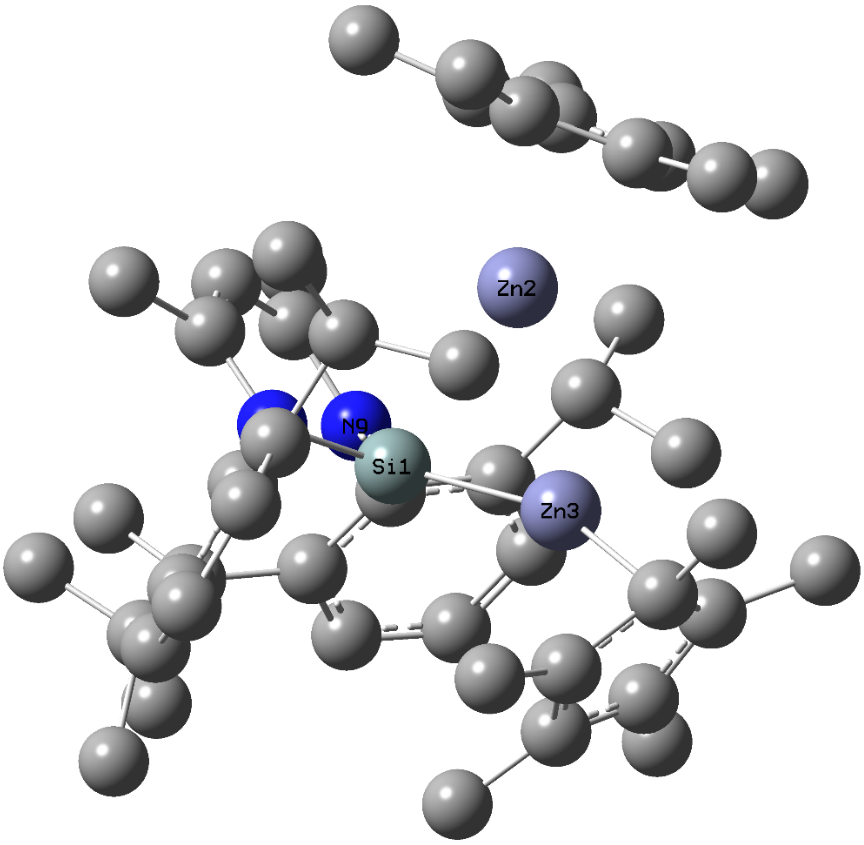

Si-TS1

Calculated Bond Lengths and Angles	
**Si1–Zn2**	2.627
**Si1–Zn3**	2.271
**Zn2–Zn3**	2.701
**∠Zn2–Si1–Zn3**	66.47
**∠Si1–Zn2–Zn3**	50.44
**∠Si1–Zn3–Zn2**	63.08

Table S5. Calculated Bond Lengths (Å) and Angles (°) of **Si-TS-1**

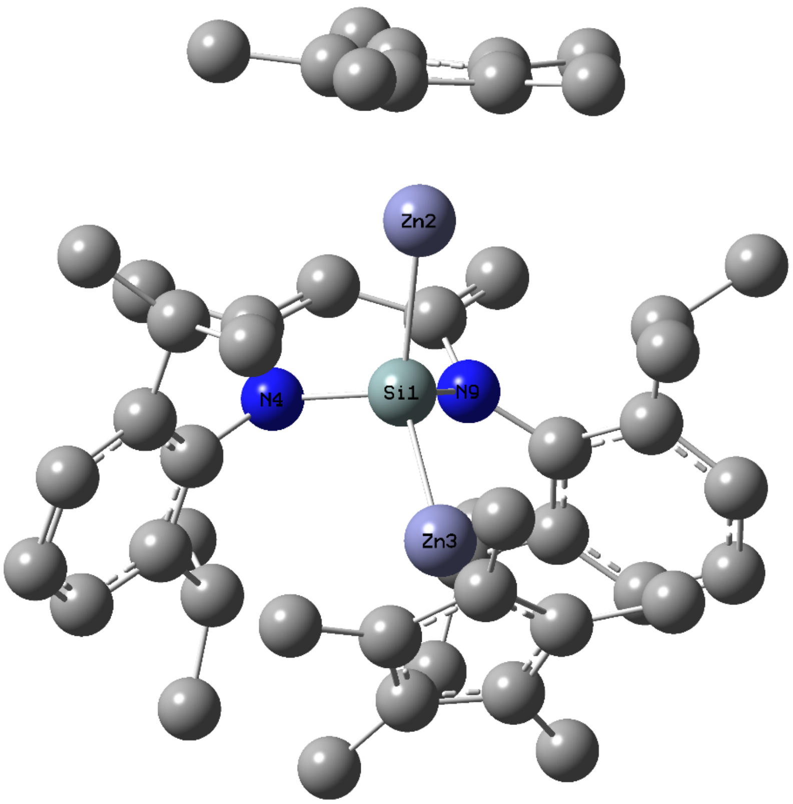

2a

	**Exp.**	**Calc.**
**Si1–Zn2**	2.383	2.373
**Si1–Zn3**	2.327	2.312
**Zn2–Zn3**	4.158	4.213
**∠Zn2–Si1–Zn3**	123.99	128.14
**∠Si1–Zn2–Zn3**	27.64	25.57
**∠Si1–Zn3–Zn2**	28.37	26.29

Table S6. Comparison of Calculated and Solid-State Bond Lengths (Å) and Angles (°) of **2a**

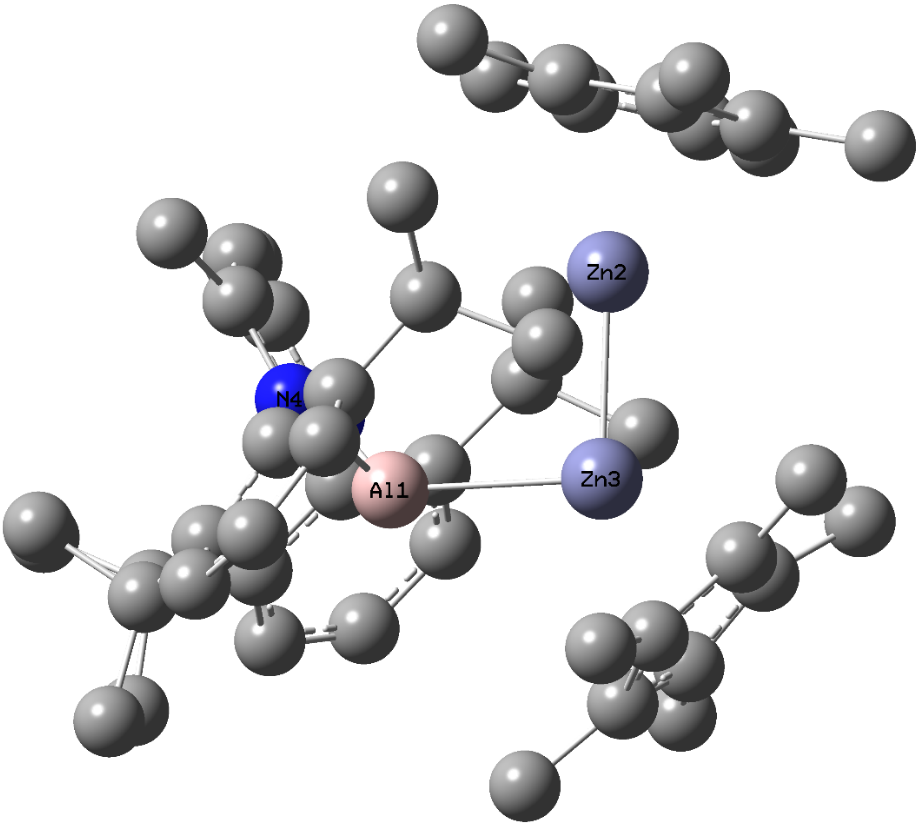

Al-INT-1

Calculated Bond Lengths and Angles	
**Al1–Zn2**	3.574
**Al1–Zn3**	2.496
**Zn2–Zn3**	2.419
∠**Zn2–Al1–Zn3**	42.52
∠**Al1–Zn2–Zn3**	44.20
∠**Al1–Zn3–Zn2**	93.28

Table S7. Calculated Bond Lengths (Å) and Angles (°) of **Al-INT-1**

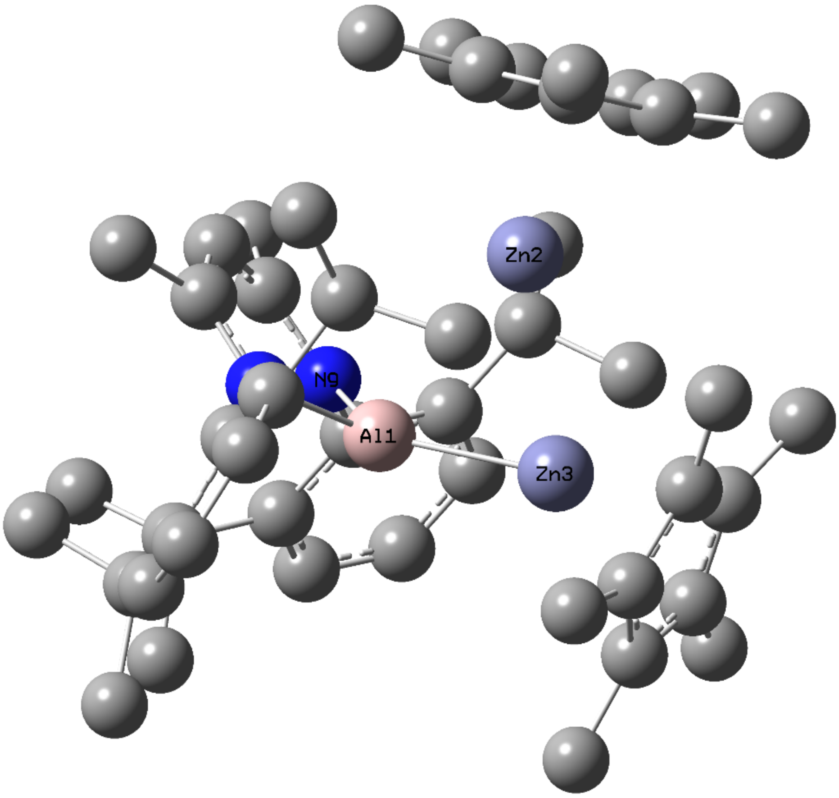

Al-TS1

Calculated Bond Lengths and Angles	
**Al1–Zn2**	2.938
**Al1–Zn3**	2.357
**Zn2–Zn3**	2.743
**∠Zn2–Al1–Zn3**	61.24
**∠Al1–Zn2–Zn3**	48.89
**∠Al1–Zn3–Zn2**	69.87

Table S8. Calculated Bond Lengths (Å) and Angles (°) of **Al-TS1**

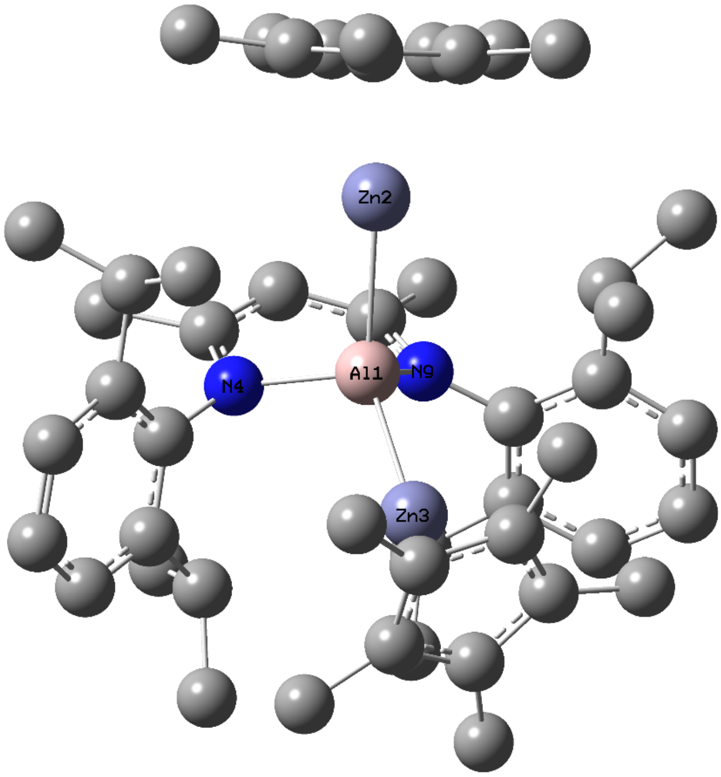

2b

	**Exp.**	**Calc.**
**Al1–Zn2**	2.496	2.502
**Al1–Zn3**	2.410	2.424
**Zn2–Zn3**	4.374	4.472
**∠Zn2–Al1–Zn3**	126.11	130.39
**∠Al1–Zn2–Zn3**	26.44	24.39
**∠Al1–Zn3–Zn2**	27.45	25.22

Table S9. Comparison of Calculated and Solid-State Bond Lengths (Å) and Angles (°) of **2b**

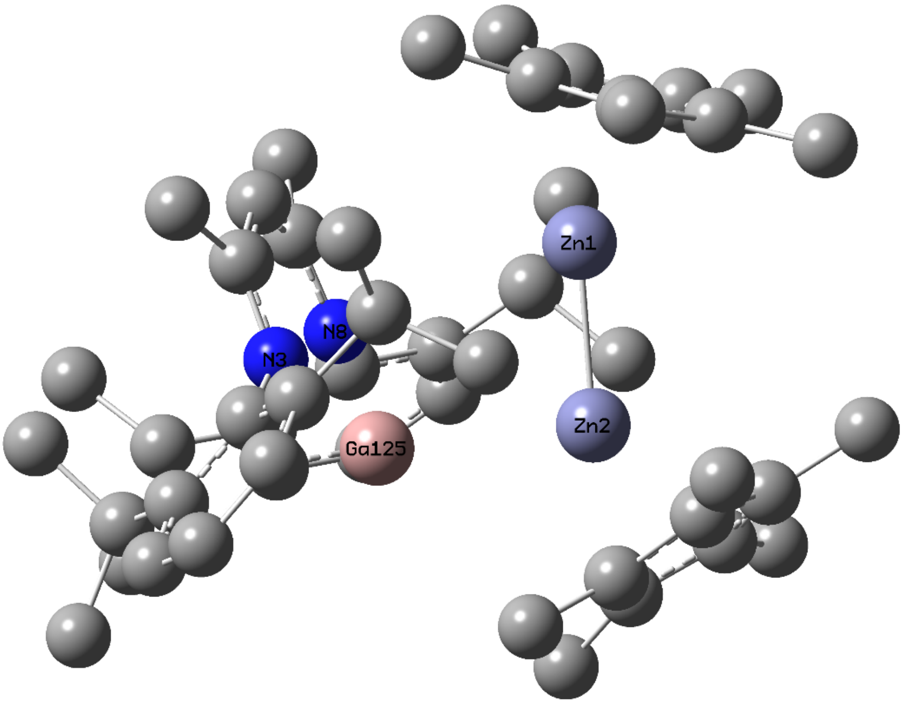

Ga-INT-1

Calculated Bond Lengths and Angles	
**Ga125–Zn1**	3.814
**Ga125–Zn2**	2.893
**Zn1–Zn2**	2.336
**∠Zn1–Ga125–Zn2**	37.70
**∠Ga125–Zn1–Zn2**	49.23
**∠Ga125–Zn2–Zn1**	93.08

Table S10. Calculated Bond Lengths (Å) and Angles (°) of **Ga-INT-1**

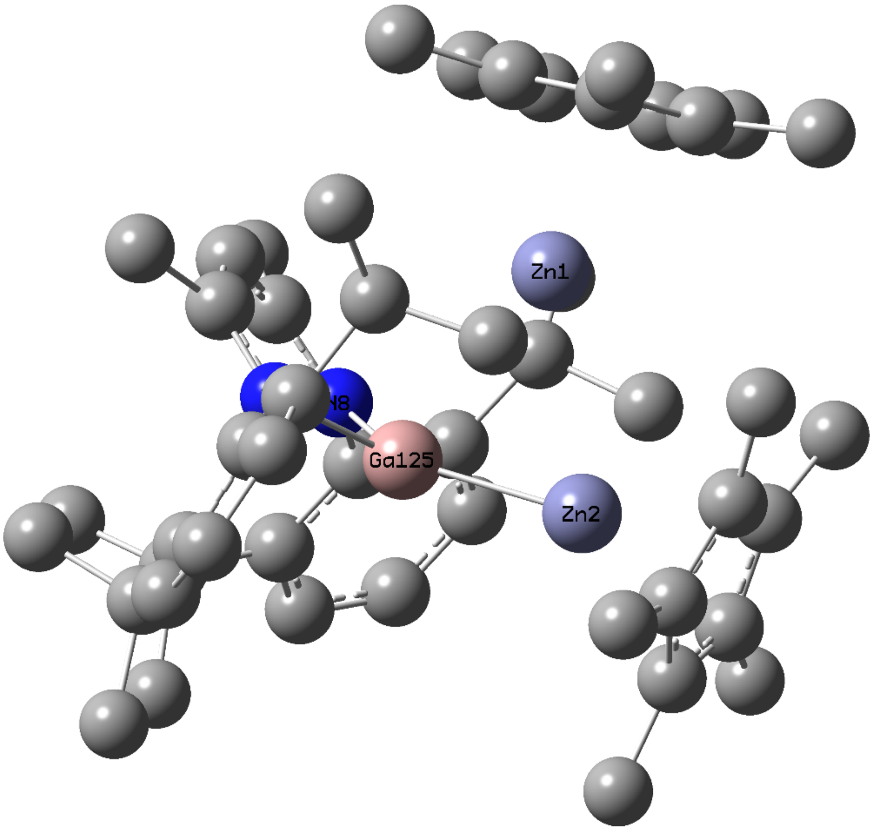

Ga-TS1

Calculated Bond Lengths and Angles	
**Ga125–Zn1**	2.888
**Ga125–Zn2**	2.341
**Zn1–Zn2**	2.930
**∠Zn1–Ga125–Zn2**	67.23
**∠Ga125–Zn1–Zn2**	47.44
**∠Ga125–Zn2–Zn1**	65.32

Table S11. Calculated Bond Lengths (Å) and Angles (°) of **Ga-TS-1**

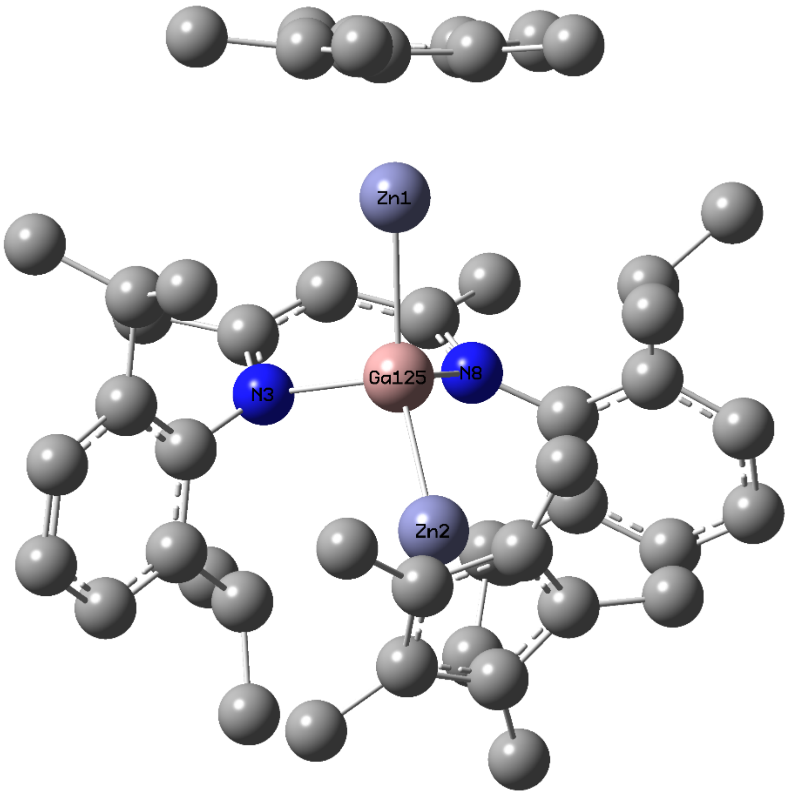

2c

	Exp.	Calc.
**Ga125–Zn1**	2.458	2.470
**Ga125–Zn2**	2.371	2.404
**Zn1–Zn2**	4.442	4.533
**∠Zn1–Ga125–Zn2**	133.84	136.85
**∠Ga125–Zn1–Zn2**	22.64	21.27
**∠Ga125–Zn2–Zn1**	23.52	21.88

Table S12. Comparison of Calculated and Solid-State Bond Lengths (Å) and Angles (°) of **2c**

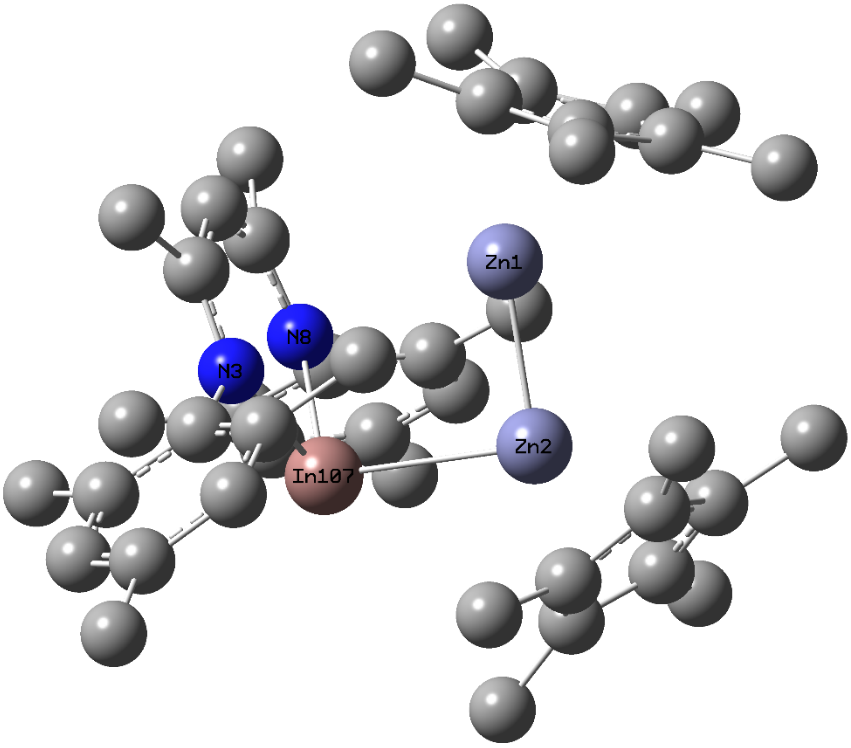

In-INT-1

Calculated Bond Lengths and Angles	
**In107–Zn1**	3.659
**In107–Zn2**	2.841
**Zn1–Zn2**	2.325
**∠Zn1–In107–Zn2**	39.44
**∠In107–Zn1–Zn2**	50.93
**∠In107–Zn2–Zn1**	89.62

Table S13. Calculated Bond Lengths (Å) and Angles (°) of **In-INT-1**

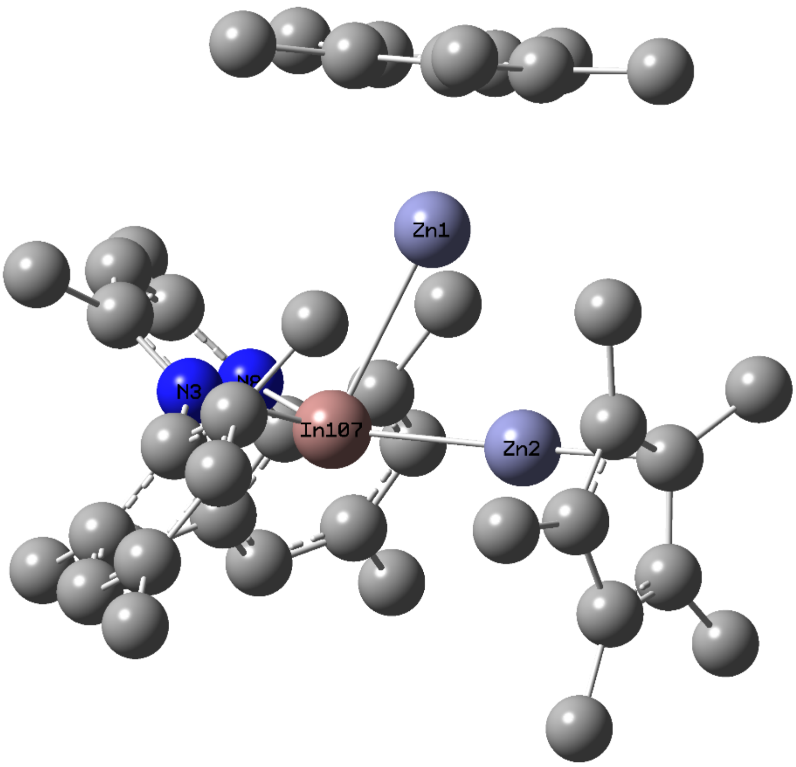

In-TS-1

Calculated Bond Lengths and Angles	
**In107–Zn1**	2.792
**In107–Zn2**	2.460
**Zn1–Zn2**	2.934
**∠Zn1–In107–Zn2**	67.59
**∠In107–Zn1–Zn2**	50.81
**∠In107–Zn2–Zn1**	61.60

Table S14. Calculated Bond Lengths (Å) and Angles (°) of **In-TS-1**

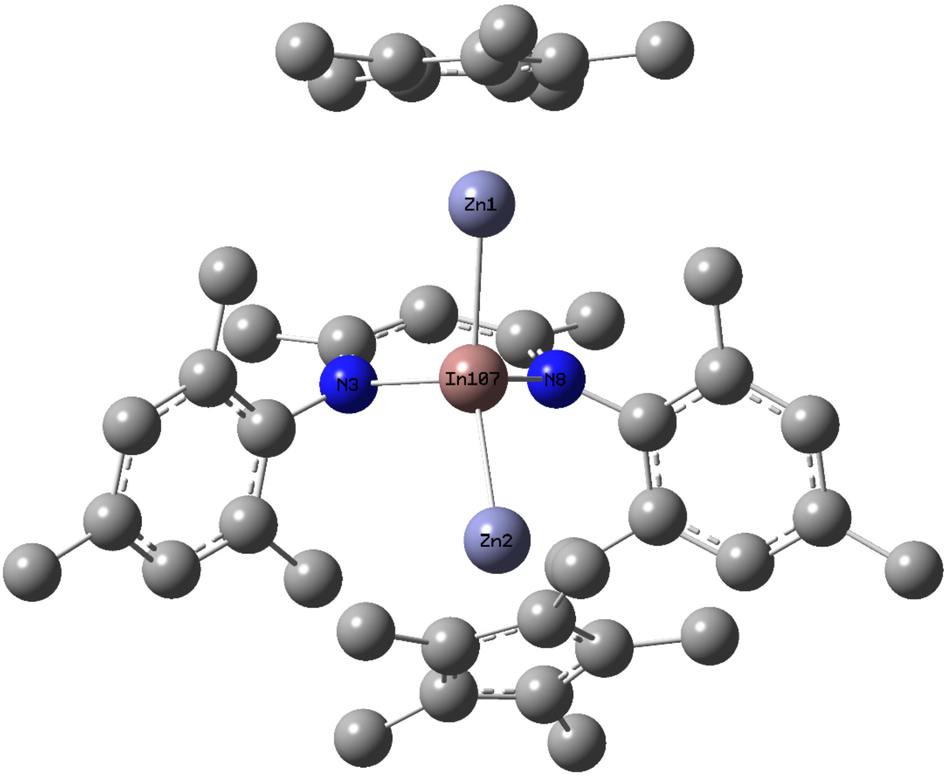

2d

Calculated Bond Lengths and Angles	
**In107–Zn1**	2.538
**In107–Zn2**	2.505
**Zn1–Zn2**	4.780
**∠Zn1–In107–Zn2**	142.83
**∠In107–Zn1–Zn2**	18.46
**∠In107–Zn2–Zn1**	18.72

Table S15. Calculated Bond Lengths (Å) and Angles (°) of **2d**

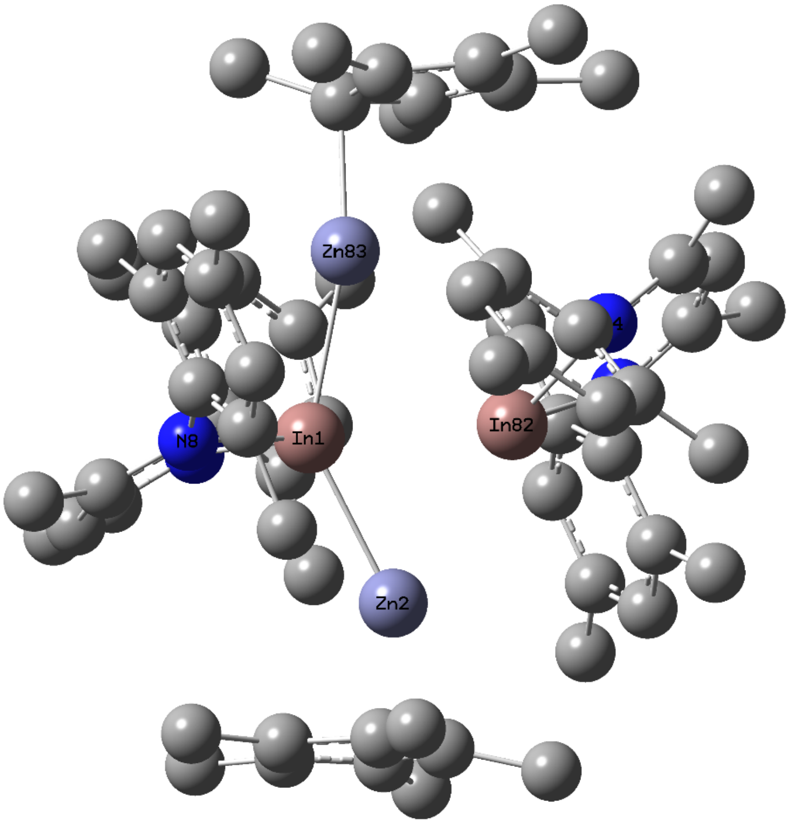

In-INT-2

Calculated Bond Lengths and Angles	
**In1–Zn2**	2.554
**In1–Zn83**	2.619
**Zn2–Zn83**	3.371
**In82-Zn2**	3.527
**In82-Zn83**	3.838
**In1-In82**	3.371
**∠Zn2–In1–Zn83**	142.07
**∠In1–Zn83–In82**	59.42
**∠Zn2–In82–Zn83**	83.13
**∠In1–Zn2–In82**	65.05

Table S16. Calculated Bond Lengths (Å) and Angles (°) of **In-INT-2**

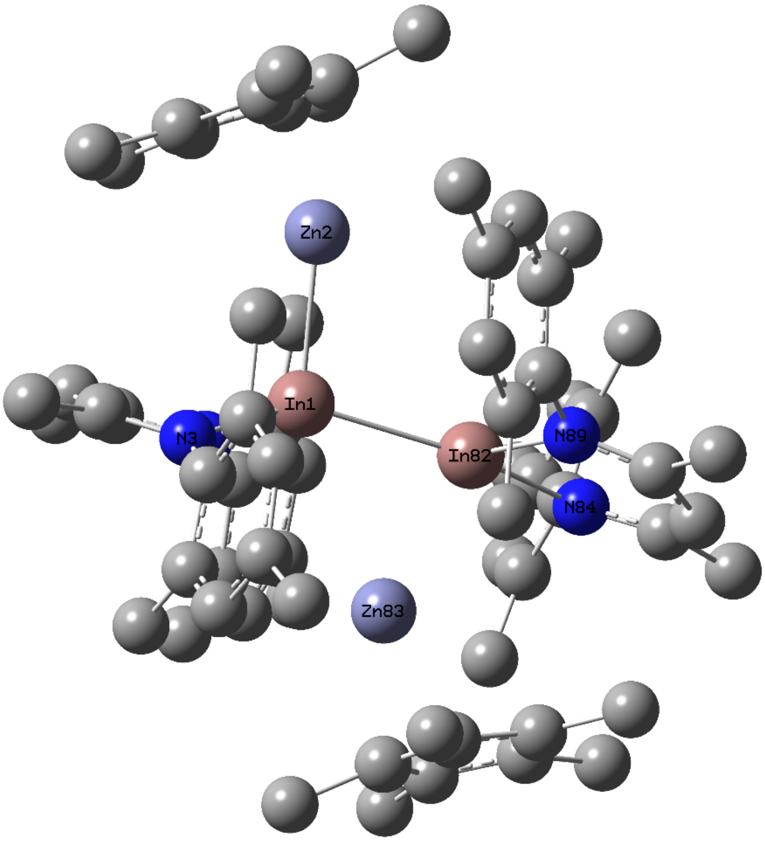

In-TS-2

Calculated Bond Lengths and Angles	
**In1–Zn2**	2.533
**In1–Zn83**	3.303
**Zn2–Zn83**	3.371
**In82-Zn2**	3.527
**In82-Zn83**	5.587
**In1-In82**	2.745
**∠Zn2–In1–Zn83**	146.11
**∠In1–Zn83–In82**	50.93
**∠Zn2–In82–Zn83**	100.33
**∠In1–Zn2–In82**	39.86

Table S17. Calculated Bond Lengths (Å) and Angles (°) of **In-TS-2**

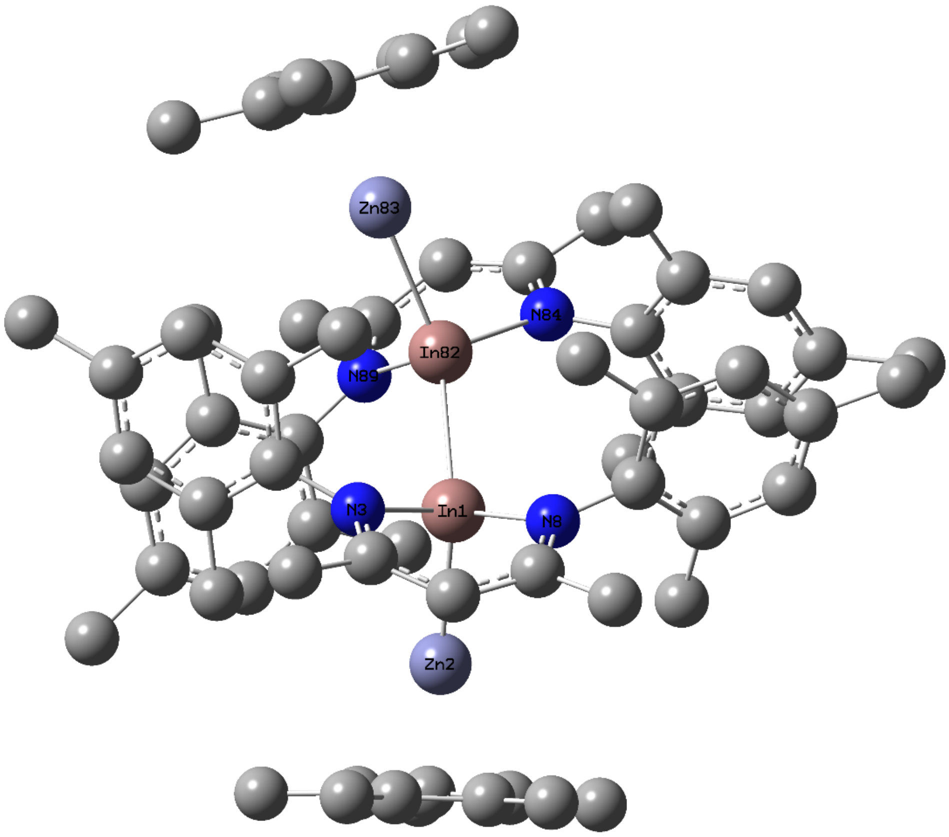

3

	Exp.	Calc.
**In1–Zn2**	2.536	2.523
**In1–Zn83**	4.849	4.779
**Zn2–Zn83**	7.230	7.041
**In82-Zn2**	4.849	4.767
**In82-Zn83**	2.536	2.523
**In1-In82**	2.760	2.729
**∠Zn2–In1–Zn83**	155.20	147.67
**∠In1–Zn83–In82**	24.80	25.55
**∠Zn2–In82–Zn83**	155.20	148.37
**∠In1–Zn2–In82**	24.80	25.86
**∠Zn2–In1–In82**	132.53	130.35
**∠In1–Zn82–Zn83**	132.53	130.94

Table S18. Comparison of Calculated and Solid-State Bond Lengths (Å) and Angles (°) of **3**

6.4. NBO Analysis

NBO analysis was performed and compared at the level of M06-L/def2tzvpp, M06-L-D3/def2tzvpp, B3PW91-D3/def2TZVPP, wB97xD/def2tzvpp and BP86-D3/ def2tzvpp for all atoms and solvent corrections (PCM, benzene, ε = 2.2706) or (PCM, tetrahydrofuran, ε = 7.4257) by using NBO 6.0.[S33]

6.4.1. NPA Charge

The NBO calculations of **1a-d**, **2a-d, 3** and **Cp*ZnZnCp*** were performed using different methods. NPA charges of key atomic sites and fragments were within close agreement of one another. Where deviation of the charges occurs with different functionals, it is most likely an effect of small changes in geometry obtained with different methods.

As a further point of comparison NBO calculations were also conducted on **1a-d, 1a-d-H_2 and 1a-d-Li_2**. The latter two series of compounds provide benchmarks for expected charge distribution for products of addition of H_2 to **1a-d**, along with theoretical dilithio products.

Cp*-Zn-Zn-Cp*

Atom	No	Charge (M06-L)	Charge (M06-L-D3)	Charge (B3PW91-D3)	Charge (wB97xD)	Charge (BP86-D3)
Zn1	1	0.87	0.87	0.88	0.89	0.87
Zn2	2	0.87	0.87	0.88	0.89	0.87

Table S19. NPA charge data for **Cp*-Zn-Zn-Cp***

Wiberg bond index matrix					
	M06-L	M06-L-D3	B3PW91-D3	wB97xD	BP86-D3
Zn1 – Zn2	0.853	0.853	0.859	0.864	0.847

Table S20. WBI data for **Cp*-Zn-Zn-Cp***

^{Dipp}BDISi

Atom	No	Charge (M06-L)	Charge (M06-L-D3)	Charge (B3PW91-D3)	Charge (wB97xD)
Si1	1	1.17	1.17	1.19	1.21

Table S21. NPA charge data for **^{Dipp}BDISi**

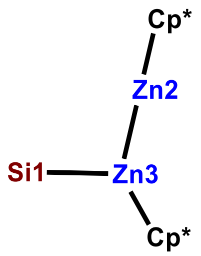

Atom	No	Charge (M06-L)
Si1	1	0.93
Zn2	2	0.97
Zn3	3	1.03

Table S22. NPA charge data for **Si-INT-1**

Wiberg bond index matrix (M06-L)	
Si1 – Zn2	0.219
Si1 – Zn3	0.319
Zn2 – Zn3	0.542

Table S23. WBI data for **Si-INT-1**

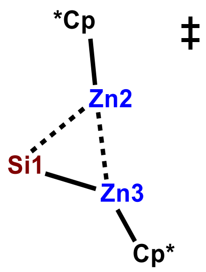

Atom	No	Charge (M06-L)
Si1	1	0.37
Zn2	2	1.07
Zn3	3	1.37

Table S24. NPA charge data for **Si-TS-1**

Wiberg bond index matrix (M06-L)	
Si1 – Zn2	0.686
Si1 – Zn3	0.456
Zn2 – Zn3	0.108

Table S25. WBI data for **Si-TS-1**

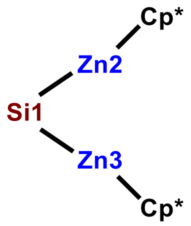

Atom	No	Charge (M06-L)	Charge (M06-L-D3)	Charge (B3PW91-D3)	Charge (wB97xD)
Si1	1	0.28	0.28	0.28	0.29
Zn2	2	1.30	1.30	1.30	1.32
Zn3	3	1.36	1.36	1.37	1.34

Table S26. NPA charge data for **2a**

Wiberg bond index matrix				
	M06-L	M06-L-D3	B3PW91-D3	wB97xD
Si1 – Zn2	0.676	0.676	0.681	0.685
Si1 – Zn3	0.682	0.682	0.682	0.677
Zn2 – Zn3	0.009	0.009	0.009	0.008

Table S27. WBI data for **2a**

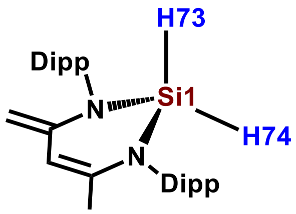

Atom	No	Charge (M06-L)
Si1	1	1.58
H73	73	-0.23
H74	74	-0.21

Table S28. NPA charge data for **1a-H_2**

Wiberg bond index matrix (M06-L)	
Si1 – H73	0.869
Si1 – H74	0.902
H73 ---- H74	0.019

Table S29. WBI data for **1a-H_2**

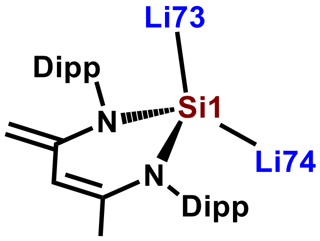

Atom	No	Charge (M06-L)
Si1	1	-0.24
Li73	73	0.83
Li74	74	0.86

Table S30. NPA charge data for **1a-Li_2**

Wiberg bond index matrix (M06-L)	
Si1 – Li73	0.210
Si1 – Li74	0.237
Li73 ---- Li74	0.003

Table S31. WBI data for **1a-Li_2**

^{Dipp}BDIAl

Atom	No	Charge (M06-L)	Charge (M06-L-D3)	Charge (B3PW91-D3)	Charge (wB97xD)
Al1	1	0.74	0.74	0.75	0.76

Table S32. NPA charge data for ^{Dipp}**BDIAl**

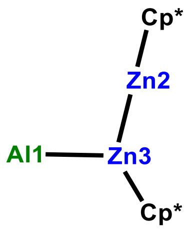

Atom	No	Charge (M06-L)
Al1	1	0.54
Zn2	2	0.84
Zn3	3	1.02

Table S33. NPA charge data for **Al-INT-1**

Wiberg bond index matrix (M06-L)	
Al1 – Zn2	0.417
Al1 – Zn3	0.494
Zn2 – Zn3	0.404

Table S34. WBI data for **Al-INT-1**

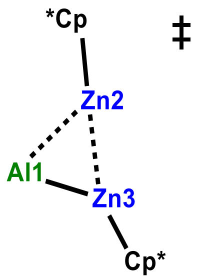

Atom	No	Charge (M06-L)
Al1	1	0.29
Zn2	2	0.78
Zn3	3	1.25

Table S35. NPA charge data for **Al-TS-1**

Wiberg bond index matrix (M06-L)	
Al1 – Zn2	0.752
Al1 – Zn3	0.715
Zn2 – Zn3	0.083

Table S36. WBI data for **Al-TS-1**

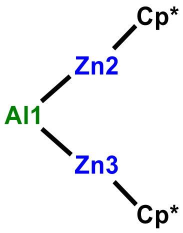

Atom	No	Charge (M06-L)	Charge (M06-L-D3)	Charge (B3PW91-D3)	Charge (wB97xD)
Al1	1	0.13	0.13	0.12	0.12
Zn2	2	1.09	1.09	1.09	1.11
Zn3	3	1.13	1.13	1.14	1.16

Table S37. NPA charge data for **2b**

Wiberg bond index matrix				
	**M06-L**	**M06-L-D3**	**B3PW91-D3**	**wB97xD**
Al1 – Zn2	0.833	0.833	0.837	0.847
Al1 – Zn3	0.848	0.848	0.853	0.856
Zn2 – Zn3	0.012	0.012	0.011	0.010

Table S38. WBI data for **2b**

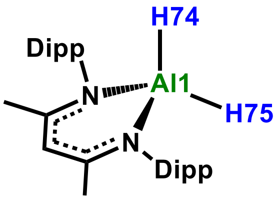

Atom	No	Charge (M06-L)
Al1	1	1.42
H74	74	-0.42
H75	75	-0.42

Table S39. NPA charge data for **1b-H$_2$**

Wiberg bond index matrix (M06-L)	
Al1 – H74	0.782
Al1 – H75	0.788
H74 ---- H75	0.011

Table S40. WBI data for **1b-H$_2$**

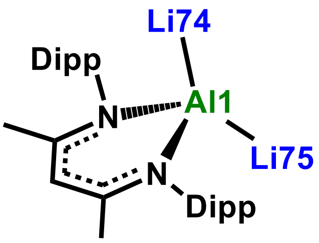

Atom	No	Charge (M06-L)
Al1	1	-0.45
Li74	74	0.68
Li75	75	0.54

Table S41. NPA change data for **1b-Li$_2$**

Wiberg bond index matrix (M06-L)	
Al1 – Li74	0.480
Al1 – Li75	0.604
Li74 ---- Li75	0.035

Table S42. WBI data for **1b-Li$_2$**

^{Dipp}BDIGa

Atom	No	Charge (M06-L)	Charge (M06-L-D3)	Charge (B3PW91-D3)	Charge (wB97xD)
Ga73	73	0.71	0.71	0.72	0.74

Table S43. NPA charge data for ^{Dipp}**BDIGa**

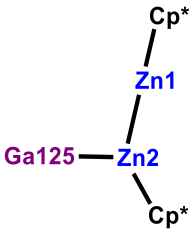

Atom	No	Charge (M06-L)
Ga125	125	0.49
Zn1	1	0.93
Zn2	2	0.94

Table S44. NPA charge data for **Ga-INT-1**

Wiberg bond index matrix (M06-L)	
Ga125 – Zn1	0.163
Ga125 – Zn2	0.230
Zn1 – Zn2	0.628

Table S45. WBI data for **Ga-INT-1**

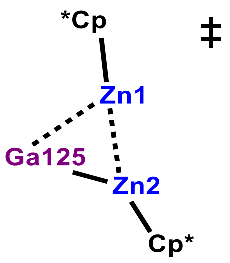

Atom	No	Charge (M06-L)
Ga125	125	0.09
Zn1	1	0.78
Zn2	2	1.37

Table S46. NPA charge data for **Ga-TS-1**

Wiberg bond index matrix (M06-L)	
Ga125 – Zn2	0.785
Ga125 – Zn3	0.644
Zn1 – Zn2	0.055

Table S47. WBI data for **Ga-TS-1**

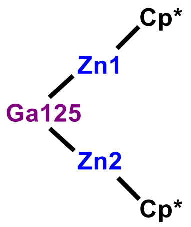

Atom	No	Charge (M06-L)	Charge (M06-L-D3)	Charge (B3PW91-D3)	Charge (wB97xD)
Ga125	125	-0.12	-0.12	-0.16	-0.14
Zn1	1	1.19	1.19	1.20	1.21
Zn2	2	1.21	1.21	1.23	1.25

Table S48. NPA charge data for **2c**

Wiberg bond index matrix				
	M06-L	M06-L-D3	B3PW91-D3	wB97xD
Ga125 – Zn1	0.784	0.784	0.785	0.795
Ga125 – Zn2	0.789	0.789	0.789	0.792
Zn1 – Zn2	0.014	0.014	0.011	0.010

Table S49. WBI data for **2c**

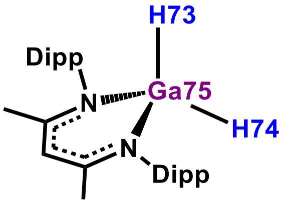

Atom	No	Charge (M06-L)
Ga75	75	1.18
H73	73	-0.33
H74	74	-0.32

Table S50. NPA charge data for **1c-H_2**

Wiberg bond index matrix (M06-L)	
Ga75 – H73	0.838
Ga75 – H74	0.843
H73 ---- H74	0.022

Table S51. WBI data for **1c-H_2**

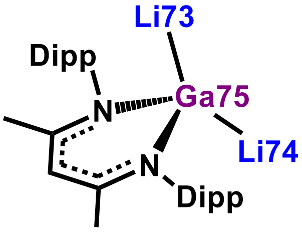

Atom	No	Charge (M06-L)
Ga75	75	-0.60
Li73	73	0.65
Li74	74	0.74

Table S52. NPA change data for **1c-Li_2**

Wiberg bond index matrix (M06-L)	
Ga75 – Li73	0.523
Ga75 – Li74	0.348
Li73 ---- Li74	0.029

Table S53. WBI data for **1c-Li_2**

^{Mes}BDIIn

Atom	No	Charge (BP86-D3)
In1	1	0.73

Table S54. NPA change data for ^{Dipp}BDIIn

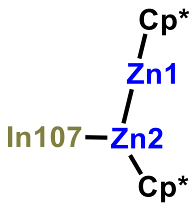

Atom	No	Charge (BP86-D3)
In107	107	0.616
Zn1	1	0.953
Zn2	2	0.836

Table S55. NPA change data for **In-INT-1**

Wiberg bond index matrix (BP86-D3)	
In107 – Zn1	0.170
In107 – Zn2	0.250
Zn1 – Zn2	0.563

Table S56. WBI data for **In-INT-1**

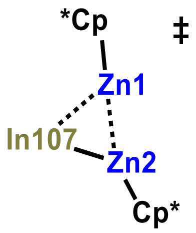

Atom	No	Charge (BP86-D3)
In107	107	0.16
Zn1	1	0.76
Zn2	2	1.19

Table S57. NPA change data for **In-TS-1**

Wiberg bond index matrix (BP86-D3)	
In107 – Zn1	0.807
In107 – Zn2	0.595
Zn1 – Zn2	0.043

Table S58. WBI data for **In-TS-1**

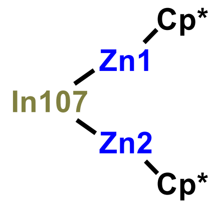

Atom	No	Charge (BP86-D3)
In107	107	0.08
Zn1	1	1.08
Zn2	2	1.12

Table S59. NPA charge data for **2d**

Wiberg bond index matrix (BP86-D3)	
In107 – Zn1	0.800
In107 – Zn2	0.821
Zn1 – Zn2	0.275

Table S60. WBI data for **2d**

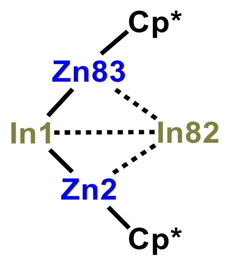

Atom	No	Charge (BP86-D3)
In1	1	0.11
Zn2	2	1.03
Zn83	83	1.02
In82	82	0.83

Table S61. NPA charge data for **In-INT-2**

Wiberg bond index matrix (BP86-D3)	
In1 – Zn2	0.676
In1 – Zn83	0.631
In1 – In82	0.510
In82 – Zn2	0.060
In82 – Zn83	0.060

Table S62. WBI data for **In-INT-2**

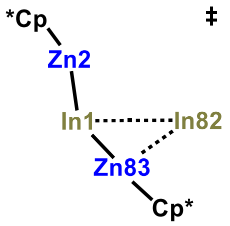

Atom	No	Charge (BP86-D3)
In1	1	0.32
Zn2	2	1.07
Zn83	83	0.76
In82	82	0.77

Table S63. NPA charge data for **In-TS-2**

Wiberg bond index matrix (BP86-D3)	
In1 – Zn2	0.648
In1 – Zn83	0.345
In1 – In82	0.917
In82 – Zn2	0.099
In82 – Zn83	0.418

Table S64. WBI data for **In-TS-2**

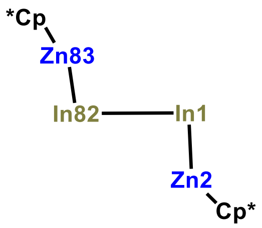

Atom	No	Charge (BP86-D3)
In1	1	0.58
Zn2	2	0.92
Zn83	83	0.91
In82	82	0.58

Table S65. NPA charge data for **3**

Wiberg bond index matrix (BP86-D3)	
In1 – Zn83	0.050
In1 – In82	0.820
In1 – Zn2	0.735
In82 – Zn2	0.050
In82 – Zn83	0.737

Table S66. WBI data for **3**

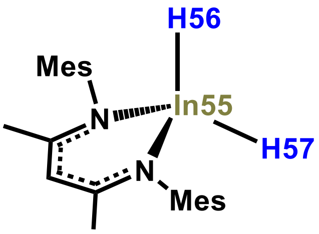

Atom	No	Charge (BP86-D3)
In55	55	1.11
H56	56	-0.31
H57	57	-0.31

Table S67. NPA change data for **1d-H_2**

Wiberg bond index matrix (M06-L)	
In55 – H56	0.838
In55 – H57	0.838
H56 ---- H57	0.035

Table S68. WBI data for **1d-H_2**

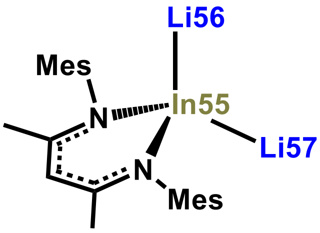

Atom	No	Charge (BP86-D3)
In55	55	-0.89
Li56	56	0.85
Li57	57	0.88

Table S69. NPA change data for **1d-Li_2**

Wiberg bond index matrix (M06-L)	
In55 – Li56	0.231
In55 – Li57	0.196
Li56 ---- Li57	0.006

Table S70. WBI data for **1d-Li_2**

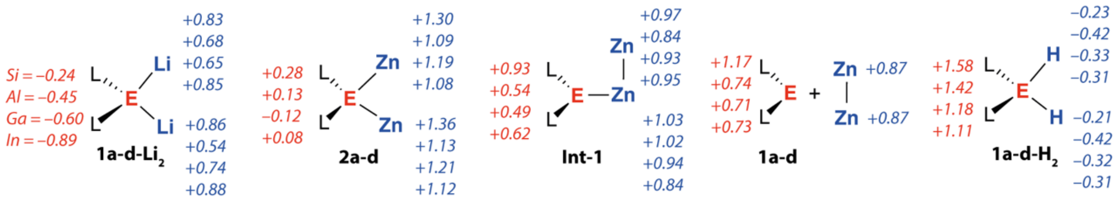

Figure S19. Comparison of NPA charges for stationary points in the pathway for the addition of Cp*ZnZnCp* to **1a-d**. For **a-c**, G09: M06-L /def2TZVPP/ PCM (benzene) // M06-L / 6-31G** / 6-311+G* / SDDAll (Ga, Zn). For **d**, G16: BP86-D3/def2TZVPP/ PCM (Tetrahydrofuran) // BP86-D3 / 6-31G** / 6-311+G* / SDDAll (Zn, In).

6.4.3. NPA Charge Difference

NPA Charge Difference (M06-L) ($\Delta Q_{(E-X)}$)		
**Si-INT-1**	$\Delta Q_{(Si1-Zn2)}$	-0.04
	$\Delta Q_{(Si1-Zn3)}$	-0.10
**Si-TS-1**	$\Delta Q_{(Si1-Zn2)}$	-0.70
	$\Delta Q_{(Si1-Zn3)}$	-1.00
**2a**	$\Delta Q_{(Si1-Zn2)}$	-1.02
	$\Delta Q_{(Si1-Zn3)}$	-1.08
**1a-H_2**	$\Delta Q_{(Si1-H73)}$	1.81
	$\Delta Q_{(Si1-H74)}$	1.79
**1a-Li_2**	$\Delta Q_{(Si1-Li73)}$	-1.07
	$\Delta Q_{(Si1-Li74)}$	-1.10
**Al-INT-1**	$\Delta Q_{(Al1-Zn2)}$	-0.30
	$\Delta Q_{(Al1-Zn3)}$	-0.48
**Al-TS-1**	$\Delta Q_{(Al1-Zn2)}$	-0.49
	$\Delta Q_{(Al1-Zn3)}$	-0.96
**2b**	$\Delta Q_{(Al1-Zn2)}$	-0.96
	$\Delta Q_{(Al1-Zn3)}$	-1.00
**1b-H_2**	$\Delta Q_{(Al1-H74)}$	1.84
	$\Delta Q_{(Al1-H75)}$	1.84
**1b-Li_2**	$\Delta Q_{(Al1-Li74)}$	-1.13
	$\Delta Q_{(Al1-Li75)}$	-0.99
**Ga-INT-1**	$\Delta Q_{(Ga125-Zn1)}$	-0.44
	$\Delta Q_{(Ga125-Zn2)}$	-0.45
**Ga-TS-1**	$\Delta Q_{(Ga125-Zn1)}$	-0.69
	$\Delta Q_{(Ga125-Zn2)}$	-1.28
**2c**	$\Delta Q_{(Ga125-Zn1)}$	-1.31
	$\Delta Q_{(Ga125-Zn2)}$	-1.33
**1c-H_2**	$\Delta Q_{(Ga75-H73)}$	1.51
	$\Delta Q_{(Ga75-H74)}$	1.50
**1c-Li_2**	$\Delta Q_{(Ga75-Li73)}$	-1.25
	$\Delta Q_{(Ga75-Li74)}$	-1.34
**In-INT-1**	$\Delta Q_{(In107-Zn1)}$	-0.34
	$\Delta Q_{(In107-Zn2)}$	-0.22
**In-TS-1**	$\Delta Q_{(In107-Zn1)}$	-0.60
	$\Delta Q_{(In107-Zn2)}$	-1.03
**2d**	$\Delta Q_{(In107-Zn1)}$	-1.00
	$\Delta Q_{(In107-Zn2)}$	-1.04
**1d-H_2**	$\Delta Q_{(In55-H56)}$	1.42
	$\Delta Q_{(In55-H57)}$	1.42
**1d-Li_2**	$\Delta Q_{(In55-Li56)}$	-1.74
	$\Delta Q_{(In55-Li57)}$	-1.77

Table S71. Charge differences between E and Zn (or H or Li) in selected stationary points.

6.4.2. NPA Fragment Charge

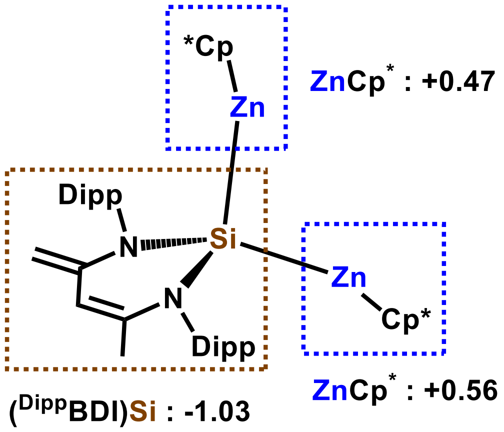

Figure S20. Calculated fragment charges in **2a**. Units of charge are those from each fragment highlighted by a box.

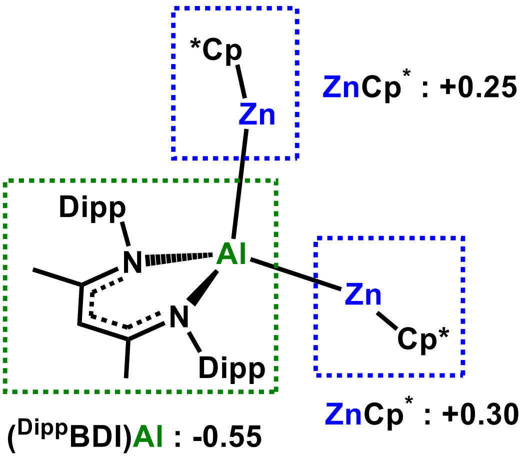

Figure S21. Calculated fragment charges in **2b**. Units of charge are those from each fragment highlighted by a box.

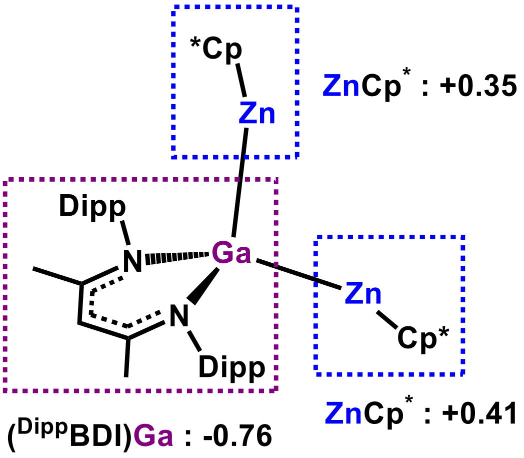

Figure S22. Calculated fragment charges in **2c**. Units of charge are those from each fragment highlighted by a box.

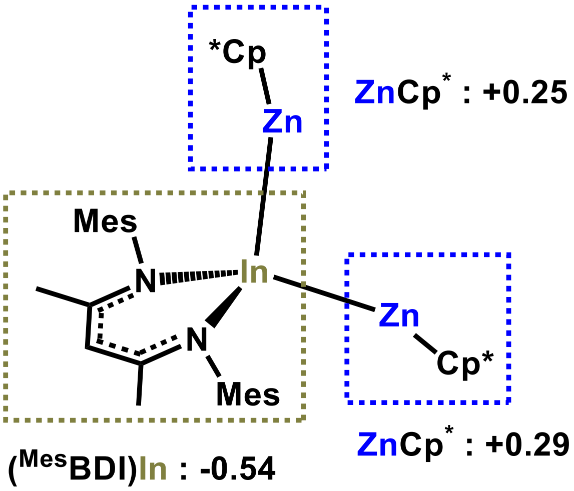

Figure S23. Calculated fragment charges in **2d**. Units of charge are those from each fragment highlighted by a box.

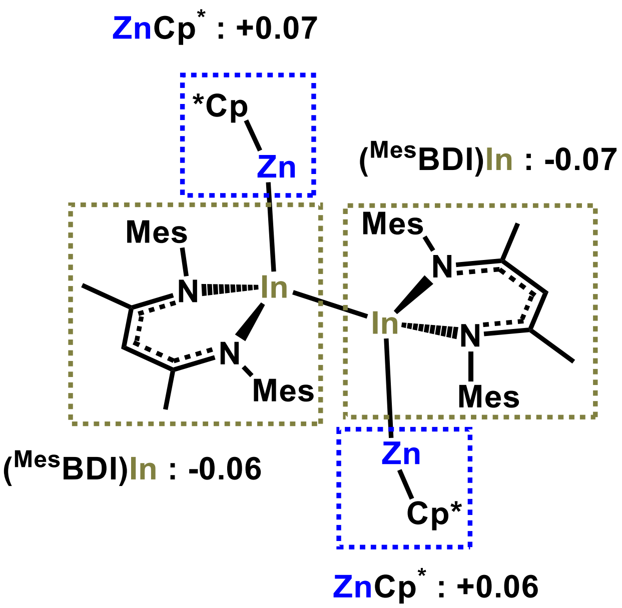

Figure S24. Calculated fragment charges in **3**. Units of charge are those from each fragment highlighted by a box.

6.4.4. NLMO Analysis

6.4.4.3. NLMO Analysis of 2a

**Si¹–Zn² σ NLMO**<br><br>**NLMO: 71.6 % Si¹, 28.4 % Zn²**	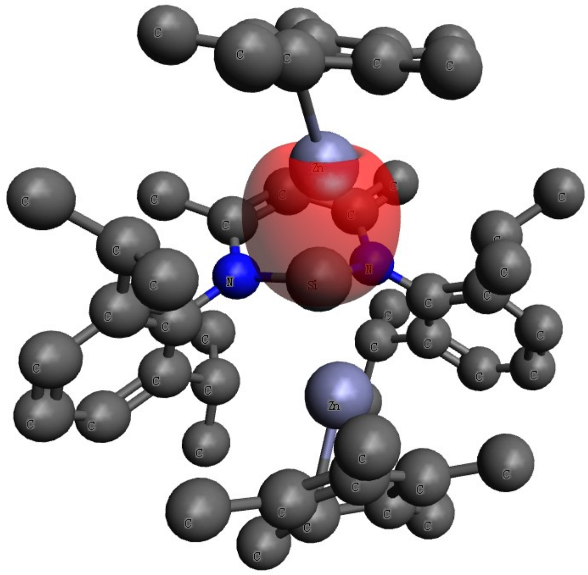
**Si¹–Zn³ σ NLMO**<br><br>**NLMO: 73.5 % Si¹, 26.5 % Zn³**	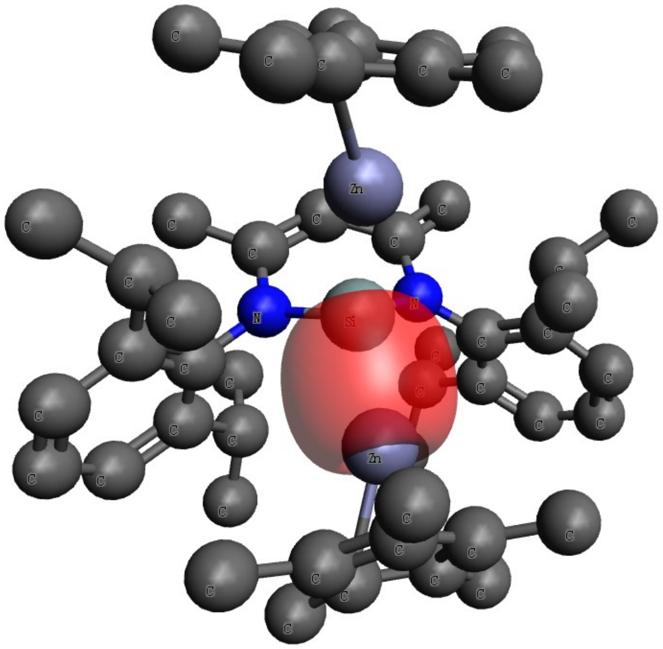

**Bond**	**Hybridisation (NLMO)**	
**σ (Si1 – Zn2)**	0.8463*Si1: $s^{0.38}p^{0.62}$	0.5328*Zn1: $s^{0.98}p^{0.01}d^{0.01}$
**σ (Si1 – Zn3)**	0.8575*Si1: $s^{0.39}p^{0.61}$	0.5145*Zn2: $s^{0.98}p^{0.01}d^{0.01}$

Table S72. Analysis of AO contributions to NLMOs of **2a**.

6.4.4.1. NLMO Analysis of 2b

**Al^{1}–Zn^{2} σ NLMO**<br><br>**NLMO: 58.4 % Al^{1}, 41.6 % Zn^{2}**	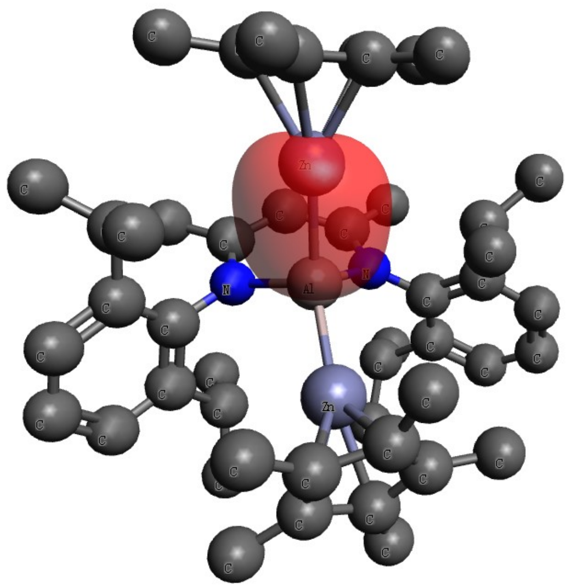
**Al^{1}–Zn^{3} σ NLMO**<br><br>**NLMO: 60.4 % Al^{1}, 39.5% Zn^{3}**	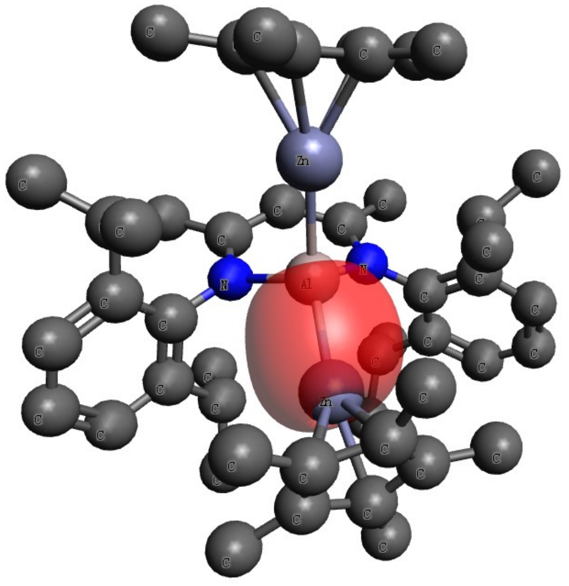

**Bond**	**Hybridisation (NLMO)**	
**σ (Al1 - Zn2)**	0.7639*Al1: $s^{0.39}p^{0.61}$	0.6453*Zn2: $s^{0.98}p^{0.01}d^{0.01}$
**σ (Al1 - Zn3)**	0.7769*Al1: $s^{0.44}p^{0.51}$	0.6296*Zn3: $s^{0.98}p^{0.01}d^{0.01}$

Table S73. Analysis of AO contributions to NLMOs of **2b**.

6.4.4.2. NLMO Analysis of 2c

**Ga^{125}–Zn^{1} σ NLMO**<br><br>**NLMO: 64.1 % Ga^{125}, 35.9 % Zn^{1}**	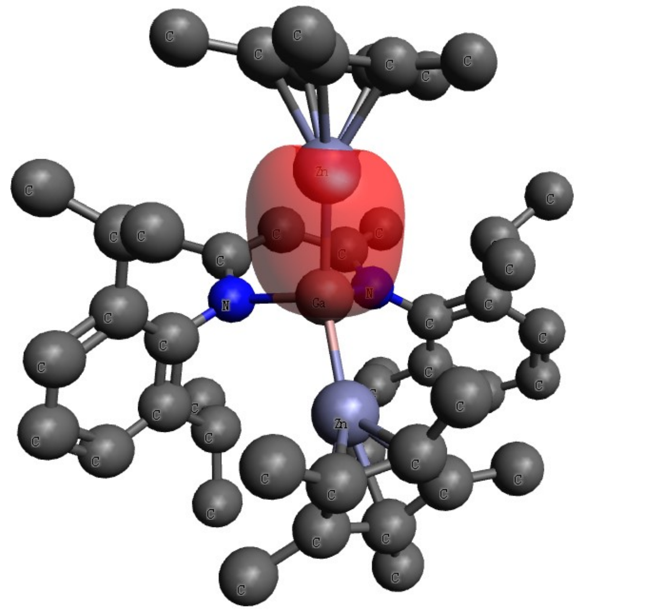
**Ga^{125}–Zn^{2} σ NLMO**<br><br>**NLMO: 65.4 % Ga^{125}, 34.6 % Zn^{2}**	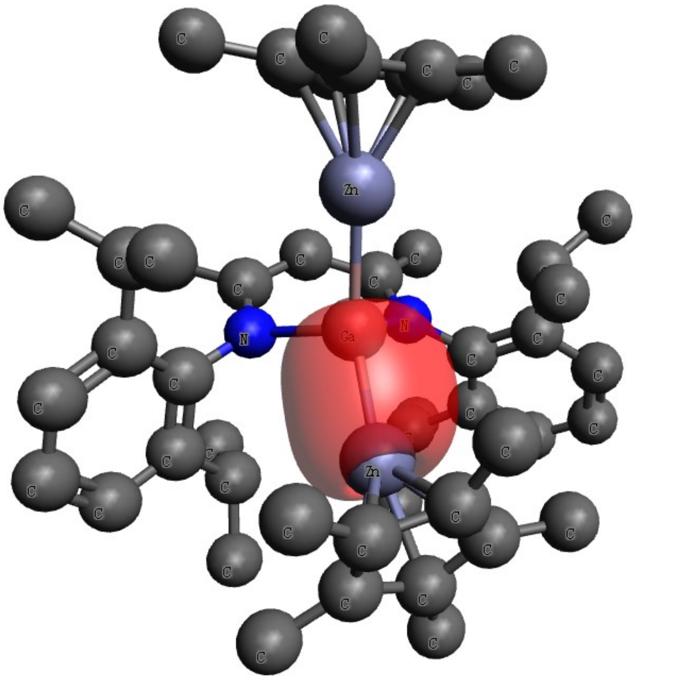

Bond	Hybridisation (NLMO)	
**σ (Ga125 – Zn1)**	0.8005*Ga125: $s^{0.42}p^{0.58}$	0.5993*Zn1: $s^{0.98}p^{0.01}d^{0.01}$
**σ (Ga125 – Zn2)**	0.8085*Ga125: $s^{0.47}p^{0.53}$	0.5886*Zn2: $s^{0.98}p^{0.01}d^{0.01}$

Table S74. Analysis of AO contributions to NLMOs of **2c**.

6.4.4.3. NLMO Analysis of 2d

**In^{107}–Zn^{1} σ NLMO**<br><br>**NLMO: 58.7 % In^{107}, 41.3 % Zn^{1}**	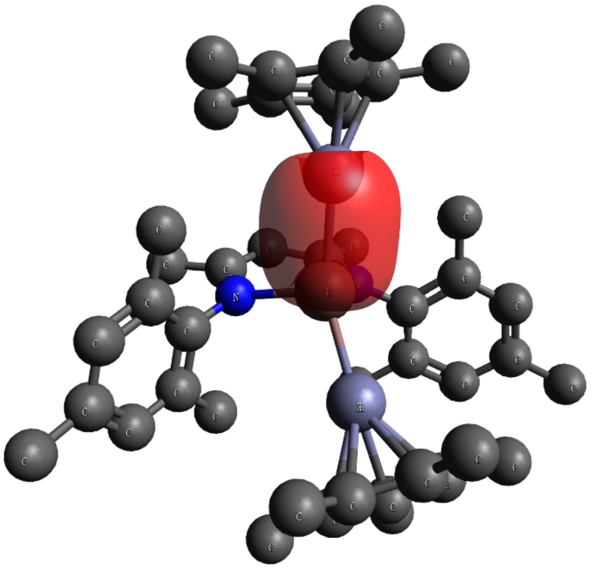
**In^{107}–Zn^{2} σ NLMO**<br><br>**NLMO: 60.3 % In^{107}, 39.8 % Zn^{2}**	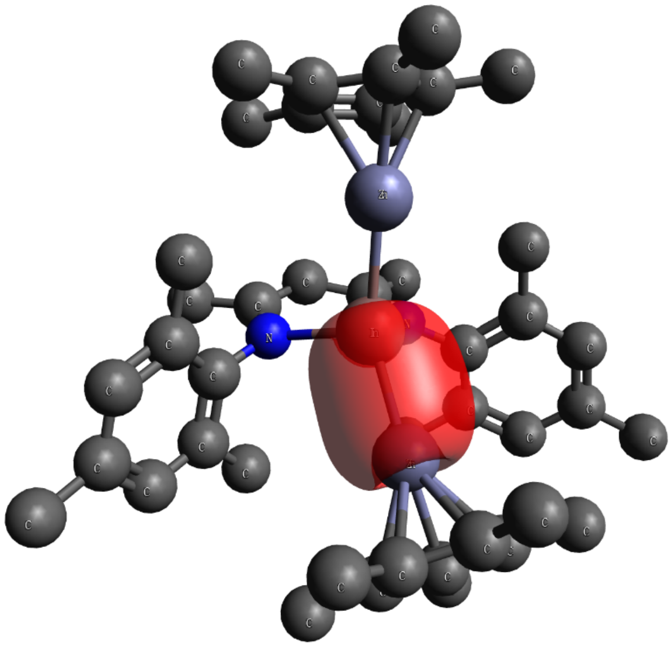

Bond	Hybridisation (NLMO)	
**σ (In107 – Zn1)**	0.7664*In107: $s^{0.44}p^{0.56}$	0.6424*Zn1: $s^{0.98}p^{0.01}d^{0.01}$
**σ (In107 – Zn2)**	0.7762* In107: $s^{0.47}p^{0.53}$	0.6305*Zn2: $s^{0.98}p^{0.01}d^{0.01}$

Table S75. Analysis of AO contributions to NLMOs of **2d**.

6.4.4.3. NLMO Analysis of 3

**In[1]–Zn[2] σ NLMO**<br><br>**NLMO: 49.1 % In[1], 50.9 % Zn[2]**	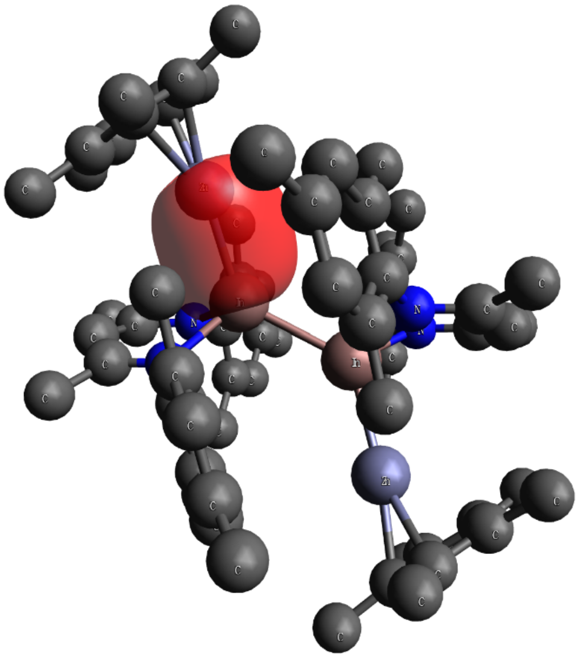
**In[1]–In[82] σ NLMO**<br><br>**NLMO: 49.8 % In[1], 50.2 % In[82]**	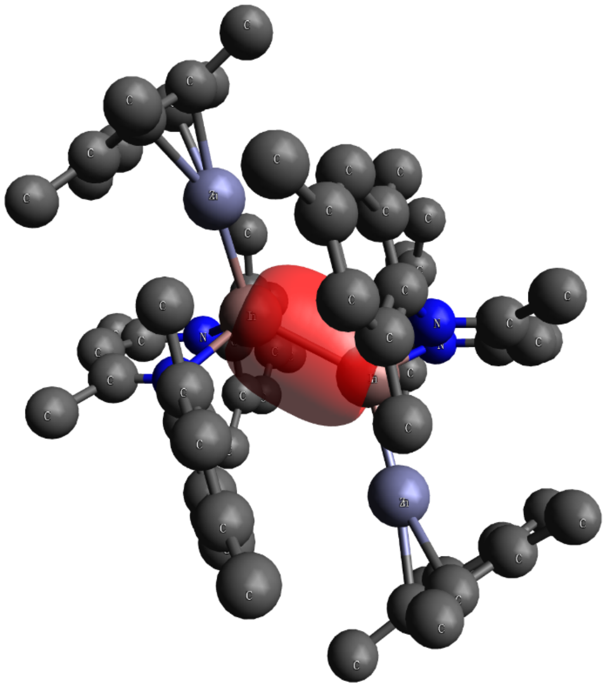

**In^{82}–Zn^{83} σ NLMO**<br><br>**NLMO: 49.0 % In^{82}, 51.0 % Zn^{83}**	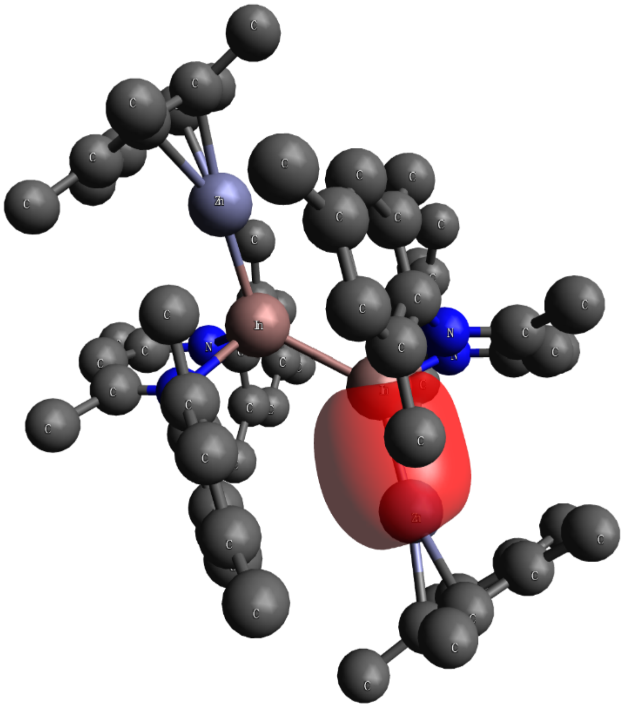

Bond	Hybridisation (NLMO)	
**σ (In1 – Zn2)**	0.7006*In1: $s^{0.42}p^{0.58}$	0.7136*Zn1: $s^{0.98}p^{0.01}d^{0.01}$
**σ (In1 – In82)**	0.7095*In1: $s^{0.50}p^{0.50}$	0.7083*In1: $s^{0.51}p^{0.49}$
**σ (In82 – Zn83)**	0.7000* In82: $s^{0.41}p^{0.59}$	0.7141*Zn83: $s^{0.98}p^{0.01}d^{0.01}$

Table S76. Analysis of AO contributions to NLMOs of **3.**

6.5. QTAIM Analysis

QTAIM analysis was performed using input files generated with the B3PW91-D3/def2tzvpp or BP86-D3/ def2tzvpp level for all atoms and solvent corrections (PCM, benzene, ε = 2.2706) or (PCM, tetrahydrofuran, ε = 7.4257) and conducted using the AIMAll software. [S34-S35] Bond critical points shown in green, ring critical points shown in red. The Laplacian of electron density value below 10^{-5} not be shown. Bond paths terminating at a critical point with an electron density which below 0.025 will be shown as dashed (instead of solid). Bond critical points with the Laplacian of electron density above 0.025 will be shown as solid lines.

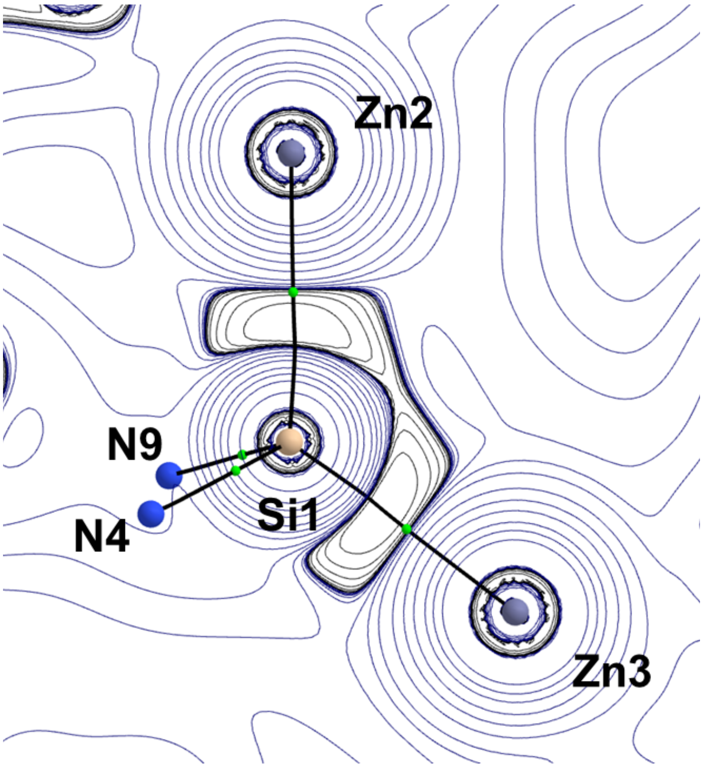

Figure S25. Plot of the Laplacian of the electron density $\nabla^2\rho$ (r) for **2a**

Atoms	ρ (r) (e bohr^{-3})	∇^2 ρ (r) (e bohr^{-5})	Ellipticity
Si1 - Zn3	0.084	-0.022	0.011
Si1 - Zn2	0.078	-0.023	0.023
Si1 - N9	0.120	0.449	0.153
Si1 - N4	0.117	0.433	0.146

Table S77. QTAIM parameters at bond critical points for **2a**

Atom	No	Charge
Si1	1	0.16
Zn2	2	0.89
Zn3	3	0.90

Table S78. AIM charges for **2a**

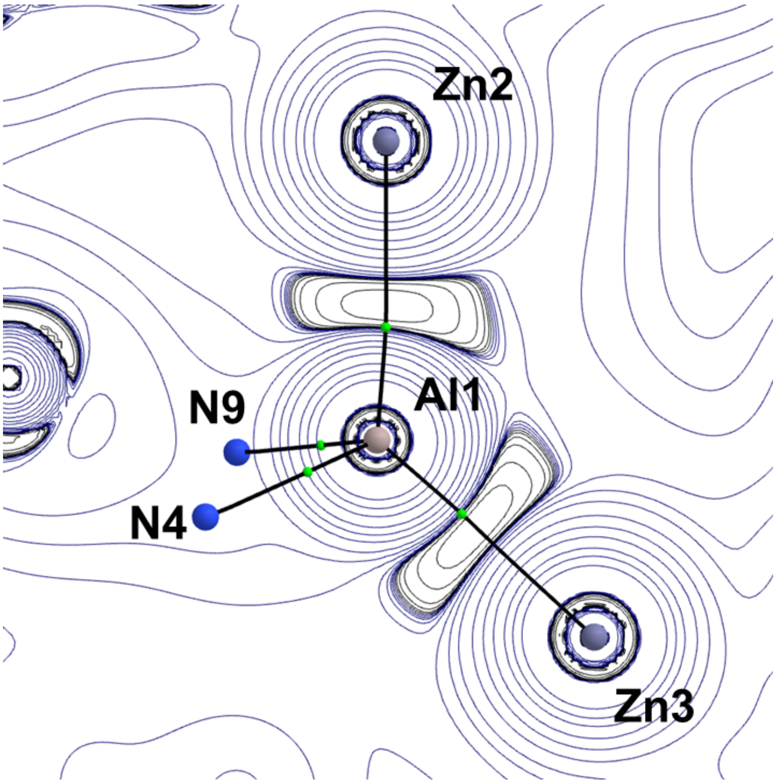

Figure S26. Plot of the Laplacian of the electron density $\nabla^2\rho$ (r) for **2b**

Atoms	ρ (r) (e bohr^{-3})	$\nabla^2\rho$ (r) (e bohr^{-5})	Ellipticity
Al1 - N4	0.070	0.332	0.108
Al1 - N9	0.067	0.315	0.103
Al1 - Zn2	0.053	-0.012	0.035
Al1 - Zn3	0.059	-0.009	0.022

Table S79. QTAIM parameters at bond critical points for **2b**

Atom	No	Charge
Al1	1	2.03
Zn2	2	0.03
Zn3	3	-0.01

Table S80. AIM charge data for **2b**

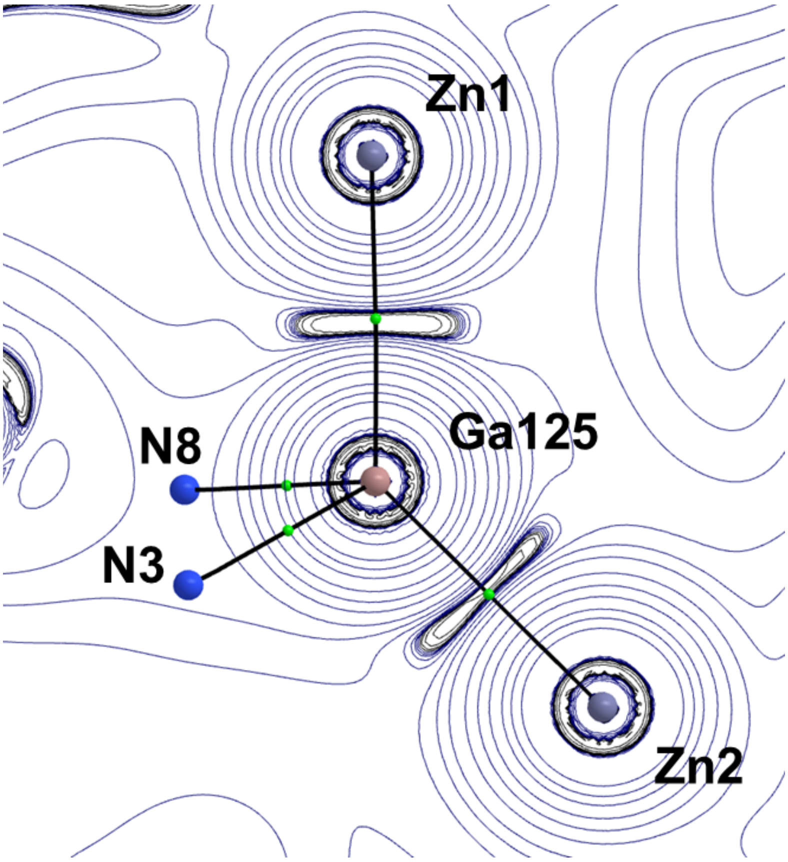

Figure S27. Plot of the Laplacian of the electron density $\nabla^2\rho$ (r) for **2c**

Atoms	ρ (r) (e bohr^{-3})	∇^2 ρ (r) (e bohr^{-5})	Ellipticity
Zn2 - Ga125	0.069	-0.006	0.008
Zn1 - Ga125	0.063	-0.015	0.017
N8 - Ga125	0.080	0.267	0.091
N3 - Ga125	0.084	0.287	0.096

Table S81. QTAIM parameters at bond critical points for **2c**

Atom	No	Charge
Ga125	125	0.80
Zn1	1	0.48
Zn2	2	0.49

Table S82. AIM charge data for **2c**

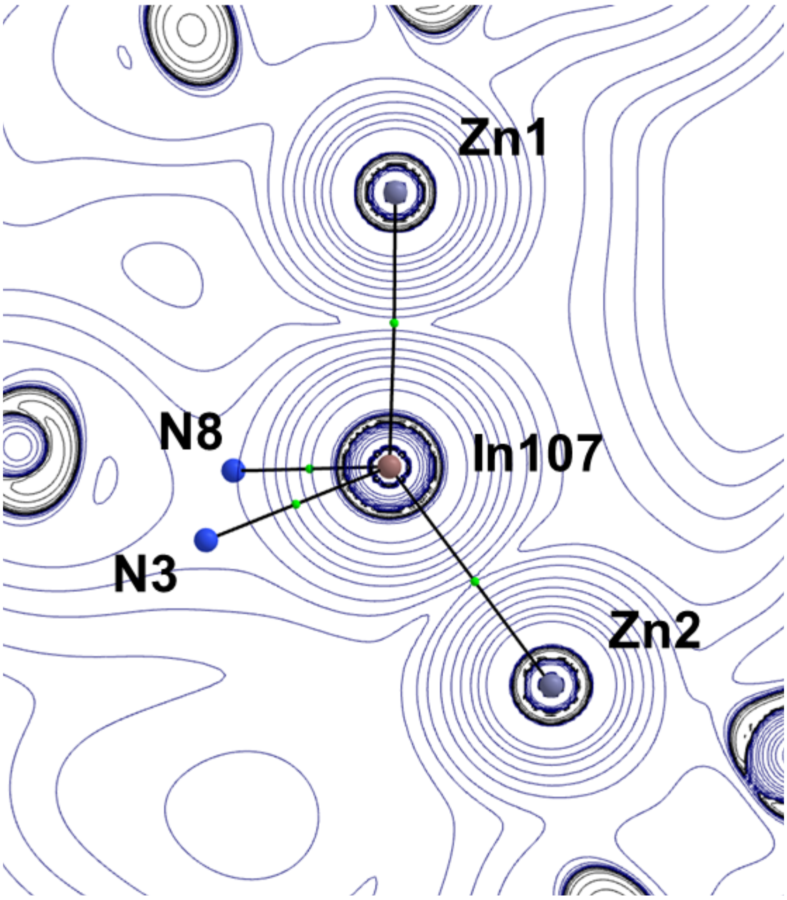

Figure S28. Plot of the Laplacian of the electron density $\nabla^2\rho$ (r) for **2d**

Atoms	ρ (r) (e bohr^{-3})	∇^2 ρ (r) (e bohr^{-5})	Ellipticity
In107 – Zn1	0.059	0.030	0.015
In107 – Zn2	0.062	0.038	0.013
In107 – N3	0.059	0.030	0.015
In107 – N8	0.062	0.038	0.013

Table S83. QTAIM parameters at bond critical points for **2d**

Atom	No	Charge
In107	107	0.71
Zn1	2	0.45
Zn2	3	0.48

Table S84. AIM charge data for **2d**

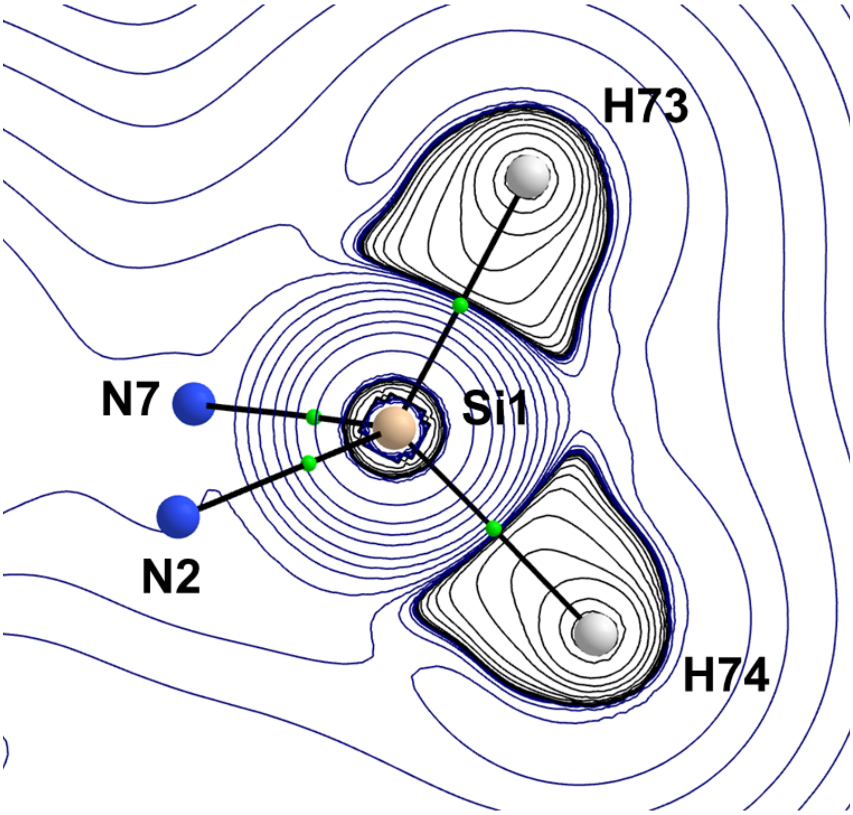

Figure S29. Plot of the Laplacian of the electron density $\nabla^2\rho$ (r) for **1a-H₂**

Atoms	ρ (r) (e bohr^{-3})	∇^2 ρ (r) (e bohr^{-5})	Ellipticity
Si1 - N7	0.131	0.530	0.191
Si1 - N2	0.129	0.505	0.170
Si1 - H74	0.125	0.155	0.011
Si1 - H73	0.124	0.149	0.009

Table S85. QTAIM parameters at bond critical points for **1a-H_2**

Atom	No	Charge
Si1	1	2.93
H73	73	-0.68
H74	74	-0.68

Table S86. AIM charge data for **1a-H_2**

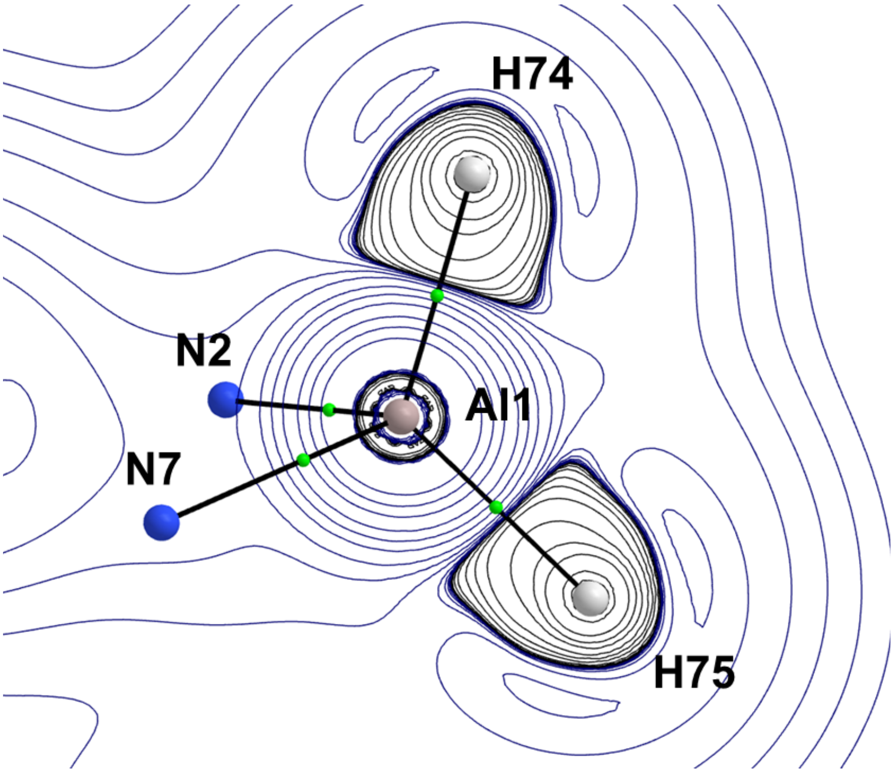

Figure S30. Plot of the Laplacian of the electron density $\nabla^2\rho$ (r) for **1b-H₂**

Atoms	ρ (r) (e bohr^{-3})	∇^2 ρ (r) (e bohr^{-5})	Ellipticity
Al1 - H74	0.079	0.209	0.001
Al1 - H75	0.080	0.212	0.004
Al1 - N2	0.071	0.343	0.097
Al1 - N7	0.072	0.347	0.098

Table S87. QTAIM parameters at bond critical points for **1b-H_2**

Atom	No	Charge
Al1	1	2.34
H74	74	-0.75
H75	75	-0.75

Table S88. AIM change data for **1b-H_2**

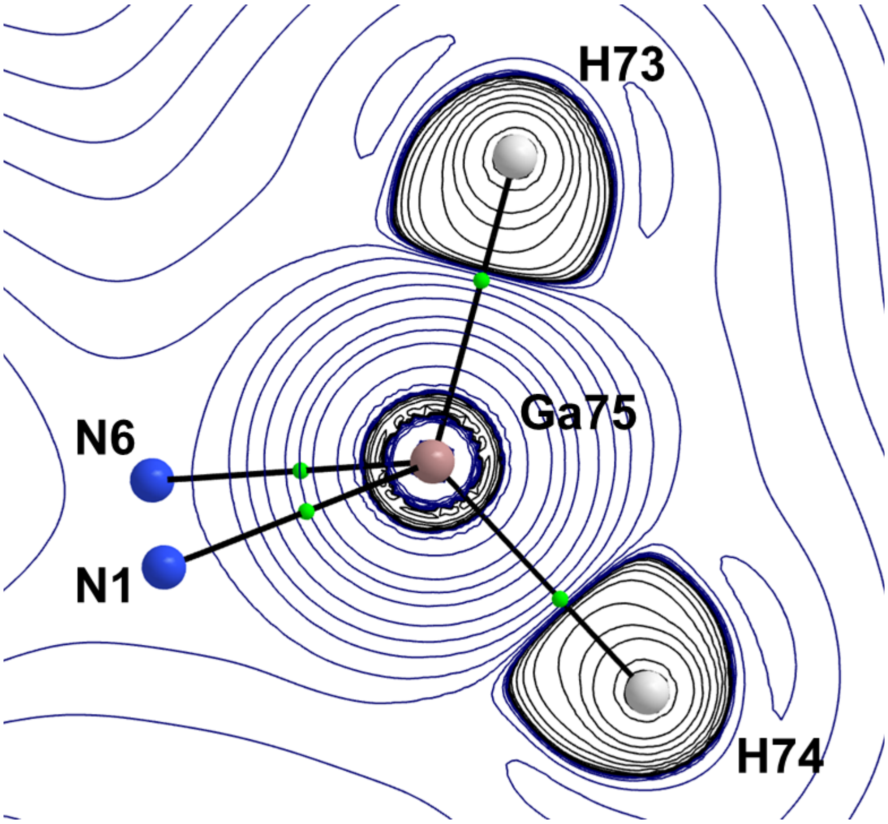

Figure S31. Plot of the Laplacian of the electron density $\nabla^2\rho$ (r) for **1c-H$_2$**

Atoms	ρ (r) (e bohr^{-3})	$\nabla^2\rho$ (r) (e bohr^{-5})	Ellipticity
N6 - Ga75	0.089	0.317	0.092
N1 - Ga75	0.088	0.312	0.092
H74 - Ga75	0.112	0.151	0.009
H73 - Ga75	0.111	0.150	0.010

Table S89. QTAIM parameters at bond critical points for **1c-H$_2$**

Atom	No	Charge
Ga75	75	1.47
H73	73	-0.44
H74	74	-0.44

Table S90. AIM change data for **1c-H$_2$**

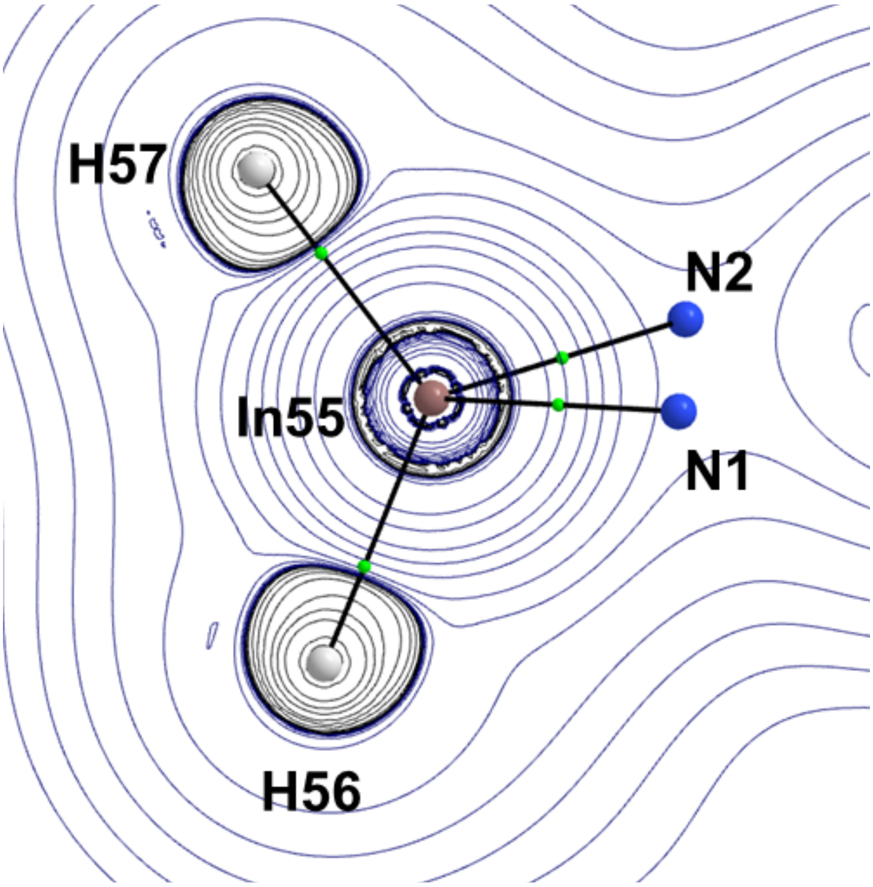

Figure S32. Plot of the Laplacian of the electron density $\nabla^2\rho$ (r) for **1d-H₂**

**Atoms**	**ρ (r) (e bohr^{-3})**	**$\nabla^2\rho$ (r) (e bohr^{-5})**	**Ellipticity**
In55 - H56	0.098	0.112	0.013
In55 - H57	0.098	0.112	0.013
In55 - N1	0.087	0.285	0.098
In55 - N2	0.087	0.285	0.098

Table S91. QTAIM parameters at bond critical points for **1d-H_2**

**Atom**	**No**	**Charge**
In55	55	1.24
H56	56	-0.37
H57	57	-0.37

Table S92. AIM change data for **1d-H_2**

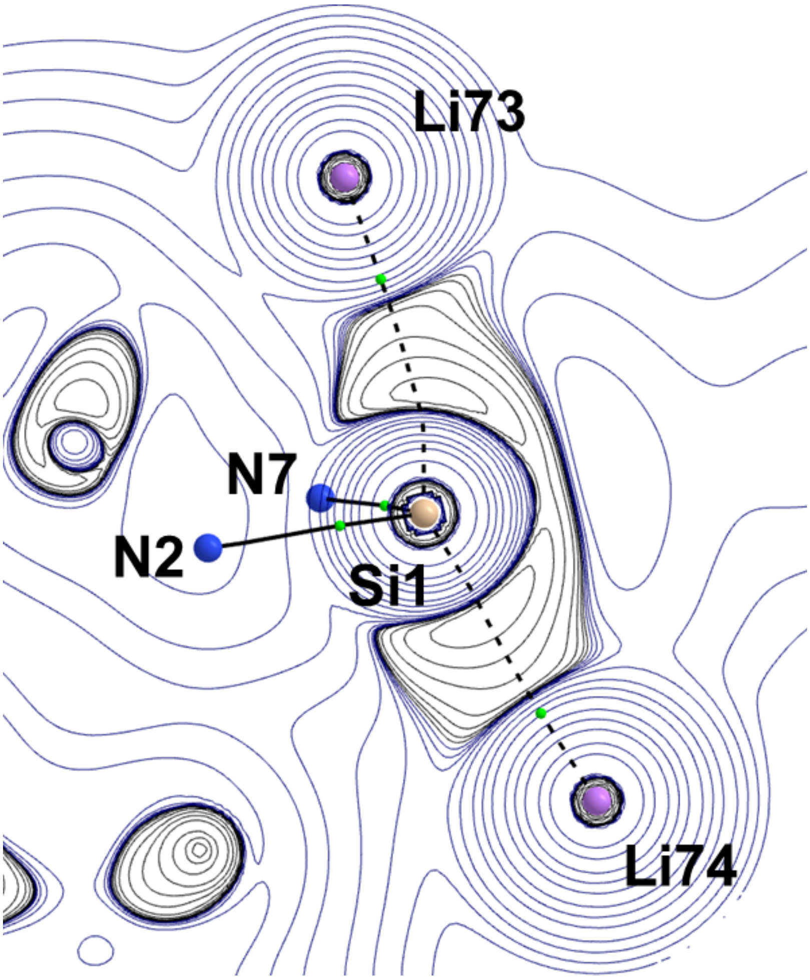

Figure S33. Plot of the Laplacian of the electron density $\nabla^2\rho$ (r) for **1a-Li₂**

Atoms	ρ (r) (e bohr^{-3})	$\nabla^2\rho$ (r) (e bohr^{-5})	Ellipticity
Si1 - N7	0.097	0.311	0.188
Si1 - N2	0.093	0.287	0.250
Si1 - Li74	0.025	0.068	0.025
Si1 - Li73	0.020	0.054	0.127

Table S93. QTAIM parameters at bond critical points for **1a-Li$_2$**

Atom	No	Charge
Si1	1	0.16
Li73	73	0.89
Li74	74	0.90

Table S94. AIM change data for **1a-Li$_2$**

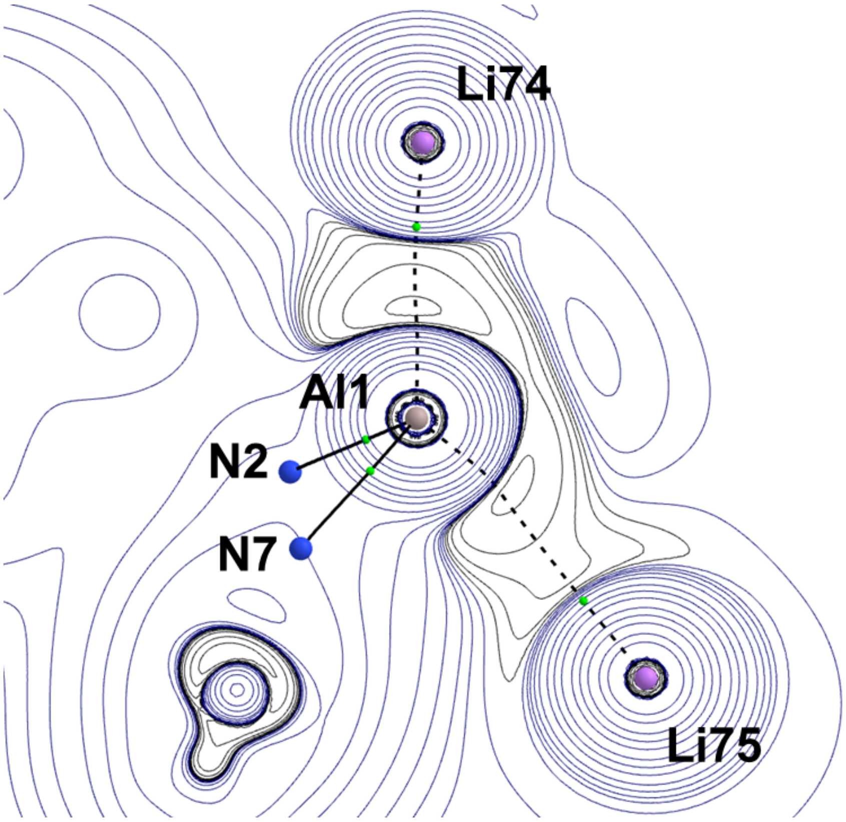

Figure S34. Plot of the Laplacian of the electron density $\nabla^2\rho$ (r) for **1b-Li₂**

Atoms	ρ (r) (e bohr^{-3})	$\nabla^2\rho$ (r) (e bohr^{-5})	Ellipticity
Al1 - Li74	0.019	0.040	0.044
Al1 - Li75	0.011	0.011	0.017
Al1 - N2	0.057	0.240	0.125
Al1 - N7	0.057	0.240	0.126

Table S95. QTAIM parameters at bond critical points for **1b-Li$_2$**

Atom	No	Charge
Al1	1	-0.19
Li74	74	0.86
Li75	75	0.64

Table S96. AIM change data for **1b-Li$_2$**

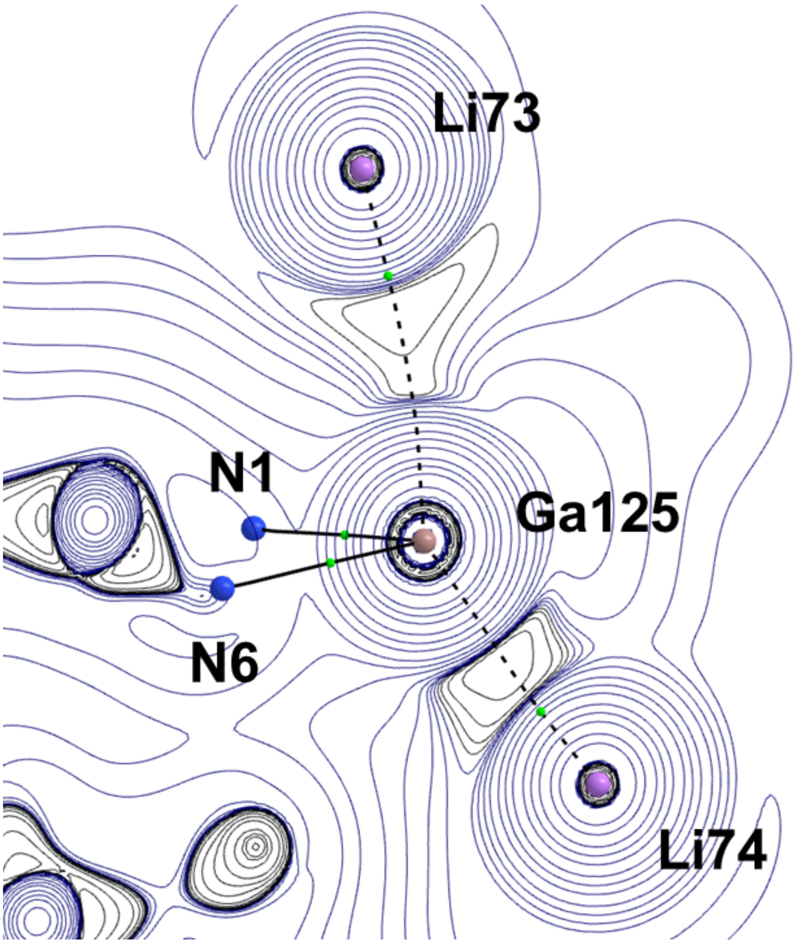

Figure S35. Plot of the Laplacian of the electron density $\nabla^2\rho$ (r) for **1c-Li₂**

Atoms	ρ (r) (e bohr^{-3})	$\nabla^2\rho$ (r) (e bohr^{-5})	Ellipticity
N6 - Ga75	0.059	0.160	0.124
N1 - Ga75	0.060	0.162	0.132
Li74 - Ga75	0.010	0.010	0.012
Li73 - Ga75	0.018	0.040	0.031

Table S97. QTAIM parameters at bond critical points for **1c-Li$_2$**

Atom	No	Charge
Ga75	1	-0.50
Li73	73	0.85
Li74	74	0.72

Table S98. AIM charge data for **1c-Li$_2$**

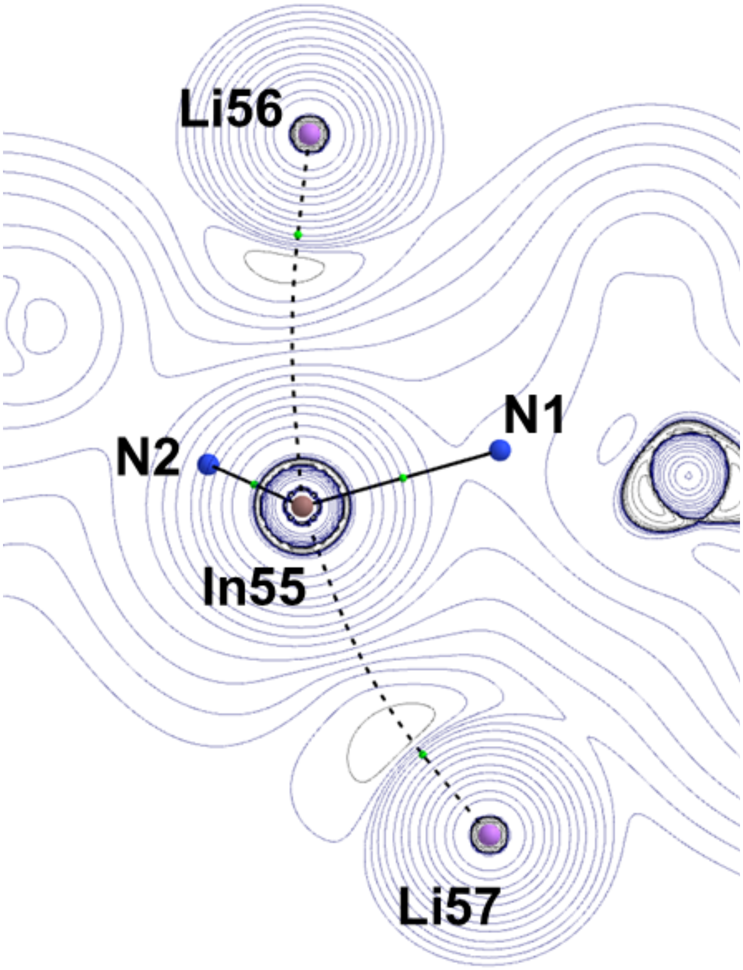

Figure S36. Plot of the Laplacian of the electron density $\nabla^2\rho$ (r) for **1d-Li$_2$**

Atoms	ρ (r) (e bohr^{-3})	$\nabla^2\rho$ (r) (e bohr^{-5})	Ellipticity
In55 - Li56	0.006	0.005	0.041
In55 - Li57	0.006	0.005	0.055
In55 - N1	0.045	0.123	0.115
In55 - N2	0.045	0.143	0.130

Table S99. QTAIM parameters at bond critical points for **1d-Li$_2$**

Atom	No	Charge
In55	55	-0.76
Li56	56	0.91
Li57	57	0.93

Table S100. AIM charge data for **1d-Li$_2$**

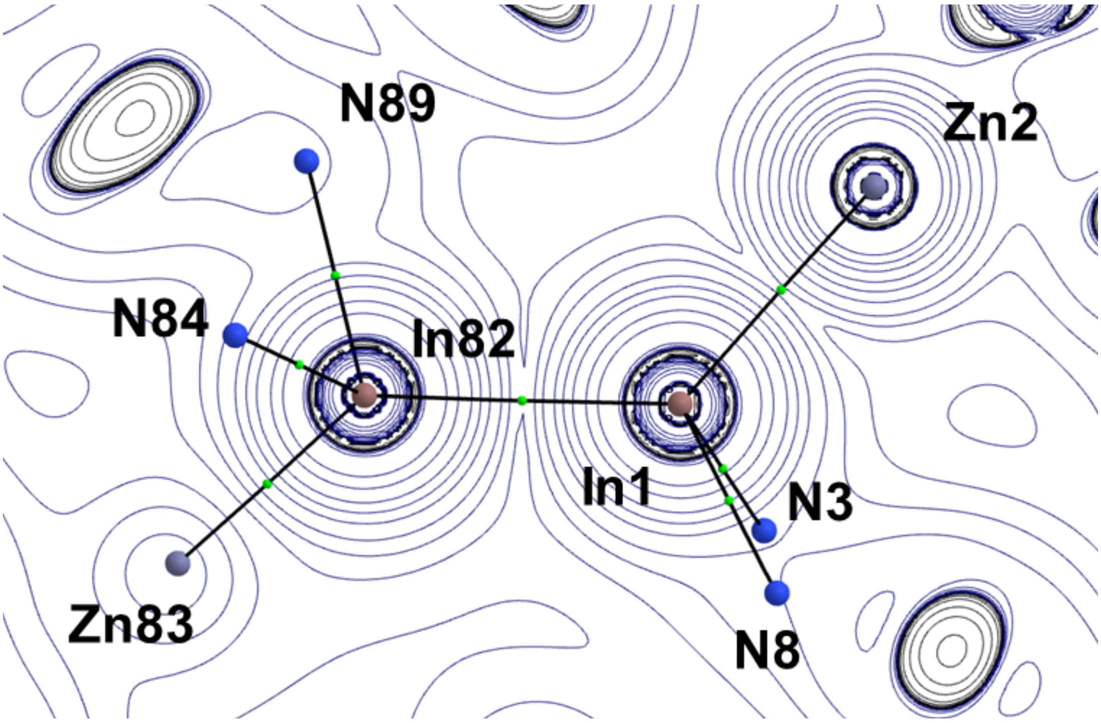

Figure S37. Plot of the Laplacian of the electron density $\nabla^2\rho$ (r) for **3**

Atoms	ρ (r) (e bohr^{-3})	$\nabla^2\rho$ (r) (e bohr^{-5})	Ellipticity
In82 – Zn83	0.060	0.035	0.016
In82 – N89	0.079	0.252	0.088
In82 – N84	0.082	0.265	0.089
In1 – Zn2	0.060	0.035	0.016
In1 – N8	0.082	0.265	0.089
In1 – N3	0.079	0.254	0.088
In1 – In82	0.056	0.020	0.011

Table S101. QTAIM parameters at bond critical points for **3**

Atom	No	Charge
In1	1	0.69
In82	82	0.69
Zn2	2	0.43
Zn83	83	0.43

Table S102. AIM charge data for **3**

6.6. ETS-NOCV

ETS-NOCV calculations were performed on complexes **2a**, **2b** and **2c** to give insight into bonding. Three sets of calculations with different fragment splitting were conducted.

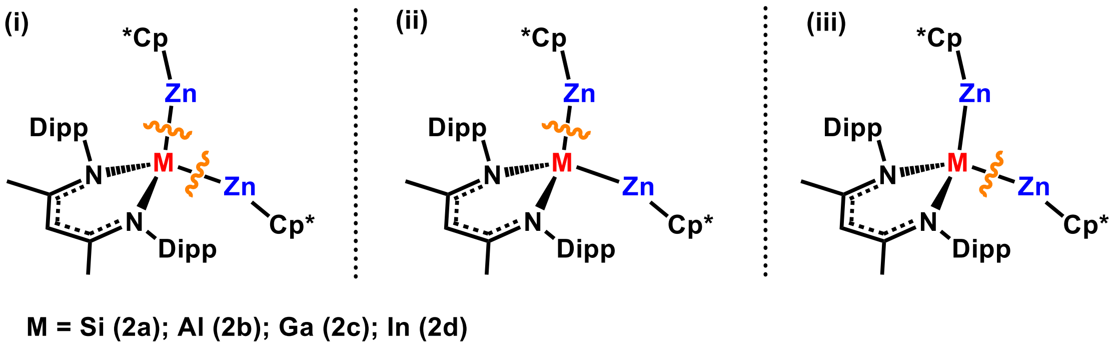

Figure S38. Splitting of fragments for ETS-NOCV calculations for complexes **2a**, **2b** and **2c**.

Splitting	ΔE_{ORB}	$\Delta\rho1$	$\Delta\rho2$
2a (i)	-315.3	-144.6 (46%)	-93.2 (30%)
2b (i)	-372.0	-216.7 (58%)	-99.6 (27%)
2c (i)	-354.9	-213.9 (60%)	-86.3 (24%)
2d (i)	-367.4	-188.7 (51%)	-129.4 (35%)
2a (ii)	-119.7	-85.4 (71%)	-4.9 (4%)
2b (ii)	-145.1	-117.4 (81%)	-4.3 (3%)
2c (ii)	-129.6	-101.2 (78%)	-3.8 (3%)
2d (ii)	-130.0	-105.4 (81%)	-3.0 (3%)
2a (iii)	-103.0	-68.9 (67%)	-4.4 (4%)
2b (iii)	-117.2	-88.4 (75%)	-3.2 (3%)
2c (iii)	-111.5	-83.1 (75%)	-3.1 (3%)
2d (iii)	-116.1	-91.9 (79%)	-3.0 (3%)

Table S103. Summary of ETS-NOCV data for complexes **2a-d**. All values in kcal mol^{-1}. Percentage contribution of $\Delta\rho x$ to ΔE_{ORB} shown in brackets.

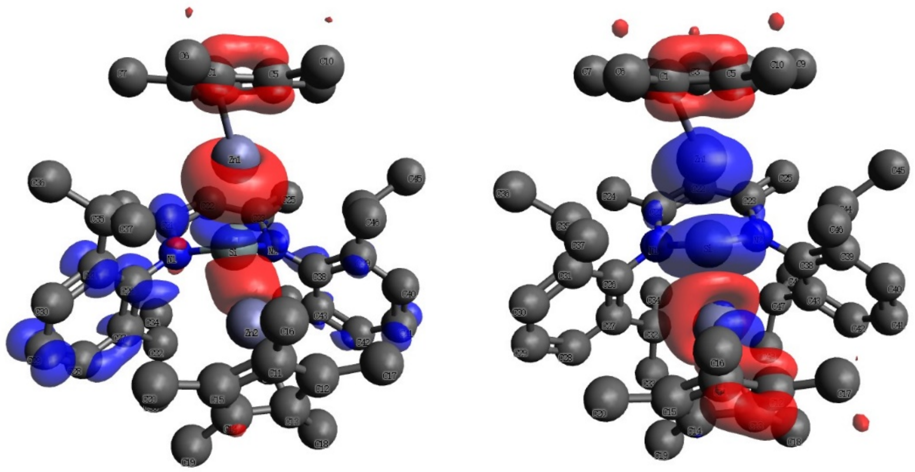

Δρ1 **Δρ2**

-144.6 kcal mol^{-1} **-93.2 kcal mol^{-1}**

Figure S39. Selected deformation density data for complex **2a** with splitting (i). Charge flow is from blue to red. All hydrogen atoms omitted for clarity.

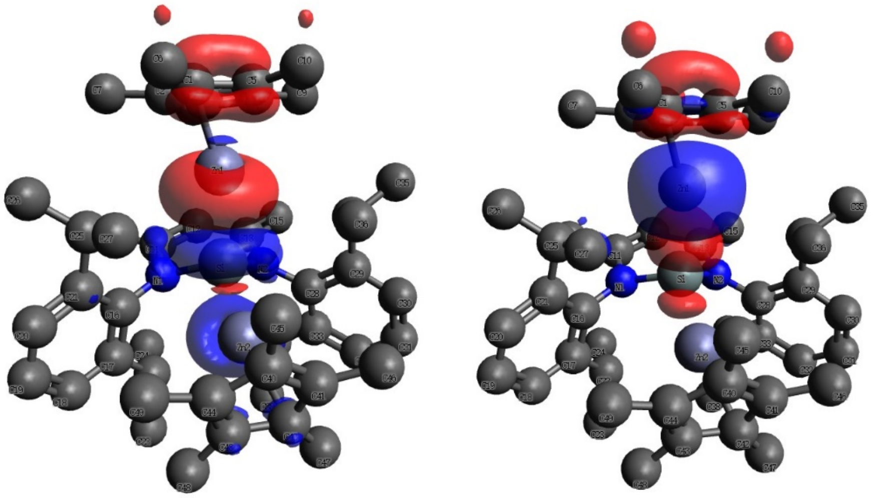

Δρ1 **Δρ2**

-85.4 kcal mol^{-1} **-4.9 kcal mol^{-1}**

Figure S40. Selected deformation density data for complex **2a** with splitting (ii). Charge flow is from blue to red. All hydrogen atoms omitted for clarity.

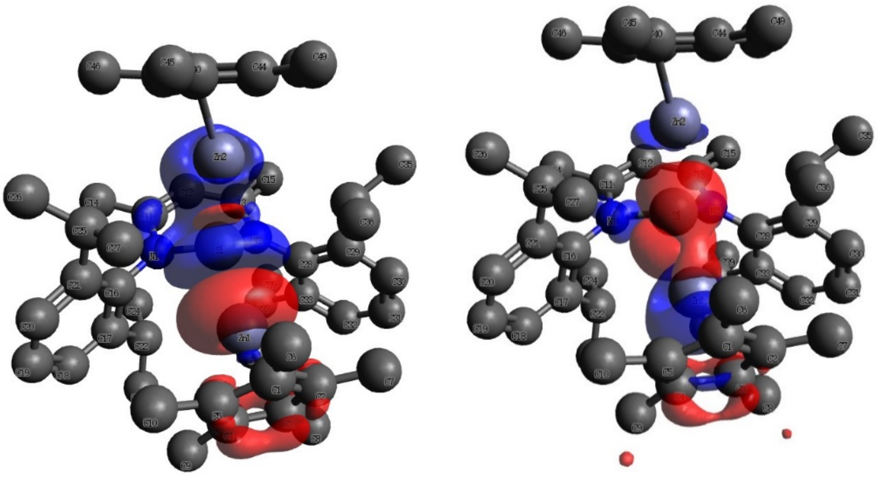

Δρ1 **Δρ2**

-68.9 kcal mol^{-1} **-4.4 kcal mol^{-1}**

Figure S41. Selected deformation density data for complex **2a** with splitting (iii). Charge flow is from blue to red. All hydrogen atoms omitted for clarity.

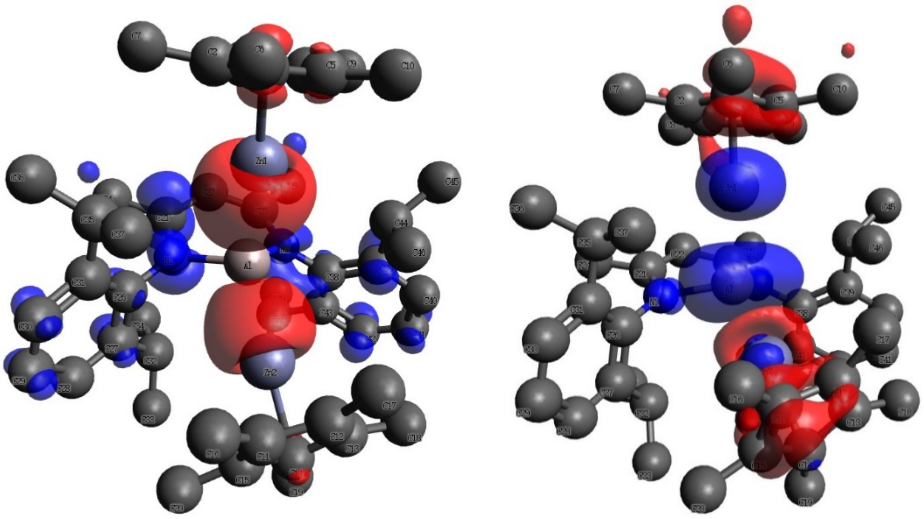

Δρ1 **Δρ2**

-216.7 kcal mol^{-1} **-99.6 kcal mol^{-1}**

Figure S42. Selected deformation density data for complex **2b** with splitting (i). Charge flow is from blue to red. All hydrogen atoms omitted for clarity.

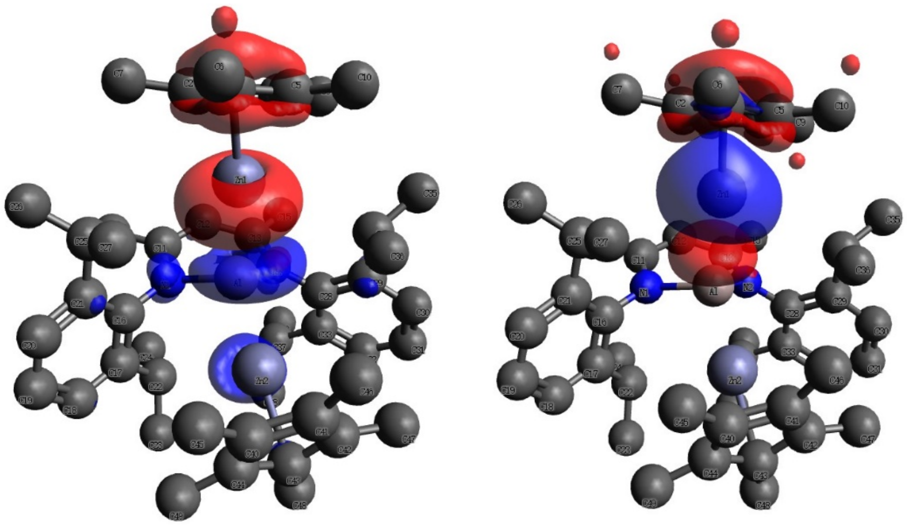

Δρ1 **-117.4 kcal mol^{-1}**

Δρ2 **-4.3 kcal mol^{-1}**

Figure S43. Selected deformation density data for complex **2b** with splitting (ii). Charge flow is from blue to red. All hydrogen atoms omitted for clarity.

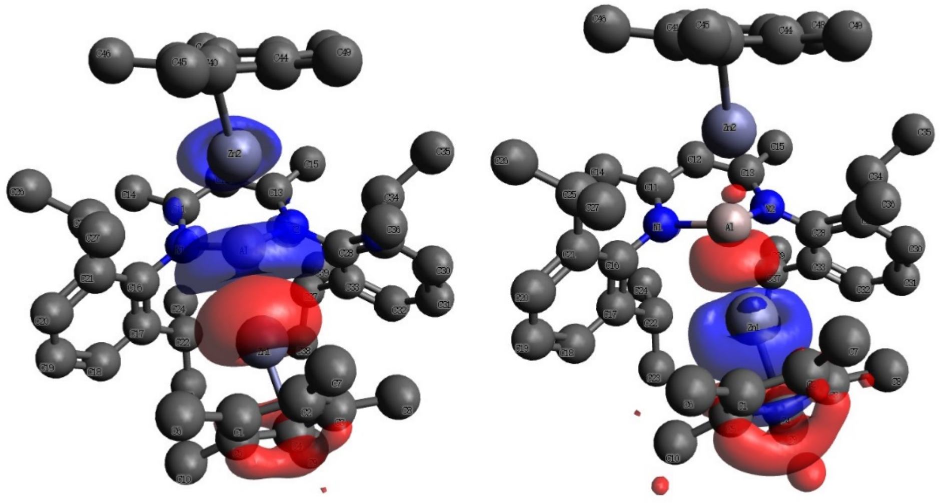

Δρ1 **-88.4 kcal mol^{-1}**

Δρ2 **-3.2 kcal mol^{-1}**

Figure S44. Selected deformation density data for complex **2b** with splitting (iii). Charge flow is from blue to red. All hydrogen atoms omitted for clarity.

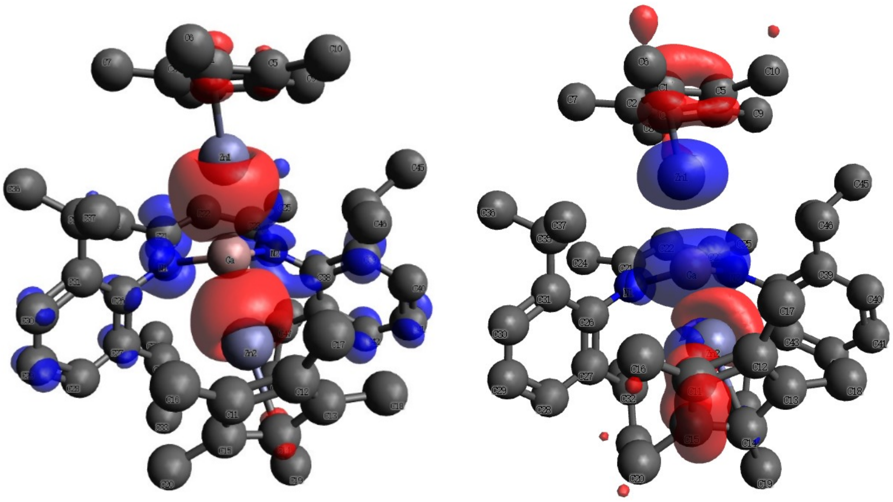

Δρ1 **-213.9 kcal mol^{-1}**

Δρ2 **-86.3 kcal mol^{-1}**

Figure S45. Selected deformation density data for complex **2c** with splitting (i). Charge flow is from blue to red. All hydrogen atoms omitted for clarity.

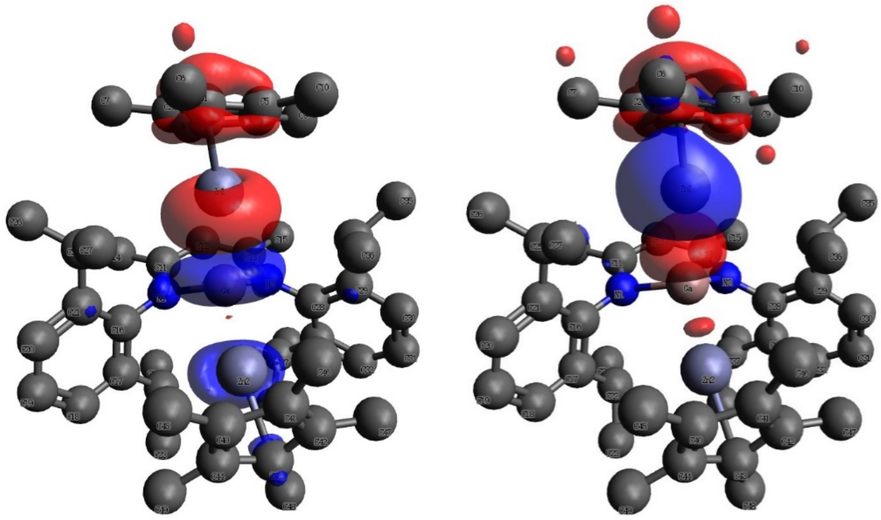

Δρ1 **-101.2 kcal mol^{-1}**

Δρ2 **-3.8 kcal mol^{-1}**

Figure S46. Selected deformation density data for complex **2c** with splitting (ii). Charge flow is from blue to red. All hydrogen atoms omitted for clarity.

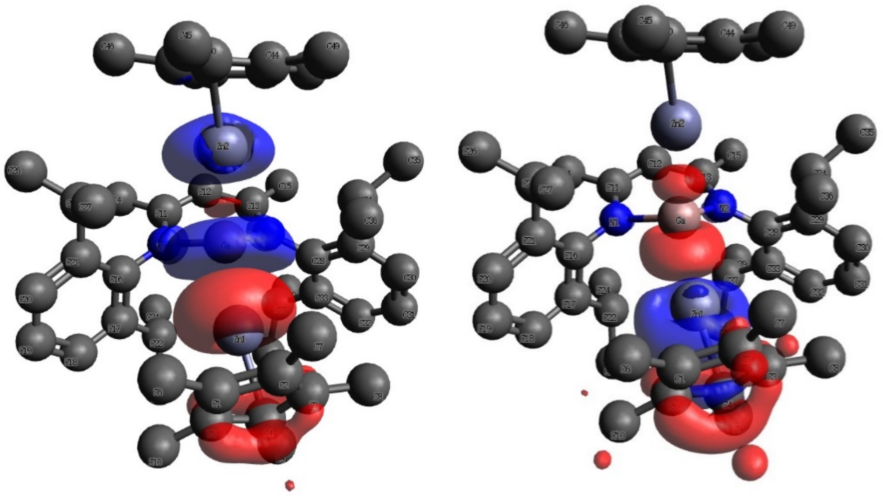

Δρ1 **Δρ2**

-83.1 kcal mol^{-1} **-3.1 kcal mol^{-1}**

Figure S47. Selected deformation density data for complex **2c** with splitting (iii). Charge flow is from blue to red. All hydrogen atoms omitted for clarity.

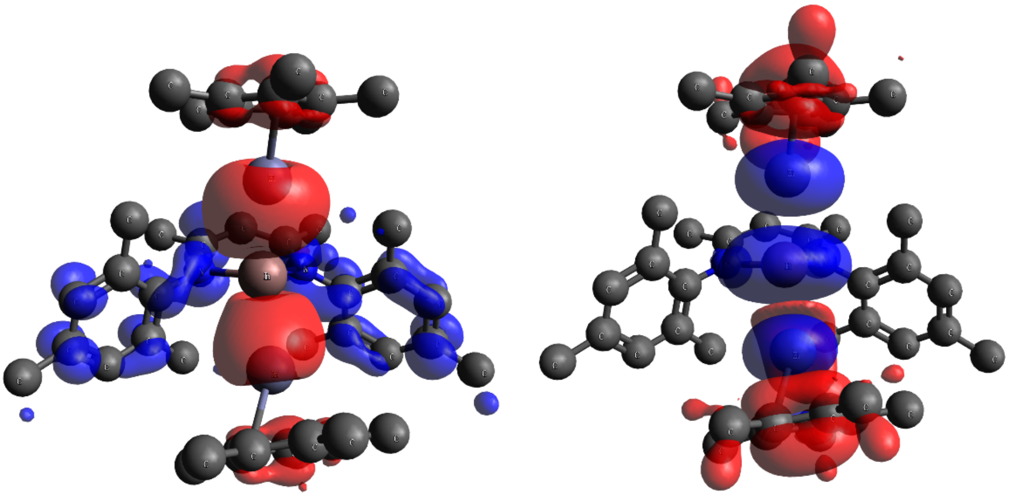

Δρ1 **Δρ2**

-188.7 kcal mol^{-1} **-129.4 kcal mol^{-1}**

Figure S48. Selected deformation density data for complex **2d** with splitting (i). Charge flow is from blue to red. All hydrogen atoms omitted for clarity.

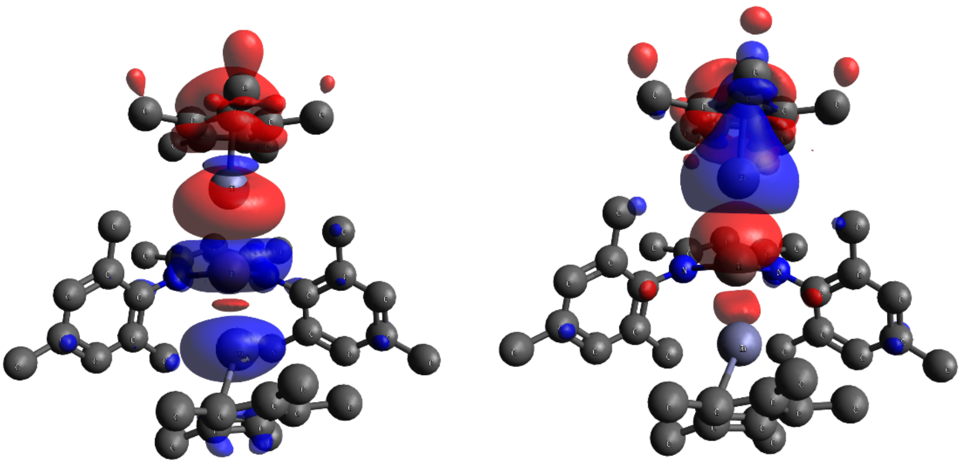

Δρ1 **Δρ2**

-105.4 kcal mol^{-1} **-3.0 kcal mol^{-1}**

Figure S49. Selected deformation density data for complex **2d** with splitting (ii). Charge flow is from blue to red. All hydrogen atoms omitted for clarity.

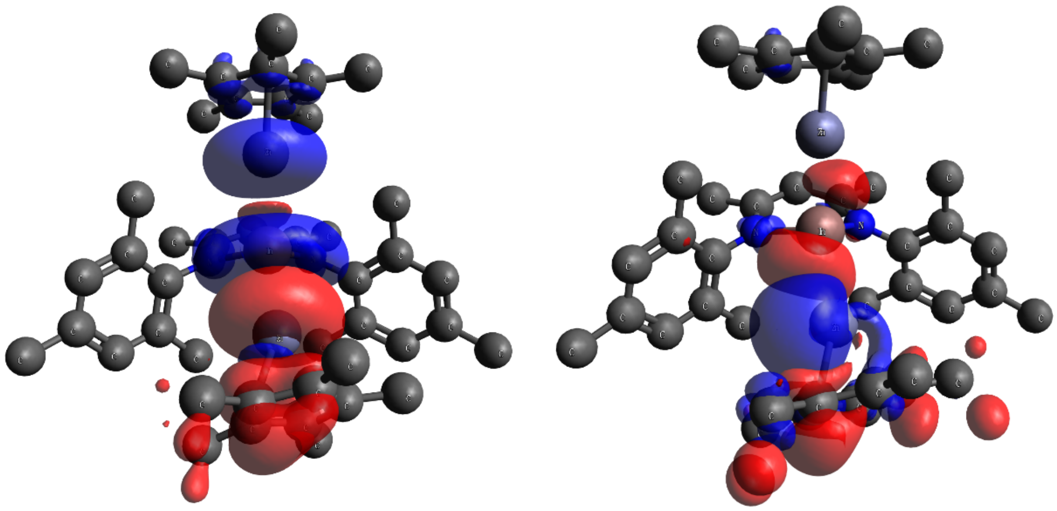

Δρ1 **Δρ2**

-91.9 kcal mol^{-1} **-3.0 kcal mol^{-1}**

Figure S50. Selected deformation density data for complex **2d** with splitting (iii). Charge flow is from blue to red. All hydrogen atoms omitted for clarity.

6.7. IBO Analysis

All IBOs below are visualised at the isovalue = 0.05.

6.7.1. IAO Charges

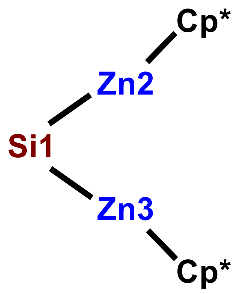

Atom	No	Charge (B3PW91-D3)
Si1	1	0.07
Zn2	2	1.17
Zn3	3	1.25

Table S104. IAO charge data for **2a**

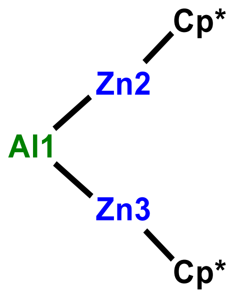

Atom	No	Charge (B3PW91-D3)
Al1	1	-0.25
Zn2	2	1.06
Zn3	3	1.14

Table S105. IAO charge data for **2b**

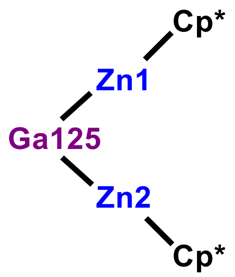

Atom	No	Charge (B3PW91-D3)
Ga125	125	-0.49
Zn1	1	1.15
Zn2	2	1.22

Table S106. IAO charge data for **2c**

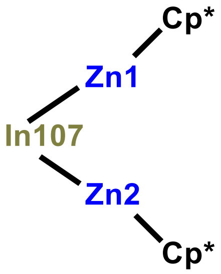

Atom	No	Charge (BP86-D3)
In107	107	1.55
Zn1	1	0.59
Zn2	2	0.63

Table S107. IAO charge data for **2d**

6.7.2. IAO Fragment Charges

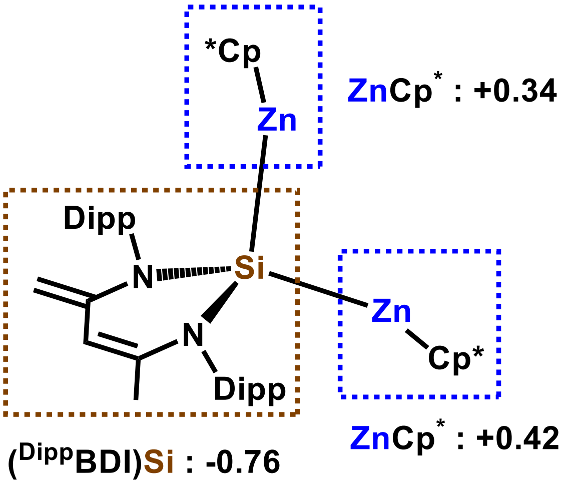

Figure S51. Calculated IAO Fragment Charges in **2a**. Units of charge are those from each fragment highlighted by a box.

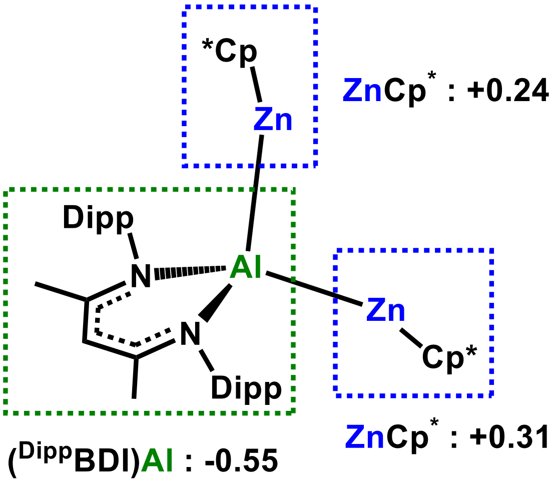

Figure S52. Calculated IAO Fragment Charges in **2b**. Units of charge are those from each fragment highlighted by a box.

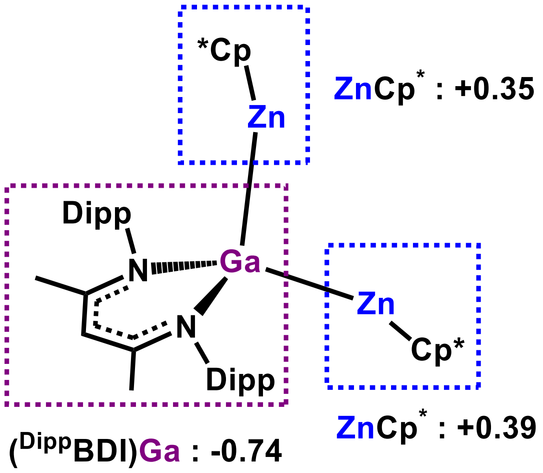

Figure S53. Calculated IAO Fragment Charges in **2c**. Units of charge are those from each fragment highlighted by a box.

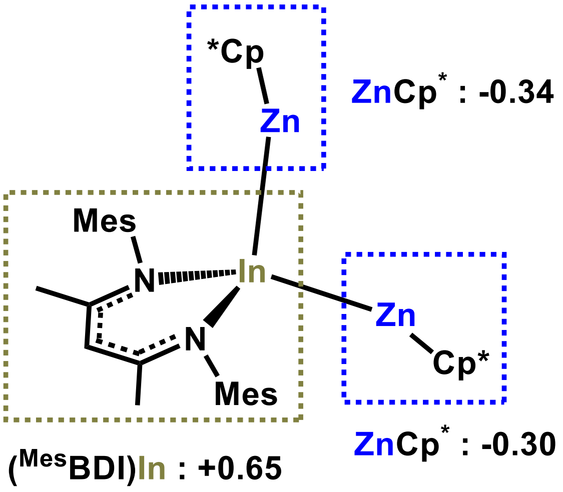

Figure S54. Calculated IAO Fragment Charges in **2d**. Units of charge are those from each fragment highlighted by a box.

6.7.3. IBO Analysis of 2a

IBO is consistent with NLMO analysis and again suggest all bonds are covalent with and that the Si analogue **2a** has the largest coefficients on the central atom across the series.

**Si^1–Zn^2 σ IBO**<br><br>**IBO: 36.4 % Si^1, 44.3 % Zn^2, 11.9 % Zn^3**	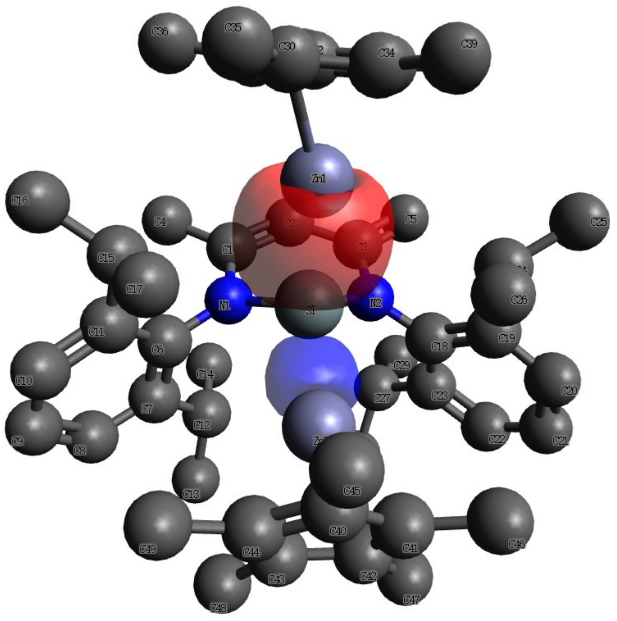
**Si^1–Zn^3 σ IBO**<br><br>**IBO: 65.7 % Si^1, 25.4% Zn^3**	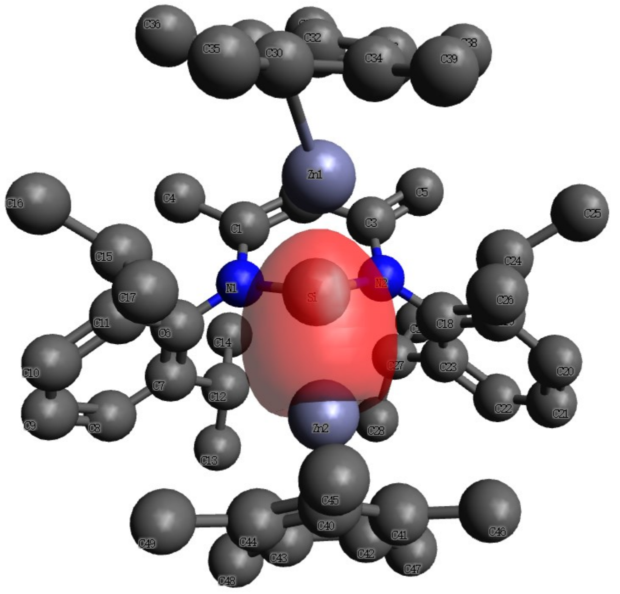

6.7.3. IBO Analysis of 2b

**Al¹–Zn² σ IBO**<br><br>**IBO: 33.3 % Al¹, 62.9 % Zn²**	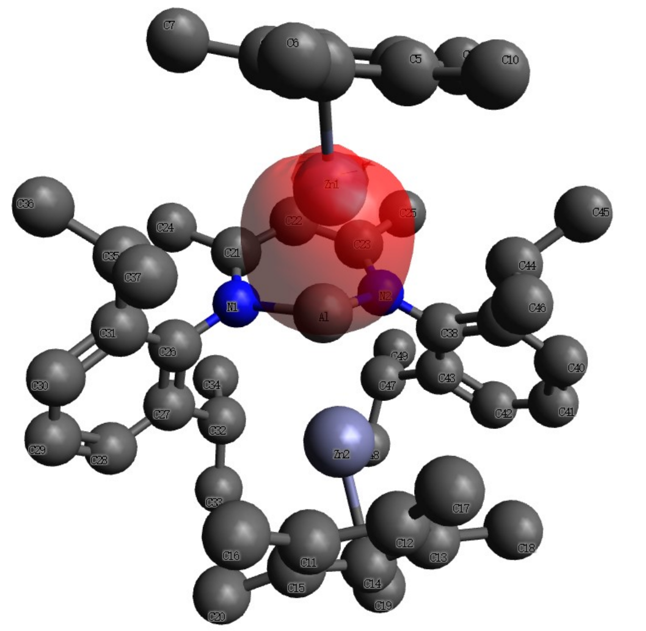
**Al¹–Zn³ σ IBO**<br><br>**IBO: 49.4 % Al¹, 52.2% Zn³**	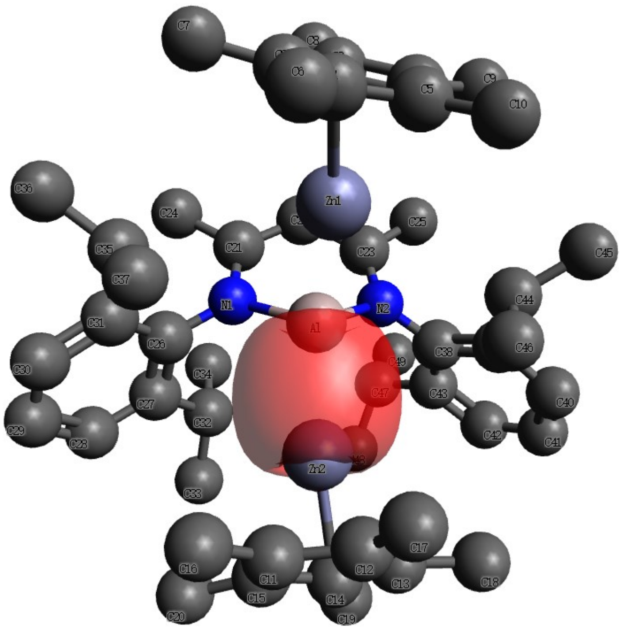

6.7.4. IBO Analysis of 2c

**Ga[125]–Zn[1] σ IBO**<br><br>**IBO: 33.3 % Ga[125], 59.5 % Zn[1]**	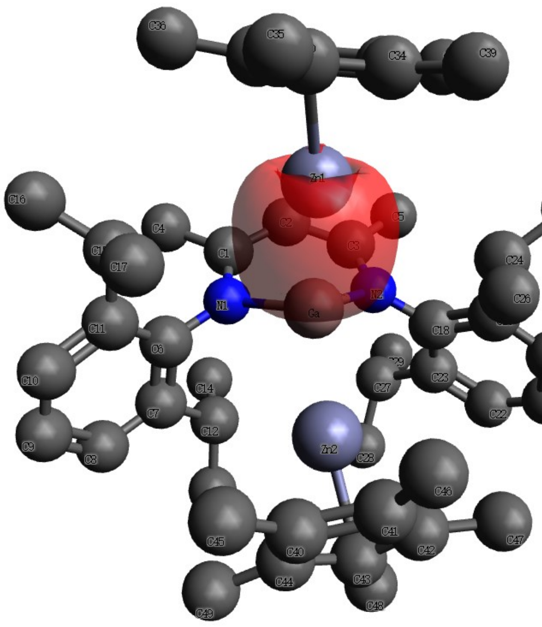
**Ga[125]–Zn[2] σ IBO**<br><br>**IBO: 49.9 % Ga[125], 51.2% Zn[2]**	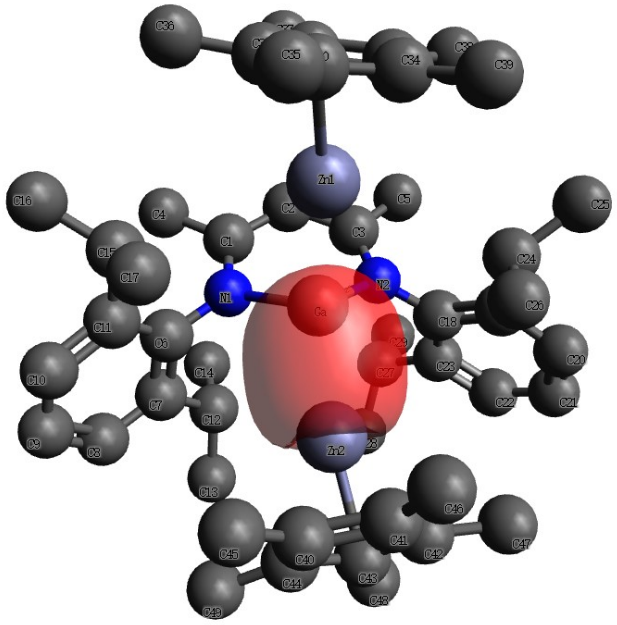

6.7.5. IBO Analysis of 2d

**In^{107}–Zn^{1} σ IBO**<br><br>**IBO: 20.1 % In^{107}, 69.1 % Zn^{1}**	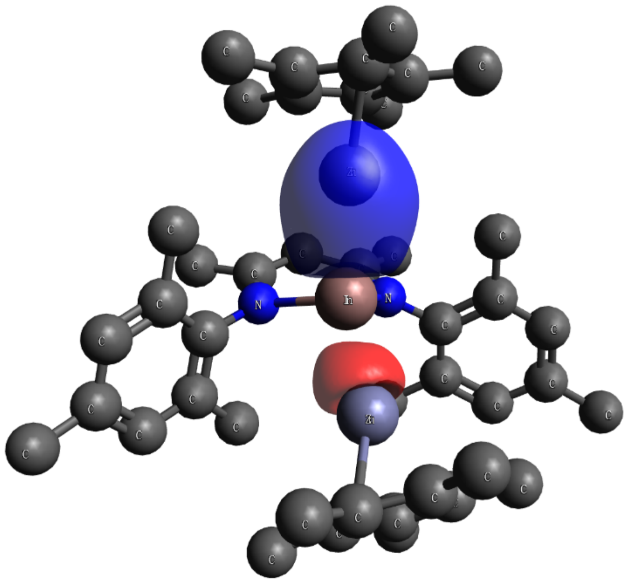
**In^{107}–Zn^{2} σ IBO**<br><br>**IBO: 51.5 % In^{107}, 45.7% Zn^{2}**	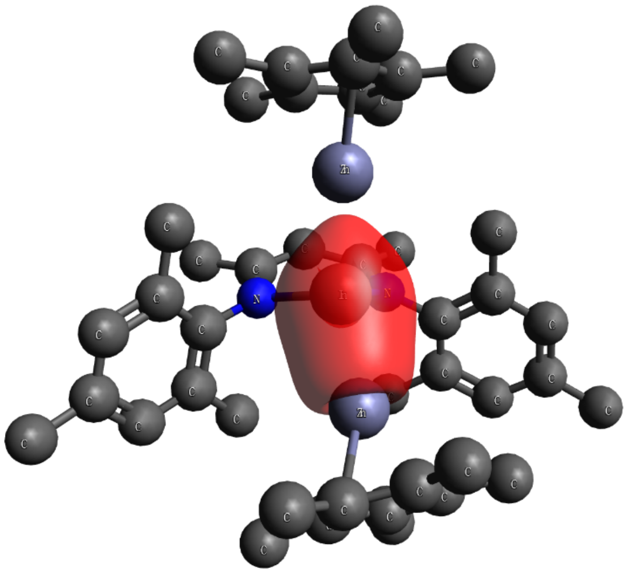

6.8. IGMH Analysis

6.8.1. IGMH Analysis for 2a

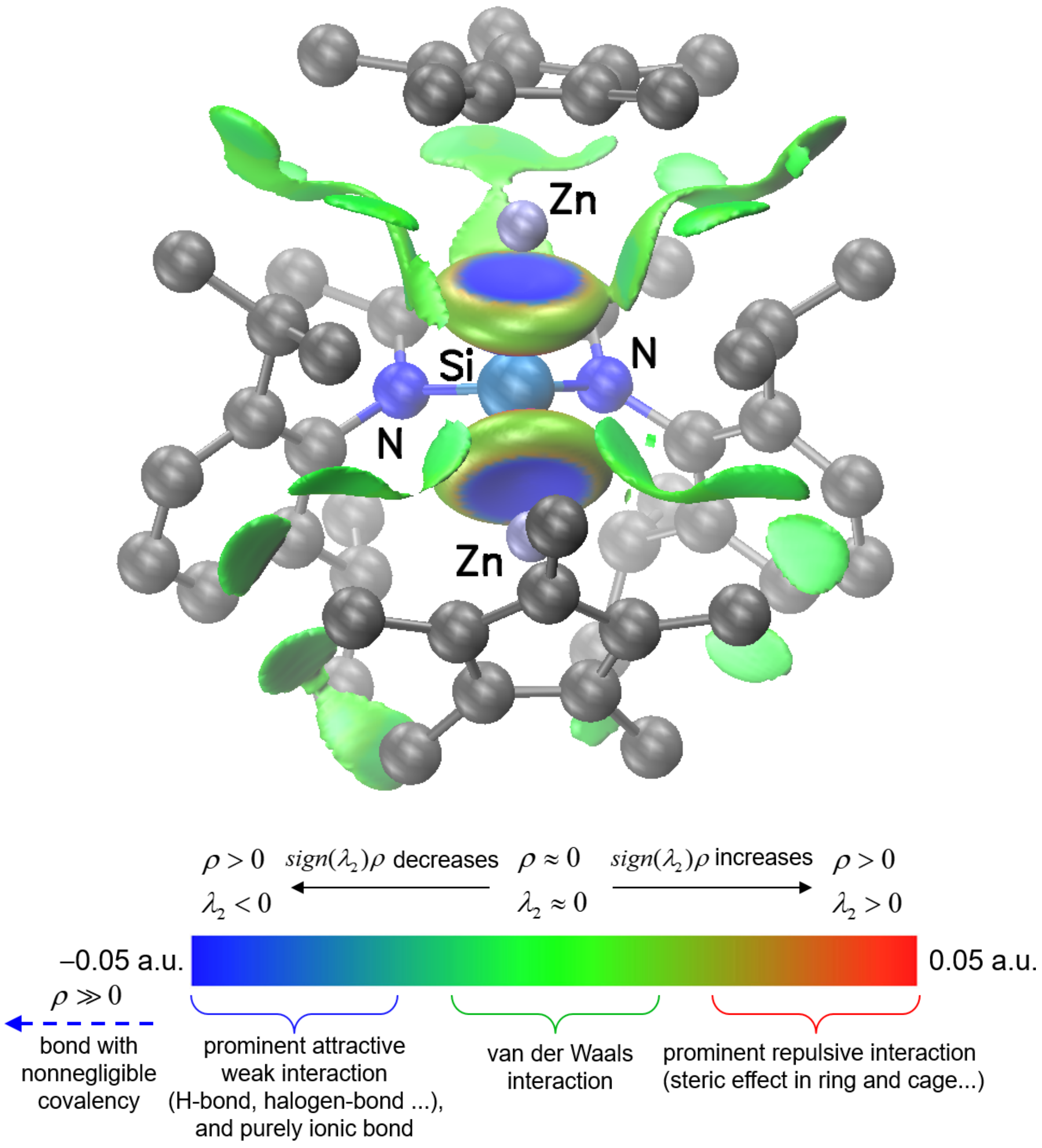

Figure S55. Plot of the interfragment interaction surface for **2a**. Fragmentation: fragment 1: [BDISi]; fragment 2: [Cp*Zn]; fragment 3: [Cp*Zn]. Isovalue = 0.005.

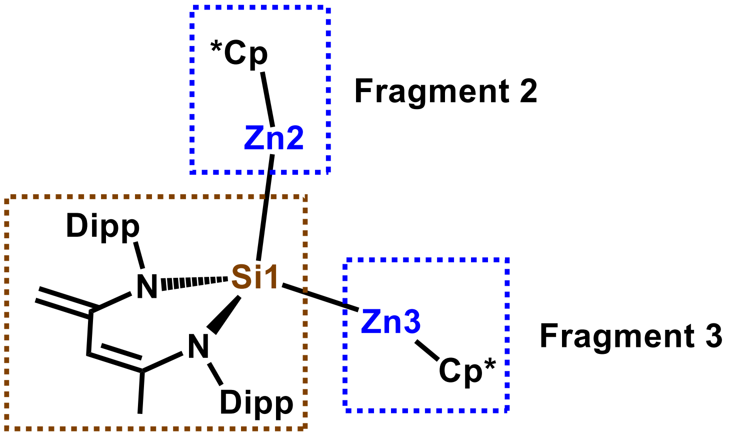

Atom	δG^{atom}	δG^{atom}%
Si1 (fragment 1)	0.628	17.37 %
Zn2 (fragment 2)	1.323	36.58 %

Table S108. Largest atomic contributions to the interfragment interaction in **2a** between fragment 1 and fragment 2

Atom	δG^{atom}	δG^{atom}%
Si1 (fragment 1)	0.663	20.24 %
Zn3 (fragment 3)	1.199	36.58 %

Table S109. Largest atomic contributions to the interfragment interaction in **2a** between fragment 1 and fragment 3

Atom	δG^{atom}	δG^{atom}%
Zn2 (fragment 2)	0.062	77.45 %
Zn3 (fragment 3)	0.052	64.51 %

Table S110. Largest atomic contributions to the interfragment interaction in **2a** between fragment 2 and fragment 3

Atom (fragment 1)	Atom (fragment 2)	δG^{atom}	δG^{atom}%
Si1	Zn2	0.485	13.41 %

Table S111. Largest pairwise contributions between fragment 1 and fragment 2 interaction in **2a**

Atom (fragment 1)	Atom (fragment 3)	δG^{atom}	$\delta G^{atom}\%$
**Si1**	**Zn3**	**0.507**	**15.46 %**

Table S112. Largest pairwise contributions between fragment 1 and fragment 3 interaction in **2a**

Atom (fragment 2)	Atom (fragment 3)	δG^{atom}	$\delta G^{atom}\%$
**Zn2**	**Zn3**	**0.043**	**53.05 %**

Table S113. largest pairwise contributions between fragment 2 and fragment 3 interaction in **2a**

Atom 1	Atom 2	Δ_g(BCP) [density, Hirshfield]	Δ_g(BCP) [promolecule]	Sign(λ_2)ρ [density]	Sign(λ_2)ρ [promolecule]
**Si1**	**Zn2**	**0.126**	**0.686**	**-0.775**	**-0.535**
**Si1**	**Zn3**	**0.138**	**0.736**	**-0.840**	**-0.587**

Table S114. IGMH data at the BCPs for **2a**

6.8.2. IGMH Analysis for 2b

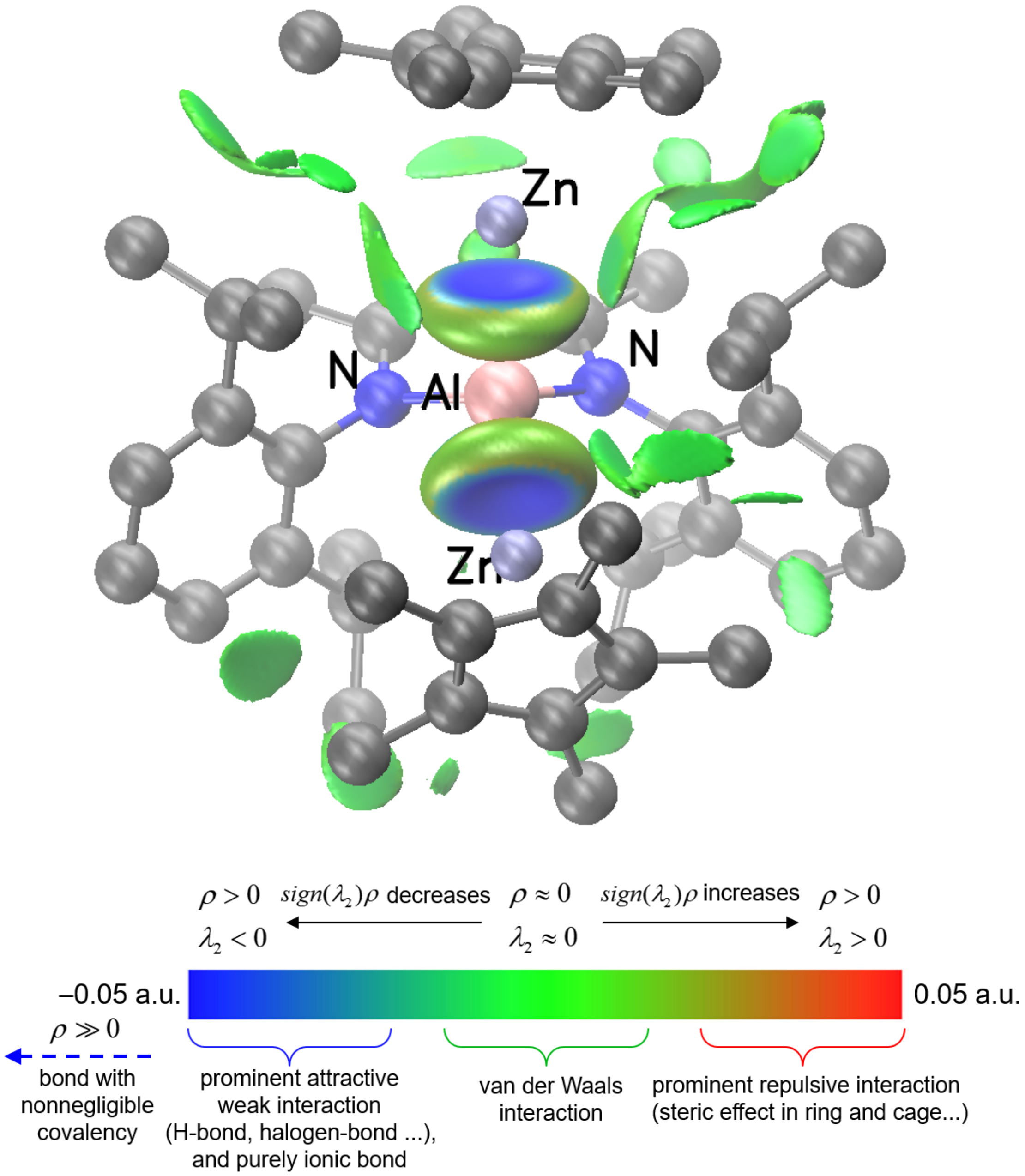

Figure S56. Plot of the interfragment interaction surface for **2b**. Fragmentation: fragment 1: [BDIAl]; fragment 2: [Cp*Zn]; fragment 3: [Cp*Zn]. Isovalue = 0.005.

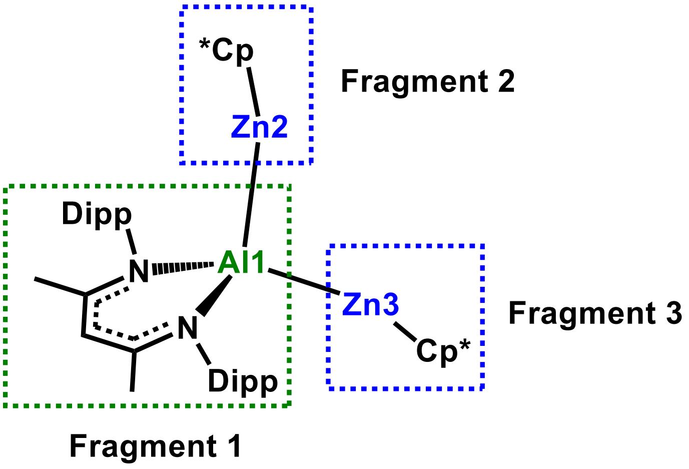

Atom	δG^{atom}	δG^{atom}%
Al1 (fragment 1)	0.563	17.48 %
Zn2 (fragment 2)	1.143	35.50 %

Table S115. Largest atomic contributions to the interfragment interaction in **2b** between fragment 1 and fragment 2

Atom	δG^{atom}	δG^{atom}%
Al1 (fragment 1)	0.602	23.97 %
Zn3 (fragment 3)	1.000	39.83 %

Table S116. Largest atomic contributions to the interfragment interaction in **2b** between fragment 1 and fragment 3

Atom	δG^{atom}	δG^{atom}%
Zn2 (fragment 2)	0.054	81.17 %
Zn3 (fragment 3)	0.043	64.49 %

Table S117. Largest atomic contributions to the interfragment interaction in **2b** between fragment 2 and fragment 3

Atom (fragment 1)	Atom (fragment 2)	δG^{atom}	δG^{atom}%
Al1	Zn2	0.420	13.04 %

Table S118. Largest pairwise contributions between fragment 1 and fragment 2 interaction in **2b**

Atom (fragment 1)	Atom (fragment 3)	δG^{atom}	δG^{atom}%
Al1	Zn3	0.436	17.35 %

Table S119. Largest pairwise contributions between fragment 1 and fragment 3 interaction in **2b**

Atom (fragment 2)	Atom (fragment 3)	δG^{atom}	δG^{atom}%
Zn2	Zn3	0.037	55.20 %

Table S120. Largest pairwise contributions between fragment 2 and fragment 3 interaction in **2b**

Atom 1	Atom 2	Δ_g(BCP) [density, Hirshfield]	Δ_g(BCP) [promolecule]	Sign(λ_2)ρ [density]	Sign(λ_2)ρ [promolecule]
Al1	Zn2	0.411	0.212	-0.534	-0.588
Al1	Zn3	0.502	0.253	-0.590	-0.637

Table S121. IGMH data at the BCPs for **2b**

6.8.2. IGMH Analysis for 2c

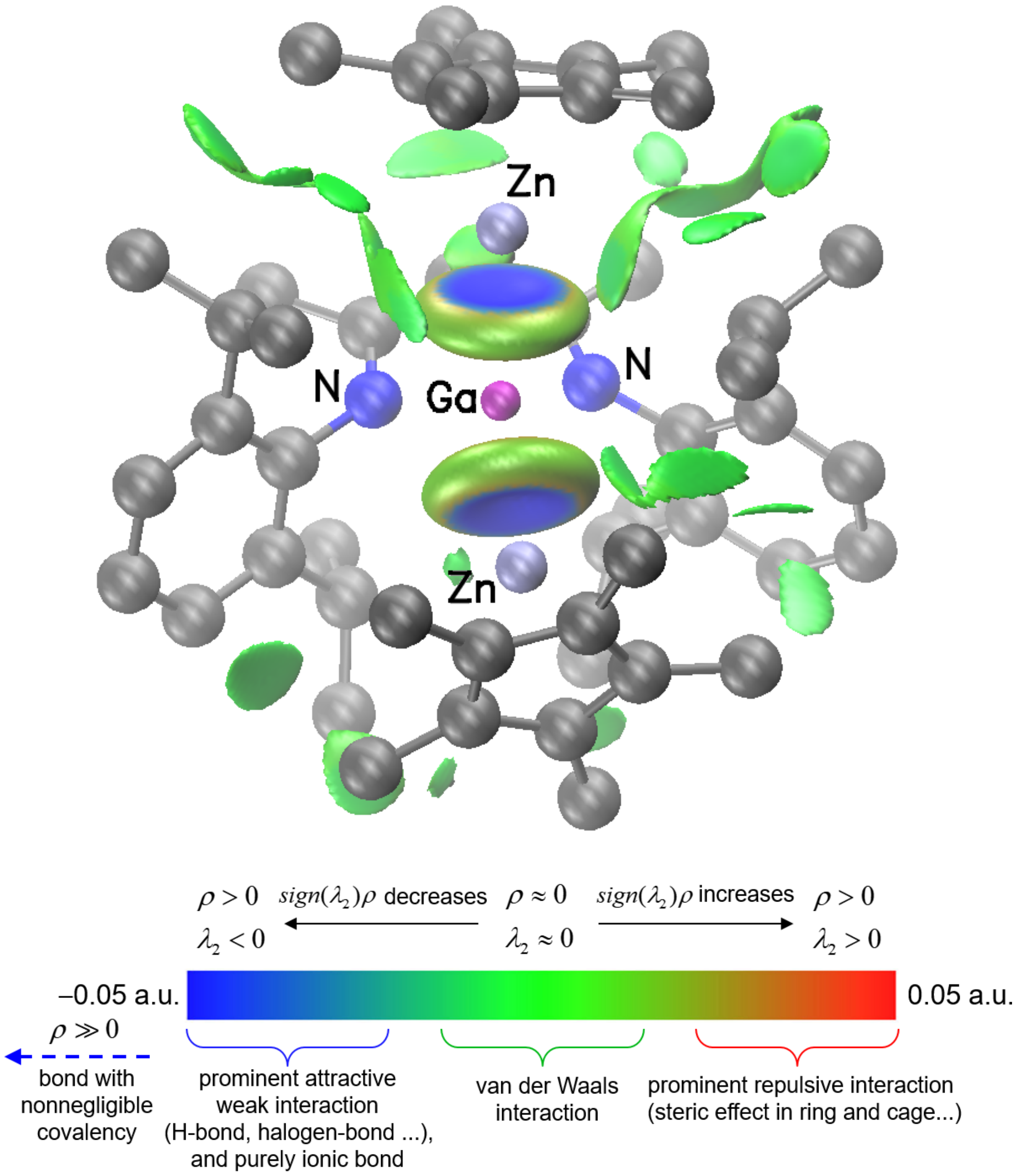

Figure S57. Plot of the interfragment interaction surface for **2c**. Fragmentation: fragment 1: [BDIGa]; fragment 2: [Cp*Zn]; fragment 3: [Cp*Zn]. Isovalue = 0.005.

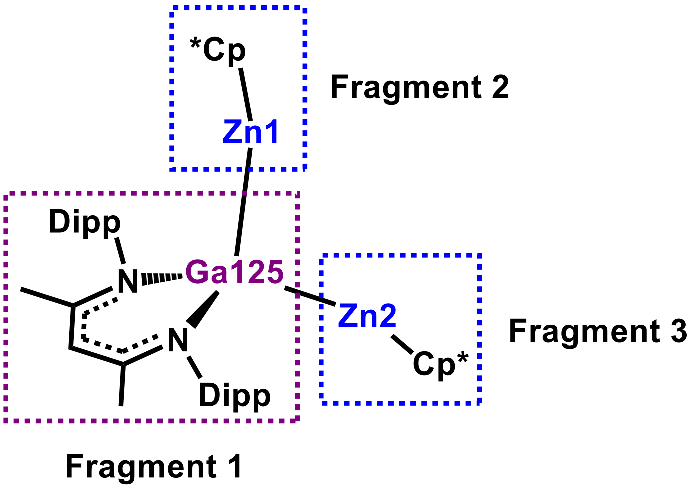

Atom	δG^{atom}	δG^{atom}%
Ga125 (fragment 1)	0.559	17.61 %
Zn1 (fragment 2)	1.104	34.80 %

Table S122. Largest atomic contributions to the interfragment interaction in **2c** between fragment 1 and fragment 2

Atom	δG^{atom}	δG^{atom}%
Ga125 (fragment 1)	0.607	24.55 %
Zn2 (fragment 3)	0.976	39.43 %

Table S123. Largest atomic contributions to the interfragment interaction in **2c** between fragment 1 and fragment 3

Atom	δG^{atom}	δG^{atom}%
Zn1 (fragment 2)	0.044	78.72 %
Zn2 (fragment 3)	0.033	59.10 %

Table S124. Largest atomic contributions to the interfragment interaction in **2c** between fragment 2 and fragment 3

Atom (fragment 1)	Atom (fragment 2)	δG^{atom}	δG^{atom}%
Ga125	Zn1	0.424	13.37 %

Table S125. Largest pairwise contributions between fragment 1 and fragment 2 interaction in **2c**

Atom (fragment 1)	Atom (fragment 3)	δG^{atom}	δG^{atom}%
**Ga125**	**Zn2**	**0.445**	**17.99 %**

Table S126. Largest pairwise contributions between fragment 1 and fragment 3 interaction in **2c**

Atom (fragment 2)	Atom (fragment 3)	δG^{atom}	δG^{atom}%
**Zn1**	**Zn2**	**0.028**	**49.13 %**

Table S127. Largest pairwise contributions between fragment 2 and fragment 3 interaction in **2c**

Atom 1	Atom 2	Δ_g(BCP) [density, Hirshfield]	Δ_g(BCP) [promolecule]	Sign(λ_2)ρ [density]	Sign(λ_2)ρ [promolecule]
**Ga125**	**Zn1**	**0.104**	**0.608**	**-0.632**	**-0.402**
**Ga125**	**Zn2**	**0.118**	**0.720**	**-0.694**	**-0.451**

Table S128. IGMH data at the BCPs for **2c**

6.8.3. IGMH Analysis for 2d

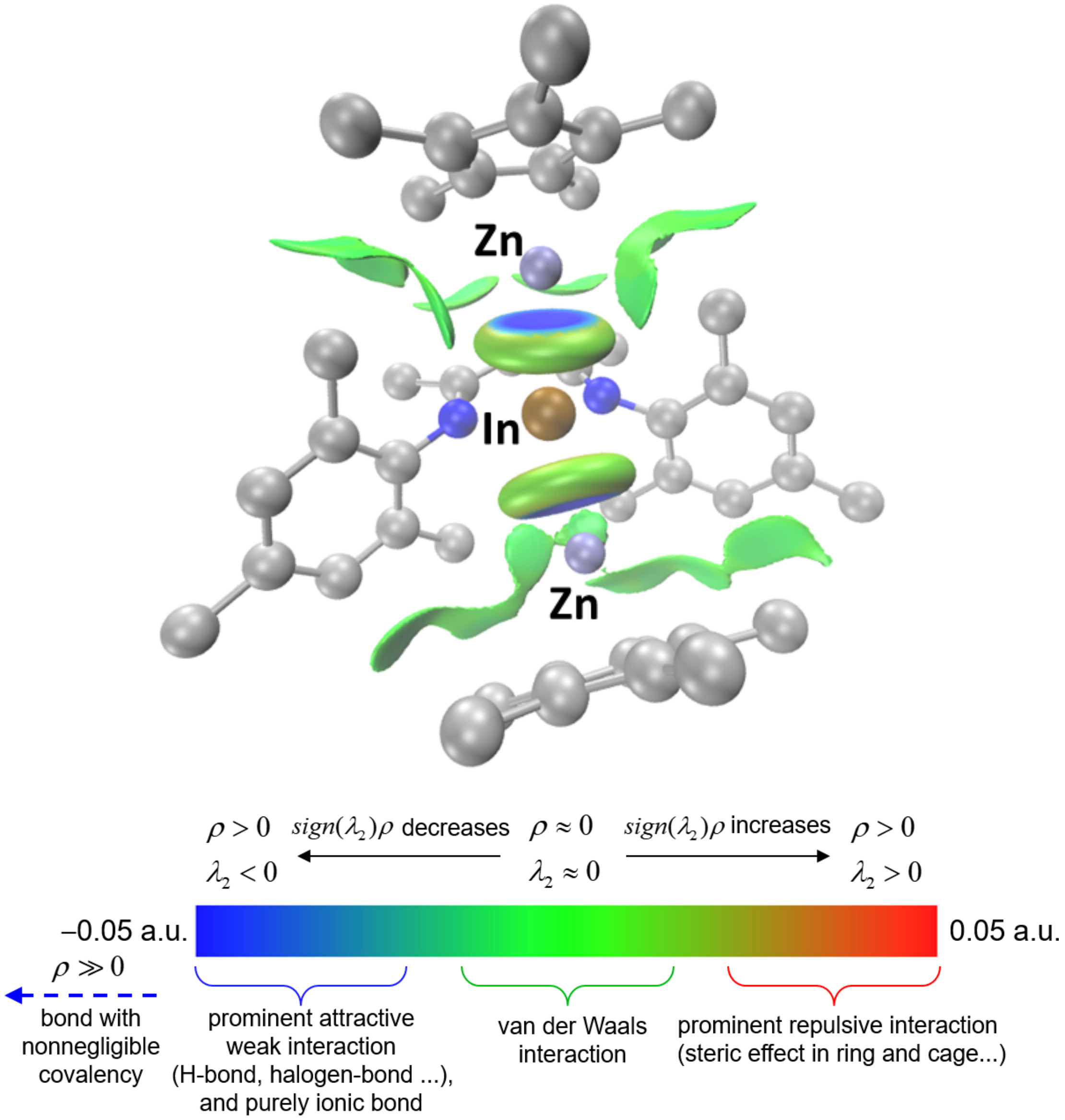

Figure S58. Plot of the interfragment interaction surface for **3**. Fragmentation: fragment 1: [BDIIn]; fragment 2: [Cp*Zn]; fragment 3: [Cp*Zn]. Isovalue = 0.005.

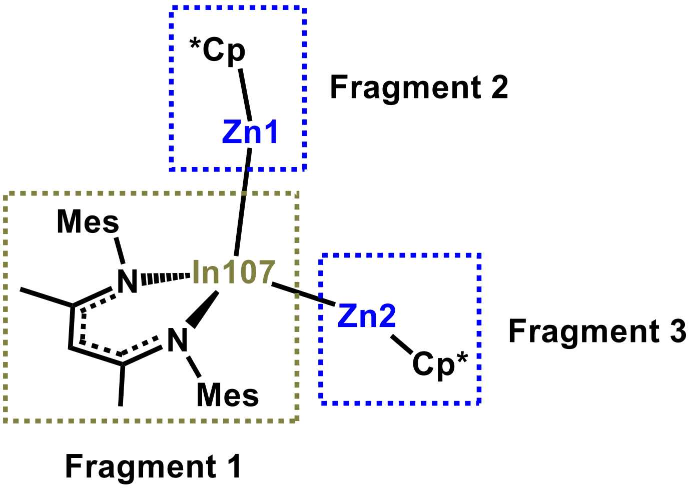

Atom	δG^{atom}	δG^{atom}%
In107 (fragment 1)	0.554	22.28 %
Zn1 (fragment 2)	0.820	36.58 %

Table S129. Largest atomic contributions to the interfragment interaction in **2d** between fragment 1 and fragment 2

Atom	δG^{atom}	δG^{atom}%
In107 (fragment 1)	0.549	24.49 %
Zn2 (fragment 3)	0.802	35.77 %

Table S130. Largest atomic contributions to the interfragment interaction in **2d** between fragment 1 and fragment 3

Atom (fragment 1)	Atom (fragment 2)	δG^{atom}	δG^{atom}%
In107	Zn1	0.390	15.70 %

Table S131. Largest pairwise contributions between fragment 1 and fragment 2 interaction in **2d**

Atom (fragment 1)	Atom (fragment 3)	δG^{atom}	δG^{atom}%
In107	Zn2	0.401	17.90 %

Table S132. Largest pairwise contributions between fragment 1 and fragment 3 interaction in **2d**

Atom 1	Atom 2	Δ_g(BCP) [density, Hirshfield]	Δ_g(BCP) [promolecule]	Sign(λ_2)ρ [density]	Sign(λ_2)ρ [promolecule]
In107	Zn1	0.102	0.643	-0.590	-0.399
In107	Zn2	0.109	0.701	-0.616	-0.422

Table S133. IGMH data at the BCPs for **2d**

6.8.3. IGMH Analysis for 3

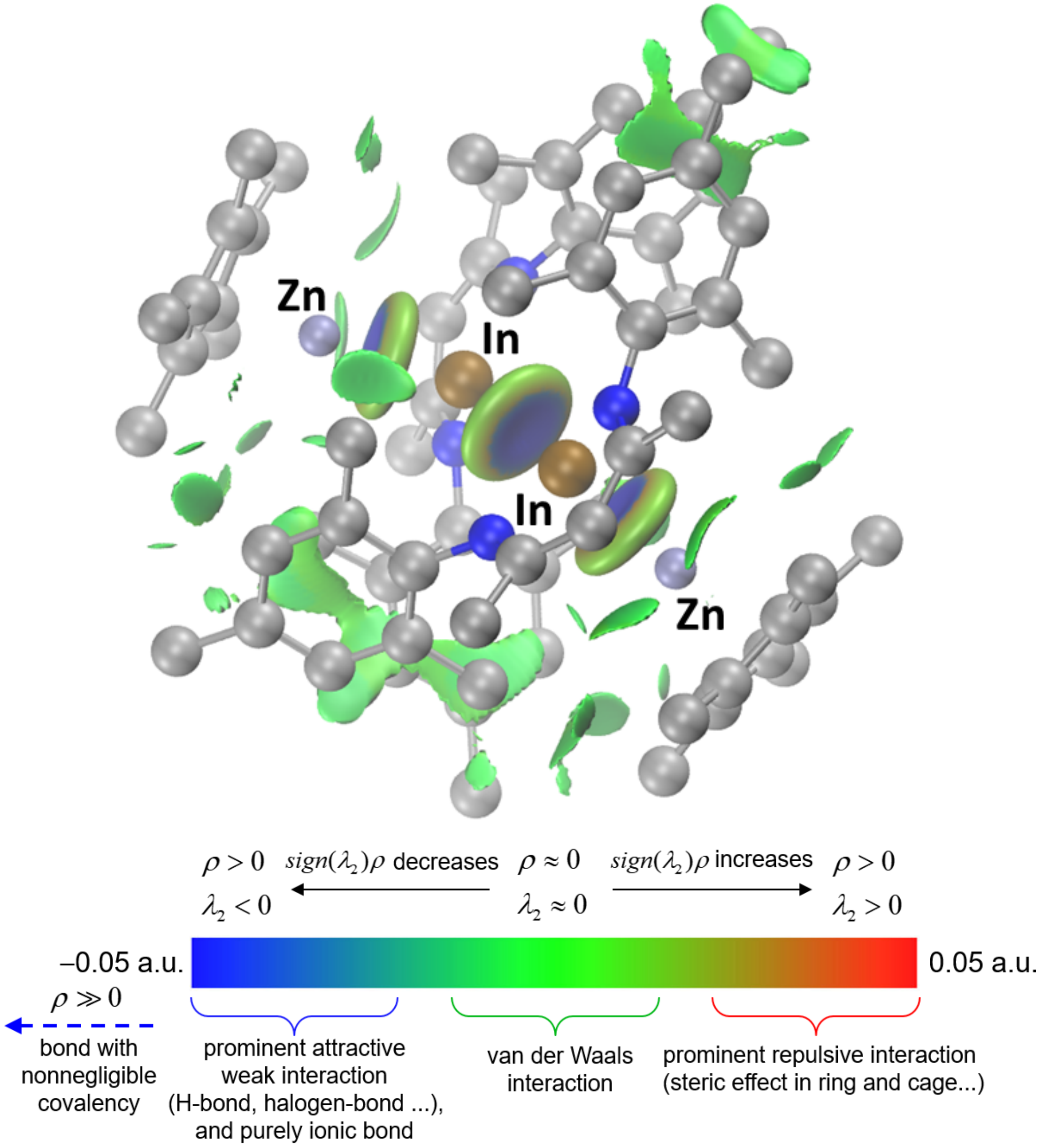

Figure S59. Plot of the interfragment interaction surface for **3**. Fragmentation: fragment 1: [BDIIn]; fragment 2: [Cp*Zn]; fragment 3: [Cp*Zn]; fragment 4: [BDIIn]. Isovalue = 0.005.

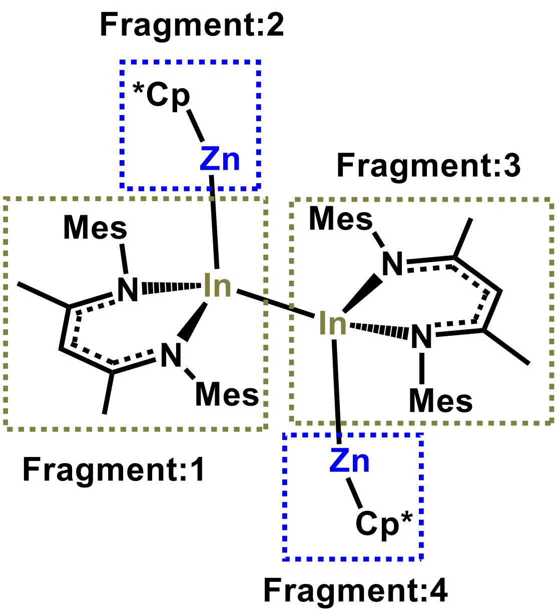

Atom	δG^atom	δG^atom%
**In1 (fragment 1)**	**0.570**	**24.66 %**
**Zn2 (fragment 2)**	**0.777**	**33.65%**

Table S134. Largest atomic contributions to the interfragment interaction in **3** between fragment 1 and fragment 2

Atom	δG^atom	δG^atom%
**In1 (fragment 1)**	**0.734**	**19.85 %**
**In82 (fragment 3)**	**0.739**	**19.98 %**

Table S135. Largest atomic contributions to the interfragment interaction in **3** between fragment 1 and fragment 3

Atom	δG^atom	δG^atom%
**In1 (fragment 1)**	**0.026**	**3.45 %**
**Zn83 (fragment 4)**	**0.213**	**27.97 %**

Table S136. Largest atomic contributions to the interfragment interaction in **3** between fragment 1 and fragment 4

Atom	δG^atom	δG^atom%
**Zn2 (fragment 2)**	**0.218**	**28.51 %**
**In82 (fragment 3)**	**0.026**	**3.47 %**

Table S137. Largest atomic contributions to the interfragment interaction in **3** between fragment 2 and fragment 3

Atom	δG^{atom}	δG^{atom}%
**In82 (fragment 3)**	**0.568**	**24.74 %**
**Zn83 (fragment 4)**	**0.789**	**34.36 %**

Table S138. Largest atomic contributions to the interfragment interaction in **3** between fragment 3 and fragment 4

Atom (fragment 1)	Atom (fragment 2)	δG^{atom}	δG^{atom}%
**In1**	**Zn2**	**0.399**	**17.28 %**

Table S139. Largest pairwise contributions between fragment 1 and fragment 2 interaction in **3**

Atom (fragment 1)	Atom (fragment 3)	δG^{atom}	δG^{atom}%
**In1**	**In82**	**0.393**	**10.63 %**

Table S140. Largest pairwise contributions between fragment 1 and fragment 3 interaction in **3**

Atom (fragment 2)	Atom (fragment 3)	δG^{atom}	δG^{atom}%
**In1**	**In83**	**0.023**	**3.04 %**

Table S141. Largest pairwise contributions between fragment 1 and fragment 4 interaction in **3**

Atom (fragment 2)	Atom (fragment 3)	δG^{atom}	δG^{atom}%
**Zn2**	**In82**	**0.023**	**3.08 %**

Table S142. Largest pairwise contributions between fragment 2 and fragment 3 interaction in **3**

Atom (fragment 3)	Atom (fragment 4)	δG^{atom}	δG^{atom}%
**In82**	**Zn83**	**0.399**	**17.41 %**

Table S143. Largest pairwise contributions between fragment 3 and fragment 4 interaction in **3**

Atom 1	Atom 2	Δ_g(BCP) [density, Hirshfield]	Δ_g(BCP) [promolecule]	Sign(λ_2)ρ [density]	Sign(λ_2)ρ [promolecule]
**In1**	**Zn2**	**0.105**	**0.656**	**-0.601**	**-0.410**
**In1**	**In82**	**0.955**	**0.637**	**-0.560**	**-0.380**
**In82**	**Zn83**	**0.105**	**0.654**	**-0.600**	**-0.410**

Table S144. IGMH data at the BCPs for **3**

6.9. NCIPLOT Analysis

6.9.1. NCIPLOT Analysis for 2a

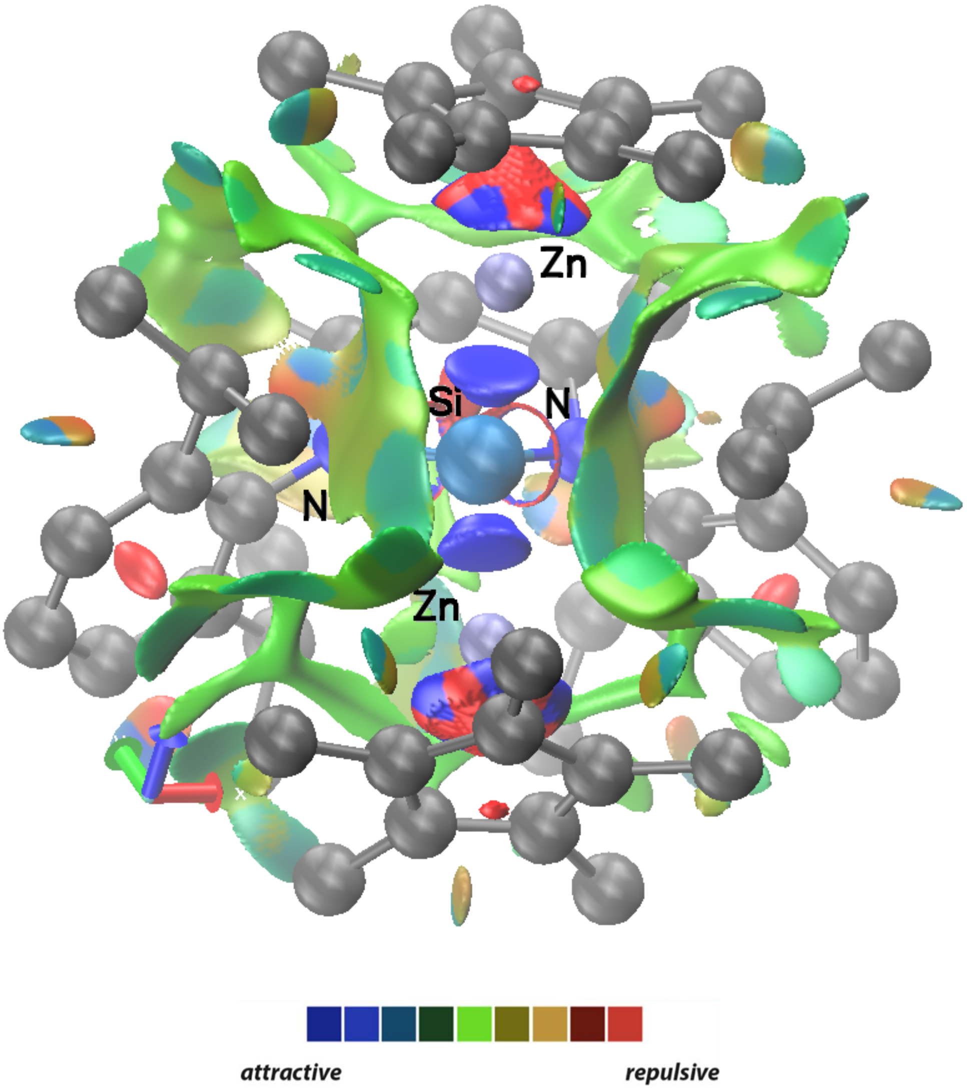

Figure S60. NCIPLOT for **2a**.

6.9.2. NCIPLOT Analysis for 2b

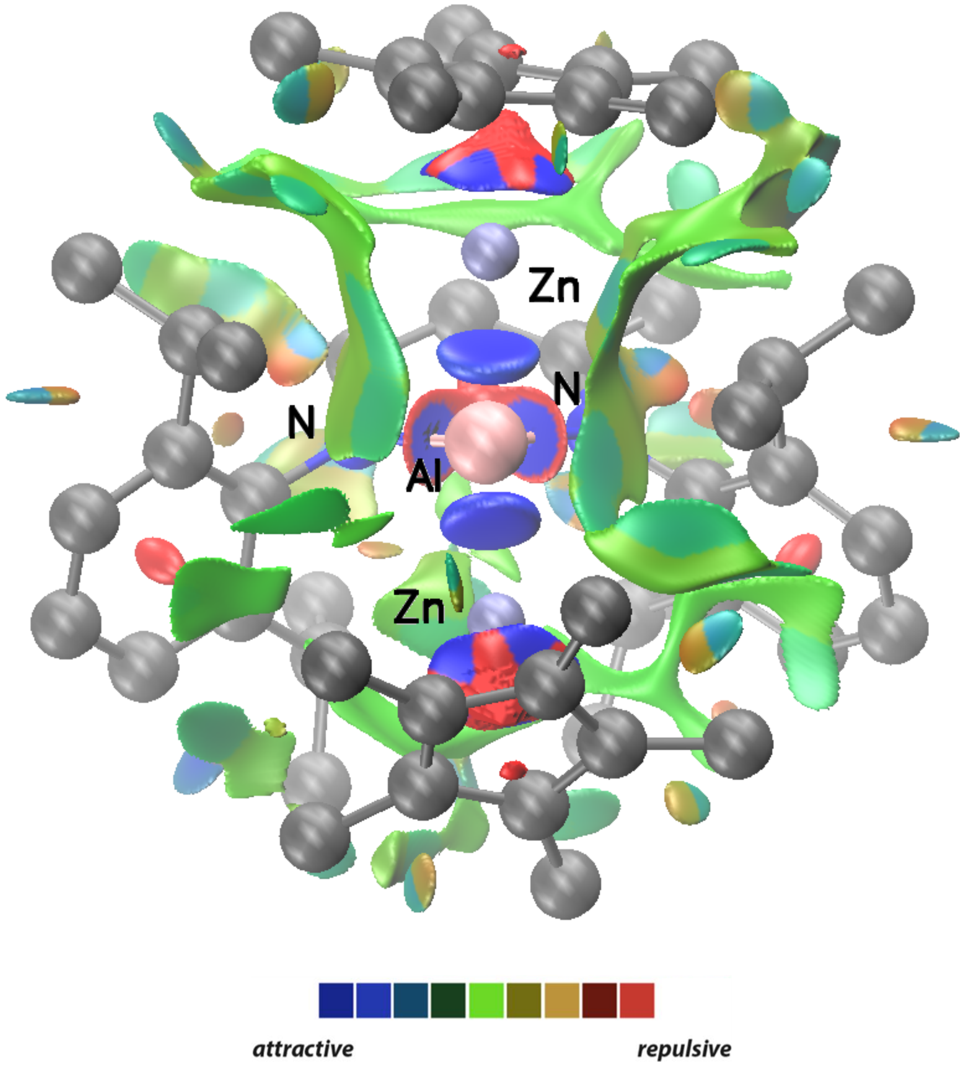

Figure S61. NCIPLOT for **2b**.

6.9.3. NCIPLOT Analysis for 2c

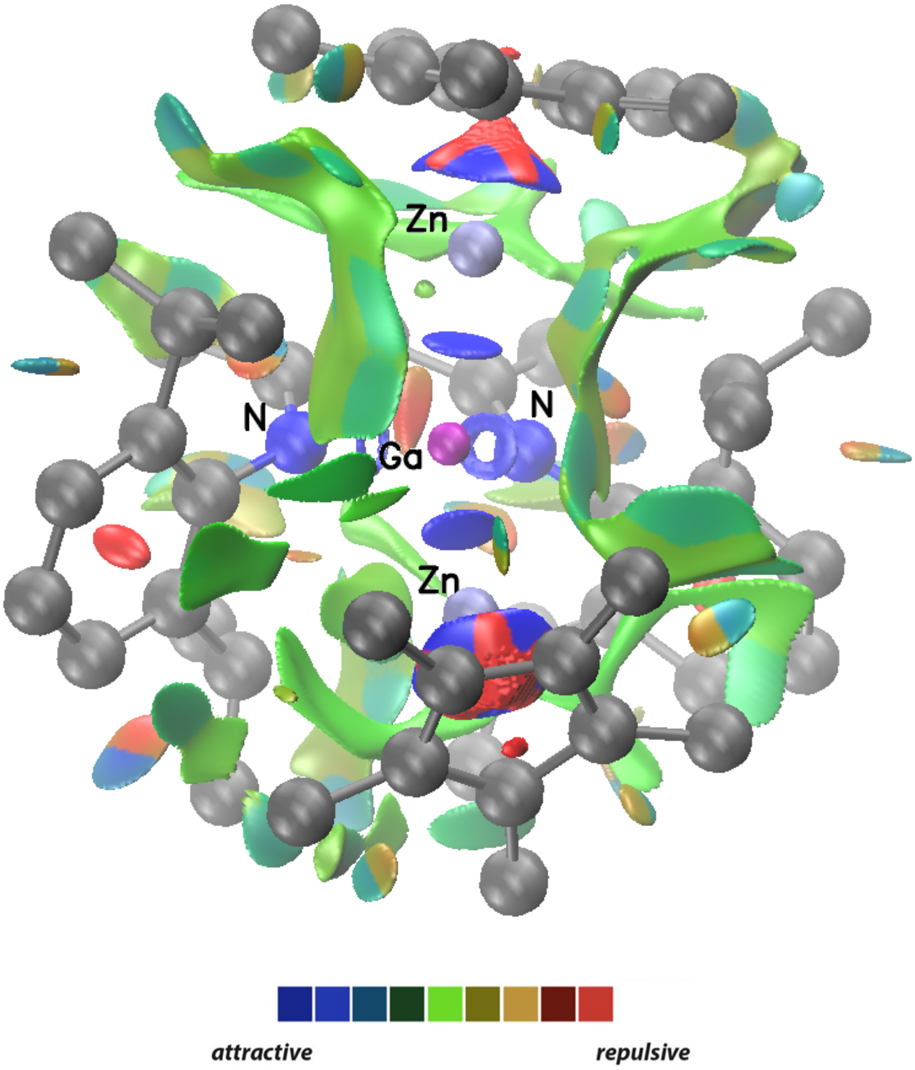

Figure S62. NCIPLOT for **2c**.

6.9.4. NCIPLOT Analysis for 2d

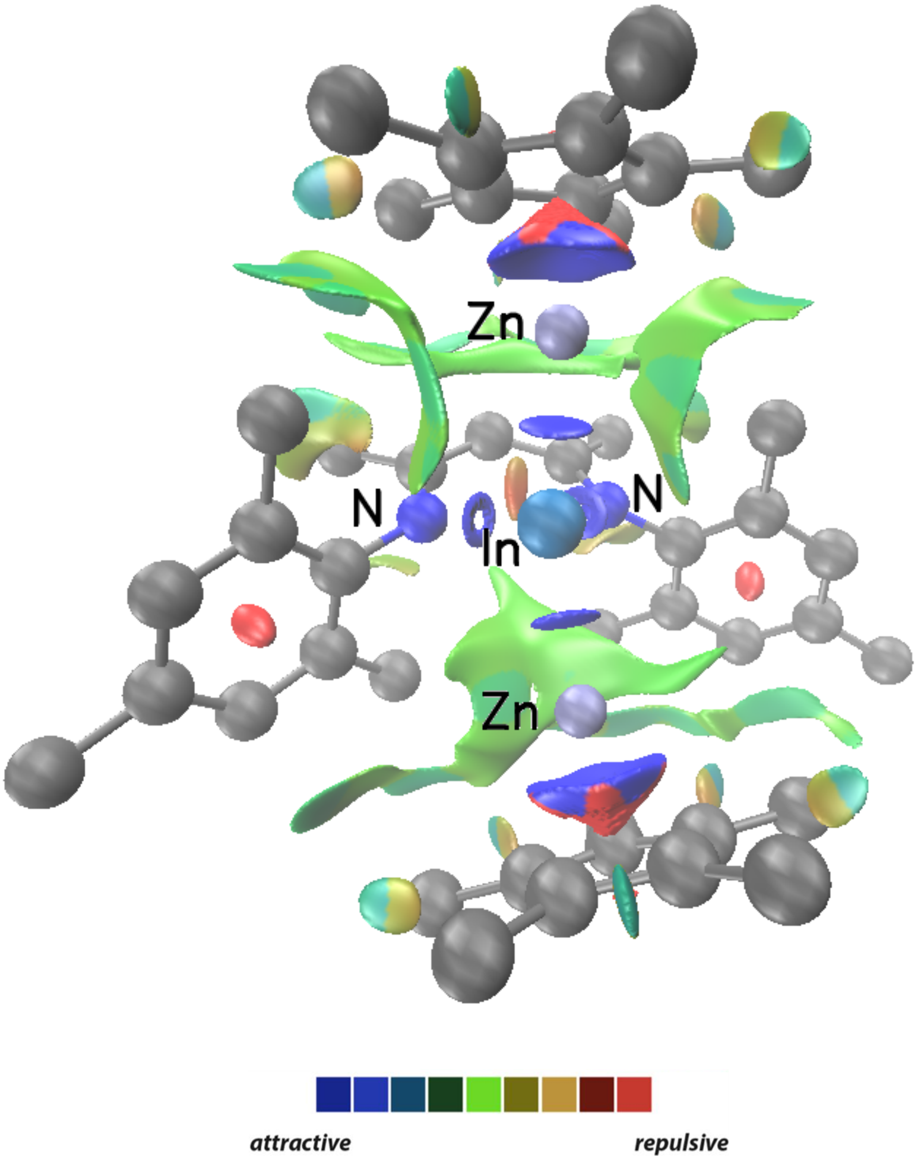

Figure S63. NCIPLOT for **2d**.

6.10 Homolytic Cleavage of E–Zn Bonds

Further calculations were undertaken to understand the energetic cost of homolytic cleavage of the E–Zn bonds in **2a-c** and **3**. These calculations suggest that barriers to dissociation are beyond room temperature process.

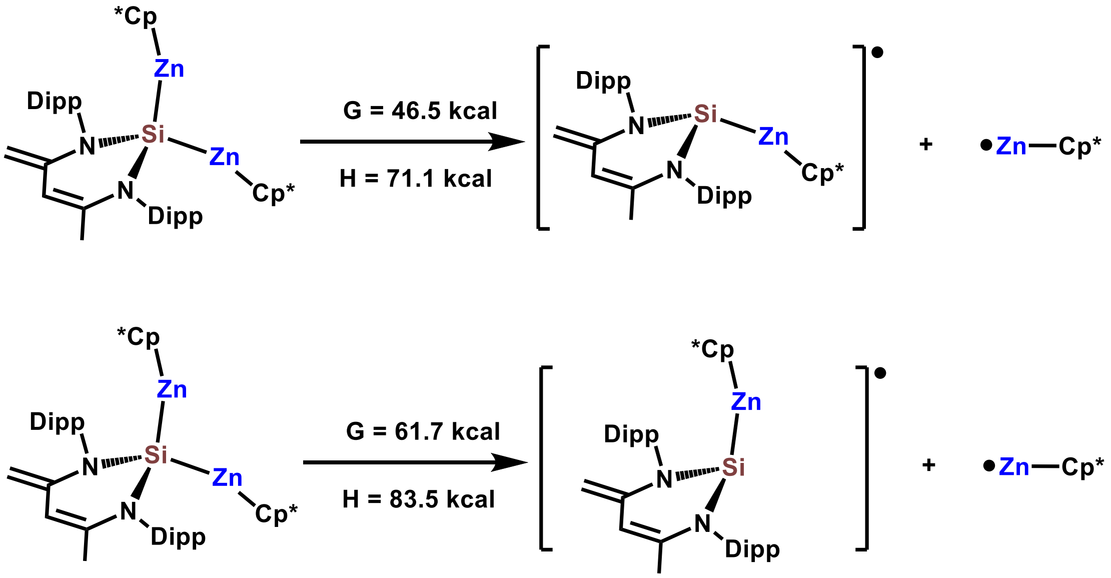

Figure S64. Proposed pathway for the formation of **2a** based on DFT calculations G09: M06-L /def2TZVPP/ PCM (benzene) // M06-L / 6-31G** / 6-311+G* / SDDAll (Zn).

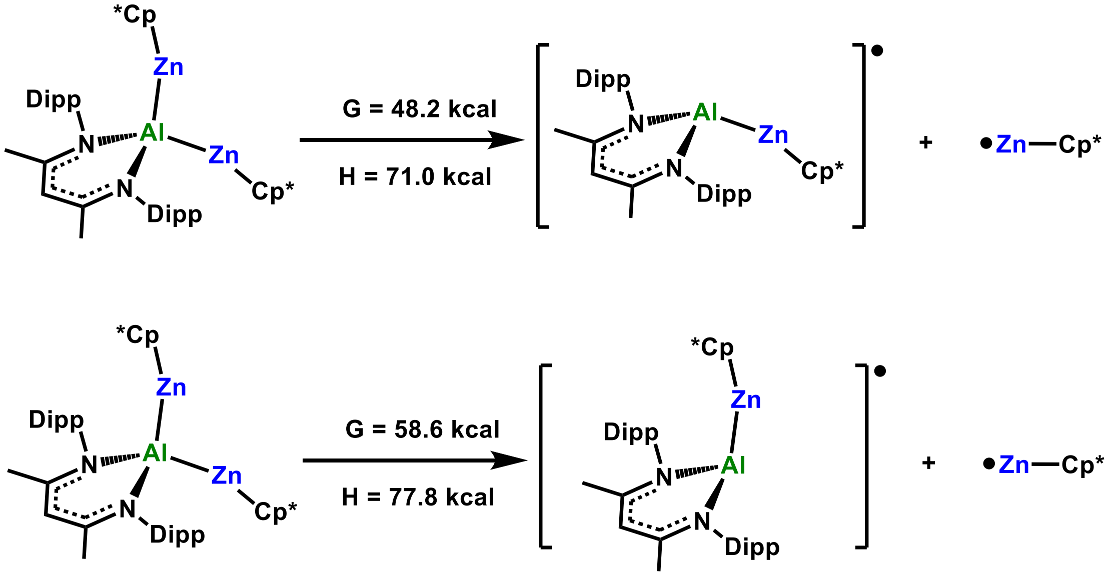

Figure S65. Proposed pathway for the formation of **2b** based on DFT calculations G09: M06-L /def2TZVPP/ PCM (benzene) // M06-L / 6-31G** / 6-311+G* / SDDAll (Al, Zn).

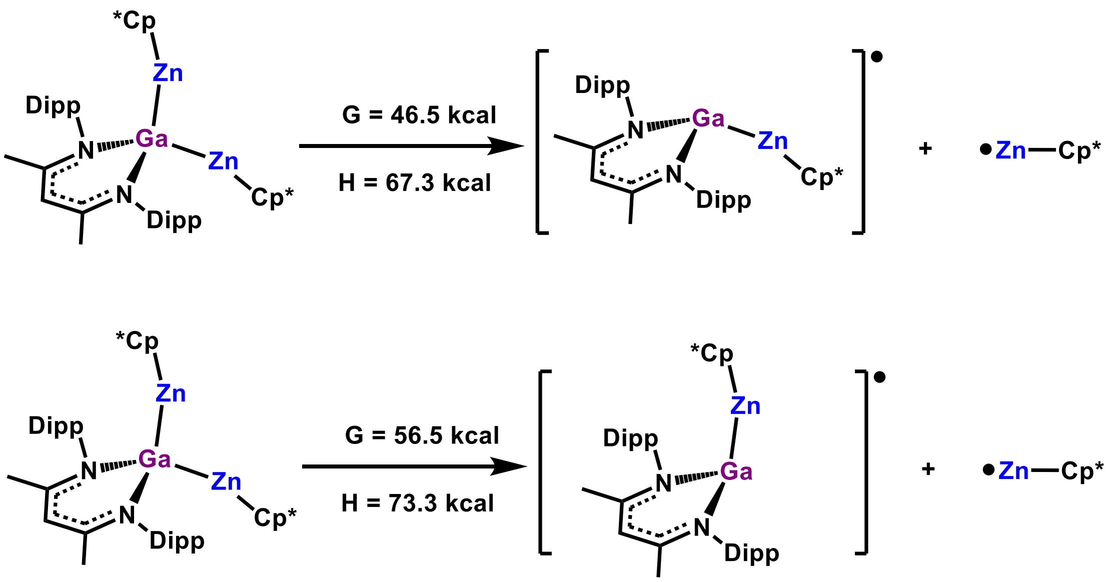

Figure S66. Proposed pathway for the formation of **2c** based on DFT calculations G09: M06-L /def2TZVPP/ PCM (benzene) // M06-L / 6-31G** / 6-311+G* / SDDAll (Ga, Zn).

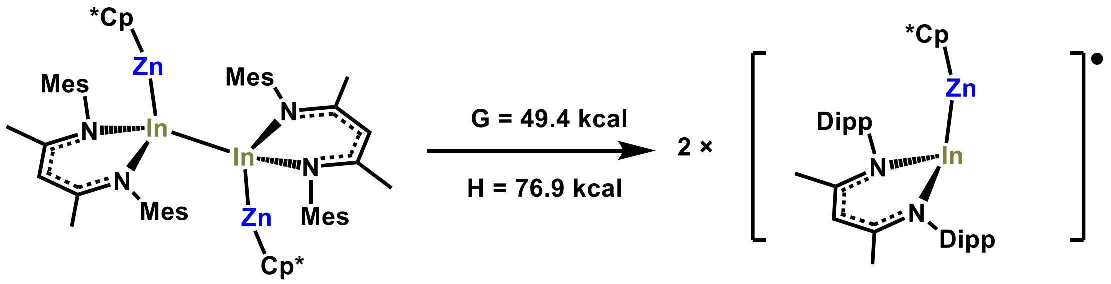

Figure S67. Proposed pathway for the formation of **3** based on DFT calculations G16: BP86-D3/def2TZVPP/ PCM (Tetrahydrofuran) // BP86-D3 / 6-31G** / 6-311+G* / SDDAll (Zn, In).

6.11 Calculated NPA and AIM Charges for Addition of Zn–Zn and H–H bonds to Ni and Ir centres

Additional calculations were undertaken to compare charge distribution in a related transition metal complex to those of **2a-d**. **[Ni(PMe$_3$)(ZnCp*)(ZnMe)]** was originally reported by Fischer[S17] while the hypothetical molecule **[Ni(PMe$_3$)$_3$(H)$_2$]** has been modelled by Parkin and coworkers.[S38] NPA charges from NBO calculations show the same trend observed for main group analogues (e.g. Si, Al-In), in which there is increasing charge localisation of the central element with the decreasing electronegativity of the ligands. (χ_{Ni} = 1.88, χ_{Zn} = 1.59, χ_{H} = 2.30).

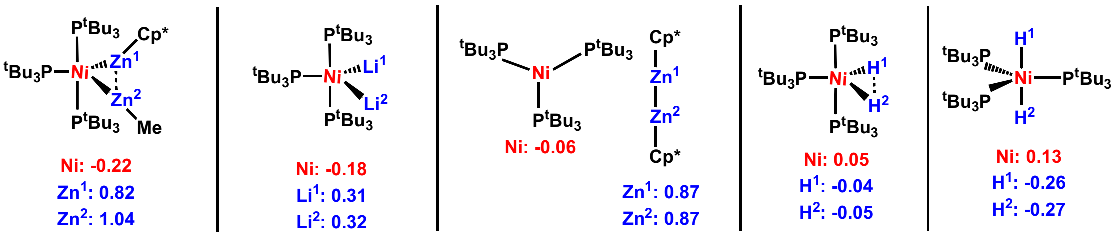

Figure S68. Analysis of NPA charges at Ni and Zn centres across a series of compounds along with dihydride and dilithium analogues. NBO calculations conducted in G09 using NBO 6.0. M06-L /def2TZVPP/ PCM (benzene).

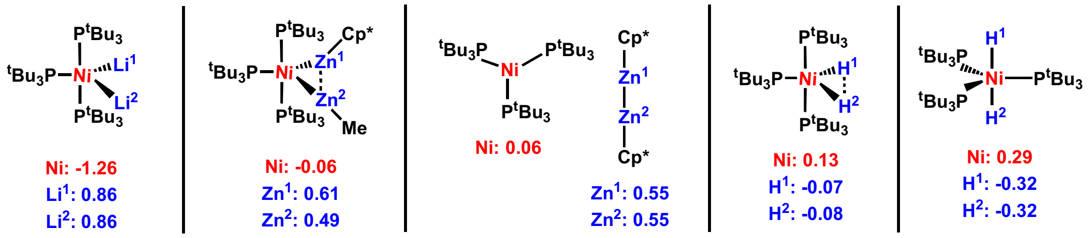

Figure S69. Analysis of AIM charges at Ni and Zn centres across a series of compounds along with dihydride and dilithium analogues.

Similarly, calculations were undertaken to investigate the theoretical addition of Cp*ZnZnCp* and H_2 to Vaska's complex [Ir(PPh_3)$_2$(CO)Cl], an archetypal fragment in oxidative addition chemistry. This system is complicated by formation of several stereoisomers for the addition products.[S38] Nevertheless, the same trends based on expected impact of electronegativity of the elements involved are observed. . (χ_{Ir} = 2.20, χ_{Zn} = 1.59, χ_{H} = 2.30). The reaction between [Ir(PPh_3)$_2$(CO)Cl] and Cp*ZnZnCp* has not been investigated experimentally and it is expected that the presence of the chloride ligand will complicate the outcome of this reaction.

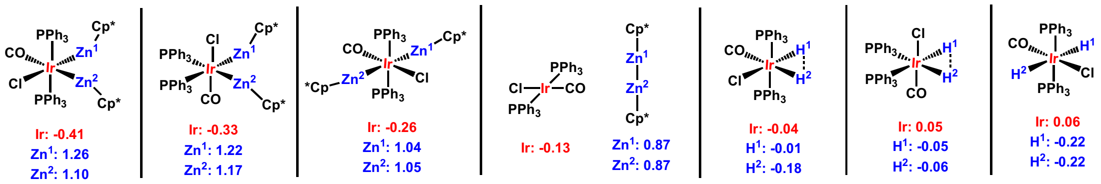

Figure S70. Analysis of NPA charges at Ir and Zn centres across a series of compounds from reaction of Cp*ZnZnCp* (or H_2) with [Ir(PPh3)2(CO)Cl]. NBO calculations conducted in G09 using NBO 6.0. Method: M06-L /def2TZVPP/ PCM

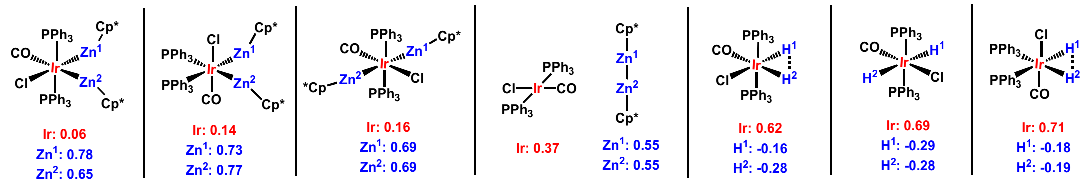

Figure S71. Analysis of AIM charges at Ir and Zn centres across a series of compounds from reaction of Cp*ZnZnCp* (or H_2) with [Ir(PPh_3)$_2$(CO)Cl]. NBO calculations conducted in G09 using NBO 6.0. Method: M06-L /def2TZVPP/ PCM

7) NMR Spectra

7.1. NMR Spectra of Isolated complexes

^{1}H NMR (C_6D_6, 298 K, 400 MHz) of **2a**:

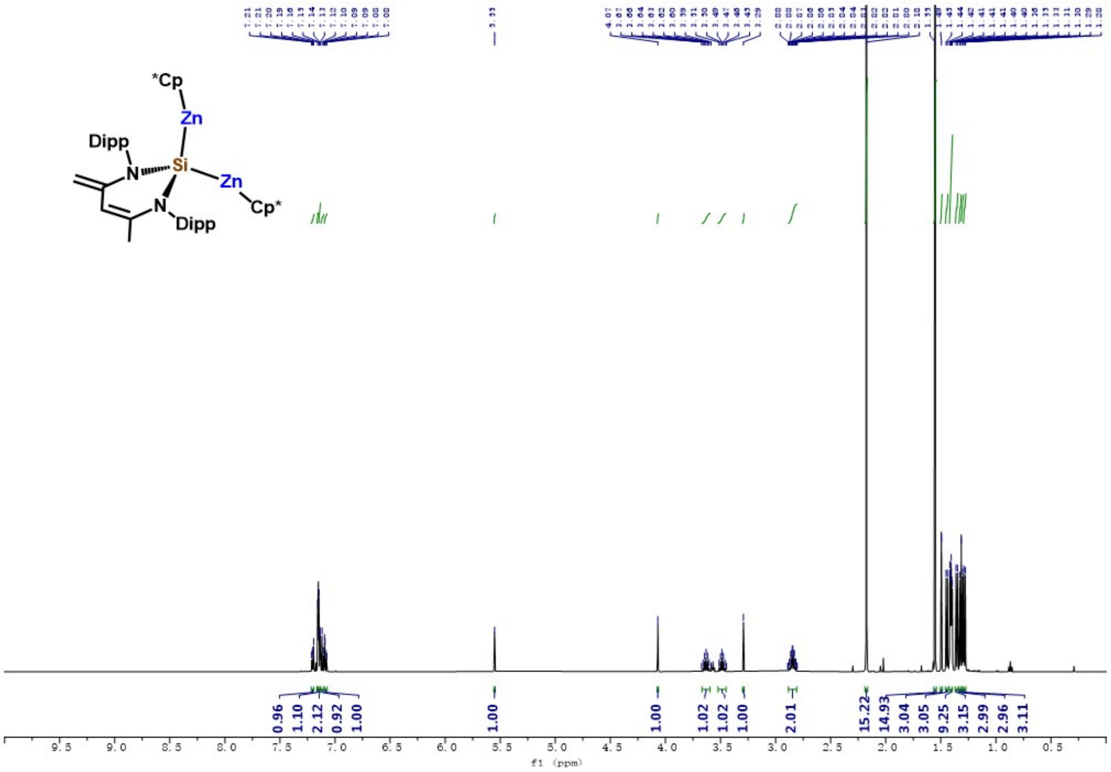

^{13}C NMR (C_6D_6, 298 K, 101 MHz) of **2a**:

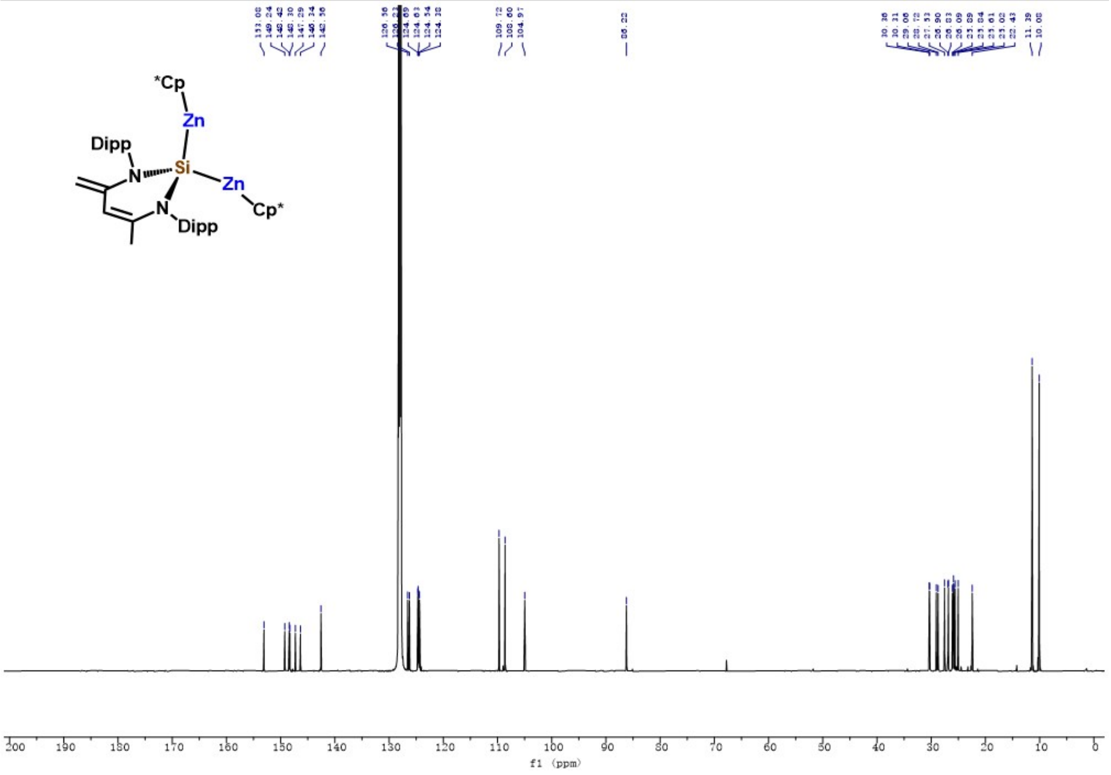

^{29}Si NMR (C_6D_6, 298 K, 99.4 MHz) of **2c**:

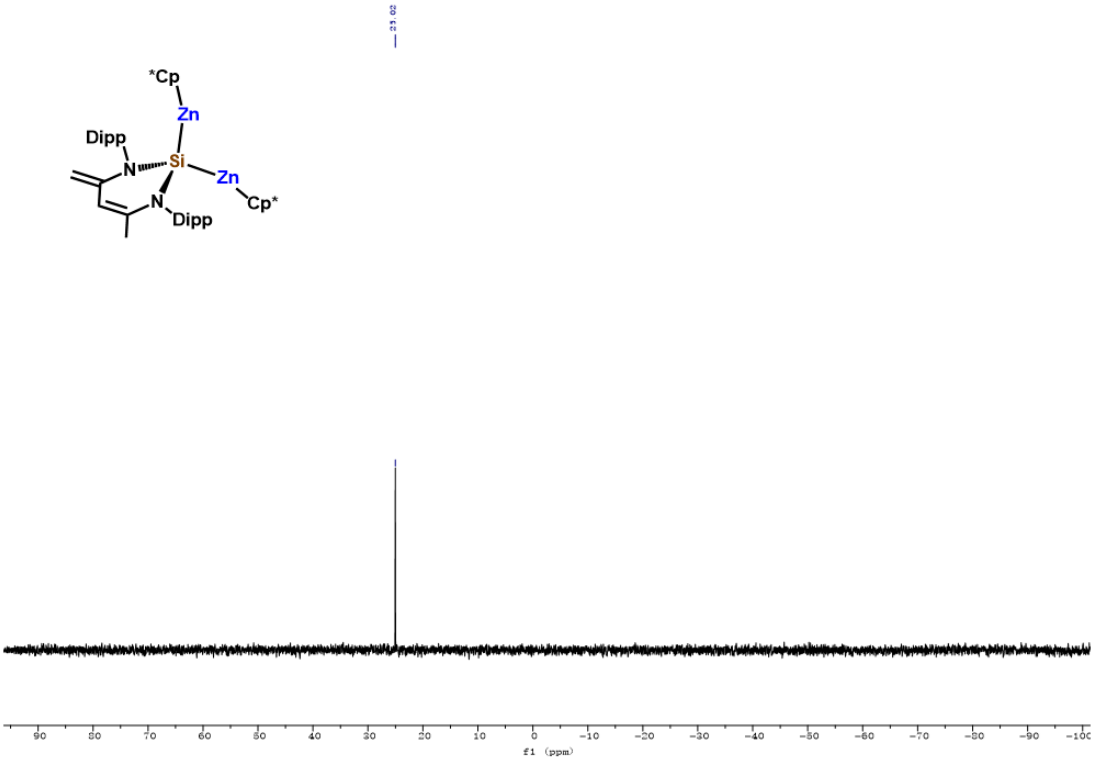

^{1}H NMR (Tol-d_8, 253 K, 400 MHz) of **2b**:

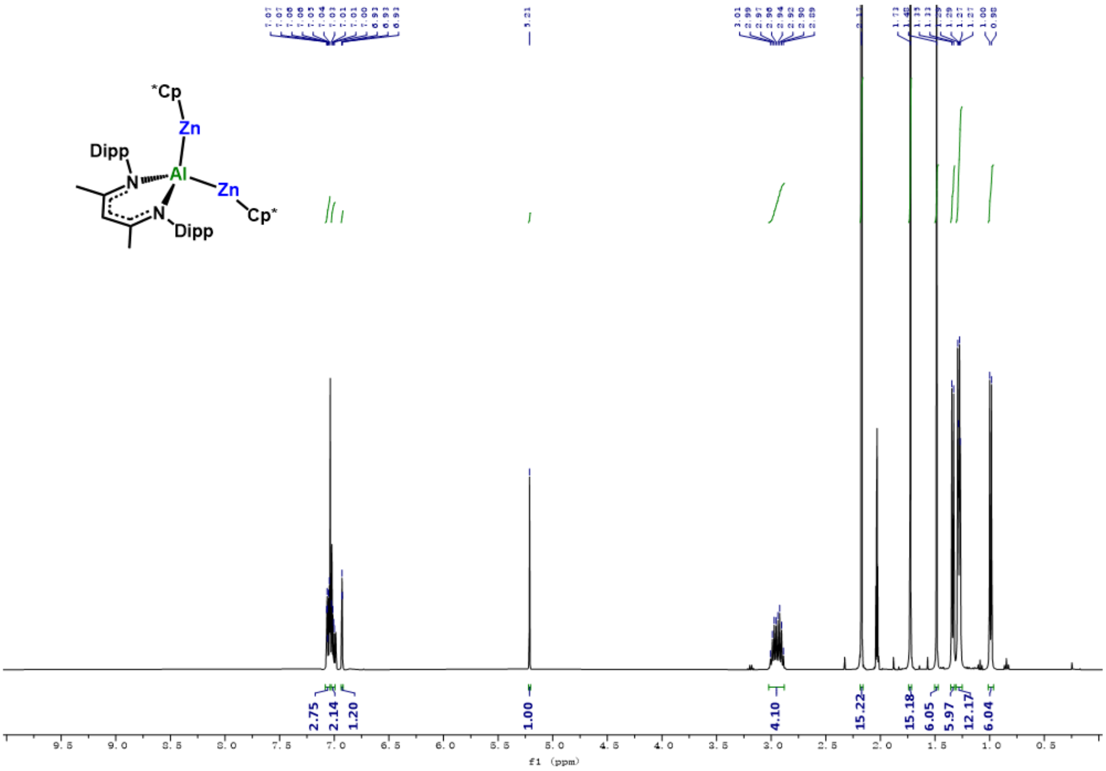

^{13}C NMR (Tol-d_8, 253 K, 101 MHz) of **2b**:

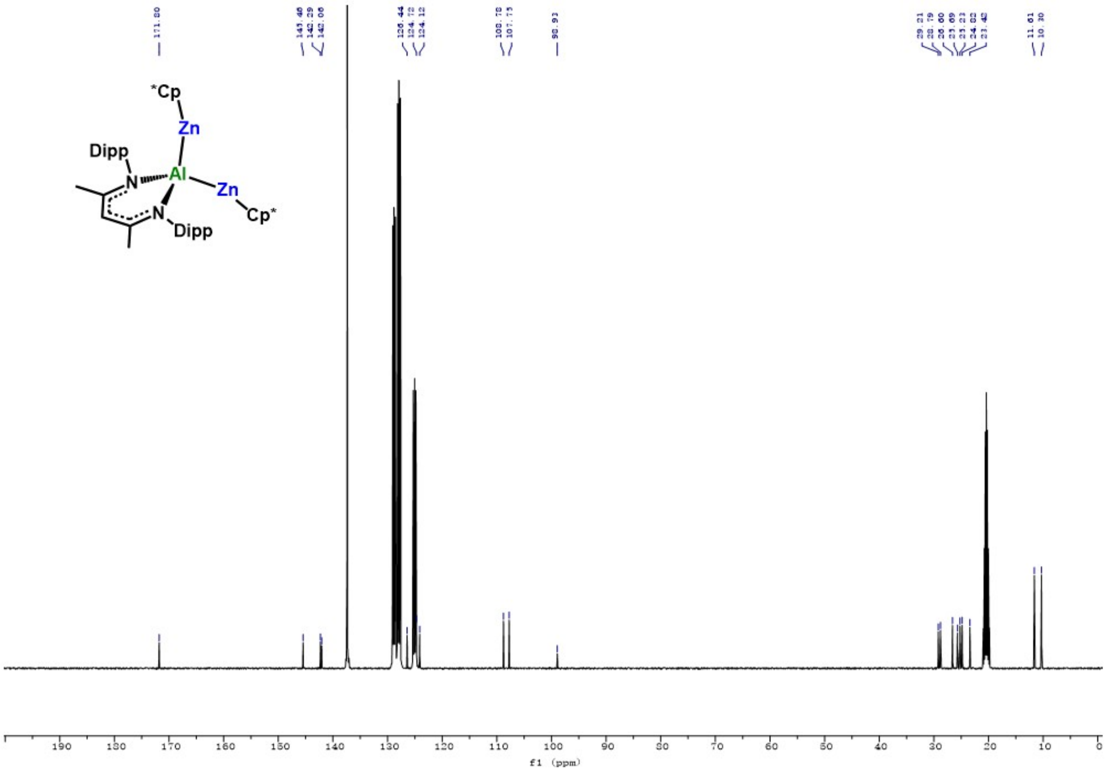

^{1}H NMR (C_6D_6, 298 K, 400 MHz) of **2c**: ("*"is the solvent peak of ^{Dipp}BDIGa(I), "*"is the solvent peak of Cp*-Zn-Zn-Cp*)

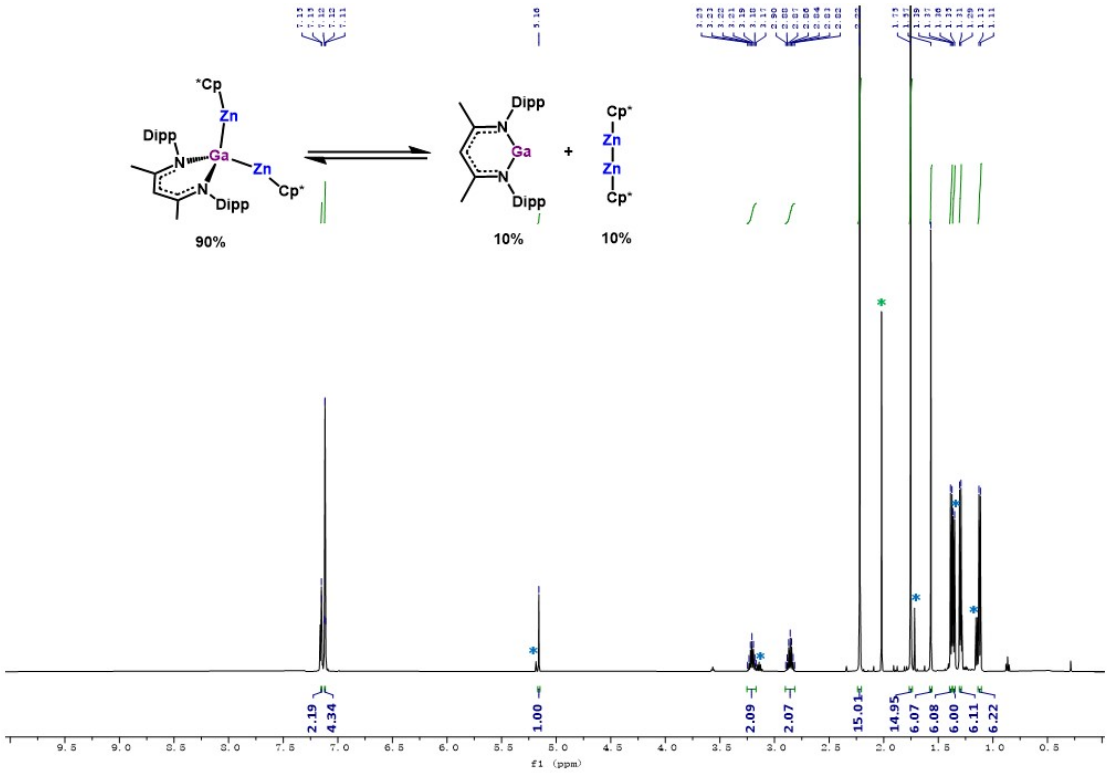

^{13}C NMR (C_6D_6, 298 K, 101 MHz) of **2b**: ("*"is the solvent peak of ^{Dipp}BDIGa(I)**,** "*"is the solvent peak of Cp*-Zn-Zn-Cp*)

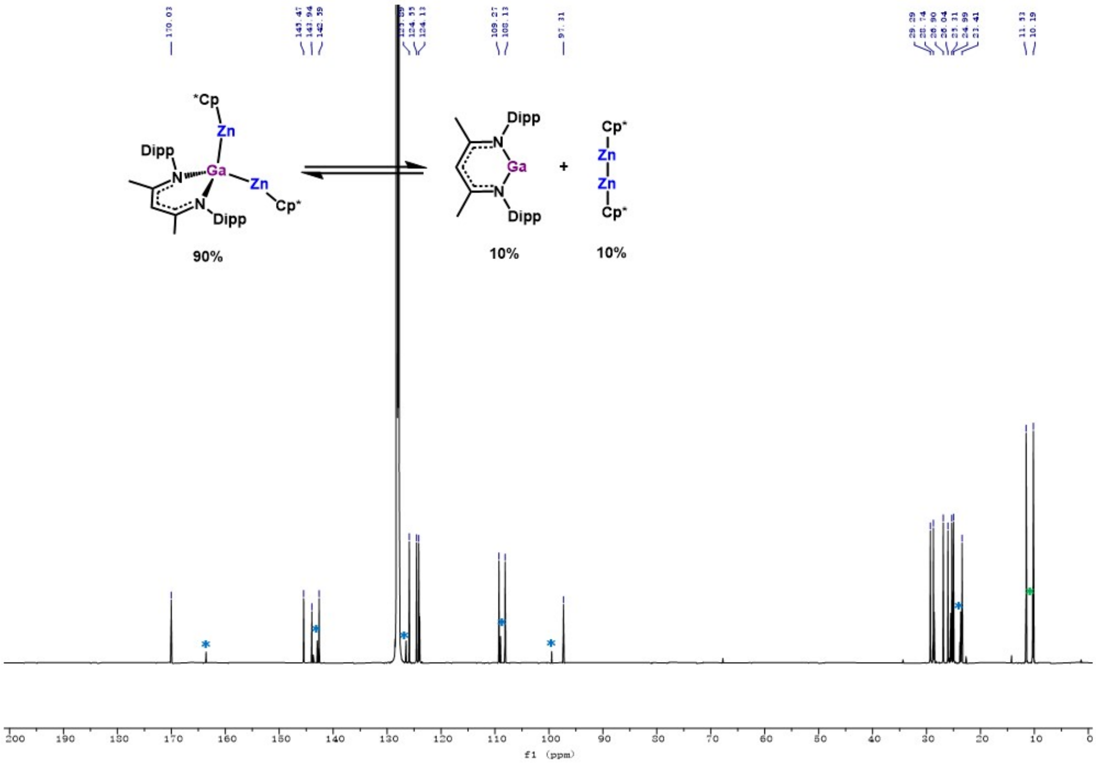

8) XYZ Coordinates

1b.log

SCF (wB97x) = -1241.27234485

E(SCF)+ZPE(0 K)= -1240.634623

H(298 K)= -1240.598514

G(298 K)= -1240.699904

Lowest Frequency = 25.4188cm-1

Al	3.861378	3.096800	4.872218
C	3.914910	5.132940	7.127897
N	4.072299	2.208704	6.663858
N	3.661123	4.849459	5.845385
C	4.276026	2.767025	7.862227
C	3.904713	0.785503	6.595138
C	2.595475	0.264645	6.619241
C	4.088405	6.834424	4.445824
C	6.433088	0.492718	6.371780
H	6.404886	1.531641	6.723536
C	3.794817	6.546898	7.621528
H	4.549561	7.191326	7.158099
H	3.917567	6.599935	8.703959
H	2.823655	6.975321	7.353466
C	0.857141	4.886863	5.222079
H	1.396023	3.923427	5.206115
C	6.255548	6.073835	3.474628
H	5.832130	5.082504	3.275992
H	7.332344	5.960143	3.637700
H	6.113429	6.679123	2.572186
C	5.021272	-0.055840	6.427661
C	4.268249	4.151812	8.063430
H	4.468165	4.495330	9.071958
C	4.494406	1.888032	9.058929
H	3.634275	1.230190	9.221472
H	4.660601	2.475600	9.962478
H	5.354546	1.226934	8.906460
C	5.583554	6.734984	4.678861
H	5.753299	6.083071	5.544209
C	6.943383	0.508421	4.930854
H	6.985031	-0.508497	4.524158
H	7.950674	0.934058	4.874256
H	6.285523	1.096384	4.280622
C	2.226187	7.904493	3.317402
H	1.849249	8.689461	2.667670
C	4.800540	-1.426383	6.274448
H	5.653118	-2.087429	6.133550
C	3.187112	5.903344	4.996186
C	1.811947	5.934552	4.675621
C	-0.389363	4.705673	4.366250
H	-1.050534	5.577734	4.418500
H	-0.967710	3.846802	4.719349
H	-0.138582	4.534230	3.315262
C	2.426485	-1.113887	6.466262
H	1.419900	-1.527957	6.479429
C	7.393134	-0.277005	7.275665
H	7.035994	-0.321900	8.309924
H	8.379860	0.196166	7.279924
H	7.531762	-1.307991	6.932817
C	1.375260	1.147921	6.803905
H	1.718674	2.180064	6.953791
C	3.581293	7.831293	3.608590
H	4.265015	8.555863	3.172008
C	6.228907	8.084361	4.982598
H	6.192178	8.755205	4.117744
H	7.283765	7.954897	5.242887
H	5.736476	8.598006	5.815241
C	0.571803	0.747768	8.040294
H	0.164556	-0.264630	7.941020
H	-0.273571	1.427496	8.189107
H	1.183054	0.767973	8.948017
C	3.516164	-1.956871	6.291803
H	3.365682	-3.025717	6.168259
C	1.354505	6.956707	3.840737
H	0.299873	7.005291	3.585447
C	0.469043	5.155850	6.676533
H	1.333056	5.136042	7.347118
H	-0.238453	4.397638	7.031377
H	-0.014371	6.134783	6.775524
C	0.490425	1.134512	5.558436
H	1.038287	1.482946	4.674400
H	-0.382409	1.782962	5.695319
H	0.123108	0.124647	5.343507

1c.log

SCF (wB97x) = -1241.35249909

E(SCF)+ZPE(0 K)= -1240.716093

H(298 K)= -1240.679252

G(298 K)= -1240.783490

Lowest Frequency = 21.9929cm-1

C	3.924812	5.134815	7.110045
N	4.102511	2.162585	6.672377
N	3.654311	4.883924	5.829350
C	4.296500	2.752812	7.851389
C	3.922806	0.749128	6.621082
C	2.608078	0.237562	6.631034
C	4.068176	6.872997	4.439203
C	6.445690	0.434319	6.417755
H	6.424120	1.458495	6.810119
C	3.809096	6.542781	7.632685
H	4.559298	7.196668	7.174626
H	3.941208	6.577758	8.714900
H	2.835154	6.975804	7.381989
C	0.849193	4.911124	5.226066
H	1.390665	3.951046	5.227741
C	6.234717	6.102166	3.474039
H	5.812932	5.107790	3.288132
H	7.311968	5.990080	3.635460
H	6.090286	6.693958	2.563020
C	5.031218	-0.107944	6.464572
C	4.288352	4.142901	8.034484
H	4.490742	4.488009	9.042386
C	4.501287	1.898189	9.072588
H	3.628662	1.260560	9.250391
H	4.675914	2.503725	9.962794
H	5.349657	1.217955	8.938477
C	5.563973	6.778802	4.670397
H	5.735646	6.138303	5.543402
C	6.941398	0.507085	4.973404
H	6.972900	-0.492095	4.524027
H	7.949699	0.931007	4.921382
H	6.280626	1.123348	4.352817
C	2.200600	7.924831	3.301697
H	1.819102	8.703693	2.647259
C	4.798527	-1.476043	6.309790
H	5.645454	-2.146450	6.178996
C	3.174524	5.936626	4.998081
C	1.799016	5.958915	4.670598
C	-0.398451	4.717758	4.373919
H	-1.062468	5.587964	4.420197
H	-0.974087	3.860348	4.735220
H	-0.150984	4.539355	3.323106
C	2.426065	-1.139021	6.476498
H	1.414904	-1.542639	6.479974
C	7.413529	-0.371617	7.279863
H	7.063128	-0.458808	8.313498
H	8.399727	0.102480	7.296597
H	7.551996	-1.387267	6.893812
C	1.397857	1.137415	6.799665
H	1.755976	2.161902	6.964454
C	3.555851	7.861816	3.595989
H	4.235451	8.588865	3.156618
C	6.209605	8.131787	4.955550
H	6.168810	8.793542	4.083792
H	7.265631	8.006759	5.213648
H	5.718802	8.652502	5.784578
C	0.561940	0.740441	8.015167
H	0.138063	-0.263341	7.898452
H	-0.273660	1.434416	8.153864
H	1.155554	0.741021	8.934581
C	3.508479	-1.994123	6.314282
H	3.348418	-3.061589	6.190507
C	1.335608	6.972989	3.829311
H	0.280722	7.012973	3.572760
C	0.460098	5.195771	6.677681
H	1.325678	5.186271	7.346130
H	-0.244988	4.439855	7.042368
H	-0.025831	6.174583	6.764539
C	0.536441	1.148059	5.537636
H	1.101749	1.496468	4.664499
H	-0.330224	1.807020	5.663164
H	0.160375	0.145160	5.305368
Ga	3.880332	3.042278	4.714282

1a.log

SCF (wB97x) = -1528.13183782

E(SCF)+ZPE(0 K)= -1527.505470

H(298 K)= -1527.469889

G(298 K)= -1527.571593

Lowest Frequency = 18.1564cm-1

Si	3.626805	1.845271	3.247121
N	2.723382	2.912966	4.298383

C	1.647031	4.963956	5.034715
H	1.472074	6.022670	4.885141
H	1.177473	4.486518	5.885423
N	4.114566	3.035418	2.037119
C	2.440640	4.294062	4.162725
C	3.068684	4.963662	3.044580
H	2.902379	6.034690	2.988802
C	3.837101	4.410208	2.073706
C	4.434431	5.257786	0.996708
H	4.185032	6.307939	1.153618
H	4.079709	4.964237	0.001814
H	5.525756	5.158834	0.971019
C	2.154216	2.253370	5.445384
C	2.870017	2.263998	6.657505
C	2.332377	1.572752	7.744678
H	2.876445	1.553495	8.686234
C	1.115007	0.907858	7.640244
H	0.713214	0.372612	8.496354
C	0.410507	0.935227	6.444139
H	-0.546219	0.421905	6.368634
C	0.909097	1.612630	5.326974
C	0.101184	1.633638	4.044888
H	0.626308	2.267877	3.319732
C	-1.279210	2.246890	4.271271
H	-1.874172	1.647866	4.969891
H	-1.836662	2.302399	3.330875
H	-1.200476	3.258746	4.679760
C	-0.010761	0.232367	3.446010
H	0.976711	-0.195014	3.238918
H	-0.576635	0.250503	2.508700
H	-0.526951	-0.449098	4.131835
C	4.202586	2.976231	6.776920
H	4.277847	3.680061	5.939755
C	4.316107	3.788758	8.062939
H	3.486316	4.494838	8.163361
H	5.249308	4.360251	8.072078
H	4.322295	3.149612	8.952606
C	5.357549	1.981693	6.654587
H	5.332825	1.251341	7.471560
H	6.324643	2.495018	6.691367
H	5.308642	1.419847	5.713846
C	4.893764	2.515039	0.943112
C	6.273355	2.300631	1.113927
C	6.988182	1.738330	0.051020
H	8.054468	1.555551	0.168130
C	6.361708	1.414296	-1.144010
H	6.934480	0.979100	-1.958315
C	4.999689	1.651065	-1.301147
H	4.516613	1.398339	-2.241560
C	4.241785	2.201919	-0.267173
C	2.748352	2.415414	-0.425518
H	2.447082	3.210881	0.268615
C	1.984292	1.151788	-0.026589
H	2.239542	0.319358	-0.691980
H	0.902375	1.311913	-0.082792
H	2.225738	0.836189	0.995697
C	2.350423	2.858949	-1.829371
H	2.920594	3.733277	-2.158674
H	1.288101	3.118168	-1.858151
H	2.504622	2.064962	-2.567803
C	7.001995	2.647643	2.397886
H	6.303418	3.182726	3.054766
C	7.451241	1.381085	3.126407
H	8.145867	0.801073	2.508110
H	6.601173	0.732584	3.365059
H	7.964119	1.628081	4.061697
C	8.191009	3.571796	2.141314
H	8.956926	3.080348	1.531566
H	8.663149	3.861699	3.084955
H	7.891855	4.485912	1.619303

2b.log

SCF (wB97x) = -2475.82901765

E(SCF)+ZPE(0 K)= -2474.746012

H(298 K)= -2474.679177

G(298 K)= -2474.845134

Lowest Frequency = 19.2913cm-1

Al	5.458513	9.700740	13.757083
Zn	7.091937	8.711750	15.373635
Zn	5.831331	10.983775	11.734187
N	4.269325	8.185428	13.381632
C	3.632600	7.657503	14.427165
C	3.432219	8.373729	15.626550
H	2.960985	7.818511	16.430112
C	3.550107	9.762939	15.832791
N	4.186019	10.572802	14.980016

C	3.103000	6.256418	14.346718	H	1.774891	14.407174	14.173478
H	2.571135	6.085365	13.405939	C	2.822892	12.572061	14.544694
H	2.437837	6.026679	15.180094	C	6.312669	12.263593	16.159885
H	3.934676	5.540440	14.366566	H	6.183009	11.182814	16.319642
C	2.904096	10.341962	17.056829	C	6.648560	12.898574	17.507855
H	3.547522	11.096380	17.521241	H	5.853698	12.739596	18.243539
H	2.675768	9.563513	17.787077	H	7.572504	12.475238	17.913984
H	1.967045	10.849555	16.805546	H	6.810055	13.977686	17.419422
C	4.314718	7.528954	12.107725	C	7.470019	12.457117	15.178724
C	3.432876	7.965193	11.089780	H	7.237867	12.047957	14.187778
C	3.536576	7.384424	9.823984	H	7.688727	13.524536	15.046951
H	2.867991	7.706525	9.032129	H	8.382231	11.967980	15.538934
C	4.495596	6.418827	9.544483	C	1.702230	11.762910	13.923589
H	4.567509	5.991559	8.548142	H	1.980649	10.701157	13.945300
C	5.364560	6.016393	10.544170	C	1.515125	12.171300	12.463856
H	6.129201	5.275249	10.323704	H	0.703198	11.599715	12.002965
C	5.291236	6.550468	11.835662	H	1.252659	13.231904	12.381510
C	2.399390	9.043405	11.353278	H	2.427911	12.010778	11.879689
H	2.891773	9.803754	11.980280	C	0.380445	11.929601	14.673619
C	1.924106	9.718970	10.071785	H	-0.396235	11.296312	14.232376
H	2.764744	10.079230	9.469718	H	0.461226	11.667726	15.732650
H	1.335345	9.036486	9.449198	H	0.026974	12.965623	14.621842
H	1.281725	10.572375	10.297200	C	8.879057	7.718760	16.357430
C	1.197348	8.510439	12.136586	C	7.803121	6.795213	16.542389
H	1.474148	8.150809	13.131599	C	6.845126	7.401285	17.401405
H	0.446285	9.297422	12.271583	C	7.326808	8.685388	17.772670
H	0.717999	7.684428	11.598105	C	8.582400	8.891634	17.127891
C	6.310154	6.082011	12.854901	C	10.161470	7.459868	15.637029
H	6.030072	6.472528	13.844774	H	10.053784	6.678567	14.877155
C	6.354967	4.555941	12.934284	H	10.536674	8.354469	15.127494
H	7.077662	4.221537	13.682301	H	10.956576	7.129265	16.319401
H	5.378147	4.134734	13.195453	C	7.745013	5.388564	16.054984
H	6.654215	4.113036	11.979012	H	6.725043	5.086277	15.787783
C	7.689126	6.650372	12.516440	H	8.376744	5.230335	15.174729
H	8.425044	6.376880	13.281610	H	8.089617	4.679602	16.820324
H	8.044330	6.268760	11.551621	C	5.584230	6.769739	17.888808
H	7.666406	7.744709	12.453291	H	5.109247	6.147192	17.120716
C	4.005114	11.993151	15.057674	H	5.756144	6.121157	18.759484
C	5.026043	12.817221	15.581121	H	4.848001	7.522701	18.190450
C	4.852393	14.203913	15.552474	C	6.633467	9.611925	18.716164
H	5.646283	14.841604	15.933874	H	7.191436	10.540917	18.856866
C	3.693838	14.782197	15.055104	H	5.625880	9.890008	18.376700
H	3.576847	15.862236	15.048974	H	6.510766	9.159882	19.708666
C	2.688072	13.962861	14.564847	C	9.514201	10.043017	17.314943

H	8.993009	10.944130	17.654313
H	10.289706	9.825056	18.062028
H	10.038662	10.303848	16.388458
C	7.384880	11.130739	10.042537
C	7.699327	12.163671	10.974337
C	6.579628	13.044033	11.055351
C	5.569315	12.559618	10.161004
C	6.075320	11.376131	9.527246
C	8.276110	10.001300	9.642961
H	8.982022	9.740290	10.438704
H	8.874300	10.239931	8.752978
H	7.703660	9.096654	9.406400
C	8.977615	12.267732	11.740735
H	8.922025	13.034691	12.519043
H	9.823822	12.529236	11.092839
H	9.242139	11.324973	12.237840
C	6.457496	14.269805	11.900166
H	6.662029	15.186979	11.331991
H	7.153318	14.253944	12.745159
H	5.451290	14.374673	12.322845
C	4.277362	13.246239	9.861205
H	3.494569	12.542689	9.555531
H	4.381266	13.979787	9.050348
H	3.897405	13.790855	10.733054
C	5.418725	10.594312	8.437082
H	5.405614	9.516144	8.638757
H	5.936440	10.730469	7.478597
H	4.382279	10.909361	8.283173

2c.log

SCF (wB97x) = -2475.88416430
E(SCF)+ZPE(0 K)= -2474.802351
H(298 K)= -2474.734986
G(298 K)= -2474.901804
Lowest Frequency = 21.3705cm-1

Zn	7.093823	8.690518	15.402637
Zn	5.816794	10.973199	11.700712
N	4.265148	8.156145	13.369882
C	3.640549	7.646323	14.425561
C	3.441690	8.372854	15.620310
H	2.966793	7.819514	16.423495
C	3.541258	9.765776	15.827318
N	4.176718	10.591918	14.998160
C	3.109144	6.241641	14.365174
H	2.568576	6.061497	13.430787
H	2.450017	6.019799	15.205774
H	3.940083	5.524704	14.384553
C	2.857280	10.326523	17.042929
H	3.452412	11.123225	17.500678
H	2.670499	9.545695	17.783019
H	1.891003	10.770117	16.780172
C	4.311924	7.502490	12.103856
C	3.438308	7.948691	11.081524
C	3.551078	7.381981	9.810288
H	2.887960	7.712708	9.017011
C	4.512269	6.419000	9.527381
H	4.590750	6.001326	8.527421
C	5.375322	6.007240	10.529186
H	6.142321	5.268898	10.306195
C	5.293345	6.529357	11.824623
C	2.405221	9.025156	11.354118
H	2.895117	9.773388	11.997983
C	1.938172	9.720464	10.080449
H	2.782725	10.092652	9.490970
H	1.356630	9.046718	9.441683
H	1.291546	10.568532	10.314499
C	1.198732	8.482785	12.123883
H	1.474278	8.099457	13.110186
H	0.452404	9.271189	12.276688
H	0.715098	7.671676	11.566831
C	6.310197	6.062349	12.846411
H	6.020622	6.447275	13.835566
C	6.364777	4.536763	12.923021
H	7.086195	4.205078	13.673944
H	5.389179	4.109714	13.178675
H	6.671200	4.098046	11.967973
C	7.687747	6.640096	12.517487
H	8.422796	6.367476	13.284012
H	8.049482	6.265381	11.552405
H	7.659262	7.734471	12.457858
C	3.993588	12.004481	15.074541
C	5.024208	12.831799	15.577165
C	4.860319	14.218827	15.534113
H	5.662519	14.855282	15.900818
C	3.701170	14.799789	15.040324
H	3.591495	15.880464	15.021453

C	2.685266	13.980458	14.570790
H	1.771500	14.425937	14.181605
C	2.809749	12.588576	14.567299
C	6.311202	12.276181	16.153504
H	6.181290	11.194787	16.307878
C	6.642102	12.904673	17.505880
H	5.848137	12.733228	18.239667
H	7.570228	12.488287	17.910291
H	6.791905	13.986016	17.423811
C	7.471490	12.477038	15.177044
H	7.239864	12.081927	14.180487
H	7.694127	13.545058	15.057780
H	8.382229	11.981789	15.533133
C	1.679421	11.780691	13.962407
H	1.941372	10.715689	14.011587
C	1.506210	12.154853	12.491839
H	0.692198	11.580359	12.037932
H	1.256372	13.216296	12.382719
H	2.421667	11.970739	11.918920
C	0.356649	11.986316	14.700755
H	-0.423732	11.342374	14.281832
H	0.433483	11.766406	15.769658
H	0.010825	13.022088	14.607817
C	8.884234	7.717859	16.369656
C	7.804668	6.794576	16.544780
C	6.840502	7.400873	17.399614
C	7.320763	8.685952	17.773989
C	8.582041	8.891339	17.137589
C	10.169474	7.458367	15.654957
H	10.061383	6.687590	14.884610
H	10.555167	8.356601	15.160288
H	10.956105	7.113006	16.339518
C	7.750701	5.387451	16.057706
H	6.732287	5.083428	15.787523
H	8.385462	5.229919	15.179642
H	8.094560	4.680893	16.825328
C	5.580436	6.766599	17.885058
H	5.108888	6.142049	17.116992
H	5.755381	6.120440	18.756689
H	4.841687	7.517743	18.183822
C	6.620849	9.614034	18.711151
H	7.175040	10.545568	18.849181
H	5.613791	9.886109	18.366227
H	6.497353	9.165097	19.704780
C	9.509309	10.045857	17.327177
H	8.982332	10.947546	17.655467
H	10.276689	9.833360	18.083814
H	10.042982	10.301891	16.404822
C	7.384941	11.132987	10.026861
C	7.705341	12.157724	10.965046
C	6.588363	13.039287	11.060396
C	5.570892	12.564838	10.167688
C	6.072096	11.383470	9.519951
C	8.271842	10.004382	9.615215
H	8.985940	9.741853	10.402995
H	8.860452	10.245649	8.719747
H	7.697496	9.099985	9.382606
C	8.984593	12.245728	11.732056
H	8.942367	13.019998	12.503750
H	9.835590	12.485849	11.082508
H	9.232105	11.302556	12.237491
C	6.471348	14.259144	11.914686
H	6.639375	15.181156	11.342650
H	7.195856	14.253632	12.734977
H	5.478545	14.344698	12.372376
C	4.285010	13.264257	9.871181
H	3.496687	12.570054	9.559028
H	4.398183	14.002162	9.065653
H	3.908954	13.806148	10.746206
C	5.414033	10.621519	8.416632
H	5.409761	9.539341	8.594288
H	5.926003	10.783677	7.459202
H	4.374670	10.932569	8.275578
Ga	5.585577	9.713473	13.735014

2a.log

SCF (wB97x) = -2762.68019368

E(SCF)+ZPE(0 K)= -2761.607328

H(298 K)= -2761.542269

G(298 K)= -2761.702505

Lowest Frequency = 17.0477cm-1

Si	3.147234	11.907381	2.779823
Zn	2.930317	12.184202	5.126116
Zn	2.792356	9.996138	1.528789
N	2.171388	13.252832	2.094736
C	2.585686	14.499284	2.553787

C	3.861132	14.717234	3.004382	C	7.781592	9.969091	0.946209
H	4.061722	15.699004	3.420606	H	8.515066	9.280808	0.534678
C	5.023536	13.839830	2.920568	C	7.303884	11.026332	0.180803
N	4.784854	12.534750	2.461978	H	7.679801	11.167797	-0.830492
C	1.565818	15.594361	2.588273	C	6.334167	11.901820	0.673089
H	1.186383	15.818610	1.584266	C	6.023873	10.536157	4.265889
H	1.989152	16.509652	3.006500	H	5.449606	11.425554	4.564751
H	0.691827	15.306221	3.186800	C	7.272004	10.476196	5.145841
C	6.265145	14.280305	3.258552	H	6.995740	10.352505	6.198015
H	7.131981	13.630751	3.195059	H	7.872395	11.386872	5.058186
H	6.419814	15.316230	3.538479	H	7.910488	9.626496	4.882325
C	0.939177	13.095653	1.377300	C	5.160038	9.299188	4.507803
C	0.931997	13.215074	-0.035972	H	4.261009	9.296158	3.879271
C	-0.284070	13.056043	-0.707979	H	4.841315	9.238687	5.554229
H	-0.312849	13.148853	-1.788762	H	5.720790	8.385842	4.276051
C	-1.460328	12.763440	-0.028848	C	5.852601	13.037058	-0.203917
H	-2.389225	12.634522	-0.577675	H	4.966494	13.472759	0.273900
C	-1.436837	12.626484	1.349325	C	5.455683	12.555691	-1.596455
H	-2.353381	12.378860	1.878917	H	4.670793	11.791393	-1.557016
C	-0.252753	12.797376	2.074162	H	5.083852	13.392176	-2.197833
C	2.203499	13.472010	-0.828625	H	6.306774	12.128321	-2.137991
H	2.969605	12.799055	-0.410344	C	6.915243	14.131883	-0.307176
C	2.049989	13.165305	-2.316158	H	7.198500	14.501417	0.682584
H	3.021581	13.217676	-2.813276	H	7.817530	13.754034	-0.803367
H	1.629216	12.174777	-2.505464	H	6.542809	14.979160	-0.893617
H	1.403178	13.901638	-2.806662	C	1.990991	12.239576	7.141680
C	2.728725	14.904432	-0.692097	C	2.024308	13.610742	6.729297
H	3.596412	15.046906	-1.346687	C	3.386622	14.000844	6.604913
H	1.963137	15.626019	-1.001426	C	4.207604	12.892949	6.950386
H	3.047655	15.149066	0.322320	C	3.354103	11.792651	7.279988
C	-0.288307	12.597157	3.576290	C	0.785183	11.456499	7.545139
H	0.617914	13.051763	4.003123	H	-0.129848	11.848965	7.088355
C	-1.492624	13.263333	4.236163	H	0.631939	11.478967	8.632485
H	-1.514515	14.342755	4.053848	H	0.861767	10.400927	7.261054
H	-1.468383	13.105340	5.318788	C	0.858102	14.523994	6.544845
H	-2.439333	12.845850	3.879018	H	0.835970	14.991224	5.551301
C	-0.268094	11.103307	3.909358	H	0.876553	15.344728	7.272717
H	-1.185145	10.619154	3.551343	H	-0.092556	14.002080	6.678756
H	-0.197941	10.939656	4.990656	C	3.883682	15.364430	6.264243
H	0.577399	10.589926	3.435176	H	4.809521	15.321319	5.679040
C	5.854264	11.716379	1.990698	H	4.098752	15.951039	7.168294
C	6.391058	10.693889	2.802738	H	3.150234	15.930693	5.680673
C	7.326115	9.812234	2.247280	C	5.697415	12.941256	7.021854
H	7.715821	9.000094	2.858230	H	6.125697	11.967791	7.271978

H	6.032972	13.645829	7.793544
H	6.149884	13.270272	6.076335
C	3.772615	10.474824	7.844188
H	3.158829	9.647328	7.470573
H	3.689270	10.458326	8.939170
H	4.814830	10.240818	7.602328
C	1.960119	7.893728	1.796683
C	3.300577	7.808002	1.302811
C	3.349420	8.458674	0.027895
C	2.032135	8.945035	-0.267226
C	1.175680	8.596141	0.828439
C	1.470323	7.336247	3.093033
H	2.223866	7.416299	3.884837
H	1.208636	6.273702	3.011950
H	0.574971	7.857899	3.446434
C	4.440299	7.078788	1.932123
H	5.379003	7.642704	1.865119
H	4.613553	6.111994	1.441685
H	4.256616	6.872683	2.990531
C	4.547882	8.518379	-0.859963
H	4.469064	9.317974	-1.602948
H	4.685110	7.579998	-1.413223
H	5.468679	8.694707	-0.292543
C	1.592091	9.606084	-1.529176
H	0.829088	10.371726	-1.343774
H	1.156880	8.884980	-2.233363
H	2.427218	10.091365	-2.044946
C	-0.288363	8.882184	0.893282
H	-0.675265	8.789990	1.912373
H	-0.863632	8.187014	0.268134
H	-0.527845	9.894922	0.546562

Al-Int-1.log

SCF (wB97x) = -2475.80282378

E(SCF)+ZPE(0 K)= -2474.720690

H(298 K)= -2474.653839

G(298 K)= -2474.819505

Lowest Frequency = 18.7087cm-1

Al	4.462844	10.245398	13.130321
Zn	7.464093	9.129777	14.717220
Zn	6.919779	10.363806	12.709207
N	3.683533	8.451699	13.279166
C	3.293178	7.889821	14.428664
C	3.264561	8.585874	15.649700
H	2.967485	8.007241	16.517508
C	3.403784	9.966619	15.849806
N	3.783324	10.800128	14.876064
C	2.822410	6.466546	14.442450
H	2.109389	6.280360	13.633303
H	2.355672	6.213417	15.394857
H	3.660127	5.780054	14.283707
C	3.089804	10.516497	17.208397
H	2.367148	11.335974	17.150997
H	3.995434	10.936164	17.661229
H	2.701353	9.745044	17.874101
C	3.572234	7.715645	12.045837
C	2.551946	8.083971	11.135762
C	2.448348	7.383008	9.932679
H	1.667398	7.647533	9.227117
C	3.330663	6.358751	9.613191
H	3.233238	5.827971	8.670485
C	4.347894	6.037122	10.496033
H	5.062786	5.260975	10.232105
C	4.495006	6.702329	11.717824
C	1.577590	9.204580	11.449195
H	2.161939	10.023013	11.906799
C	0.916235	9.787595	10.206686
H	1.654471	10.077464	9.452491
H	0.221337	9.078152	9.744622
H	0.335875	10.675973	10.467616
C	0.518589	8.776055	12.465987
H	0.955037	8.490880	13.428426
H	-0.187438	9.593109	12.654455
H	-0.055236	7.922008	12.088329
C	5.694512	6.346101	12.573874
H	5.626189	6.882062	13.532706
C	5.778331	4.845737	12.856078
H	6.634444	4.618667	13.499439
H	4.880212	4.454479	13.344644
H	5.918086	4.275955	11.931495
C	6.971233	6.811756	11.873681
H	7.850951	6.646863	12.504103
H	7.119581	6.266884	10.933007
H	6.928825	7.878868	11.631375
C	3.620966	12.219899	15.044978
C	4.633422	13.008223	15.622125

C	4.450426	14.393824	15.668592
H	5.235095	15.012700	16.098789
C	3.296478	14.990002	15.181626
H	3.174057	16.068196	15.231674
C	2.297097	14.196822	14.633616
H	1.384907	14.660153	14.264478
C	2.439746	12.810688	14.544262
C	5.911598	12.428779	16.191571
H	5.851044	11.330469	16.143423
C	6.093122	12.844614	17.652263
H	5.241528	12.553126	18.276016
H	6.993623	12.394587	18.081822
H	6.204554	13.930449	17.742374
C	7.121150	12.860209	15.364681
H	7.008685	12.579710	14.311556
H	7.254908	13.948529	15.401354
H	8.038833	12.394207	15.739950
C	1.307579	11.986241	13.962884
H	1.676103	10.965369	13.801499
C	0.837327	12.524277	12.615181
H	0.027960	11.901948	12.218833
H	0.445310	13.543394	12.695927
H	1.649818	12.536482	11.879740
C	0.139511	11.892419	14.945255
H	-0.670022	11.281754	14.531165
H	0.442685	11.442431	15.896070
H	-0.269872	12.885170	15.164402
C	9.494526	8.398504	15.792893
C	8.710482	7.242726	15.516781
C	7.475753	7.366402	16.222108
C	7.507277	8.600590	16.949810
C	8.762654	9.231097	16.686511
C	10.880537	8.652333	15.300597
H	11.057316	8.199476	14.318898
H	11.094020	9.722819	15.207537
H	11.641007	8.239093	15.977795
C	9.175427	6.067576	14.724222
H	8.344952	5.423529	14.418334
H	9.710835	6.363167	13.813526
H	9.866845	5.437941	15.300374
C	6.384603	6.350112	16.310211
H	6.420741	5.637443	15.479617
H	6.445039	5.761161	17.235974
H	5.389562	6.814087	16.296596
C	6.459043	9.050057	17.913772
H	6.482509	10.133682	18.075521
H	5.450963	8.793586	17.563596
H	6.578678	8.580966	18.900412
C	9.283659	10.483517	17.306660
H	8.479611	11.097020	17.723983
H	9.979750	10.272619	18.129740
H	9.830540	11.107130	16.588637
C	8.426440	10.368298	11.092007
C	8.484488	11.624000	11.806326
C	7.371274	12.413636	11.373829
C	6.650747	11.672809	10.406852
C	7.276119	10.412264	10.242793
C	9.493380	9.322150	11.127026
H	9.816540	9.099861	12.153431
H	10.391154	9.627961	10.572343
H	9.151699	8.377661	10.690757
C	9.622837	12.057211	12.672746
H	9.344904	12.892581	13.324417
H	10.491676	12.383015	12.084084
H	9.970195	11.245171	13.325286
C	7.017603	13.790398	11.836390
H	6.998665	14.502942	11.002105
H	7.737415	14.173119	12.566717
H	6.024814	13.838586	12.306984
C	5.339467	12.059848	9.828059
H	4.503488	11.738710	10.484113
H	5.165901	11.597033	8.850278
H	5.242904	13.144301	9.707405
C	6.799167	9.338871	9.317743
H	7.465885	8.470972	9.318454
H	6.741598	9.697798	8.282381
H	5.794869	8.972076	9.575031

Ga-Int-1.log

SCF (wB97x) = -2475.87510103

E(SCF)+ZPE(0 K)= -2474.793047

H(298 K)= -2474.725734

G(298 K)= -2474.892988

Lowest Frequency = 21.0509cm-1

Zn	7.420409	9.241760	14.660767
Zn	7.180832	10.476210	12.692356

N	3.612797	8.429817	13.232848
C	3.337555	7.889493	14.415987
C	3.339808	8.609240	15.627641
H	3.129145	8.020825	16.515095
C	3.378824	9.997268	15.823363
N	3.625401	10.873843	14.851540
C	2.964270	6.434188	14.520014
H	2.380465	6.103029	13.656943
H	2.395556	6.238339	15.430645
H	3.864671	5.810965	14.556339
C	3.078193	10.504212	17.208903
H	2.206376	11.166566	17.207623
H	3.913935	11.101549	17.590580
H	2.893586	9.684990	17.905707
C	3.489468	7.665607	12.034010
C	2.420067	7.968057	11.152945
C	2.336297	7.281394	9.940530
H	1.519445	7.500192	9.259689
C	3.278838	6.325303	9.581540
H	3.196938	5.806213	8.630736
C	4.326328	6.047552	10.444503
H	5.075470	5.310415	10.161773
C	4.455129	6.702076	11.673727
C	1.358087	8.986051	11.528604
H	1.873492	9.843736	11.989509
C	0.586263	9.525421	10.331131
H	1.255108	9.904850	9.552488
H	-0.054185	8.759107	9.880780
H	-0.068287	10.344716	10.640468
C	0.390348	8.431917	12.576037
H	0.897472	8.165943	13.508552
H	-0.380041	9.173392	12.818690
H	-0.116471	7.536230	12.198630
C	5.664706	6.372135	12.526116
H	5.588588	6.915296	13.479442
C	5.760644	4.874723	12.822137
H	6.601173	4.660463	13.491406
H	4.851453	4.478489	13.284519
H	5.932516	4.303152	11.903615
C	6.938971	6.843036	11.829465
H	7.821333	6.670913	12.455004
H	7.088939	6.306108	10.884348
H	6.897996	7.912141	11.599036
C	3.319894	12.250659	15.066524
C	4.271870	13.135466	15.609927
C	3.939734	14.487890	15.725482
H	4.674404	15.179725	16.132802
C	2.694974	14.962862	15.333902
H	2.453812	16.017066	15.438112
C	1.760808	14.079366	14.808447
H	0.781623	14.447679	14.508818
C	2.052657	12.722241	14.655776
C	5.646578	12.669290	16.042589
H	5.658159	11.569141	16.021578
C	5.978985	13.124974	17.462210
H	5.237770	12.776803	18.189560
H	6.958826	12.750688	17.776349
H	6.018487	14.217155	17.533621
C	6.705709	13.157588	15.057211
H	6.449202	12.875252	14.029312
H	6.793530	14.250736	15.082536
H	7.691920	12.734025	15.285509
C	0.993738	11.786889	14.103699
H	1.473715	10.823103	13.892163
C	0.392909	12.298536	12.798016
H	-0.351433	11.589795	12.418495
H	-0.117641	13.258524	12.930497
H	1.158478	12.431862	12.025848
C	-0.103999	11.530021	15.136672
H	-0.861213	10.844694	14.740550
H	0.296985	11.087071	16.053891
H	-0.609865	12.462624	15.412505
C	9.474211	8.566203	15.729285
C	8.683750	7.411001	15.475139
C	7.436780	7.564423	16.157090
C	7.469266	8.827141	16.848169
C	8.735457	9.438065	16.577302
C	10.864825	8.785092	15.235115
H	11.013033	8.373915	14.229852
H	11.124720	9.847375	15.192853
H	11.608676	8.301854	15.882972
C	9.144545	6.202471	14.732462
H	8.307924	5.572356	14.414809
H	9.720946	6.457378	13.835368
H	9.796601	5.573189	15.352841
C	6.369251	6.529931	16.297328
H	6.367709	5.827552	15.456344
H	6.502448	5.932062	17.209318

H	5.370682	6.978539	16.349975
C	6.432821	9.304470	17.810752
H	6.426480	10.396059	17.907034
H	5.426807	8.997237	17.503459
H	6.598380	8.898802	18.818195
C	9.226678	10.736224	17.124770
H	8.400415	11.371824	17.457570
H	9.886352	10.594632	17.990789
H	9.797865	11.309198	16.384570
C	8.765605	10.071116	10.920105
C	9.297049	11.141053	11.691883
C	8.358707	12.209999	11.692094
C	7.226647	11.801806	10.913858
C	7.480228	10.464261	10.439870
C	9.460686	8.772841	10.670925
H	9.757441	8.270795	11.601964
H	10.377680	8.911305	10.084817
H	8.828657	8.072239	10.118152
C	10.620187	11.159244	12.380205
H	11.371848	11.729876	11.817916
H	11.021340	10.150283	12.516651
H	10.558391	11.621097	13.373777
C	8.596982	13.565936	12.267352
H	9.246526	14.167279	11.617279
H	9.089504	13.520373	13.245673
H	7.667836	14.128751	12.396782
C	6.080378	12.657328	10.497486
H	5.163385	12.070309	10.373686
H	6.278113	13.158686	9.540439
H	5.862422	13.439209	11.233557
C	6.572403	9.712280	9.520962
H	6.871274	8.665187	9.411901
H	6.558734	10.151054	8.514733
H	5.531882	9.711784	9.876707
Ga	4.292355	10.460773	12.849993

Si-Int-1.log

SCF (wB97x) = -2762.65278424

E(SCF)+ZPE(0 K)= -2761.580042

H(298 K)= -2761.514766

G(298 K)= -2761.675150

Lowest Frequency = 21.0685cm-1

Si	3.617423	12.030335	1.470853
Zn	2.414157	11.350012	4.829141
Zn	2.348585	10.157588	2.780368
N	2.674199	13.544098	1.549031
C	3.113186	14.682161	2.232089
C	4.328410	14.749805	2.844464
H	4.556261	15.678755	3.358769
C	5.417958	13.797499	2.791475
N	5.146231	12.573399	2.149476
C	2.212441	15.875200	2.257707
H	1.868980	16.137978	1.250715
H	2.734102	16.735078	2.680521
H	1.316814	15.697968	2.859366
C	6.642694	14.104487	3.290824
H	7.485678	13.428084	3.221786
H	6.800816	15.068898	3.759068
C	1.467349	13.581569	0.751966
C	1.593360	13.637029	-0.661674
C	0.431987	13.635049	-1.437220
H	0.512426	13.674760	-2.518259
C	-0.828807	13.585349	-0.857865
H	-1.718216	13.584068	-1.481699
C	-0.939375	13.534624	0.520301
H	-1.925089	13.485063	0.977498
C	0.189014	13.528256	1.349024
C	2.945572	13.731354	-1.348546
H	3.616625	13.008725	-0.857363
C	2.902618	13.348321	-2.822839
H	3.915018	13.316424	-3.232420
H	2.446881	12.365121	-2.977824
H	2.342512	14.077969	-3.417728
C	3.570173	15.117692	-1.180942
H	4.531839	15.169693	-1.704258
H	2.917924	15.889493	-1.606007
H	3.752823	15.367250	-0.131569
C	-0.061894	13.430952	2.842136
H	0.901322	13.496386	3.370412
C	-0.982896	14.549427	3.338145
H	-0.621696	15.550153	3.085040
H	-1.101730	14.499609	4.425680
H	-1.984416	14.449520	2.906450
C	-0.701231	12.085619	3.181003
H	-1.673549	11.982229	2.685006
H	-0.863009	11.987958	4.257308

H	-0.075036	11.249989	2.860646
C	6.270832	11.730751	1.827006
C	6.739596	10.774899	2.745588
C	7.783394	9.929730	2.355103
H	8.139667	9.175307	3.053855
C	8.374099	10.040912	1.104902
H	9.183209	9.374012	0.819914
C	7.938465	11.026640	0.229174
H	8.423238	11.140032	-0.738192
C	6.892377	11.887521	0.569082
C	6.188830	10.638615	4.148628
H	5.401959	11.394318	4.287710
C	7.290191	10.901770	5.176106
H	6.914362	10.783797	6.198053
H	7.690477	11.915820	5.079748
H	8.120660	10.197106	5.056384
C	5.568462	9.260395	4.355658
H	4.778814	9.069082	3.622431
H	5.127709	9.167236	5.352950
H	6.316619	8.465009	4.248199
C	6.518095	12.994712	-0.394132
H	5.612109	13.486902	-0.019906
C	6.220810	12.461057	-1.791986
H	5.403315	11.730257	-1.781751
H	5.939299	13.279757	-2.463042
H	7.096101	11.972457	-2.233326
C	7.620744	14.053729	-0.437110
H	7.820179	14.452671	0.562171
H	8.554748	13.632241	-0.827061
H	7.334244	14.887174	-1.087567
C	1.726666	10.931398	7.069906
C	1.069388	12.113886	6.623847
C	2.070597	13.071634	6.270787
C	3.354175	12.482390	6.529752
C	3.133604	11.160364	7.032065
C	1.057143	9.700667	7.584063
H	0.078819	9.541941	7.118463
H	0.887305	9.748324	8.668453
H	1.654638	8.800994	7.400271
C	-0.400659	12.351965	6.704426
H	-0.716473	13.201768	6.091539
H	-0.707789	12.575866	7.734786
H	-0.987021	11.481162	6.388645
C	1.856220	14.495226	5.873351

H	2.579312	14.820006	5.114728
H	1.966607	15.174611	6.729553
H	0.855053	14.662238	5.463153
C	4.653587	13.212116	6.446185
H	5.508968	12.531371	6.414971
H	4.798655	13.869420	7.314471
H	4.717923	13.843751	5.552671
C	4.163191	10.231128	7.580273
H	3.964852	9.184459	7.321129
H	4.199102	10.278785	8.676806
H	5.167234	10.473620	7.220847
C	2.289177	7.838022	2.820427
C	2.885183	8.289090	1.599540
C	1.874059	8.977509	0.850412
C	0.667862	8.963276	1.612011
C	0.924668	8.262943	2.827291
C	2.936662	7.021841	3.891632
H	3.999059	6.858267	3.689995
H	2.469716	6.033232	3.980450
H	2.867641	7.492190	4.881814
C	4.270954	8.044781	1.098152
H	4.743560	8.969959	0.735679
H	4.277773	7.340712	0.256004
H	4.926877	7.631173	1.870230
C	2.049611	9.531827	-0.517890
H	1.209957	10.174507	-0.805373
H	2.132301	8.739287	-1.273035
H	2.964524	10.139771	-0.594383
C	-0.662614	9.437468	1.126594
H	-1.388930	9.509158	1.941576
H	-1.081485	8.745603	0.384267
H	-0.613117	10.424092	0.649126
C	-0.059063	8.006262	3.920690
H	0.445542	7.774028	4.864269
H	-0.722954	7.160686	3.696895
H	-0.705207	8.873820	4.106933

Al-TS-1.log

SCF (wB97x) = -2475.79734068

E(SCF)+ZPE(0 K)= -2474.715001

H(298 K)= -2474.648752

G(298 K)= -2474.813100

Lowest Frequency = -100.4721cm-1

Al	4.627719	10.118181	13.344037
Zn	7.091782	9.174292	14.635916
Zn	6.700368	10.630880	12.344611
N	3.709340	8.420648	13.337927
C	3.301674	7.861168	14.483281
C	3.296002	8.550446	15.710880
H	3.004494	7.966521	16.576939
C	3.419724	9.932007	15.919255
N	3.801101	10.778812	14.953058
C	2.793876	6.451595	14.481916
H	2.063245	6.296711	13.682001
H	2.336870	6.194169	15.437644
H	3.612684	5.748426	14.295086
C	3.091079	10.475740	17.276473
H	2.329304	11.258991	17.223060
H	3.981020	10.943015	17.714657
H	2.746168	9.690825	17.950085
C	3.556828	7.708917	12.092791
C	2.525253	8.099536	11.207811
C	2.385854	7.407661	10.003006
H	1.595756	7.690756	9.315066
C	3.241708	6.368455	9.660841
H	3.115143	5.844740	8.717579
C	4.268429	6.021373	10.522626
H	4.962151	5.232013	10.242654
C	4.450563	6.678765	11.743613
C	1.570882	9.226055	11.551739
H	2.165341	10.020306	12.033497
C	0.912985	9.845051	10.324128
H	1.652433	10.135511	9.571718
H	0.203544	9.156749	9.852493
H	0.349413	10.738059	10.603979
C	0.506684	8.769265	12.552257
H	0.938206	8.467123	13.512052
H	-0.208927	9.574190	12.753824
H	-0.056376	7.918579	12.151508
C	5.661979	6.310834	12.573283
H	5.603789	6.820865	13.546823
C	5.761470	4.805923	12.819357
H	6.610883	4.572830	13.468783
H	4.860469	4.399351	13.290753
H	5.915810	4.255647	11.885288
C	6.914595	6.821723	11.862226
H	7.811998	6.650339	12.462361
H	7.045746	6.318241	10.896086
H	6.847329	7.897848	11.663248
C	3.575964	12.191638	15.123022
C	4.570121	13.020441	15.673057
C	4.320681	14.393984	15.756031
H	5.091596	15.044552	16.163080
C	3.113350	14.937084	15.343124
H	2.936133	16.005646	15.425736
C	2.130755	14.102066	14.828554
H	1.177276	14.523033	14.518150
C	2.341936	12.728236	14.695883
C	5.892172	12.491874	16.186154
H	5.863526	11.391607	16.161381
C	6.128011	12.935941	17.630560
H	5.310676	12.628198	18.291695
H	7.056448	12.515654	18.028008
H	6.213471	14.025231	17.704359
C	7.042190	12.939991	15.286689
H	6.886022	12.623743	14.247723
H	7.138313	14.032900	15.284017
H	7.993449	12.516645	15.625104
C	1.232894	11.862606	14.132636
H	1.653352	10.872876	13.917911
C	0.680156	12.429593	12.828196
H	-0.110225	11.782346	12.434399
H	0.235900	13.419974	12.970716
H	1.459964	12.520602	12.064619
C	0.107659	11.667795	15.149717
H	-0.688531	11.041761	14.732585
H	0.461945	11.184763	16.065763
H	-0.337454	12.628787	15.431933
C	9.315705	8.487496	15.582998
C	8.562220	7.296292	15.396075
C	7.394027	7.379951	16.205548
C	7.436329	8.622938	16.910817
C	8.628623	9.303174	16.524718
C	10.686559	8.747031	15.050933
H	10.882451	8.195664	14.125378
H	10.861716	9.807704	14.836469
H	11.463808	8.443470	15.767252
C	9.037433	6.102522	14.638617
H	8.216769	5.433279	14.362026
H	9.562741	6.373348	13.714372

H	9.743686	5.503102	15.230300
C	6.348491	6.326246	16.372794
H	6.325990	5.635254	15.521994
H	6.510789	5.714346	17.271550
H	5.343194	6.758408	16.463257
C	6.448984	9.060591	17.941950
H	6.426722	10.151120	18.057509
H	5.430540	8.739166	17.688004
H	6.670942	8.644582	18.935060
C	9.177414	10.559768	17.110170
H	8.398631	11.170755	17.575375
H	9.924081	10.354752	17.890565
H	9.679379	11.184150	16.359942
C	8.534615	10.299039	11.215548
C	8.583096	11.577040	11.890950
C	7.687195	12.463201	11.199387
C	7.102497	11.749493	10.124189
C	7.601330	10.420423	10.134714
C	9.432598	9.148472	11.534694
H	9.301544	8.795829	12.569096
H	10.491027	9.420114	11.425325
H	9.244689	8.292541	10.879380
C	9.548274	11.912454	12.980907
H	9.342808	12.892820	13.421507
H	10.585001	11.929109	12.618008
H	9.508776	11.177519	13.797913
C	7.412923	13.891348	11.543850
H	7.716719	14.568111	10.735633
H	7.955573	14.202417	12.441609
H	6.348235	14.081603	11.730289
C	6.026832	12.256319	9.223436
H	5.020982	11.966726	9.565069
H	6.133603	11.866257	8.204890
H	6.036956	13.348960	9.158346
C	7.200641	9.347889	9.173795
H	7.795200	8.439081	9.307080
H	7.333366	9.672440	8.134525
H	6.145548	9.057491	9.277672

Ga-TS1.log

SCF (wB97x) = -2475.85310109
E(SCF)+ZPE(0 K)= -2474.771189
H(298 K)= -2474.704782
G(298 K)= -2474.868773
Lowest Frequency = -96.8484cm-1

Zn	7.056259	9.206430	14.594041
Zn	6.623423	10.719782	12.122403
N	3.685809	8.403354	13.329250
C	3.310964	7.865058	14.490719
C	3.326095	8.564291	15.714100
H	3.052386	7.979910	16.585823
C	3.434171	9.947452	15.925681
N	3.798919	10.811125	14.973746
C	2.807012	6.451386	14.519059
H	2.055453	6.284986	13.741061
H	2.375233	6.203722	15.489123
H	3.623001	5.748354	14.319263
C	3.102760	10.477690	17.290530
H	2.323047	11.243848	17.246165
H	3.984577	10.962065	17.726717
H	2.777957	9.681742	17.961399
C	3.513075	7.682688	12.099389
C	2.462960	8.059486	11.228982
C	2.309279	7.360433	10.030048
H	1.504151	7.632136	9.354826
C	3.169851	6.328538	9.676826
H	3.031877	5.799271	8.738186
C	4.214591	5.994848	10.522479
H	4.910230	5.209776	10.234762
C	4.409913	6.658523	11.737876
C	1.507523	9.181530	11.585277
H	2.108196	9.983302	12.044074
C	0.808864	9.778468	10.369165
H	1.521498	10.064484	9.589726
H	0.091692	9.077469	9.928600
H	0.245734	10.670610	10.653351
C	0.472540	8.730595	12.618659
H	0.930681	8.441152	13.569700
H	-0.240080	9.535918	12.830120
H	-0.098253	7.873782	12.242410
C	5.633848	6.303043	12.554549
H	5.580054	6.812344	13.528336
C	5.751121	4.799543	12.800409
H	6.607647	4.576277	13.444173
H	4.857394	4.385262	13.278853
H	5.904224	4.249742	11.865753

C	6.875087	6.826768	11.834083
H	7.781780	6.640375	12.415538
H	6.989282	6.344726	10.854912
H	6.806094	7.908247	11.664284
C	3.557293	12.214315	15.148600
C	4.543030	13.051426	15.702231
C	4.279571	14.421576	15.793288
H	5.042379	15.078471	16.205597
C	3.067034	14.953963	15.380214
H	2.878100	16.020094	15.469096
C	2.094514	14.112064	14.856977
H	1.138030	14.525607	14.545379
C	2.319685	12.741074	14.718501
C	5.870784	12.526177	16.203664
H	5.836362	11.426196	16.196259
C	6.134115	12.987948	17.637123
H	5.321446	12.699224	18.312452
H	7.061909	12.558996	18.026978
H	6.236077	14.076738	17.696256
C	7.007081	12.950241	15.275469
H	6.843134	12.587430	14.252262
H	7.092337	14.043093	15.228554
H	7.965566	12.548407	15.619455
C	1.223337	11.862359	14.150555
H	1.660796	10.882073	13.926115
C	0.654903	12.428406	12.852669
H	-0.126104	11.769534	12.459266
H	0.194167	13.410261	13.002818
H	1.428441	12.536390	12.084991
C	0.105602	11.640819	15.170556
H	-0.684070	11.008188	14.750846
H	0.471254	11.152786	16.079387
H	-0.351201	12.592610	15.465257
C	9.316847	8.519924	15.552726
C	8.565827	7.328861	15.359028
C	7.396800	7.406214	16.167537
C	7.434357	8.647444	16.875491
C	8.624634	9.332081	16.492371
C	10.691706	8.779263	15.031414
H	10.889369	8.242298	14.097596
H	10.875186	9.842285	14.837033
H	11.463450	8.457613	15.746068
C	9.043345	6.141803	14.592341
H	8.225264	5.465953	14.323488

H	9.554400	6.420981	13.662020
H	9.763221	5.546226	15.171691
C	6.357505	6.346617	16.336608
H	6.338260	5.653894	15.487030
H	6.523860	5.736670	17.236173
H	5.349877	6.773183	16.427229
C	6.450246	9.076307	17.913379
H	6.432811	10.165602	18.042516
H	5.430001	8.762373	17.657745
H	6.672558	8.647679	18.901261
C	9.167219	10.588727	17.083737
H	8.384073	11.194548	17.548755
H	9.912916	10.385466	17.865780
H	9.668553	11.218742	16.337363
C	8.603598	10.283925	11.209982
C	8.656762	11.547891	11.886019
C	7.766986	12.449491	11.204756
C	7.174513	11.743361	10.116163
C	7.671473	10.404975	10.123762
C	9.433282	9.100947	11.581322
H	9.219732	8.753639	12.604517
H	10.504462	9.337821	11.547203
H	9.262540	8.253808	10.910424
C	9.537827	11.839737	13.054637
H	9.380198	12.850297	13.443209
H	10.600628	11.750960	12.794059
H	9.354650	11.138758	13.883775
C	7.545440	13.890424	11.527542
H	8.084744	14.549413	10.835477
H	7.886560	14.138040	12.537142
H	6.486940	14.168139	11.468367
C	6.183078	12.293891	9.147259
H	5.478225	11.525995	8.809584
H	6.663235	12.700586	8.247360
H	5.594018	13.106062	9.586367
C	7.314433	9.350068	9.126980
H	7.774327	8.389170	9.374090
H	7.647928	9.619385	8.117287
H	6.231684	9.178821	9.066468
Ga	4.668368	10.167149	13.285132

Si-TS-1.log

SCF (wB97x) = -2762.64320249

E(SCF)+ZPE(0 K)= -2761.570013

H(298 K)= -2761.505812

G(298 K)= -2761.662659

Lowest Frequency = -124.7397cm-1

Si	3.377309	11.945249	2.139517
Zn	2.717179	11.830102	4.679244
Zn	2.389489	9.987669	2.731625
N	2.422547	13.392025	1.755050
C	2.836930	14.605890	2.304042
C	4.048325	14.756606	2.917527
H	4.232463	15.723747	3.374595
C	5.174955	13.840737	2.969422
N	4.999946	12.580658	2.357569
C	1.891389	15.761870	2.220357
H	1.552211	15.929120	1.191648
H	2.370660	16.674761	2.577332
H	0.990350	15.592751	2.821131
C	6.357430	14.210433	3.525338
H	7.215611	13.547994	3.540879
H	6.477210	15.210847	3.925175
C	1.239979	13.290911	0.935948
C	1.383243	13.234282	-0.473769
C	0.235258	13.118673	-1.260817
H	0.330895	13.077515	-2.340791
C	-1.031029	13.059592	-0.693245
H	-1.910263	12.966821	-1.324714
C	-1.159047	13.108921	0.683437
H	-2.147613	13.040564	1.131417
C	-0.042459	13.223240	1.520217
C	2.737931	13.359112	-1.143518
H	3.454310	12.754798	-0.564145
C	2.749697	12.837567	-2.576446
H	3.769333	12.828415	-2.967612
H	2.351752	11.820500	-2.648092
H	2.160924	13.476431	-3.243684
C	3.218086	14.813493	-1.114232
H	4.159341	14.924445	-1.662496
H	2.478736	15.467901	-1.590764
H	3.387606	15.173762	-0.095668
C	-0.295908	13.222638	3.015331
H	0.648008	13.445718	3.536690
C	-1.342005	14.267475	3.409077
H	-1.051302	15.278886	3.108024
H	-1.505739	14.269738	4.489452
H	-2.308603	14.057147	2.940601
C	-0.754181	11.834662	3.460127
H	-1.699775	11.560653	2.975867
H	-0.909005	11.790489	4.543253
H	-0.015314	11.069078	3.196495
C	6.163254	11.834479	1.959164
C	6.702319	10.829822	2.786027
C	7.789798	10.083471	2.317491
H	8.188474	9.284609	2.939155
C	8.373260	10.349041	1.089184
H	9.222104	9.763980	0.745918
C	7.871649	11.382182	0.308619
H	8.345610	11.612218	-0.642620
C	6.766773	12.132345	0.715857
C	6.170959	10.516833	4.168725
H	5.417587	11.275582	4.431046
C	7.285995	10.571666	5.212331
H	6.902667	10.307250	6.203060
H	7.731689	11.568894	5.277989
H	8.085538	9.860462	4.980166
C	5.504109	9.143948	4.189756
H	4.721402	9.066936	3.427156
H	5.052857	8.930776	5.164474
H	6.233026	8.351419	3.977558
C	6.286800	13.261318	-0.169687
H	5.297526	13.570366	0.187788
C	6.157620	12.817061	-1.623782
H	5.501503	11.945291	-1.723428
H	5.752238	13.623891	-2.242820
H	7.128748	12.550487	-2.053832
C	7.213257	14.473064	-0.058466
H	7.303216	14.807283	0.979402
H	8.217355	14.230412	-0.426768
H	6.831512	15.308417	-0.655978
C	1.779022	11.481458	6.743263
C	1.599286	12.865826	6.437985
C	2.888543	13.471952	6.371050
C	3.864498	12.480413	6.678694
C	3.185635	11.247514	6.899334
C	0.702331	10.480674	7.004686
H	-0.200823	10.678914	6.416475
H	0.397223	10.470611	8.059997
H	1.028087	9.462345	6.763313

C	0.315418	13.620287	6.416520
H	0.339085	14.449820	5.701029
H	0.103469	14.063397	7.399260
H	-0.542789	12.988400	6.168388
C	3.166242	14.924440	6.176766
H	4.183924	15.087982	5.807648
H	3.063611	15.485564	7.116438
H	2.482950	15.383805	5.453402
C	5.319255	12.764236	6.855630
H	5.866150	11.880211	7.190416
H	5.472919	13.540363	7.616084
H	5.800190	13.125285	5.936616
C	3.791043	9.961861	7.356798
H	3.359943	9.090642	6.849120
H	3.638246	9.801868	8.432507
H	4.871264	9.935407	7.181479
C	2.309740	7.842103	2.659912
C	2.986356	7.981359	1.407701
C	2.121462	8.633048	0.499161
C	0.898052	8.914921	1.148708
C	0.979484	8.429469	2.497751
C	2.718664	6.990527	3.818158
H	3.805969	6.893197	3.891974
H	2.308032	5.975688	3.732008
H	2.365051	7.394767	4.773426
C	4.397633	7.592788	1.110600
H	5.032009	8.463001	0.888927
H	4.454428	6.935714	0.234895
H	4.861042	7.059766	1.945974
C	2.530955	9.155858	-0.834718
H	1.668571	9.373242	-1.472876
H	3.175914	8.453452	-1.373379
H	3.102606	10.095375	-0.734088
C	-0.275452	9.598973	0.529830
H	-0.999113	9.929521	1.281110
H	-0.811986	8.936330	-0.161449
H	0.018514	10.486322	-0.042848
C	-0.161906	8.215429	3.438798
H	0.140919	8.304470	4.487415
H	-0.593585	7.213172	3.315517
H	-0.969898	8.934340	3.271809

3.log

SCF (wB97x) = SCF

E(SCF)+ZPE(0 K)= -3244.592664

H(298 K)= -3244.499676

G(298 K)= -3244.723902

Lowest Frequency = 8.5733cm-1

In	6.510783	4.413000	8.219749
Zn	8.074884	2.609398	7.405086
N	5.833849	3.658201	10.163434
C	6.689287	3.557391	11.189561
C	7.950610	4.210050	11.229991
H	8.577908	3.955707	12.087250
C	8.388081	5.326213	10.474662
N	7.790434	5.761171	9.348992
C	6.285070	2.745895	12.408948
H	5.419960	3.216112	12.908831
H	7.108862	2.673824	13.133440
H	5.969302	1.729048	12.120770
C	9.579470	6.101237	11.015712
H	10.323218	6.275699	10.219895
H	10.059167	5.562949	11.846012
H	9.265933	7.095140	11.380047
C	4.505847	3.137179	10.301838
C	3.481812	3.972771	10.817559
C	2.186459	3.443439	10.956635
H	1.398205	4.083123	11.371867
C	1.881780	2.116185	10.598235
C	2.917781	1.315518	10.085575
H	2.704039	0.278958	9.800774
C	4.225592	1.805122	9.911642
C	3.780506	5.403726	11.192183
H	4.681858	5.493211	11.821748
H	2.930129	5.866706	11.716999
H	3.968537	5.995114	10.276358
C	0.468231	1.589039	10.707072
H	-0.066665	2.030397	11.564848
H	0.449099	0.491749	10.813806
H	-0.112679	1.842034	9.800053
C	5.308033	0.941344	9.309726
H	5.498469	1.226082	8.258764
H	5.018348	-0.120994	9.312049
H	6.270529	1.050209	9.835433
C	8.115541	7.081819	8.895927
C	9.090606	7.288148	7.892621

C	9.498446	8.608572	7.618123	Zn	2.171649	6.402769	7.988346
H	10.284126	8.771837	6.870612	N	4.702000	6.861106	5.329272
C	8.937619	9.714278	8.279984	C	3.828390	6.879293	4.304274
C	7.888902	9.481550	9.190646	C	2.920752	5.831716	4.010490
H	7.404440	10.334398	9.682539	H	2.243007	6.026104	3.176177
C	7.462804	8.183232	9.508366	C	2.953922	4.484031	4.460247
C	9.680229	6.114434	7.143336	N	3.640911	4.078577	5.536197
H	9.833400	5.239404	7.797028	C	3.838016	8.069602	3.357948
H	10.637822	6.378122	6.666031	H	4.768047	8.088281	2.763277
H	8.992314	5.783571	6.341643	H	2.984585	8.032762	2.665403
C	9.420705	11.125625	8.028271	H	3.804266	9.015808	3.923974
H	8.666927	11.714738	7.476170	C	2.202588	3.454198	3.633571
H	10.350442	11.140909	7.437487	H	1.519016	2.862678	4.265752
H	9.610964	11.655945	8.977726	H	1.624570	3.929913	2.828412
C	6.339783	7.939031	10.488819	H	2.911953	2.739510	3.180412
H	5.900615	8.886244	10.840120	C	5.784782	7.800302	5.291865
H	6.671133	7.361438	11.370462	C	6.913862	7.492654	4.488332
H	5.544395	7.338812	10.013311	C	7.908767	8.467484	4.321306
C	9.342892	1.027607	6.492927	H	8.770610	8.241961	3.680634
C	8.815882	0.400811	7.687817	C	7.816110	9.732406	4.933329
C	9.403152	1.053198	8.821463	C	6.732286	9.973974	5.794420
C	10.293616	2.073979	8.344252	H	6.663698	10.936784	6.314741
C	10.270881	2.060017	6.909901	C	5.718739	9.018647	6.006730
C	9.121708	0.551256	5.080078	C	7.020540	6.137252	3.829401
H	8.132384	0.076375	4.968562	H	6.185684	5.942505	3.132484
H	9.878117	-0.197607	4.772929	H	7.966350	6.034759	3.274042
H	9.172721	1.381860	4.355462	H	6.973466	5.339244	4.590977
C	7.872571	-0.774217	7.697945	C	8.876329	10.778987	4.670487
H	7.452697	-0.946372	8.701977	H	9.083377	10.874258	3.590315
H	8.374615	-1.709283	7.383542	H	8.576664	11.769232	5.049197
H	7.019141	-0.616339	7.016587	H	9.830401	10.512024	5.159194
C	9.138924	0.768945	10.276990	C	4.587118	9.283660	6.973915
H	8.823346	1.682384	10.813625	H	3.633110	8.853790	6.625611
H	10.042299	0.386911	10.790191	H	4.802227	8.813767	7.952946
H	8.345386	0.014893	10.408897	H	4.450881	10.362582	7.152495
C	11.081737	2.998499	9.235078	C	3.772806	2.676155	5.799855
H	11.521968	3.836444	8.669249	C	2.818097	2.017538	6.612845
H	11.914680	2.474322	9.742656	C	2.960078	0.634587	6.829786
H	10.441387	3.430538	10.025144	H	2.210727	0.121029	7.442911
C	11.109484	2.900400	5.981714	C	4.034568	-0.099578	6.297211
H	10.561720	3.152216	5.057173	C	4.980288	0.587258	5.512075
H	12.037494	2.378719	5.677118	H	5.819145	0.033528	5.073036
H	11.408563	3.851182	6.452974	C	4.870458	1.964911	5.250688
In	4.335806	5.541111	7.017976	C	1.684848	2.790838	7.243661

H	0.883070	2.117310	7.584622
H	2.038849	3.350070	8.128760
H	1.255868	3.540523	6.559166
C	4.199954	-1.571815	6.602458
H	4.829049	-1.715996	7.501016
H	3.230281	-2.058301	6.800056
H	4.694125	-2.107039	5.774303
C	5.900218	2.679545	4.410339
H	6.591477	1.967336	3.932974
H	5.439946	3.309875	3.630744
H	6.503278	3.350717	5.050200
C	0.404075	7.173315	9.108045
C	0.789341	8.264156	8.237461
C	0.522877	7.863121	6.885887
C	-0.037721	6.540864	6.909125
C	-0.117669	6.108946	8.275263
C	0.369791	7.208651	10.614288
H	1.203217	7.801589	11.028312
H	-0.569822	7.653952	10.996367
H	0.443449	6.193661	11.039808
C	1.315330	9.599130	8.697814
H	1.906030	10.096079	7.910612
H	0.498005	10.290811	8.980058
H	1.969851	9.494577	9.580474
C	0.794468	8.653983	5.632912
H	1.249949	8.017635	4.853360
H	-0.130517	9.085343	5.203127
H	1.486816	9.492030	5.819987
C	-0.446201	5.764947	5.684165
H	-0.703645	4.721133	5.929157
H	-1.330093	6.212357	5.190121
H	0.366569	5.738826	4.935851
C	-0.706710	4.820561	8.789258
H	-0.062077	4.354680	9.553868
H	-1.701821	4.975729	9.248554
H	-0.828012	4.081917	7.980704

2d.log

SCF (wB97x) = SCF

E(SCF)+ZPE(0 K)= -2239.462938

H(298 K)= -2239.400520

G(298 K)= -2239.561065

Lowest Frequency = 15.5144cm-1

Zn	7.421078	8.723221	15.554042
Zn	5.913450	11.103620	11.693253
N	4.437788	8.121106	13.487466
C	3.553409	7.782704	14.435380
C	3.260311	8.583905	15.572883
H	2.577518	8.130908	16.295398
C	3.536511	9.960878	15.784848
N	4.403860	10.669683	15.050006
C	2.780965	6.484714	14.271475
C	2.776335	10.674613	16.889894
C	4.518277	7.395472	12.254492
C	3.679064	7.775911	11.175417
C	3.832106	7.127052	9.937241
H	3.177998	7.417110	9.105492
C	4.798266	6.122102	9.736957
C	5.613367	5.763182	10.825474
H	6.365959	4.975406	10.695585
C	5.495579	6.383607	12.084161
C	2.677501	8.892341	11.349427
H	3.211002	9.855170	11.456342
C	6.404706	5.990431	13.225984
H	5.865134	5.951870	14.185676
C	4.418083	12.099687	15.091552
C	5.414375	12.791646	15.821944
C	5.459606	14.197286	15.736658
H	6.229468	14.734182	16.304936
C	4.553903	14.927313	14.947470
C	3.567989	14.212733	14.237631
H	2.846398	14.762525	13.620317
C	3.487640	12.810746	14.288198
C	6.422199	12.039602	16.661513
H	5.993176	11.121362	17.092558
C	2.478111	12.049985	13.463007
H	3.010041	11.422608	12.724784
C	8.945423	7.908285	16.954361
C	8.148274	6.755553	16.589332
C	6.839031	6.931863	17.147666
C	6.818124	8.176116	17.866782
C	8.116250	8.779771	17.762101
C	10.425816	8.068805	16.718438
H	10.739186	7.595070	15.772730
H	10.715565	9.132151	16.670535
H	11.022203	7.604758	17.528584

C	8.664408	5.555786	15.838015
H	7.841336	4.930202	15.457511
H	9.280559	5.847744	14.969803
H	9.296049	4.913407	16.481398
C	5.659278	6.002694	17.030716
H	5.868898	5.156363	16.356163
H	5.379146	5.575163	18.012629
H	4.768409	6.529684	16.642679
C	5.618035	8.707093	18.605952
H	5.753534	9.757847	18.911526
H	4.709045	8.656225	17.980402
H	5.412456	8.124936	19.525199
C	8.596504	10.032605	18.447711
H	7.755234	10.651350	18.798362
H	9.222426	9.798072	19.330154
H	9.206875	10.661268	17.776319
C	6.718636	11.278598	9.516764
C	7.329989	12.324476	10.295600
C	6.283817	13.186740	10.784505
C	5.023487	12.678692	10.296366
C	5.294112	11.496645	9.512769
C	7.428037	10.163572	8.792638
H	8.387786	9.911005	9.274117
H	7.651217	10.430235	7.741362
H	6.816045	9.245415	8.771973
C	8.807082	12.496965	10.538919
H	9.004122	12.990222	11.505832
H	9.285063	13.115205	9.754923
H	9.332236	11.526751	10.551148
C	6.457686	14.428455	11.621824
H	6.472455	15.345776	11.002078
H	7.402787	14.402986	12.189439
H	5.640257	14.537133	12.355340
C	3.684768	13.341614	10.479067
H	2.859561	12.610945	10.451990
H	3.489151	14.089438	9.686389
H	3.628901	13.865519	11.447219
C	4.288457	10.678343	8.746619
H	4.481352	9.596342	8.846874
H	4.306440	10.917832	7.666050
H	3.263035	10.860473	9.106428
H	3.467926	5.641559	14.085849
H	2.111042	6.546759	13.395795
H	2.171863	6.261994	15.159346
H	2.189950	9.970622	17.497994
H	3.476614	11.219910	17.545669
H	2.087359	11.425479	16.465158
H	6.814406	12.669082	17.476565
H	7.286597	11.706637	16.056570
H	1.864460	11.363968	14.071696
H	1.806249	12.731354	12.919081
H	2.064134	8.770801	12.257997
H	2.007974	8.970359	10.478715
H	6.876254	5.011559	13.044053
H	7.214219	6.729842	13.372478
C	4.969054	5.471096	8.380425
H	3.999558	5.335597	7.871647
H	5.597668	6.094512	7.717963
H	5.455518	4.485039	8.461415
C	4.657318	16.432483	14.822830
H	5.196926	16.875480	15.676010
H	5.205912	16.713077	13.904296
H	3.662004	16.904380	14.761558
In	5.903746	9.714501	13.777267

1d.log

SCF (wB97x) = SCF

E(SCF)+ZPE(0 K)= -1005.061168

H(298 K)= -1005.030247

G(298 K)= -1005.124215

Lowest Frequency = 10.3855cm-1

In	4.025000	7.872075	6.706706
N	4.259581	9.394098	8.385906
N	4.116066	9.754490	5.425844
C	4.377264	10.733438	8.329633
C	4.374114	11.482022	7.129215
H	4.479371	12.561982	7.255723
C	4.253669	11.042739	5.789825
C	4.523937	11.511645	9.629795
H	3.657089	11.336771	10.289881
H	4.613026	12.592236	9.446619
H	5.413813	11.174619	10.188561
C	4.284265	12.111351	4.705947
H	5.117636	11.932907	4.004923
H	4.395285	13.118310	5.133558
H	3.359873	12.084177	4.103954

C	4.274457	8.755373	9.671417
C	3.052161	8.519220	10.350561
C	3.085037	7.838529	11.581107
H	2.141555	7.661883	12.112669
C	4.289588	7.379365	12.146655
C	5.485835	7.619185	11.444647
H	6.435600	7.269507	11.868400
C	5.502351	8.295613	10.211240
C	1.746209	8.997934	9.755442
H	0.897413	8.768367	10.418823
H	1.553166	8.520765	8.776704
H	1.754455	10.086469	9.569396
C	4.293659	6.617167	13.455409
H	3.470627	6.940100	14.114825
H	5.243233	6.751800	13.999882
H	4.166874	5.531939	13.284524
C	6.797839	8.537582	9.468263
H	6.790530	8.043992	8.478724
H	7.660595	8.152139	10.034207
H	6.964653	9.611773	9.273600
C	4.001286	9.443148	4.029135
C	2.717202	9.360167	3.433440
C	2.624716	8.996579	2.077631
H	1.632735	8.940000	1.612174
C	3.764724	8.704409	1.305397
C	5.025262	8.784227	1.926907
H	5.926376	8.559971	1.342418
C	5.167057	9.143179	3.279591
C	1.479527	9.664045	4.248696
H	1.378347	8.964571	5.099249
H	0.568284	9.587072	3.634765
H	1.515675	10.676872	4.687489
C	3.636664	8.280745	-0.143026
H	2.741811	8.718948	-0.615812
H	3.546208	7.181996	-0.229981
H	4.518968	8.581444	-0.732578
C	6.530390	9.215076	3.931498
H	7.330779	8.992421	3.208285
H	6.614126	8.493995	4.765717
H	6.725443	10.211173	4.366521

In-TS-1.log

SCF (wB97x) = SCF

E(SCF)+ZPE(0 K)= -2239.427482

H(298 K)= -2239.365610

G(298 K)= -2239.525623

Lowest Frequency = -79.5501cm-1

Zn	6.948398	9.326605	14.862827
Zn	6.986154	11.052917	12.490480
N	3.718438	8.555123	13.301091
C	3.199560	7.975439	14.397397
C	3.053807	8.627519	15.648516
H	2.672750	7.996179	16.454037
C	3.153389	10.005720	15.966629
N	3.667331	10.937551	15.143130
C	2.728860	6.536491	14.290486
H	1.884115	6.450940	13.585691
H	2.410343	6.144727	15.266831
H	3.539020	5.900298	13.894801
C	2.638227	10.456503	17.321487
H	1.765704	11.122544	17.209778
H	3.414442	11.035045	17.850684
H	2.344733	9.599299	17.943665
C	3.606557	7.916264	12.018717
C	2.409943	8.084124	11.276015
C	2.323877	7.504038	9.998199
H	1.399818	7.631449	9.420884
C	3.390996	6.773647	9.440400
C	4.567599	6.634084	10.198730
H	5.412277	6.072305	9.781979
C	4.703444	7.203112	11.478990
C	3.503653	12.334060	15.440063
C	4.571063	13.071994	16.006125
C	4.383323	14.447017	16.243744
H	5.202898	15.019242	16.695891
C	3.181891	15.103370	15.920230
C	2.146582	14.345337	15.339387
H	1.204861	14.839623	15.070505
C	2.285568	12.969410	15.086786
C	5.886634	12.403480	16.330515
H	5.748721	11.395762	16.754736
C	1.179565	12.177580	14.425970
H	1.544627	11.701348	13.497891
C	8.971543	8.647145	15.981655
C	8.288596	7.443168	15.582161
C	7.043817	7.386533	16.295202

C	6.966959	8.539137	17.156514
C	8.156205	9.317632	16.961648
C	10.353485	9.080379	15.565452
H	10.602772	8.747562	14.543987
H	10.453317	10.179668	15.586265
H	11.135329	8.675527	16.238303
C	8.818277	6.377067	14.658430
H	8.048865	6.020644	13.951414
H	9.666305	6.744305	14.057999
H	9.175716	5.491489	15.219108
C	6.022990	6.281370	16.220253
H	6.108073	5.716098	15.276245
H	6.134658	5.550180	17.045414
H	4.994966	6.680181	16.274333
C	5.852613	8.812165	18.131788
H	5.803003	9.877538	18.415124
H	4.872909	8.541721	17.702687
H	5.970343	8.234559	19.069758
C	8.542834	10.578146	17.691828
H	7.689348	11.003117	18.244527
H	9.351410	10.398284	18.426778
H	8.905164	11.361652	17.001915
C	8.644203	9.819359	11.147531
C	8.796333	11.233187	11.550648
C	8.208715	12.036247	10.459449
C	7.682752	11.152796	9.505924
C	7.941129	9.792090	9.931104
C	9.251020	8.671260	11.906691
H	8.920885	8.672838	12.962525
H	10.357219	8.723558	11.910523
H	8.964295	7.697133	11.479486
C	9.935113	11.713521	12.432352
H	9.760741	12.738023	12.800990
H	10.893787	11.718163	11.876716
H	10.062745	11.058595	13.310090
C	8.255313	13.541056	10.408374
H	9.228825	13.909004	10.028693
H	8.115078	13.983133	11.410374
H	7.470852	13.955489	9.753136
C	6.891673	11.498319	8.272519
H	5.885563	11.036968	8.291873
H	7.385152	11.129250	7.353227
H	6.757364	12.586621	8.159350
C	7.454127	8.579091	9.185196
H	7.785844	7.642204	9.662038
H	7.817166	8.571218	8.140171
H	6.349593	8.542564	9.138004
C	6.002269	7.091486	12.240379
H	5.844425	6.952159	13.322676
C	1.271397	8.899177	11.846761
H	1.607961	9.926634	12.077258
H	0.892698	8.480628	12.796004
H	0.429793	8.961220	11.139401
H	6.623795	6.266423	11.857084
H	6.593623	8.021183	12.139000
H	6.498276	12.250895	15.418930
H	6.483985	13.006742	17.032034
H	0.811772	11.356731	15.066763
H	0.323860	12.822160	14.171589
C	3.017008	16.590361	16.154612
H	3.677785	16.949033	16.961032
H	3.268258	17.165718	15.244424
H	1.977702	16.845054	16.421996
C	3.290775	6.190155	8.046740
H	2.250800	5.928142	7.790214
H	3.641468	6.914862	7.288816
H	3.910649	5.284208	7.941640
In	4.807357	10.390898	13.420984

In-INT-1.log

SCF (wB97x) = SCF

E(SCF)+ZPE(0 K)= -2239.450002

H(298 K)= -2239.388428

G(298 K)= -2239.545907

Lowest Frequency = 18.5197cm-1

Zn	6.829972	9.301515	14.687852
Zn	6.978749	10.470390	12.684096
N	3.596641	8.549712	13.083549
C	3.232158	8.008953	14.254633
C	3.138614	8.732498	15.472145
H	2.854146	8.136618	16.342680
C	3.224263	10.127480	15.704749
N	3.570663	11.027792	14.771188
C	2.862163	6.533646	14.294964
H	1.998056	6.338653	13.636377
H	2.608302	6.206246	15.313546

H	3.691120	5.909569	13.921213	H	5.041051	8.882339	17.478373
C	2.872649	10.624751	17.099244	H	6.233362	8.670439	18.785703
H	1.967439	11.255284	17.069651	C	8.839255	10.703526	17.251024
H	3.683144	11.254885	17.502410	H	7.979296	11.345649	17.503335
H	2.693512	9.791438	17.794091	H	9.413438	10.544317	18.184178
C	3.526742	7.760291	11.886156	H	9.488379	11.280690	16.568715
C	2.337577	7.807866	11.110415	C	8.909525	9.810334	11.321846
C	2.297944	7.123004	9.882781	C	9.213730	11.050408	11.980382
H	1.377943	7.160228	9.286159	C	8.222899	12.020069	11.594770
C	3.400996	6.391151	9.403482	C	7.315955	11.379853	10.664280
C	4.557190	6.343533	10.201917	C	7.734900	10.005158	10.517361
H	5.424770	5.767933	9.855813	C	9.647597	8.504192	11.456224
C	4.645896	7.015212	11.436161	H	10.432739	8.561580	12.226746
C	3.432030	12.425738	15.061640	H	10.135045	8.206278	10.509213
C	4.554228	13.184576	15.475764	H	8.969701	7.681090	11.744627
C	4.394471	14.567675	15.684082	C	10.351297	11.335471	12.925976
H	5.260344	15.154047	16.016320	H	9.992988	11.582008	13.942576
C	3.162339	15.215261	15.479836	H	10.955937	12.193561	12.579390
C	2.066368	14.437597	15.058764	H	11.025968	10.471364	13.021689
H	1.096406	14.922848	14.892748	C	8.210434	13.473724	11.993032
C	2.177062	13.052848	14.841562	H	8.817491	14.095372	11.306644
C	5.894017	12.518089	15.682863	H	8.618657	13.613328	13.008187
H	5.797684	11.560534	16.221418	H	7.186853	13.884340	11.988170
C	0.989933	12.238171	14.375382	C	6.247754	12.058450	9.856325
H	1.205134	11.733769	13.414702	H	5.425351	11.363789	9.613575
C	9.178416	8.551545	15.797770	H	6.655493	12.444694	8.902286
C	8.388617	7.417623	15.424346	H	5.803710	12.908821	10.400818
C	7.096013	7.547162	16.036310	C	7.092848	8.992775	9.604677
C	7.092666	8.798372	16.798253	H	7.419598	7.968918	9.848938
C	8.391452	9.405086	16.631326	H	7.348012	9.176862	8.543456
C	10.613954	8.781134	15.409264	H	5.991964	9.006080	9.685005
H	10.823380	8.439095	14.381106	C	5.915794	6.937513	12.248677
H	10.889685	9.846419	15.467198	H	5.717676	6.850427	13.329189
H	11.307760	8.230247	16.073600	C	1.134817	8.579691	11.608769
C	8.841824	6.266277	14.566133	H	1.357008	9.660687	11.692776
H	8.001995	5.823823	14.004398	H	0.825857	8.250247	12.616354
H	9.599648	6.582329	13.829497	H	0.276742	8.462929	10.928222
H	9.294455	5.453693	15.167340	H	6.538508	6.084141	11.935238
C	6.037393	6.479516	16.133050	H	6.522078	7.854707	12.136210
H	6.074202	5.789317	15.273585	H	6.374198	12.267661	14.719026
H	6.166129	5.867487	17.047301	H	6.585382	13.167482	16.243601
H	5.026702	6.917028	16.166709	H	0.731932	11.436024	15.089409
C	6.047832	9.173729	17.816672	H	0.100462	12.872748	14.237275
H	6.032717	10.258973	18.013039	C	3.022786	16.710737	15.674127

H	3.775859	17.100127	16.379141
H	3.158478	17.250474	14.718559
H	2.023159	16.977525	16.056851
C	3.351754	5.697186	8.058533
H	2.332592	5.350912	7.817815
H	3.662935	6.381103	7.247173
H	4.027412	4.826148	8.027143
In	4.147647	10.698363	12.628537

In-TS-2.log

SCF (wB97x) = SCF

E(SCF)+ZPE(0 K)= -3244.545047

H(298 K)= -3244.454130

G(298 K)= -3244.669157

Lowest Frequency = -92.8828cm-1

In	6.135412	4.269177	8.483755
Zn	7.499784	2.340670	7.570502
N	5.553093	3.471628	10.474920
C	6.489152	3.390894	11.431720
C	7.664033	4.185766	11.451368
H	8.358709	3.971447	12.266404
C	7.957502	5.349334	10.697971
N	7.297325	5.718368	9.581470
C	6.282509	2.413599	12.577601
H	5.397970	2.689250	13.176704
H	7.157649	2.383749	13.242881
H	6.097025	1.398590	12.186671
C	9.071532	6.245254	11.212076
H	9.783394	6.487939	10.404964
H	9.614151	5.768332	12.040900
H	8.660685	7.204998	11.571322
C	4.274334	2.863406	10.667529
C	3.289930	3.586882	11.390991
C	1.986382	3.067630	11.460984
H	1.221878	3.627168	12.014168
C	1.637151	1.852227	10.837424
C	2.652977	1.122796	10.194975
H	2.415761	0.146334	9.755075
C	3.976204	1.599115	10.106168
C	3.663546	4.883557	12.069557
H	4.531347	4.763515	12.740835
H	2.825721	5.285393	12.658022
H	3.954748	5.638538	11.319126
C	0.203887	1.368345	10.837649
H	-0.305151	1.600182	11.788494
H	0.139438	0.281101	10.666256
H	-0.370736	1.865600	10.033608
C	5.050174	0.761956	9.451592
H	5.131369	0.982109	8.373707
H	4.824391	-0.312856	9.551313
H	6.044885	0.959053	9.881639
C	7.502063	7.049020	9.083470
C	8.442829	7.297080	8.054375
C	8.685575	8.631879	7.677274
H	9.420453	8.833344	6.888929
C	8.023696	9.710761	8.290619
C	7.037927	9.425717	9.255515
H	6.484696	10.251659	9.720039
C	6.757811	8.109870	9.660895
C	9.175036	6.152082	7.394631
H	9.528354	5.411809	8.131171
H	10.035767	6.507813	6.806584
H	8.497652	5.604538	6.709370
C	8.391960	11.143956	7.974032
H	7.497407	11.784924	7.888867
H	8.960863	11.220557	7.034157
H	9.017885	11.572613	8.778547
C	5.695539	7.816754	10.694914
H	5.235622	8.743363	11.072830
H	6.092596	7.247204	11.553587
H	4.896879	7.193295	10.249480
C	9.061636	1.103555	6.649115
C	8.610173	0.376319	7.830270
C	9.142536	1.047057	8.979129
C	9.927890	2.163127	8.531118
C	9.883907	2.204230	7.104704
C	8.978286	0.626840	5.222151
H	8.101048	-0.019373	5.059030
H	9.873521	0.038281	4.939979
H	8.906316	1.470343	4.515525
C	7.826088	-0.909725	7.808978
H	7.220072	-1.031537	8.722322
H	8.484647	-1.796134	7.728729
H	7.131449	-0.937640	6.953291
C	8.919604	0.683694	10.423647
H	8.673912	1.576156	11.026394

H	9.818198	0.222963	10.878341	H	5.228505	10.035331	7.213562
H	8.092182	-0.036112	10.540809	H	4.050561	10.219123	5.887693
C	10.642907	3.116557	9.451788	C	3.996230	2.860458	5.506195
H	11.128718	3.937209	8.898593	C	3.050704	2.243082	6.366419
H	11.431809	2.603776	10.034649	C	3.180168	0.866917	6.634163
H	9.948046	3.568867	10.181760	H	2.440866	0.396304	7.292981
C	10.599281	3.160329	6.185487	C	4.221294	0.090253	6.101644
H	9.920620	3.576070	5.419126	C	5.168259	0.736339	5.284875
H	11.429868	2.665781	5.645585	H	5.990016	0.153997	4.852858
H	11.026266	4.013185	6.736579	C	5.080849	2.106426	4.979606
In	5.273239	5.589137	6.236618	C	1.893524	2.992464	6.980469
Zn	3.207328	5.798654	8.484163	H	0.925944	2.592284	6.624629
N	4.902873	7.066115	4.724636	H	1.906650	2.879055	8.078490
C	3.910057	7.028697	3.812342	H	1.924194	4.068514	6.759732
C	3.018377	5.947702	3.642544	C	4.337421	-1.381171	6.432323
H	2.240971	6.107015	2.892445	H	4.788219	-1.523682	7.431358
C	3.067277	4.634261	4.182293	H	3.348839	-1.870365	6.449405
N	3.903663	4.253861	5.159119	H	4.973600	-1.913081	5.705786
C	3.775403	8.203720	2.856630	C	6.139720	2.758065	4.118621
H	4.622779	8.222723	2.148810	H	6.696560	2.006467	3.537744
H	2.841708	8.141200	2.279324	H	5.714146	3.500979	3.424730
H	3.796772	9.159853	3.405185	H	6.874657	3.289562	4.758789
C	2.131791	3.605908	3.568773	C	1.457588	6.984362	9.278151
H	1.395925	3.259985	4.314191	C	1.873679	8.071441	8.413999
H	1.586581	4.022379	2.710006	C	1.411417	7.783020	7.101913
H	2.696876	2.718468	3.237044	C	0.694806	6.535732	7.131571
C	5.943764	8.049454	4.585308	C	0.691159	6.050416	8.464327
C	7.030693	7.757560	3.715664	C	1.380891	7.070231	10.783607
C	8.056535	8.705916	3.576122	H	2.300693	7.492251	11.223670
H	8.897867	8.482399	2.908284	H	0.536262	7.709542	11.113764
C	8.027049	9.936003	4.263598	H	1.228414	6.074199	11.229714
C	6.947149	10.185653	5.123853	C	2.595935	9.307512	8.887980
H	6.906477	11.132763	5.672070	H	2.559214	10.109985	8.131781
C	5.914281	9.250431	5.327114	H	2.148221	9.706889	9.817865
C	7.080231	6.439677	2.974348	H	3.665835	9.126051	9.102479
H	6.176080	6.271187	2.363058	C	1.527006	8.653719	5.878648
H	7.959301	6.387746	2.312828	H	2.319450	8.322283	5.186293
H	7.134741	5.591691	3.683215	H	0.583803	8.647599	5.301188
C	9.157314	10.932615	4.118109	H	1.745736	9.702580	6.142621
H	9.536826	10.971094	3.082853	C	0.028056	5.912578	5.932049
H	8.841709	11.948678	4.407227	H	-0.339761	4.895613	6.148745
H	10.012183	10.657731	4.764385	H	-0.842860	6.505591	5.586642
C	4.814265	9.546252	6.316912	H	0.719769	5.839496	5.073493
H	4.286114	8.634428	6.638733	C	-0.069391	4.868201	9.005964

H	0.547128	4.265372	9.695364
H	-0.973057	5.178832	9.567792
H	-0.404480	4.196128	8.198235

In-INT-2.log

SCF (wB97x) = SCF
E(SCF)+ZPE(0 K)= -3244.569723
H(298 K)= -3244.478059
G(298 K)= -3244.696158
Lowest Frequency = 17.5851cm-1

In	5.771225	4.355105	8.922601
Zn	7.091587	2.601847	7.616407
N	5.473664	3.375446	10.894737
C	6.434350	3.283621	11.818131
C	7.589980	4.109069	11.828802
H	8.309839	3.889874	12.620539
C	7.849273	5.291819	11.089339
N	7.161989	5.684365	10.001573
C	6.279313	2.257903	12.928121
H	5.350064	2.436575	13.496302
H	7.128176	2.279607	13.626728
H	6.198878	1.244227	12.498049
C	8.964248	6.192363	11.601375
H	9.643721	6.480783	10.782099
H	9.546492	5.696723	12.391661
H	8.547702	7.127674	12.014769
C	4.224651	2.693050	11.017280
C	3.155132	3.368220	11.667210
C	1.864926	2.818974	11.603434
H	1.037806	3.351315	12.089271
C	1.605882	1.609383	10.926086
C	2.694952	0.923506	10.363973
H	2.525692	-0.048551	9.883248
C	4.007656	1.439261	10.395121
C	3.429130	4.650269	12.416496
H	4.197684	4.507413	13.196541
H	2.518176	5.038131	12.898095
H	3.822500	5.427620	11.739962
C	0.191094	1.089996	10.792448
H	-0.378264	1.211035	11.729931
H	0.172159	0.023901	10.512595
H	-0.354450	1.649053	10.009409
C	5.140523	0.651007	9.778382
H	5.087401	0.677276	8.676037
H	5.093640	-0.408352	10.086227
H	6.124241	1.056868	10.054147
C	7.311512	7.036429	9.541882
C	8.186340	7.357437	8.477524
C	8.298167	8.704937	8.075777
H	8.972293	8.951329	7.246924
C	7.575063	9.733190	8.701784
C	6.689373	9.382377	9.739684
H	6.104237	10.166811	10.235769
C	6.539596	8.053631	10.167718
C	9.052451	6.301841	7.835535
H	10.026724	6.227243	8.353924
H	9.254976	6.544330	6.780484
H	8.596322	5.299804	7.876863
C	7.723295	11.180152	8.283713
H	6.816866	11.545318	7.766182
H	8.578198	11.321003	7.602521
H	7.874514	11.836006	9.158878
C	5.593190	7.702343	11.289671
H	4.996389	8.573896	11.600918
H	6.122497	7.306835	12.175266
H	4.902320	6.902708	10.969458
C	8.695452	1.545285	6.565225
C	8.373979	0.713592	7.725005
C	8.986343	1.314422	8.867734
C	9.698330	2.485707	8.437647
C	9.534790	2.627781	7.029865
C	8.549342	1.135772	5.122185
H	7.637160	0.542084	4.959741
H	9.410341	0.523212	4.787063
H	8.494242	2.014345	4.458672
C	7.676666	-0.619318	7.650534
H	7.196935	-0.885891	8.606662
H	8.379647	-1.436200	7.394545
H	6.889225	-0.610313	6.879300
C	8.911651	0.839512	10.295458
H	8.579599	1.646459	10.974278
H	9.897921	0.496849	10.665309
H	8.211557	-0.004850	10.408897
C	10.460084	3.392360	9.367362
H	11.069779	4.127122	8.816865
H	11.142141	2.814561	10.018868

H	9.784049	3.954504	10.035963
C	10.144810	3.659469	6.118248
H	9.370654	4.260499	5.602995
H	10.766294	3.189128	5.332697
H	10.785787	4.364252	6.671314
In	5.936080	5.327187	5.699166
Zn	3.678713	5.919453	8.746181
N	5.220643	6.864924	4.227231
C	4.080498	6.929762	3.525819
C	3.118921	5.892024	3.499035
H	2.237310	6.093020	2.886044
C	3.167693	4.597686	4.074662
N	4.128340	4.166856	4.913038
C	3.799194	8.177637	2.702613
H	4.552899	8.300500	1.905785
H	2.801957	8.142822	2.239729
H	3.863958	9.075674	3.340764
C	2.051953	3.638243	3.684860
H	1.467241	3.338483	4.570767
H	1.366547	4.092963	2.955126
H	2.471175	2.714423	3.250068
C	6.219106	7.879602	4.071782
C	7.177811	7.749948	3.031625
C	8.213784	8.696492	2.945119
H	8.957724	8.595927	2.144764
C	8.324222	9.761563	3.860446
C	7.371068	9.851725	4.890208
H	7.443648	10.668728	5.615509
C	6.324399	8.920792	5.026250
C	7.089250	6.593429	2.059833
H	6.143749	6.604599	1.488640
H	7.923351	6.611480	1.340609
H	7.111518	5.624627	2.592773
C	9.427260	10.791079	3.728550
H	9.144848	11.593088	3.021328
H	9.648267	11.271845	4.696233
H	10.359582	10.339286	3.349435
C	5.358390	9.000261	6.183624
H	4.337707	8.713190	5.892438
H	5.662027	8.315553	6.999367
H	5.333276	10.011859	6.616885
C	4.118908	2.784065	5.303701
C	3.350177	2.319482	6.401487
C	3.425476	0.959645	6.754019
H	2.824225	0.613937	7.603381
C	4.227905	0.042330	6.055386
C	4.968779	0.523333	4.959447
H	5.576314	-0.178568	4.375182
C	4.934237	1.876401	4.573552
C	2.407337	3.210193	7.171196
H	1.360736	2.883428	7.033143
H	2.609168	3.157345	8.256004
H	2.463566	4.262674	6.858747
C	4.266329	-1.413341	6.469516
H	4.609248	-1.517882	7.514444
H	3.263664	-1.873084	6.411051
H	4.946817	-2.000917	5.832037
C	5.714691	2.358882	3.370785
H	6.242843	1.527901	2.877972
H	5.055485	2.846380	2.630948
H	6.470252	3.113543	3.659138
C	1.939120	7.016470	9.149707
C	2.263765	8.112668	8.215706
C	1.646279	7.830569	6.990604
C	0.899524	6.590310	7.113963
C	1.029176	6.111867	8.421496
C	1.760705	7.272413	10.639122
H	2.643486	7.753927	11.091048
H	0.891079	7.935543	10.822364
H	1.579262	6.332793	11.184151
C	3.034629	9.342196	8.619691
H	3.116533	10.061497	7.788873
H	2.544747	9.865612	9.464463
H	4.064859	9.107707	8.945798
C	1.666623	8.645931	5.725101
H	2.077551	8.069418	4.877021
H	0.643536	8.947830	5.428039
H	2.262980	9.567388	5.828612
C	0.151829	5.956327	5.971132
H	-0.226084	4.953607	6.232002
H	-0.716858	6.565518	5.653211
H	0.800819	5.847622	5.082647
C	0.318691	4.949454	9.061693
H	1.002247	4.316631	9.654752
H	-0.476501	5.291140	9.754304
H	-0.162009	4.299755	8.311483

9) References

[S1] Cui C., Roesky H. W., Schmidt H., Noltemeyer M., Hao H., Cimpoesu F. *Angew. Chem. Int. Ed.*, **2000**, *39*, 4275-4276.

[S2] Hardman N.J.; Eichler B. E.; Philip P. Power. *Chem. Commun.*, **2000**, 1991–1992.

[S3] Driess M.; Yao S.; Brym M.; Wullen C.; Lentz D. *J. Am. Chem. Soc.*, **2006**, *128*, 9628-9629.

[S4] Matthias D.; Shenglai Y.; Markus B; Christoph W. *Angew. Chem. Int. Ed.*, **2006**, *45*, 4349–4352.

[S5] Hicks J.; Juckel M.; Paparo A.; Dange D.; Jones C. *Organometallics*, **2018**, *37*, 4810−4813.

[S6] Hicks J.; Juckel M.; Paparo A.; Dange D.; Jones C. *Organometallics*, **2018**, *37*, 4810−4813.

[S7] Michael S. H.; Peter B. H.; Ruti P. *Angew. Chem. Int. Ed.*, **2005**, *44*, 4231–4235.

[S8] Michael S. H.; Peter B. H. *Chem. Commun.*, **2004**, 1818–1819.

[S9] Michael S. H.; Peter B. H.; Ruti P. *Dalton Trans.*, **2007**, 731–733.

[S10] Resa I.; Carmona E.; Gutierrez-Puebla E.; Monge A. *Science*, **2004**, *305*, 1136−1138.

[S11] Río D.; Galindo A.; Resa I.; Carmona E. *Angew. Chem. Int. Ed.*, **2005**, *44*, 1244–1247.

[S12] Stephan S.; Daniella S.; Ulrich W.; Michael B. *Organometallics*, **2009**, 28, 1590–1592.

[S13] Bin L.; Kevin H.; Christoph W.; Stephan S. *Chem. Commun.*, **2021**, *57*, 13692–13695.

[S14] Martí G.; Clare B.; George A. S.; Andrew J. P. W.; Richard I. C.; Alison J. E.; Mark R. C. *Nature*, **2019**, *574*, 390–393.

[S15] Martí G.; Andrew J. P. W.; Mark R. C. *Chem. Commun.*, **2018**, *54*, 12326–12328.

[S16] Martí G.; Andreas P.; George A. S.; Christopher R.; Andrew J. P. W.; Richard I. C.; Alison J. E.; Mark R. C. *Angew. Chem. Int. Ed.*, **2022**, *44*, e202211948.

[S17] Kerstin F.; Mariusz M.; Paul J.; Katharina D.; Christoph R.; Rudiger W. S.; Christian G.; Gernot F.; Roland A. F. *Chem. Sci.*, **2016**, *7*, 6413–6421.

[S18] Timo B.; Kerstin F.; Christian G.; Rüdiger W. S.; Roland A. F. *Organometallics*, **2011**, 30, 4123–4127.

[S19] SHELXTL v5.1, Bruker AXS, Madison, WI, 1998.

[S20] SHELX-2013, Sheldrick G. M. *Acta Cryst.*, **2015**, *C71*, 3–8.

[S21] Spek A. L. (2003, 2009) PLATON, A Multipurpose Crystallographic Tool, Utrecht University, Utrecht, The Netherlands. See also Spek A. L. *Acta. Cryst.*, 2015, **C71**, 9-18.

[S22] Spek A. L. (2003, 2009) PLATON, A Multipurpose Crystallographic Tool, Utrecht University, Utrecht, The Netherlands. See also Spek A. L. *Acta. Cryst.*, 2015, **C71**, 9-18.

[S23] Frisch, M. J.; Trucks, G. W.; Schlegel, H. B.; Scuseria, G. E.; Robb, M. A.; Cheeseman, J. R.; Scalmani, G.; Barone, V.; Mennucci, B.; Petersson, G. A.; Nakatsuji, H.; Caricato, M.; Li, X.; Hratchian, H. P.; Izmaylov, A. F.; Bloino, J.; Zheng, G.; Sonnenberg, J. L.; Hada, M.; Ehara, M.; Toyota, K.; Fukuda, R.; Hasegawa, J.; Ishida, M.; Nakajima, T.; Honda, Y.; Kitao, O.; Nakai, H.; Vreven, T.; Montgomery, J. A., Jr.; Peralta, J. E.; Ogliaro, F.; Bearpark, M.; Heyd, J. J.; Brothers, E.; Kudin, K. N.; Staroverov, V. N.; Kobayashi, R.; Normand, J.; Raghavachari, K.; Rendell, A.; Burant, J. C.; Iyengar, S. S.; Tomasi, J.; Cossi, M.; Rega, N.; Millam, J. M.; Klene, M.; Knox, J. E.; Cross, J. B.; Bakken, V.; Adamo, C.; Jaramillo, J.; Gomperts, R.; Stratmann, R. E.; Yazyev, O.; Austin, A. J.; Cammi, R.; Pomelli, C.; Ochterski, J. W.; Martin, R. L.; Morokuma, K.; Zakrzewski, V. G.; Voth, G. A.; Salvador, P.; Dannenberg, J. J.; Dapprich, S.; Daniels, A. D.; Farkas, Ö.; Foresman, J. B.; Ortiz, J. V.; Cioslowski, J.; Fox, D. J. Gaussian 09, Revision D.01; Gaussian, Inc., Wallingford, CT, 2009.

[S24] Weigend F; Ahlrichs R. *Phys. Chem. Chem. Phys.* **2005**, *7*, 3297–3305.

[S25] Glendening E. D.; Landis C. R.; Weinhold. F. *J. Comput. Chem.*, **2013**, *34*, 1429–1437.

[S26] Keith T. A.; AIMALL (Version 19.10.12). TK Gristmill Software, Overland Park KS, USA., 2019.

[S27] Cortés-Guzmán F.; Bader R. F. W. *Coord. Chem. Rev.*, **2005**, *249*, 633–662.

[S28] Mitoraj M.P.; Michalak A.; Ziegler T. *J. Chem. Theory Comput.*, **2009**, *5*, 962-975.

[S29] Neese F.; WIREs Comput Mol Sci 2018, 8: e1327.

[S30] Neese F.; Wennmohs F.; Becker U.; Riplinger C. *J. Chem. Phys.*, **2020**, *152*, 1–18.

[S31] Chai J. D.; Head-Gordon M. *J. Chem. Phys.*, **2008**, *128*, 084106.

[S32] Lu T.; Chen Q. *J. Comput. Chem.*, **2022**, *43*, 539–555.

[S33] Lu T.; Chen Q. *J. Comput. Chem.*, **2012**, *33*, 580–592.

[S34] Boto R. A.; Peccati F.; Laplaza R.; Quan C.; Carbone A.; Piquemal J. -P.; Maday Y.; ContrerasGarcía J. *J. Chem. Theory Comput.*, **2020**, *16*, 4150–4158.

[S35] Neese F. *Wiley Interdiscip. Rev. Comput. Mol. Sci.*, **2022**, *12*, e1606.

[S36] Neese F.; Wennmohs F.; Hansen A.; Becker U. *Chem. Phys.*, **2009**, *356*, DOI: 10.1016/j.chemphys.2008.10.036.

[S37] Weigend F. *Phys. Chem. Chem. Phys.*, **2006**, *8*, 1057–1065.

[S38] L. V.; John W.D. *J. Am. Chem. Soc.*, **1962**, *84*, 679-680.
